# Computational Approaches for the Prediction of Environmental Transformation Products: Chlorination of Steroidal Enones

**DOI:** 10.1021/acs.est.1c04659

**Published:** 2021-10-12

**Authors:** Christopher J. Knutson, Nicholas C. Pflug, Wyanna Yeung, Matthew Grobstein, Eric V. Patterson, David M. Cwiertny, James B. Gloer

**Affiliations:** † Department of Chemistry, University of Iowa, Iowa City, Iowa 52242, United States; ‡ Institute of Biogeochemistry and Pollutant Dynamics, ETH Zurich, 8092 Zurich, Switzerland; § Department of Chemistry, Stony Brook University, Stony Brook, New York 11794, United States; ∥ Department of Civil and Environmental Engineering, University of Iowa, Iowa City, Iowa 52242, United States

**Keywords:** Computational Chemistry, Steroids, Prediction, Chlorination, Water Treatment

## Abstract

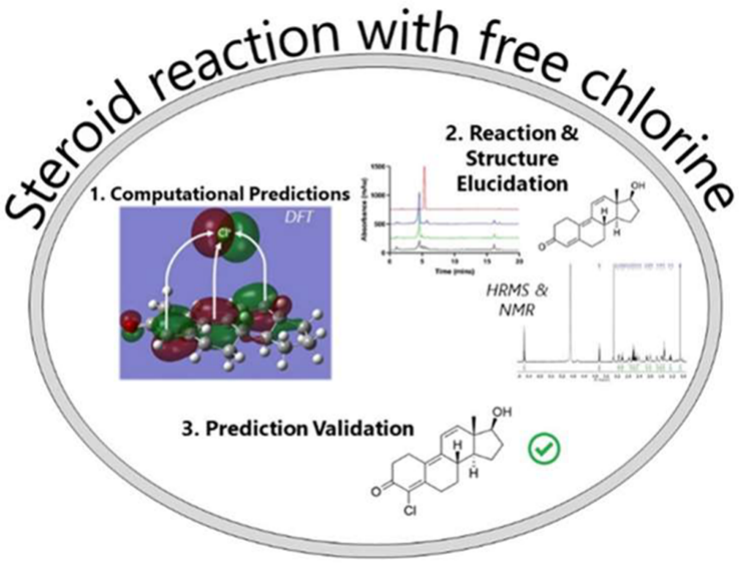

There is growing interest in the fate and effects of transformation products generated from emerging pollutant classes, and new tools that help predict the products most likely to form will aid in risk assessment. Here, using a family of structurally related steroids (enones, dienones, and trienones), we evaluate the use of density functional theory to help predict products from reaction with chlorine, a common chemical disinfectant. For steroidal dienones (e.g., dienogest) and trienones (e.g., 17β-trenbolone), computational data support that reactions proceed through spontaneous C4 chlorination to yield 4-chloro derivatives for trienones and, after further reaction, 9,10-epoxide structures for dienones. For testosterone, a simple steroidal enone, in silico predictions suggest that C4 chlorination is still most likely, but slow at environmentally relevant conditions. Predictions were then assessed through laboratory chlorination reactions (0.5–5 mg Cl_2_/L) with product characterization via HRMS and NMR, which confirmed near exclusive 4-chloro and 9,10-epoxide products for most trienones and all dienones, respectively. Also consistent with computational expectations, testosterone was effectively unreactive at these same chlorine levels, although products consistent with in silico predictions were observed at higher concentrations (in excess of 500 mg Cl_2_/L). Although slight deviations from in silico predictions were observed for steroids with electron-rich substituents (e.g., C17 allyl-substituted altrenogest), this work highlights the potential for computational approaches to improve our understanding of transformation products generated from emerging pollutant classes.

## Abstract 2

To aid risk assessment, computational chemical approaches can be used to predict probable transformation products for common environmental reactions.

## Introduction

Although hazards associated with chemical contaminants are often assumed to be mitigated through their environmental transformation, bioactive and potentially harmful reaction products often persist and can complicate risk assessment for some emerging pollutant classes.^1−5^ A good example are synthetic steroids widely used in medicine and agriculture, for which we have previously demonstrated retained and/or altered bioactivity in products generated from environmental reactions including photolysis in sunlit surface waters and chemical disinfection with free chlorine.^3,4^ Nevertheless, a major challenge associated with bioactive transformation products is that environmental reactions can have a multiplicative effect on the sheer number of species that need to be evaluated for ecological and human health impacts, including many that lack readily available analytical standards. As such, tools that can help predict likely products generated from environmental processes without extensive laboratory investigation will be critical to more comprehensive risk assessment of high priority chemical contaminants.

Given this need, recent attention has focused on whether computational tools can facilitate accurate prediction of environmental transformation products. A popular approach has been the use of rules-based prediction systems based on transformation pathways reported in the literature. For example, the US EPA’s Chemical Transformation Simulator (CTS) generates viable transformation pathways based on user-specified reaction conditions and a library of known pathways (e.g., hydrolysis and reduction) for reactive functional groups.^6^ Yuan et al. also recently extended the capability of CTS by demonstrating that its cheminformatics approach can also predict transformation products (with 40% accuracy) of direct photolysis based on reported reaction pathways.^7^ A similar approach was developed by Wackett et al.^8−15^ for biotransformation pathways. The Biocatalysis/Biodegradation Database, now maintained by the Swiss Federal Institute of Aquatic Science and Technology (EAWAG), offers a rules-based pathway prediction system for microbial degradation of chemical compounds.^11^

A promising alternative to rules-based cheminformatics prediction systems is the use of theory to identify most probable reaction pathways and products based on thermodynamic considerations. For example, Barr et al. used electronic theory to model the biotransformation of 17α-ethinylestradiol (EE2), finding good agreement between frontier electron density modeling and measured metabolites of EE2 under aerobic conditions in mixed culture.^16^ We have similarly used complementary experimental and theoretical approaches to better understand formation of products generated during direct photolysis of trenbolone acetate (TBA) metabolites (i.e., 17α- and 17β-trenbolone). Using a combination of density functional theory (DFT) and multireference molecular orbital theory, we were able to rationalize the formation and stability of experimentally observed phototransformation products across a range of reaction conditions (e.g., from acidic to basic pH).^17^ Such theory-based approaches for transformation product prediction may be valuable when there is insufficient information available for a particular process or specific functional groups to develop a rules-based pathway prediction system.

In this study, we explored the use of in silico modeling for identifying the most probable environmental reaction products generated during the reaction of free chlorine with a family of structurally related steroidal trienones (i.e., 17α-trenbolone, 17β-trenbolone, methyltrenbolone, gestrinone, and altrenogest), dienones (i.e., dienogest, dienedione, and methyldienolone), and enones (i.e., testosterone) (see structures in Figure S1 of the Supporting Information, SI). We focused on chlorination reactions due to the widespread use of free chlorine in chemical disinfection of water and wastewater,^18−20^ the well-recognized ability of chlorination of the steroid ring structure to amplify anabolic activity,^21^ and the limited amount of existing work using computational tools to predict chlorination products from emerging pollutant classes, including aromatic hydrocarbons, various amines, and naproxen.^22−26^ Although other steroid classes (e.g., estrogens^27^ and glucocorticoids^4^) have been extensively investigated, far less is known about the reaction of free chlorine with steroidal enones. Further, based on recent work with α,β-unsaturated carbonyls,^28^ we anticipated that most of these steroidal enones would be reactive toward free chlorine under conditions representative of water treatment and distribution.

**Figure 1:**
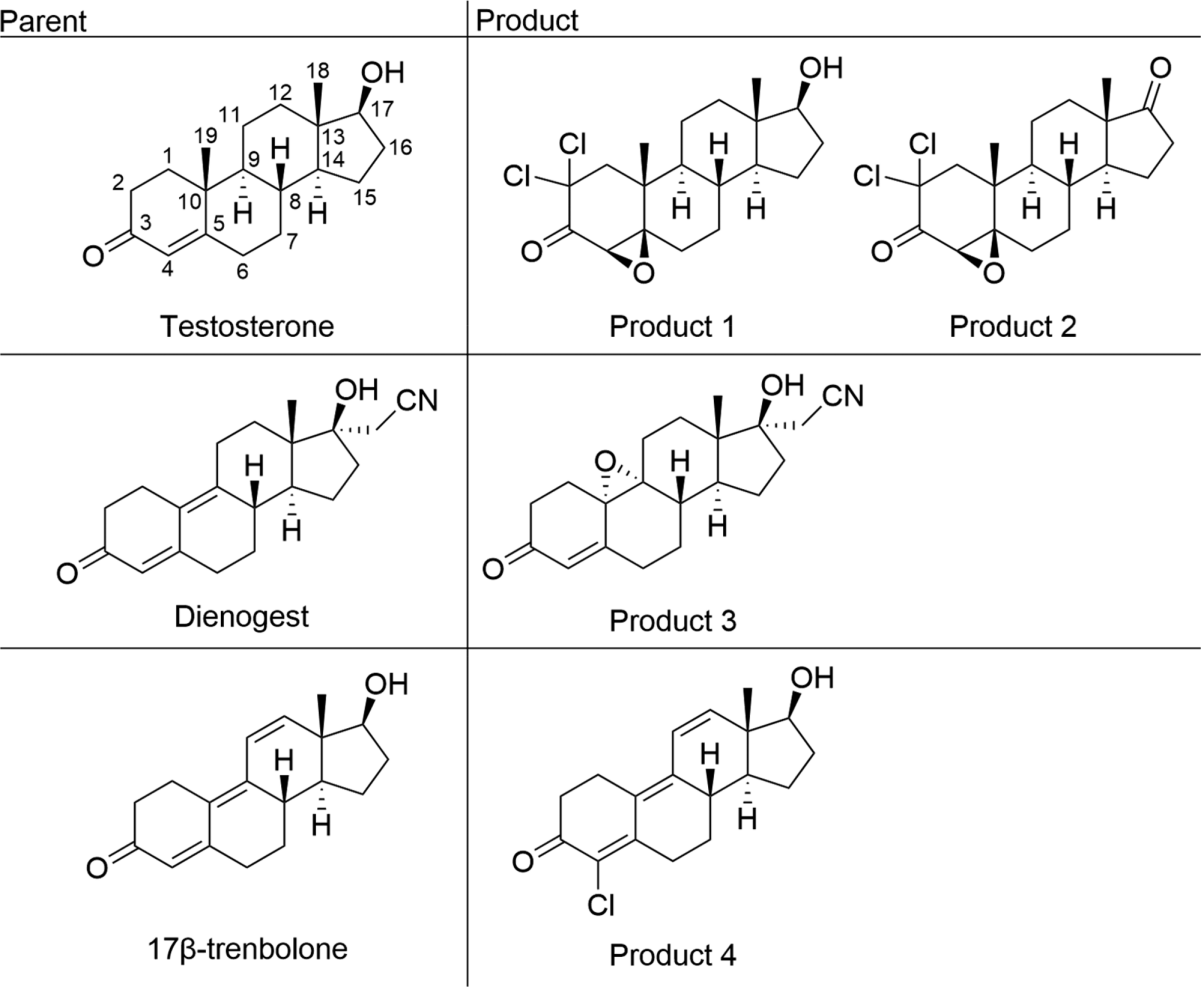
Representative structures of an enone, dienone, and trienone along with associated chlorination products that were fully characterized during this study.

As a proof of concept, we independently predicted the chlorination products of three of these steroids (shown in Figure 1), testosterone, dienogest, and 17β-trenbolone, in silico. In complementary laboratory experiments, we examined the rate and extent of steroid transformation through kinetic batch studies via reaction with chlorine, while also using semipreparative high performance liquid chromatography (HPLC), high-resolution electrospray ionization mass spectrometry (HRESIMS), high-resolution electron ionization mass spectrometry (HREIMS), ultraviolet–visible spectroscopy (UV–vis), and 1D and 2D nuclear magnetic resonance (NMR) techniques complemented by electronic circular dichroism (ECD), where necessary, to identify major transformation products, including stereochemistry, when appropriate. This approach for verifying the in silico results provides us the highest degree of certainty among existing approaches^29^ with regard to the structure elucidation of novel transformation products. It also allows factors such as stereochemistry, which can have a dramatic effect on bioactivity,^30^ to be explicitly addressed. Outcomes of this work should improve predictions of the environmental fate of potent synthetic steroids, especially those with structural features common to our analyte suite (e.g., α,β-unsaturated ketones), while also guiding future occurrence studies for novel or bioactive steroidal transformation products.

## Experimental and Theoretical Methods

### Reagents

A full list of reagents can be found in the SI.

### Chlorination Reactions

Steroid chlorination reactions followed protocols outlined in our earlier work.^4^ Briefly, chlorination experiments were generally conducted in batch systems with 25 μM (6.8–7.8 mg/L) initial steroid concentration. Reactors were dosed between 0.5 to 5 mg (7–70 μM) Cl_2_/L at pH 7 and allowed to react for 5 h. For testosterone, reactors contained 1 mM testosterone and were dosed with 0.5 to 5.9 g (7–83 mM) Cl_2_/L at pH 7 and allowed to react while stirring for 20 h. Further details regarding the reaction conditions and reactor sampling can be found in the SI.

### Analytical Methods

A combination of techniques including HPLC, NMR, HRESIMS, HREIMS, UV–vis, and ECD were used to identify products. Analytical details regarding product isolation and characterization, including instrumentation, can be found in the SI.

### Theoretical Methods

All stationary points were located using the MN15^31^ density functional in conjunction with the 6-31+G(d,p) basis set in the presence of SMD (solvent model density)^32^ water. Vibrational frequencies were determined to characterize each stationary point as a minimum or a transition state (one imaginary vibrational mode) and to provide thermodynamic corrections. Intrinsic reaction coordinate (IRC) calculations were performed to confirm the minima connected to each transition state. Energies discussed are Gibbs free energies at standard conditions. Calculations were performed using Gaussian 16.^33^

On the basis of a preliminary screening with skeletal steroid structures (discussed below), full reactions were modeled between testosterone, dienogest, and 17β-trenbolone with HOCl, focusing on initial chlorination at C4 in all cases. HOCl was chosen as the chlorinating agent because [HOCl] greatly exceeds [Cl_2_] at all pH values of relevance to wastewater treatment. Several explicit water molecules were used to stabilize the nascent hydroxide ion formed following electrophilic addition of chlorine to the steroid, giving more accurate energetics and allowing for various proton transfers necessary for subsequent mechanistic steps. The utility of this approach has been demonstrated for the chlorination of amines by HOCl.^22−24^ All atoms were present in all calculations (i.e., the reactant species are reaction complexes rather than separated reactants and so on; see the SI for complete structures).

## Results and Discussion

### Preliminary Calculations on Model Steroid Structures

**Figure 2:**
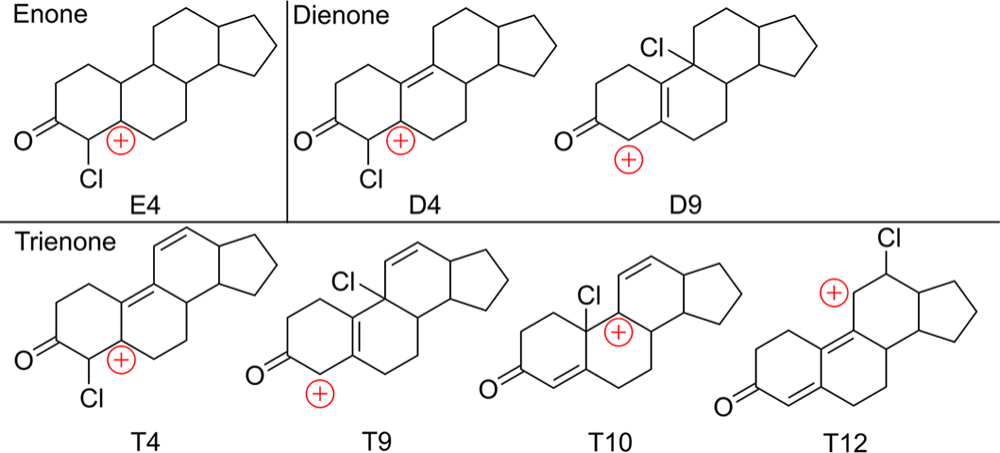
Model cationic intermediate structures considered for bare enone, dienone, and trienone skeletal structures.

Computational studies first examined all possible monochlorinated cationic intermediates of bare enone, dienone, and trienone skeletal structures (e.g., with no substituents, Figure 2). These structures are all β-carbocations rather than bridged chloronium ions as noted by Olah.^34^ Unsurprisingly, structure **E4** in Figure 2 is the only intermediate observed in chlorination of the model enone. Any attempt to place the chlorine at C5 results in a shift to give **E4**. Therefore, electrophilic chlorination at C4 of enones is the only pathway to consider.

In the dienone model system, it was found that **D4** is 10 kcal/mol more stable than **D9**. This is attributable to two causes. First, as evident in Figure 2, even though cation **D9** is resonance-stabilized, some positive charge ends up adjacent to the strongly electron-withdrawing carbonyl group. This is a much less electronically favorable situation than is seen in **D4** where the charge is insulated from the carbonyl. Second, the C9 position is more sterically crowded than C4. We can therefore predict that C4 chlorination will be the dominant pathway for dienones.

While the situation is less clear-cut for the model trienone, intermediate **T4** remains the most favorable. Intermediate **T9** is 23 kcal/mol less stable than **T4** due to the same considerations as for **D9**, but also due to loss of conjugation compared to **T4**. Intermediate **T10** is 9 kcal/mol less stable than **T4** due to loss of conjugation and steric issues. Intermediate **T12** lies only 3 kcal/mol higher than **T4**. Intermediate **T12** does not suffer from steric issues and the conjugation extends through the carbonyl, making it more conjugated than **T4**. However, conjugation of a carbocation with a carbonyl is a destabilizing feature. Therefore, **T4** remains the most likely site for chlorination even in the trienone model, albeit by a smaller margin.

### Steroid Chlorination Experiments

Consistent with prior reports, testosterone was resistant to transformation unless very high concentrations of chlorine (i.e., 500–5900 mg Cl_2_/L at pH 7) were used.^35,36^ We observed greater reactivity among all the trienone and dienone steroids examined, with near-complete transformation in the 5 mg Cl_2_/L systems over 5 h (Figure S2). Our experimental approach, which did not include collection of samples over time under conditions of excess (or ∼constant) chlorine, precludes us from determining second order rate constants for this reaction that could be used to model trienone and dienone fate in treatment systems. We can say definitively that compared to glucocorticoids, which we previously estimated^4^ would be at most only partially degraded (∼10%) based on their rate of reaction with chlorine, trienones and dienones are far more reactive. This is consistent with the work of Mash, who found trenbolone to be highly reactive and oxidized at rates comparable to those previously reported for estrogens including 17β-estradiol, estrone, and ethynyl estradiol.^37^ In fact, in our work with glucocorticoids,^4^ we conducted preliminary experiments that showed 17β-trenbolone and estrone to be degraded at comparable rates and over much shorter time scales than glucocorticoids. Collectively, therefore, our results suggest that trienone steroids such as 17β-trenbolone, 17α-trenbolone, methyltrenbolone, gestrinone, and altrenogest, along with the dienone steroids dienogest, dienedione, and methyldienolone, are susceptible to transformation and likely to be at least partially degraded, if not fully so, under typical chlorine concentrations and residence times used for wastewater treatment and during drinking water treatment and distribution.

**Figure 3:**
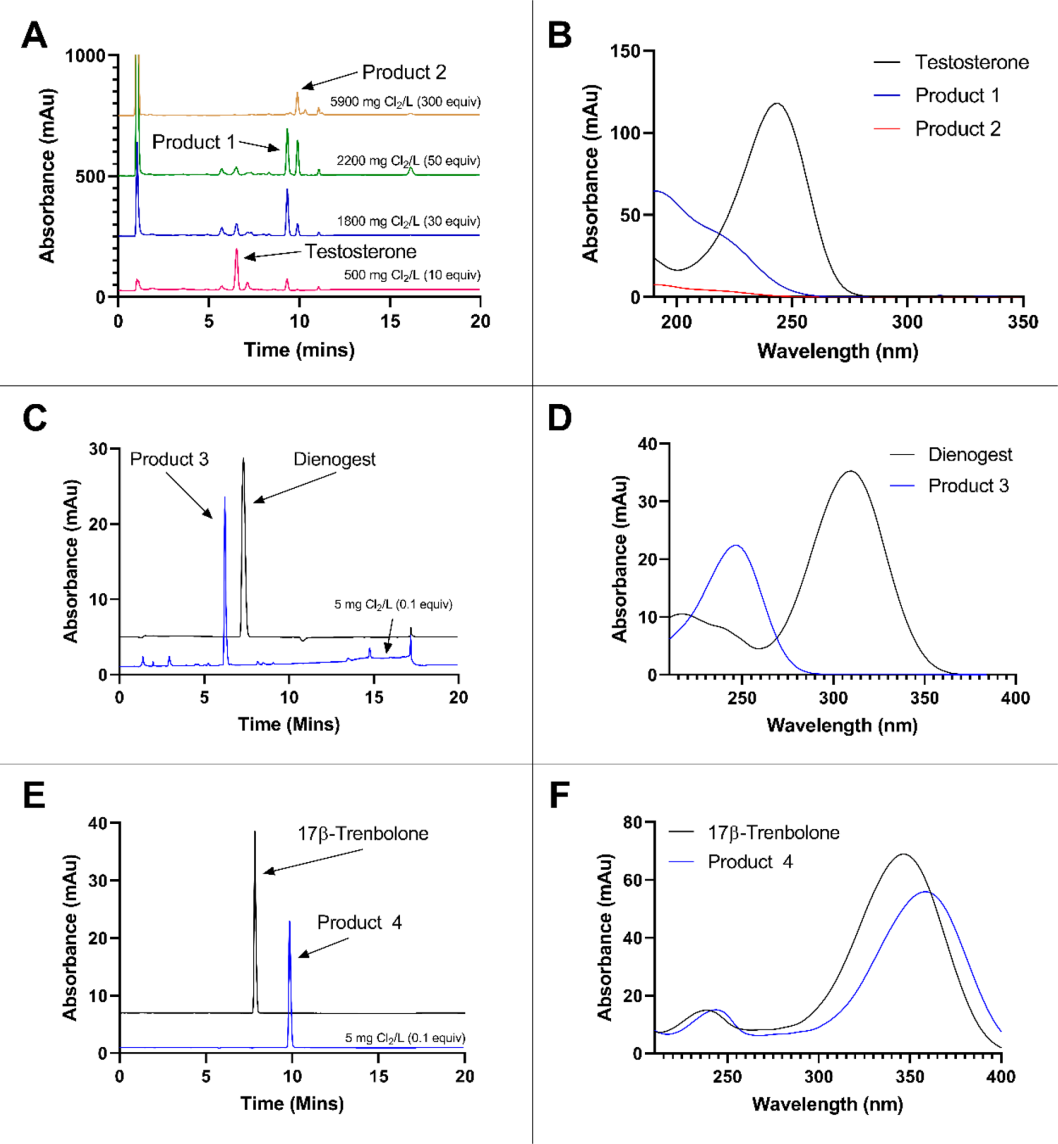
Reversed phase HPLC chromatograms of starting materials and the product mixtures obtained upon chlorination of testosterone, dienogest, and 17β-trenbolone are shown in panels A, C, and E, respectively. UV spectra of the products and parent compounds are displayed in panels B, D, and F. Individual chromatograms were monitored at different wavelengths suggested by their UV spectra. Testosterone was monitored at 250 nm, and its chlorination products **1** and **2**, which eluted at 9.3 and 9.9 min, were monitored at 210 nm. Dienogest was monitored at 300 nm, and its product **3** (eluting at 6.2 min) was monitored at 240 nm. 17β-Trenbolone and its reaction product **4** (eluting at 9.8 min) were both monitored at 350 nm.

Initial HPLC assessment of reaction mixtures of testosterone revealed the formation of two new, less polar, products at high chlorine concentrations (Figure 3A). Testosterone products **1** and **2** were produced in a ratio that was ultimately dependent on chlorine concentration. At lower chlorine concentrations, evidence of additional products was observed, but these were not generated in sufficient quantities to allow isolation for structural identification.

In all dienone (dienogest, dienedione, and methyldienolone) and most trienone systems (17α-trenbolone, 17β-trenbolone, methyltrenbolone, and gestrinone), we observed exclusive transformation to a single product across all ranges of chlorine doses tested (0.5–5 mg Cl_2_/L). For example, chlorination of dienogest produced a single more polar product **3** (Figure 3C). Reaction of 17β-trenbolone (Figure 3E) formed a new less polar product **4**. UV spectra of the products (Figure 3B,D,F) showed changes in the chromophores consistent with disruption or modification of the conjugated systems, most notably for the products of testosterone and dienogest. Thus, our preliminary assessment suggested clear differences in the end products generated from the reactions of dienones and trienones with free chlorine.

Altrenogest was the only trienone to demonstrate a more complex product formation pathway. Like other trienones, altrenogest was highly reactive across all concentrations investigated (from 0.1 to 5.0 mg/L as Cl_2_), but several peaks were observed using LC-DAD suggesting that a mixture of products was formed. Presumably, the electron-rich allyl substituent at C17 enables additional, alternative reaction pathways that influence the product distribution upon electrophilic chlorination.

### Product Structure Elucidation

Guided by outputs from our preliminary computational studies, structural identification focused on presumed changes in the A-ring of all steroids. In all cases below, the compounds that were isolated and characterized were the major products of the process, as judged both chromatographically and by the yields of material obtained.

#### Testosterone

We observed formation of two products (**1** and **2**) at high free chlorine concentrations. HREIMS produced a molecular ion M^+•^ for product **1** at *m*/z 372.1246, indicating a formula of C_19_H_26_O_3_Cl_2_, and for product **2** at *m*/z 370.1117, indicating a formula of C_19_H_24_O_3_Cl_2_.

NMR signal assignments for testosterone were based on those previously published in the literature.^38^ The structures of products **1** and **2** were independently determined mainly by analysis of heteronuclear single quantum coherence spectroscopy (HSQC) and heteronuclear multiple-bond correlation spectroscopy (HMBC) data. These two-dimensional NMR techniques provide much more detailed structural information than standard ^1^H or ^13^C NMR experiments, as they allow independent determination of single and multiple carbon–hydrogen bond relationships based on heteronuclear (*J*_CH_) couplings. Analysis of these data led to assignment of the structure of product **1** as 2,2-dichloro-4β,5β-epoxy-17β-hydroxyandrost-3-one and that of product **2** as 2,2-dichloro-4β,5β-epoxyandrostan-3,17-dione. Structure elucidation details, including epoxide stereochemical assignment, can be found in the SI (Figures S3–S13 and Tables S1–S3).

Attempts to characterize the minor products observed via reaction with testosterone at lower concentrations of free chlorine using LC-HRMS were unsuccessful, presumably due to their limited abundance and/or poor ability to ionize during analysis. Because these products were not observed at high chlorine concentration, they are presumed to be reactive and likely precursors to formation of products **1** and **2**.

#### Dienogest

HRESIMS analysis of the reaction product from dienogest (**3**) gave an (M + H)^+^ ion at *m*/*z* 328.1867, corresponding to the formula C_20_H_25_NO_3_, indicative of the addition of an oxygen atom. Analysis of the spectroscopic data collected for product **3** indicated epoxidation across the C-9/C-10 olefin of the parent dienogest resulting in the product 9,10-epoxy-dienogest.

Detailed characterization and the spectroscopic data gathered for the structure elucidation of the dienogest product (**3**) can be found in the SI (Figures S14–S23 and Table S4).

##### 17β-Trenbolone

For the trienone steroid 17β-trenbolone, HRESIMS analysis (Figure S25) of the chlorination product gave an (M + H)^+^ ion at *m*/*z* 305.1312, corresponding to the formula C_18_H_21_ClO_2_ and indicative of replacement of a hydrogen with a chlorine atom. Spectroscopic information collectively allowed identification of product **4** as 4-chloro-17β-trenbolone. NMR, HRESIMS, and a structure elucidation narrative can be found in the SI (Figures S24–S27 and Table S5).

#### Additional Dienones and Trienones

With the exception of altrenogest, the other dienones and trienones we examined were assumed to also yield exclusively 9α,10α-epoxidation and 4-chlorination products, respectively, based on HRESIMS, UV, and relative retention time data (Figures S34–S43). In fact, 9α,10α-epoxy-methyldienolone is a known product of reaction of methyldienolone with *m*-chloro-perbenzoic acid, and the λ_max_ (∼244 nm) previously reported for this product matched that observed for the product in our chlorination systems.^39,40^

For altrenogest, complete characterization of its more complex product distribution generated from the reaction with free chlorine is beyond the scope of the current work. However, we did isolate the two most abundant products (again judged on the basis of both chromatographic prominence and yields obtained) with HPLC and characterized them using NMR and HRESIMS. Analysis of the gathered spectroscopic data for products **5** and **6** allowed them to be assigned as 4-chloro-altrenogest and 11,12-epoxy-altrenogest, respectively. Notably, although chlorination at C4 is consistent with the results for other trienones, additional products including an 11,12-epoxide were not observed for any other species. Characterization details, including elucidation narrative, NMR, and HRESIMS data, can be found in the SI (Figures S28–S33 and Table S6).

### Modeling Complete Reactions

**Figure 4:**
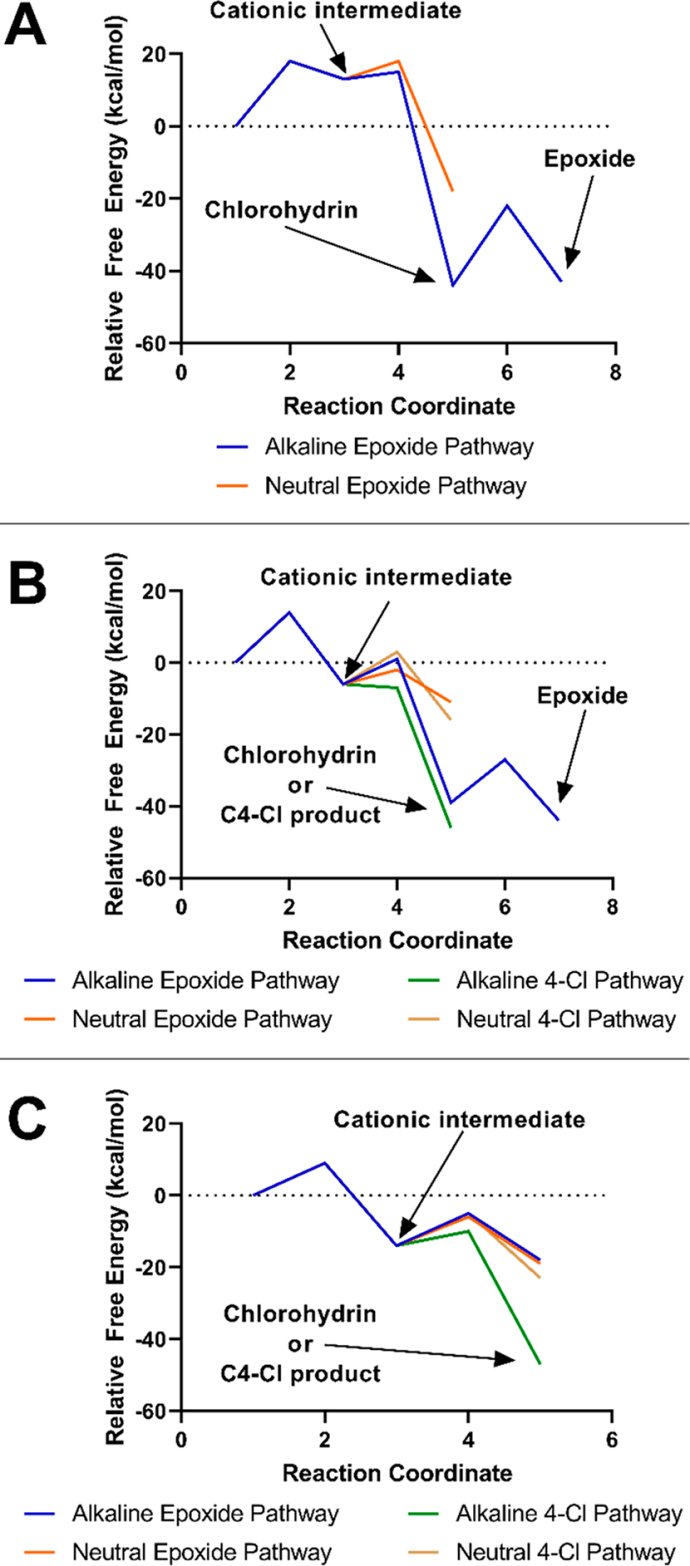
Reaction coordinates for testosterone (A), dienogest (B), and 17β-trenbolone (C), with key species (e.g., cationic intermediate, chlorohydrin/C4–Cl product, and epoxide) noted. Note the reversible nature of the carbocationic intermediate (point 3 on reaction coordinate) for testosterone.

Having established that electrophilic addition of chlorine to C4 is favored in enone, dienone, and trienone steroids (from preliminary calculations with model steroid structures; Figure 2 and Table S7), we proceeded to model complete reaction pathways for select steroids. Free energy diagrams associated with the chlorination of testosterone, dienogest, and 17β-trenbolone are shown in Figure 4. We considered the reaction under both neutral and alkaline pH conditions, which are most representative of the use of free chlorine as hypochlorous acid/hypochlorite during water treatment. In every case, we modeled the initial electrophilic chlorination by adding HOCl to the bottom face of each steroid at C4 (which we define as being *anti* to the C10 methyl group of testosterone or the C13 methyl group of both dienogest and 17β-trenbolone). This attack was chosen because it avoids steric conflict with the C10 methyl in testosterone, and we wished to explore a consistent attack for all three steroids. Regardless of steroid, this leads to a cationic intermediate structure with Cl in a pseudoequatorial position at C4 and generates a free hydroxide, which can then migrate via various proton transfers to other reactive positions.

### Initial Chlorination of Testosterone, Dienogest, and 17β-Trenbolone

The computational data support a slow and endergonic initial chlorination event via HOCl at C4 in testosterone. The same chlorination event is predicted to have a more modest barrier and be exergonic with dienogest, and be quite rapid and significantly exergonic with 17β-trenbolone (Table S8). These predictions are consistent with our current and prior experimental observations that testosterone is difficult to chlorinate,^35^ especially relative to the high reactivity of dienones and trienones. This trend in the ease of initial electrophilic chlorine addition is easily understood from physical organic principles; the cationic intermediate formed from addition to testosterone is a secondary carbocation with no other stabilizing features. As such, the electrophilic addition is predicted to be unfavorable and reversible, resulting in a slow reaction. In contrast, the intermediate for dienogest is an allylic cation, which benefits from resonance stabilization, whereas for 17β-trenbolone there is additional resonance leading to even greater stability of the intermediate. For both conjugated cases, the electrophilic addition is favorable, and rapid reaction is expected.

### Subsequent Reaction Steps for Testosterone

We probed the additional chemistry of the carbocationic intermediate formed from electrophilic chlorination at C4 in testosterone by exploring several reasonable possibilities. Despite multiple attempts, no satisfactory stationary points could be located corresponding to a transition state for deprotonation. However, a pathway was found for C4–C5 chlorohydrin formation. The transition state for chlorohydrin formation lies 15 kcal/mol above reactants, placing it just a few kcal/mol lower in energy than the initial chlorination transition state. While the remainder of the pathway is favorable (Figure 4 and Table S9), it is evident that the first step is slow and at least partially reversible. We note that the C4–C5 chlorohydrin appears to be isoenergetic and in rapid equilibrium with the corresponding epoxide under these conditions. This equilibrium will be greatly affected by actual reaction conditions, and we treat computational evidence for the chlorohydrin as sufficient evidence for formation of the epoxide.

Collectively, our predicted reaction pathway for testosterone is in good agreement with experimental observations. No evidence for C4 deprotonation is seen in either case, while a C4–C5 epoxide product is isolated. Presumably, some of the products observed to form at low chlorine equivalents in experimental systems correspond to the C4–C5 epoxide product, which we suspect subsequently chlorinates at C2 to yield the dichlorinated derivatives identified via NMR. Indeed, we suspect that C2 chlorination is possible for all of the steroidal enones investigated herein at high free chlorine concentrations like those used to generate sufficient product mass for isolation in testosterone systems.

### Subsequent Reaction Steps for Dienogest

We located two forward pathways from the dienogest carbocationic intermediate, one for deprotonation to form the C4–Cl product and one for C9–C10 epoxide formation via the C4–C9 chlorohydrin (Figure 4 and Table S9). Under the conditions modeled, the base/nucleophile is the nascent hydroxide formed from HOCl. The deprotonation step is found to proceed with no apparent additional barrier. The final C4–Cl product lies well below reactants.

Due to the stereochemical constraints, the *anti* C4–C5 and *syn* C4–C9 chlorohydrins are the two most reasonable products that can form from the initial cationic intermediate. The C4–C9 is considerably more stable than the C4–C5, so that is the only pathway we pursued in detail. There is a noticeable barrier of 7 kcal/mol to formation of the *syn* C4–C9 chlorohydrin, but the overall reaction is highly exergonic. In contrast to the deprotonation step, chlorohydrin formation requires very little reorganization of the steroid ring structure. The importance of this will be discussed below. Closure to the C9–C10 epoxide requires a modest barrier, but is also very exergonic. The overall pathway from reactants to epoxide shows that the initial chlorination is the rate-determining step with each ensuing mechanistic step being effectively spontaneous.

Since both deprotonation and chlorohydrin formation are predicted to be highly exergonic and irreversible, any bifurcation between the two is determined by the highest barrier along each pathway subsequent to the common intermediate. Such an analysis shows that the deprotonation pathway is the dominant pathway (i.e., the *difference* in activation barriers between the two pathways is 7 kcal/mol in favor of deprotonation). This should lead to an overwhelming preponderance of C4–Cl dienogest product and negligible epoxide product, in direct contradiction to our experimental result.

To address this contradiction, we note that the use of hydroxide as the base/nucleophile in our model is necessitated to maintain mass balance in the computational system. This implies an alkaline environment. However, hydroxide will be found at very low concentrations in the experimental system at pH 7, and water is the more probable base/nucleophile. We would expect water to be much less efficient for deprotonation, but perhaps similarly efficient for nucleophilic attack on the chlorohydrin. To probe this possibility, we performed a limited set of calculations replacing hydroxide with chloride, thus maintaining charge balance, but forcing water to be the base/nucleophile. We computed only the two transition states for deprotonation and chlorohydrin formation as well as the 4-Cl and C4–C9 chlorohydrin products. Indeed, we saw that deprotonation is now *less* favored by 5 kcal/mol, and the chlorohydrin to epoxide pathway is now dominant, as seen in the experiment. Irreversibility of both pathways is retained (Table S10). This suggests a strong pH dependence on product formation in dienogest, which was subsequently confirmed experimentally; we observed a change in product formation, including evidence of new products, for chlorination reactions with dienogest under alkaline conditions (pH 9) (Figure S44).

### Subsequent Reaction Steps for 17β-Trenbolone

By analogy to the studies of dienogest, we explored both deprotonation and chlorohydrin formation pathways via hydroxide reaction with the 17β-trenbolone cationic intermediate (Figure 4 and Table S9). Preliminary calculations revealed that the *syn* C4–C12 chlorohydrin is the most stable possible chlorohydrin, so that was the only one examined in detail.

The overall features of the deprotonation pathway are comparable to those seen with dienogest. While there is a modest barrier to deprotonation, the electrophilic addition is still rate-determining by a large margin, and the overall reaction is highly exergonic and irreversible. However, we note a distinct contrast between the chlorohydrin pathways of these two steroids. The forward and reverse barriers for C4–C12 chlorohydrin formation in 17β-trenbolone are similar and low enough to be rapid at room temperature, and chlorohydrin formation is predicted to be reversible. This suggests that thermodynamic equilibrium plays a role in the chlorination chemistry of 17β-trenbolone that is not seen in dienogest.

We also examined the effect of pH on the process with 17β-trenbolone, as we did with dienogest. With hydroxide as the base/nucleophile, deprotonation is favored by 9 kcal/mol. When water is the base/nucleophile, chlorohydrin formation is very slightly favored. However, deprotonation is irreversible under both pH conditions and chlorohydrin formation is reversible under both pH conditions (Table S10). Therefore, even at pH 7 where there is close kinetic competition between the two pathways, the thermodynamic product (i.e., the 4-Cl product) will come to dominate the equilibrium mixture, as was observed in our experimental systems.

### Environmental Implications

Although continued development and demonstration is necessary, this work illustrates the promise of using computational tools to aid prediction and identification of products generated via the environmental transformation of emerging contaminant classes. In nearly all cases, the computational predictions of relative reactivity and major products of steroid chlorination were confirmed with experimental evidence, and instances where theory and experiment were at odds could be easily rationalized. Several generalizable best practices derived from this work may help advance the use of computational tools for predicting environmental transformation products by establishing a framework for computational prescreening that could ultimately minimize the amount of confirmatory lab work required. These are as follows:

1. *Start by screening the initial attack using simple model systems.* Because there are likely to be multiple locations on a compound that are susceptible to reaction, using simple structures with minimal functionalities (like those in Figure 2) can help to identify the most reactive sites. For steroidal trienones, dienones, and enones, this helped identify the C4 locations as a primary reactive point during chlorination.
2. *Next, screen for potential pH effects*. Herein, deviations between computational predictions and experimental results could be rationalized when pH effects were considered. For example, observation of epoxide rather than C4 chlorination products for steroidal dienones could be rationalized with water (neutral pH), rather than hydroxide (alkaline pH), as the probable nucleophile. For chlorination, another pH effect to probe is how the pH-dependent equilibrium between HOCl and OCl^–^ affects the mechanism. Accordingly, limiting pH-dependent pathways can be predicted, and cases can be identified where the product distribution is likely to be influenced by pH.
3. *Analyze the data from these two screening approaches to classify reactive and unreactive compounds.* We found that theory predicts and experiments confirm that testosterone is effectively unreactive toward chlorine under conditions representative of chemical disinfection, while trienone and dienone steroids exhibit much greater reactivity. Thus, computational approaches will hold value in helping identify which emerging pollutants to prioritize when considering environmental transformation processes. Initial screening to probe potential reactivity can be useful in limiting the number of chemicals that merit experimental investigation, even without proceeding on to prediction of probable transformation products.
4. *Probe functional groups that may perturb predictions and/or lead to additional reaction*s. In our work, altrenogest, with its unique allyl group at C17, provides a useful counter example of where other products could be anticipated (i.e., in cases where electron-rich substituents are also present). Interestingly, the generalized trend in product formation still held to some degree for altrenogest; although 4-chlorination and similar epoxidation products were identified, these were accompanied by other minor, as-yet unidentified products that we presume involve reactions with the allyl substituent. Such products, as well as products of secondary transformations (i.e., those formed from the subsequent reaction of transformation products), would not be captured in our approach, which was limited to modeling pathways localized around a generally reactive carbon center (C4) across most steroidal enones, dienones, and trienones.

In applying this framework, it is recognized that computational chemistry cannot capture every nuance of complex environmental systems. However, we find significant value in using computational chemistry to screen model systems to produce a strong, baseline prediction for relative reactivity and probable transformation products in such systems.

Beyond providing a framework for using computational tools to predict transformation products, this work also illustrates the pressing need to carefully consider transformation products for potent synthetic chemicals, including steroids used in medicine and agriculture. Observation of the trienone chlorination products is noteworthy, as introduction of a chlorine atom at position C-4 is known to increase anabolic potency of androgenic steroids.^21^ This is especially pertinent for the highly potent trienones 17β-trenbolone and methyl trenbolone, which have a 20-fold and 250-to-350-fold increased anabolic effect over testosterone, respectively.^41−43^ Several studies have shown that fish exposed to low doses (ng/L) of androgens can lead to male secondary sex characteristics in females, and also decreased fecundity, plasma vitellogenin, and sex steroid concentrations.^44−46^ Thus, an increase in anabolic activity for these already potent androgens is expected to also increase ecological risk associated with these contaminants.

Observation of the dienone epoxidation products is also environmentally relevant. As mentioned above, 9,10-epoxy-methyldienolone is a known product of methyldienolone reaction with *m*-chloro-perbenzoic acid, although, to the best of our knowledge, its bioactivity has not previously been reported.^40^ However, in the same study, the 9,10-epoxy-methyldienolone product was treated with aqueous potassium hydroxide, resulting in epoxide ring-opening, dehydration, and aromatization to yield an aromatic product (Aromatic 1, Figure S44).^40^ To test this, the previously isolated 9,10-epoxy-dienogest product was dissolved in water followed by subsequent addition of either aqueous acid or base, which afforded the analogous aromatic dienogest derivative (Aromatic 2, Figure S44). Observation of this aromatic compound, which has been previously isolated,^47^ is notable because it has been reported to exhibit estrogenic activity at ∼30% that of the endogenous ligand 17β-estradiol.^48,49^ This level of estrogenic activity is similar to that of estrone,^50^ one of the most well-recognized steroidal transformation products in aquatic environments. Likewise, the analogous aromatic product (Aromatic 3, Figure S44) derived from 9,10-epoxy-dienedione has been reported to possess estrogenic activity at ∼50% that of estrone.^51^

More broadly, this work provides yet another example where a common environmental transformation process (chemical disinfection with free chlorine) does not entirely eliminate ecosystem risks associated with emerging pollutant classes. Conserved, enhanced, or broader spectrum receptor bioactivity through environmental transformation processes challenges our current regulatory and risk assessment paradigms, while also complicating prioritization of analytical targets for environmental monitoring. Collectively, outcomes of this work may help to guide future occurrence studies for persistent and bioactive steroid transformation products, while also improving predictions of the environmental fate of potent synthetic steroids.

## Supporting Information Available

Analytical methods, product identification, HRMS, NMR, UV–vis, and computational data (PDF)
