# Supplementary material for: Computational Approaches for the Prediction of Environmental Transformation Products: Chlorination of Steroidal Enones
Source: Environ Sci Technol. 2021 Oct 12;55(21):14658–66. doi: 10.1021/acs.est.1c04659 (PMC8567416; doi:10.1021/acs.est.1c04659)
Supplement: Supplementary file 1 — es1c04659_si_001.pdf [file es1c04659_si_001.pdf]

Supporting Information

For

Computational approaches for the prediction of environmental transformation products: Chlorination of steroidal enones

Christopher J. Knutson,[1] Nicholas C. Pflug,[2] Wyanna Yeung,[3] Matthew Grobstein,[3] Eric V. Patterson,[3] David M. Cwiertny,[4]* and James B. Gloer[1]*

[1]Department of Chemistry, University of Iowa, Iowa City, IA 52242, USA
[2]Institute of Biogeochemistry and Pollutant Dynamics, ETH Zurich, 8092 Zurich, Switzerland
[3]Department of Chemistry, Stony Brook University, Stony Brook, NY 11794, USA
[4]Department of Civil and Environmental Engineering, University of Iowa, Iowa City, IA 52242, USA

*Co-corresponding Author Contact Information:

James B. Gloer

Address: Department of Chemistry

University of Iowa, Iowa City, IA 52242

E-mail: james-gloer@uiowa.edu

Phone: 319-335-1361

David M. Cwiertny

Address: 4105 Seamans Center

University of Iowa, Iowa City, IA 52242

E-mail: david-cwiertny@uiowa.edu

Phone: 319-335-1401

Fax: 319-335-5660

Contents: Analytical methods, details of product identification, HRMS, NMR, UV-Vis, and computational data

Summary : 214 pages, 44 Figures, 10 Tables

Contents

Structures 6

Figure S1. Structures of all parent compounds investigated along with their associated products. 6

Reagents 7

Analytical Methods and Product Characterization 7

NMR Details 7

HRMS Details 8

Chlorine Concentration Standardization 8

Overview of Chlorination Experiments 9

Testosterone Reaction 9

*Testosterone Product **1** and **2** Structure Elucidation* 10

Dienogest Reaction 11

*Dienogest Product **3** Structure Elucidation* 12

17β-Trenbolone Reactions 13

*17β-Trenbolone Product **4** Structure Elucidation* 13

Altrenogest Reaction 14

*Altrenogest Product **5** Structure Elucidation* 15

*Altrenogest Product **6** Structure Elucidation* 15

Concentration of parent compounds as a function of chlorine concentration after 5 h reaction. 16

Figure S2. Concentration of 17β-trenbolone (17β-TBOH), 17α-trenbolone (17α-TBOH), gestrinone (GES), methyl-trenbolone (Me-TBOH), dienedione (DND), dienogest (DNG) and methyldienolone (MDL) as a function of initial aqueous chlorine concentration. Reaction conditions: Initial steroid concentration of 25 µM (~ 7-8 mg/L), initial chlorine concentration of 0.5-5 mg Cl2/L, ~ 5h reaction time, and pH 7. 16

Testosterone Data 17

Figure S3. HRESIMS of testosterone with an $M+H^+$ ion at *m/z* 287.2009. 17

Figure S4. ^1H NMR spectrum of testosterone standard in $CDCl_3$ (600 MHz). 18

Product 1 (2,2-Dichloro-4β,5β-epoxy-17β-hydroxyandrost-3-one) Data. 19

Figure S5. ^{1}H NMR spectrum of product **1** (2,2-dichloro-4β,5β-epoxy-17β-hydroxyandrost-3-one) in $CDCl_3$ (600 MHz). 19

Figure S6. HSQC of product **1** (2,2-dichloro-4β,5β-epoxy-17β-hydroxyandrost-3-one) in $CDCl_3$. 20

Figure S7. HMBC of product **1** (2,2-dichloro-4β,5β-epoxy-17β-hydroxyandrost-3-one) in $CDCl_3$. 21

Table S1. NMR data for product **1** (2,2-dichloro-4β,5β-epoxy-17β-hydroxyandrost-3-one) in $CDCl_3$ (600 MHz). 22

Figure S8. HREIMS of product **1** (2,2-dichloro-4β,5β-epoxy-17β-hydroxyandrost-3-one) produced an $M^{+\bullet}$ ion at *m*/z 372.1246, indicating a formula of $C_{19}H_{26}O_3Cl_2$. An expansion of the peak at *m/z* 372.1246 and the accompanying characteristic chlorine-37 isotope peak has been inset for reference. 23

Product 2 (2,2-dichloro-4β,5β-epoxyandrostan-3,17-dione) Data. 24

Figure S9. ^{1}H NMR spectrum of product **2** (2,2-dichloro-4β,5β-epoxyandrostan-3,17-dione) in $CDCl_3$ (600 MHz). 24

Figure S10. HSQC of product **2** (2,2-dichloro-4β,5β-epoxyandrostan-3,17-dione) in $CDCl_3$. 25

Figure S11. HMBC of product **2** (2,2-dichloro-4β,5β-epoxyandrostan-3,17-dione) in $CDCl_3$. 26

Table S2. NMR data for product **2** (2,2-dichloro-4β,5β-epoxyandrostan-3,17-dione) in $CDCl_3$ (600 MHz). 27

Figure S12. HREIMS of product **2** (2,2-dichloro-4β,5β-epoxyandrostan-3,17-dione) produced an $M^{+\bullet}$ ion at *m*/z 370.1117, indicating a formula of $C_{19}H_{24}O_3Cl_2$. 28

Product 2 (2,2-dichloro-4β,5β-epoxyandrostan-3,17-dione) ECD Study 29

Figure S13. Comparison of the ECD spectrum of 2,2-dichloro-4β,5β-epoxyandrostan-3,17-dione and the calculated spectra of the α and β epoxide isomers 29

Table S3. Cartesian coordinates of the optimized models of α and β epoxide isomers of product **2** at RI-BP/TZVP level. 31

Dienogest data 32

Figure S14. Proposed mechanism of dienogest to product 3 (9,10-epoxydienogest) transformation. ...32

Figure S15. HRESIMS of dienogest standard with an $(M+H)^+$ ion at *m/z* 312.1928. 33

Figure S16. ^{1}H NMR of dienogest standard in $CDCl_3$ (600 MHz). 34

Product 3 (9,10-epoxy-dienogest) Data 35

Figure S17. HRESIMS of product **3** (9,10-epoxy-dienogest) with an $(M+H)^+$ ion at *m/z* 328.1867, corresponding to the formula $C_{20}H_{25}NO_3$ along with minor impurities. 35

Figure S18. ^{1}H NMR spectrum of product **3** (9,10-epoxy-dienogest) with minor impurities in $CDCl_3$ (600 MHz). 36

Figure S19. HSQC spectrum of product **3** (9,10-epoxy-dienogest) in $CDCl_3$ (600 MHz). 37

Figure S20. HMBC spectrum of product **3** (9,10-epoxy-dienogest) in $CDCl_3$ (600 MHz). 38

Table S4. NMR data for product **3** (9,10-epoxy-dienogest) in $CDCl_3$ (600 MHz). 39

Figure S21. UV-Vis spectrum of (A) 17β-trenbolone standard, (B) dienogest standard, (C) 17β-trenbolone chlorination product (**4**), and (D) dienogest chlorination product (**3**). 40

Figure S22. Key HMBC correlations and HRESIMS data for product **3** (9,10-epoxy-dienogest) and product **4** (4-chloro-17β-trenbolone). 41

Dienogest Product 3 (9,10-epoxy-dienogest) as a Function of pH 42

Figure S23. Dienogest product **3** (9,10-epoxy-dienogest) distribution changes as a function of pH. 42

17β-trenbolone data 43

Figure S24. HRESIMS data for 17β-trenbolone standard gave an $(M+H)^+$ ion at *m/z* 271.1689....43

Product 4 (4-Chloro-17β-Trenbolone) Data 44

Figure S25. HRESIMS data for product **4** (4-chloro-17β-trenbolone) gave an $(M+H)^+$ ion at *m/z* 305.1312....44

Figure S26. ^{1}H NMR spectrum of product **4** (4-chloro-17β-trenbolone) in CD_3OD (600 MHz)....45

Figure S27. HMBC spectrum of product **4** (4-chloro-17β-trenbolone) in CD_3OD (600 MHz). 46

Table S5. NMR data for product **4** (4-chloro-17β-trenbolone) in CD_3OD (600 MHz). 47

Altrenogest Data 48

Figure S28. HRESIMS data for altrenogest gave an $(M+H)^+$ ion at *m/z* 311.2001. 48

Figure S29. ^{1}H NMR spectrum of altrenogest in CD_3CN (500 MHz). 49

Table S6. ^{1}H NMR data for altrenogest in CD_3CN (500 MHz). 50

Product 5 (4-chloro-altrenogest) Data 51

Figure S30. HRESIMS data for product **5** (4-chloro-altrenogest) yielded an $(M+H)^+$ ion at *m/z* 345.1617....51

Figure S31. ^{1}H NMR spectrum of product **5** (4-chloro-altrenogest) in CD_3CN (600 MHz). 52

Product 6 (11,12-epoxy-altrenogest) Data 53

Figure S32. HRESIMS data for product **6** (11,12-epoxy-altrenogest) yielded an $(M+H)^+$ ion at *m/z* 327.1954....53

Figure S33. ^{1}H NMR spectrum of product **6** (11,12-epoxy-altrenogest) in CD_3CN (600 MHz). 54

Dienedione Data 55

Figure S34. HRESIMS data of dienedione standard yielded an $(M+H)^+$ ion at *m/z* 271.1664. 55

Product 7 (9,10-epoxy-dienedione) data 56

Figure S35. HRESIMS data of product **7** (9,10-epoxy-dienedione) yielded an $(M+H)^+$ ion at *m/z* 287.1635....56

Methyldienolone Data 57

Figure S36. HRESIMS data of methyldioenolone standard yielded an $(M+H)^+$ ion at *m/z* 287.1978...57

Product 8 (9,10-epoxy-methyldienolone) Data 58

Figure S37. HRESIMS data of product **8** (9,10-epoxy-methyldienolone) yielded an $(M+H)^+$ ion at *m/z* 303.1932....58

Methyl Trenbolone Data 59

Figure S38. HRESIMS of methyl trenbolone standard yielded an $(M+H)^+$ ion at *m/z* 285.1866. 59

Product 9 (4-chloro-methyl-trenbolone) data 60

Figure S39. HRESIMS of product **9** (4-chloro-methyl-trenbolone) yielded an $(M+H)^+$ ion at *m/z* 319.1474..60

17α-trenbolone data..61

Figure S40. HRESIMS of 17α -trenbolone standard yielded an $(M+H)^+$ ion at *m/z* 271.1674.............61

Product 10 (4-chloro-17α-trenbolone) data..62

Figure S41. HRESIMS of product **10** (4-chloro-17α -trenbolone) yielded an $(M+H)^+$ ion at *m/z* 305.1290..62

Gestrinone Data 63

Figure S42. HRESIMS of gestrinone standard yielded an $(M+H)^+$ ion at *m/z* 309.1846.....................63

Product 11 (4-chloro-gestrinone) data 64

Figure S43. HRESIMS of product **11** (4-chloro-gestrinone) yielded an $(M+H)^+$ ion at *m/z* 343.1443. 64

Computational Data 65

Table S7. Relative free energies (kcal mol-1) for bare cationic intermediates (MN15/6-31+G(d,p) + SMD water)..65

Table S8. Relative free energies (kcal mol^{-1}) for initial chlorination at C4 via HOCl (MN15/6-31+G(d,p) + SMD water)..65

Table S9. Relative free energies (kcal/mol) for deprotonation and chlorohydrin formation, with either hydroxide or water. Energies are relative to common cationic intermediate. (MN15/6-31+G(d,p) + SMD water). [1]C4-C9 chlorohydrin for dienogest, C4-C12 chlorohydrin for beta-trenbolone...............65

Table S10. Forward and reverse free energies of activation (kcal/mol) for 4-Cl and chlorohydrin formation from the common intermediate, using either hydroxide or water. (MN15/6-31+G(d,p) + SMD water)..66

Figure S44. Reaction of dienones with aqueous chlorine results in exclusive 9,10-epoxidation. Subsequent treatment of epoxy products with aqueous acid or base results in epoxide ring opening and aromatization to form known estrogenic products..67

Coordinates, Electronic Energies and Free Energies for Bare Cationic Intermediates; MN15/6-31+G(d,p) + SMD water...68

Coordinates, Electronic Energies and Free Energies for Reactions with HOCl; MN15/6-31+G(d,p) + SMD water 79

Coordinates, Electronic Energies and Free Energies for Reactions with Cl_2; MN15/6-31+G(d,p) + SMD water 165

Structures

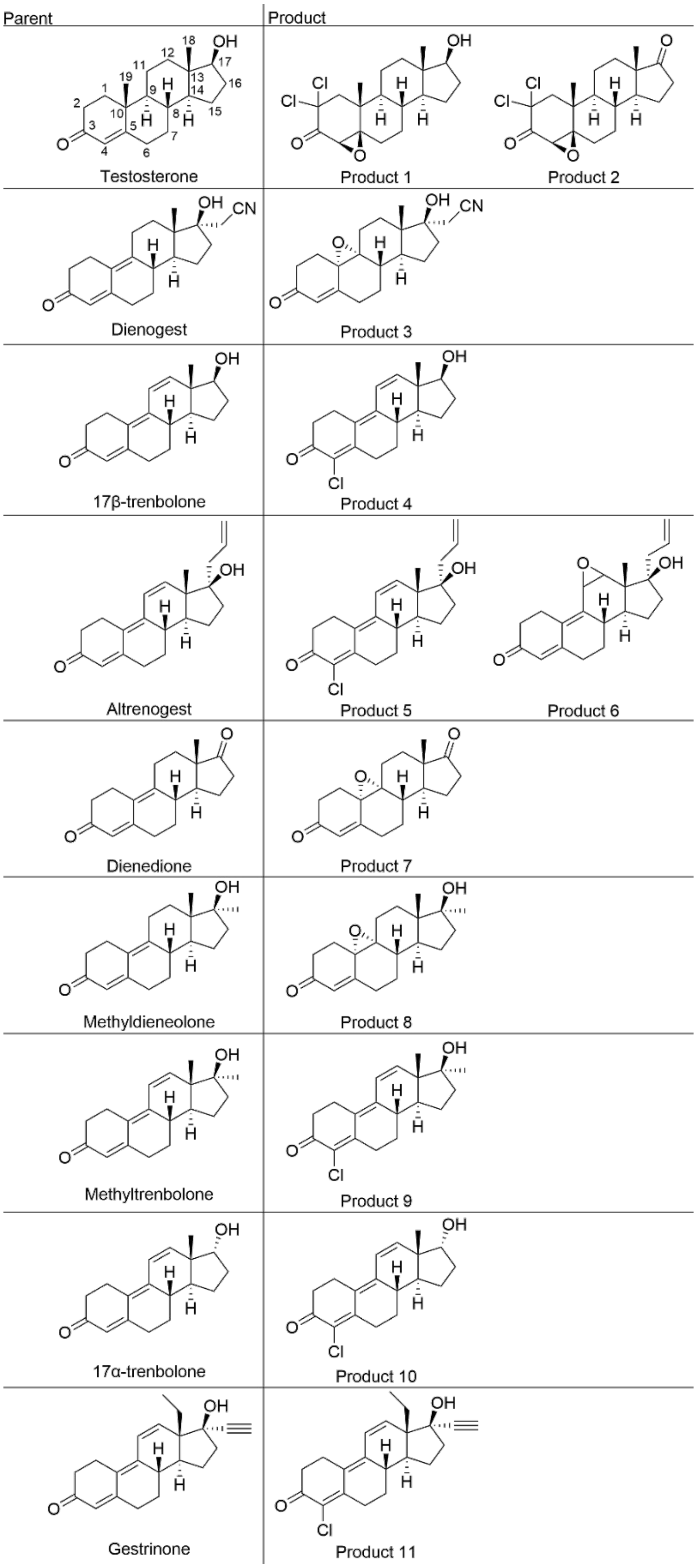

Figure S1. Structures of all parent compounds investigated along with their associated products.

Reagents

Chlorination experiments used sodium hypochlorite (NaOCl; Fisher Scientific; 5.65–6%), anhydrous potassium phosphate monobasic (KH_2PO_4; RPI; ACS grade), deionized water (Millipore, Q-Grade 2) and the following steroids: 17β-trenbolone (Sigma; ≥ 98%), testosterone (Sigma; ≥ 98%), dienogest (Sigma; ≥ 98%), dienedione (Boc Sciences; ≥ 98% HPLC), methyldienolone (Steraloids; ≥ 98% HPLC), methyltrenbolone (Sigma; ≥ 98% HPLC), 17α-trenbolone (Australian Government National Measurement Institute; ≥ 98%), gestrinone (Sigma; ≥ 98% HPLC), and altrenogest (Sigma; ≥ 98% HPLC). Reagents used for free chlorine concentration analysis included anhydrous sodium phosphate dibasic (Na_2HPO_4; RPI; ACS grade), disodium ethylenediamine tetra acetic acid dihydrate (EDTA; Sigma; ACS grade), ferrous ammonium sulfate hexahydrate (FAS; J.T. Baker; ACS grade), and N,N-diethyl-p-phenylenediamine (DPD; Aldrich; 97%). Liquid–liquid extractions were performed with chloroform (Fisher Scientific; ACS grade). HPLC analysis used deionized water (Millipore, Q-Grade 2) and acetonitrile (Fisher Scientific; ACS HPLC grade) as the mobile phase.

Analytical Methods and Product Characterization

NMR Details

NMR analysis of all compounds was conducted on a Bruker AVANCE-600 spectrometer. Chemical shift values for product **1** (2,2-dichloro-4β,5β-epoxy-17β-hydroxyandrost-3-one), product **2** (2,2-dichloro-4β,5β-epoxyandrostan-3,17-dione), and product **3** (9,10-epoxy-dienogest) were referenced to residual solvent signals for deuterated chloroform ($CDCl_3$, δ_H/δ_C 7.24/77.0). Chemical shift values for product **4** (4-chloro-17β-trenbolone) were referenced to residual solvent signals for deuterated methanol (CD_3OD, δ_H/δ_C 3.35, 4.78/49.3). ^1H NMR Chemical shift values

for product **5** (4-chloro-altrenogest) and product **6** (11,12-epoxy-altrenogest) were referenced to the residual solvent signal for deuterated acetonitrile (CD_3CN, δ_H 1.96).

HRMS Details

HR LCMS was conducted using either a Thermo Scientific Q Exactive or a Waters Q-TOF Premier. The Thermo Scientific Q Exactive was run using an Agilent Poroshell 120 EC-C18 (4.6 x 100 mm, 2.7 μm) column with 60% acetonitrile/water for 20 minutes at 0.5 mL/min over a mass range of 70-1000 in positive electrospray ionization (ESI) and a wavelength of 210 nm. The Waters Q-TOF Premier instrument was run using either an Agilent Zorbax Eclipse XDB-C18 (4.6 x 150 mm, 3.5 μm) column or an Agilent PREP-C_{18} Scalar (4.6 x 150 mm, 5 μm) column with a gradient of 25%-80% acetonitrile/water over 11 minutes at 0.5 mL/min with the addition of a 0.1% formic acid solution and collected using a reference standard of Leu-enkephalin and positive ESI over a mass range of 120-1000 Da under the following instrument parameters: 20 μL injection volume; 2.8 kV capillary, 35.0 V sampling cone, 4.0 V extraction cone, and 2.0 V ion guide voltages; 110 °C source temperature, and 400 °C desolvation temperature. HREIMS data were obtained using a Waters GCT Premier instrument via the solids probe inlet and collected using a reference standard of PFK and electron ionization over a mass range of 50-650 Da.

Chlorine Concentration Standardization

Aqueous stock solutions of hypochlorous acid were prepared by diluting concentrated sodium hypochlorite to ~5000 mg as Cl_2/L in amber glass bottles sealed with Teflon-lined screw caps and were stored at 4 °C. Free chlorine concentrations of hypochlorous acid stock solutions were measured via titration prior to use: 5 mL of each buffer reagent and the N,N-diethyl-p-phenylenediamine indicator solution were added to 100 mL of diluted sample, with the solution

turning pink in color in the presence of free chlorine. The free chlorine was then quantified by titration with ferrous ammonium sulfate hexahydrate solution until the pink color was no longer visible.

Overview of Chlorination Experiments

As noted briefly in the text, chlorination experiments were generally conducted in batch systems with 25 μM (6.8-7.8 mg/L) initial steroid concentration. Reactors were dosed between 0.5 to 5 mg (7-70 μM) Cl_2/L at pH 7 and allowed to react for 5 hours. For testosterone (see additional details below), reactors contained 1 mM testosterone, and were dosed with 0.5 to 5.9 g (7-83 mM) Cl_2/L at pH 7 and allowed to react while stirring for 20 hours. All reaction mixtures were extracted with chloroform, and products isolated by reverse phase HPLC.

Testosterone Reaction

Testosterone (3.0 mg; 0.0104 mmol) was dissolved in 3.0 mL of HPLC grade acetonitrile and diluted with 7.38 mL of 5 mM phosphate buffer. The reaction was initiated with 150 equivalents of hypochlorous acid (225 μL of 7 M HOCl) and left to stir for 20 hours. The hypochlorous acid concentration was measured via titration with ferrous ammonium sulfate hexahydrate according to standard methods.[1] The reaction was quenched with water and extracted 3 times with 10 mL of chloroform and the organic phase was evaporated to dryness under a light stream of air. The resulting product mixture was separated by HPLC using an Agilent 1260 Infinity II instrument with a diode array detector. The reverse-phase HPLC method used to isolate the reaction products began with ten minutes at 60% acetonitrile / water, a 1-minute ramp to 80% acetonitrile / water, followed by a 5-minute hold, a 1-minute ramp to 100% acetonitrile and a final 3-minute hold using an Agilent Pursuit XRS C_{18} column (250 x 4.6 mm; 10-μm particle size) at a flow rate of 1 mL/min with UV detection at 210 nm.

*Testosterone Product **1** and **2** Structure Elucidation*

We observed formation of two products (**1** and **2**) at high free chlorine concentrations. Products **1** (2,2-dichloro-4β,5β-epoxy-17β-hydroxyandrost-3-one) (1.6 mg) and **2** (2,2-dichloro-4β,5β-epoxyandrostan-3,17-dione) (0.6 mg) eluted at 13.6 and 15.0 minutes, respectively. HREIMS and NMR data can be found below for the testosterone standard in **Figures S3-S4**, products **1** (2,2-dichloro-4β,5β-epoxy-17β-hydroxyandrost-3-one) in **Figures S5-S8** and **Table S1** and **2** (2,2-dichloro-4β,5β-epoxyandrostan-3,17-dione) in **Figures S9-S12** and **Table S2**. HREIMS produced a molecular ion $M^{+\bullet}$ for product **1** at *m*/z 372.1246, indicating a formula of $C_{19}H_{26}O_3Cl_2$, and for product **2** at *m*/z 370.1117, indicating a formula of $C_{19}H_{24}O_3Cl_2$. Given the difference of two hydrogen atoms and the absence of the oxymethine signal at δ 3.65 in the ^1H NMR spectrum of product **1**, it was concluded that product **2** is an oxidized derivative of product **1**.

^1H NMR analysis showed that both **1** and **2** had two new mutually coupled doublets at δ 2.91 and 2.78 not present in the spectrum of testosterone. HSQC correlations for **2** indicated that the corresponding two protons were attached to the same carbon at δ 47.8. HMBC correlations from these two protons were observed to methyl carbon C-19, C-10, C-5, C-2, and carbonyl C-3, leading to the assignment of these protons as H-1 α and β. ^1H NMR also showed a new singlet at δ 3.37 for both products not observed in the spectrum of testosterone. HSQC data for product **1** showed correlation of this signal to a carbon resonating at δ 61.3, along with HMBC correlations to carbonyl C-3, C-2, C-5, and C-6, allowing assignment of this proton as H-4. The relative downfield shift of non-protonated carbon C-2 along with those of C-4 and C-5 led to the placement of an epoxide bridging C-4 and C-5, with C-2 being doubly chlorinated in both products **1** and **2**.

Spectroscopic data used in the structure elucidation of the two testosterone products (**1** and **2**) observed at high free chlorine concentrations are summarized in **Figures S3-S12** and **Tables S1-S2.** Based on this analysis, the structure of product **1** was assigned as 2,2-dichloro-4β,5β-epoxy-17β-hydroxyandrost-3-one and that of product **2** as 2,2-dichloro-4β,5β-epoxyandrostan-3,17-dione leaving only a question regarding the stereochemical assignment of the new epoxide unit in these products.

Standard NMR approaches were not helpful in assigning the stereochemical orientation of the newly formed epoxide unit due to the small number of hydrogen atoms in the epoxide-bearing portion of the molecule, and the corresponding absence of relevant heteronuclear multiple-bond correlation spectroscopy (NOESY) correlations and *J*-values. Thus, electronic circular dichroism (ECD) and TDDFT calculations were employed in order to make the assignment. The ECD spectrum was calculated for energy-minimized molecular models of each of the two possible diastereomers of product **2** and compared to experimental ECD data. As expected, the two calculated spectra were not mirror images of one another, but the calculated data for the β-configured epoxide clearly matched much better with the experimental spectrum than the data for the α-isomer (**Figure S13**), leading to assignment of the configuration of product **2**, and by analogy, that of product **1**, as shown. A complete narrative of the ECD work is provided below in **Figure S13** and **Table S3**.

Dienogest Reaction

100 mL of 25 μm (7.8 mg/L) dienogest in 5 mM phosphate buffer stock solution was equally apportioned to a set of 5 40-mL amber reaction vessels with Teflon caps. The reactors were dosed with either 0, 0.5, 1, 2.5, or 5 mg Cl_2/L at pH 7 and allowed to react for 5 hours. HPLC analysis of these reactions employed a Beckman System Gold instrument with a model 166P

variable wavelength detector (VWD) connected to a 128P solvent module, with acetonitrile/water gradient elution (25-100% acetonitrile over 11 min; 2 mL/min), an Apollo C_{18} semi-preparative (10 x 250 mm, 5 µm). UV detection of analytes was employed while monitoring at 240 nm**.** Chromatography of the reaction mixtures showed a single product, more polar relative to the parent, with a retention time of 6.3 minutes, which grew in over time.

*Dienogest Product **3** Structure Elucidation*

Dienogest (15.5 mg; 0.0499 mmol) was dissolved in 3.0 mL methanol and added to 400 mL of phosphate buffer (5 mM, pH 7) dosed with 357 µL of 5600 mg/L hypochlorous acid aqueous stock solution and allowed to sit at room temperature for 4 hours. The reaction mixture was extracted with chloroform (50 mL x 3) and the organic phase was evaporated to dryness under a light stream of air to yield 11.8 mg of crude residue. Isolation of the product by the same HPLC method used for the batch reactions resulted in one fraction of product **3** (10.5 mg), identified as 9,10-epoxy-dienogest. HRESIMS and NMR data can be found below in **Figures S14-S23** and **Table S4**.

HRESIMS analysis of the reaction product from dienogest (**3**) gave an $(M+H)^+$ ion at *m*/*z* 328.1867, corresponding to the formula $C_{20}H_{25}NO_3$, indicative of the addition of an oxygen atom. ^1H NMR analysis revealed the conservation of olefinic H-4, while UV analysis showed some disruption of conjugation with a 62 nm hypsochromic shift to λ_{max} 248 nm (**Figure S21**). The HMBC spectrum of product **3** showed all expected correlations from H_3-18 to C-12, C-13, C-14, and C-17. H_2-12 showed key correlations to C-11 and an oxygenated carbon at δ 68.1 (C-9), in addition to C-17. H_2-11 showed key correlations to C-9 and another oxygenated carbon at δ 60.0 (C-10). H-4 showed a key correlation to the same oxygenated carbon (C-10). Together, these results indicate epoxidation across the C-9/C-10 olefin of the parent dienogest resulting in the

product 9,10-epoxy-dienogest. Spectroscopic data used in the structure elucidation of the dienogest product (**3**) are summarized in **Figures S14-S23** and **Table S4**.

17β-Trenbolone Reactions

100 mL of 25 μM (6.8 mg/L) 17β-trenbolone in 5 mmol phosphate buffer stock solution was equally apportioned to a set of 5 40-mL amber reaction vessels with Teflon caps. The reactors were dosed with either 0, 0.5, 1, 2.5, or 5 mg Cl_2/L at pH 7 and allowed to react for 5 hours. HPLC analysis of these reactions employed a Beckman System Gold instrument with a model 166P variable wavelength detector (VWD) connected to a 128P solvent module, with acetonitrile/water gradient elution (25-100% acetonitrile over 11 min; 2 mL/min), an Apollo C_{18} semi-preparative (10 x 250 mm, 5 μm). UV detection of analytes was employed while monitoring at 350 nm. Chromatography of the reaction mixtures showed a single product, less polar relative to the parent, with a retention time of 9.8 minutes which grew in over time.

*17β-Trenbolone Product **4** Structure Elucidation*

17β-trenbolone (13.5 mg; 0.0499 mmol) was dissolved in 3.0 mL methanol and added to 400 mL of phosphate buffer (5 mM, pH 7) dosed with 357 μL of 5600 mg/L hypochlorous acid aqueous stock solution and allowed to sit at room temperature for 4 hours. The reaction mixture was extracted with chloroform (50 mL x 3) and the organic phase was evaporated to dryness under a light stream of air to yield 10.9 mg of crude residue. Isolation of the product by the same HPLC method used for the batch reactions resulted in a single fraction of product **4** (6.5 mg), identified as 4-chloro-17β-trenbolone. HRESIMS and NMR data for product **4** (4-chloro-17β-trenbolone) can be found below in **Figures S24-S27** and **Table S5**.

For 17β-trenbolone, HRESIMS analysis (**Figure S25**) of the chlorination product gave an $(M+H)^+$ ion at *m*/*z* 305.1312, corresponding to the formula $C_{18}H_{21}ClO_2$ and indicative of

replacement of a hydrogen with a chlorine atom. ^{1}H NMR analysis (**Figure S26 and Table S5**) revealed the absence of olefinic H-4, while UV analysis showed retained conjugation with a 12 nm bathochromic shift to λ_{max} 360 nm (**Figure S21**). The HMBC spectrum of product **6 (Figure S27)** showed key correlations from H_2-1 to a ketone carbon at δ 191.7 (C-3), which is shifted upfield relative to C-3 of the parent (δ 202.0). H_2-2 showed key correlations to an olefinic carbon at δ 127.0 (C-4; in addition to olefinic C-10), and H_2-6 showed correlations to the same carbon (C-4), which is shifted downfield relative to C-4 of the parent (δ 123.2). Together, these results indicate replacement of olefinic H-4 with a chlorine atom, which rationalizes the upfield shift of C-3 and the downfield shift of C-4 relative to the parent 17β-trenbolone and allowed identification of product **4** as 4-chloro-17β-trenbolone.

Altrenogest Reaction

Altrenogest (5 mg; 0.0161 mmol) was dissolved in 5 mL acetonitrile and added to 20 mL phosphate buffer (5 mM, pH 7) in a 50-mL round bottom flash and allowed to stir for 24 hours. The resultant product mixture was extracted with chloroform (25 mL x 3) and the combined chloroform fractions were allowed to evaporate to dryness under a light stream of air. The product mixture was solvated with 1 mL acetonitrile and separated by HPLC using an Agilent 1260 Infinity II instrument with a diode array detector. The reverse-phase HPLC method used to isolate the reaction products began with ten minutes at 60% acetonitrile/water, a 1-minute ramp to 80% acetonitrile/water, followed by a 5-minute hold, a 1-minute ramp to 100% acetonitrile and a final 3-minute hold using an Agilent Pursuit XRS C_{18} column (250 x 4.6 mm; 10-µm particle size) at a flow rate of 1 mL/min with UV detection at 210 nm. Product **6** eluted at 6.4 mins and was identified as 4-chloro-altrenogest and product **5** eluted at 12.3 mins and was identified as 11,12-epoxy-altrenogest. HRESIMS and NMR data for the standard can be found below in **Figures S28-S29**,

and **Table S6**, for product **5** (11,12-epoxy-altrenogest) in **Figures S30-S31**, and for product **6** (4-chloro-altrenogest) in **Figures S32-S33**.

*Altrenogest Product **5** Structure Elucidation*

HRESIMS yielded an $(M+H)^+$ ion at *m/z* 345.1617 corresponding to a molecular formula of $C_{21}H_{25}O_2Cl$, indicating the addition of a chlorine atom and loss of a hydrogen atom relative to altrenogest. The ^1H NMR spectrum showed the loss of a singlet at δ 5.68 observed in the spectrum of altrenogest corresponding to H-4. This result was analogous to data collected for other 4-chloro derivatives encountered during the study, leading to assignment of product **5** as 4-chloro-altrenogest.

*Altrenogest Product **6** Structure Elucidation*

HRESIMS yielded an $(M+H)^+$ ion at *m/z* 327.1954 corresponding to a molecular formula of $C_{21}H_{26}O_3$, indicating the addition of an oxygen atom. Aside from some minor impurities, the ^1H NMR spectrum showed the loss of two doublets at δ 6.42 and 6.53 that were observed in the spectrum of altrenogest corresponding to H-11 and H-12. Given these changes, together with a new signal at δ 3.30 consistent with an allylic epoxide proton and a partner presumed to be buried somewhat more upfield, product **6** was assigned as 11,12-epoxy-altrenogest.

Concentration of parent compounds as a function of chlorine concentration after 5 h reaction.

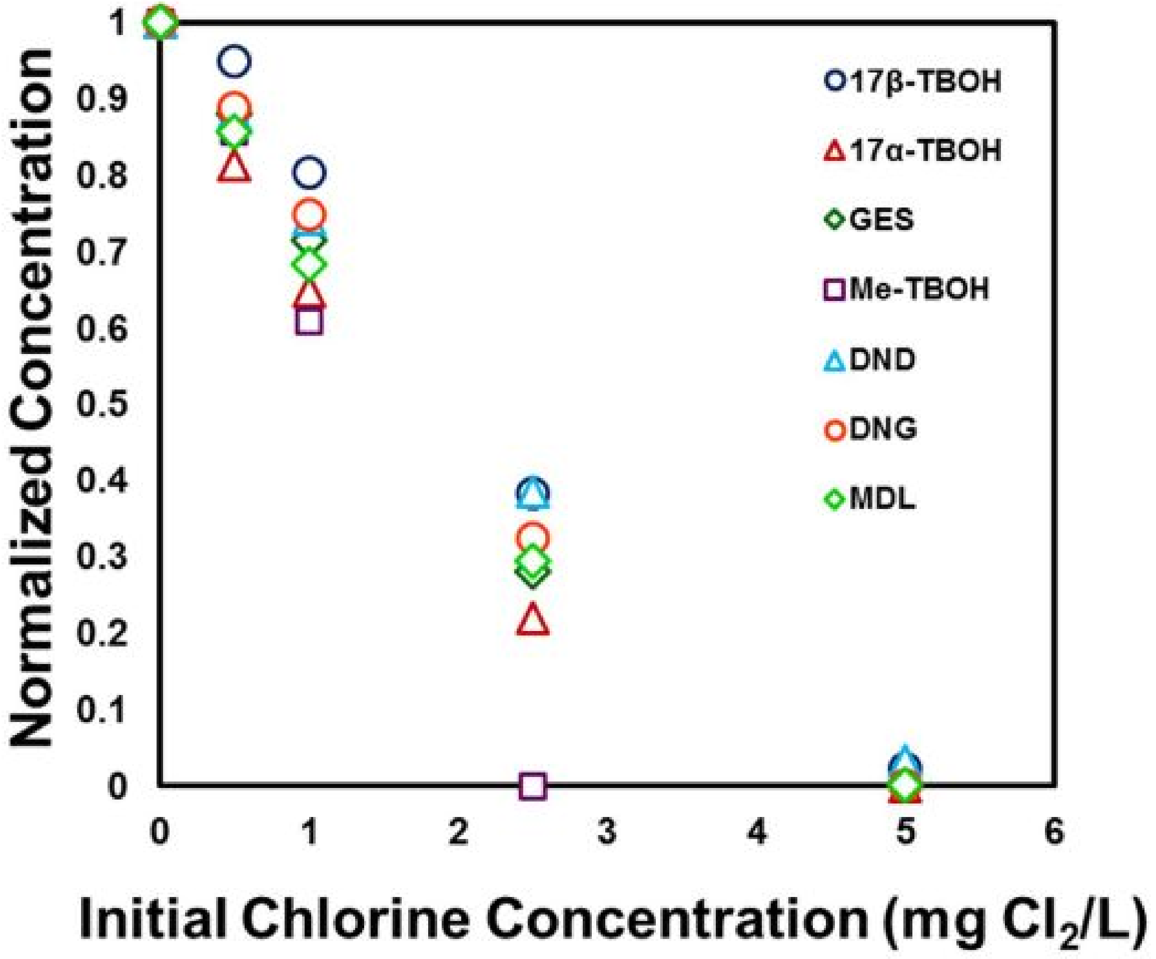

Figure S2. Concentration of 17β-trenbolone (17b-TBOH), 17α-trenbolone (17a-TBOH), gestrinone (GES), methyl-trenbolone (Me-TBOH), dienedione (DND), dienogest (DNG) and methyldienolone (MDL) as a function of initial aqueous chlorine concentration. Reaction conditions: Initial steroid concentration of 25 µM (~ 7-8 mg/L), initial chlorine concentration of 0.5-5 mg Cl2/L, ~ 5h reaction time, and pH 7.

Testosterone Data

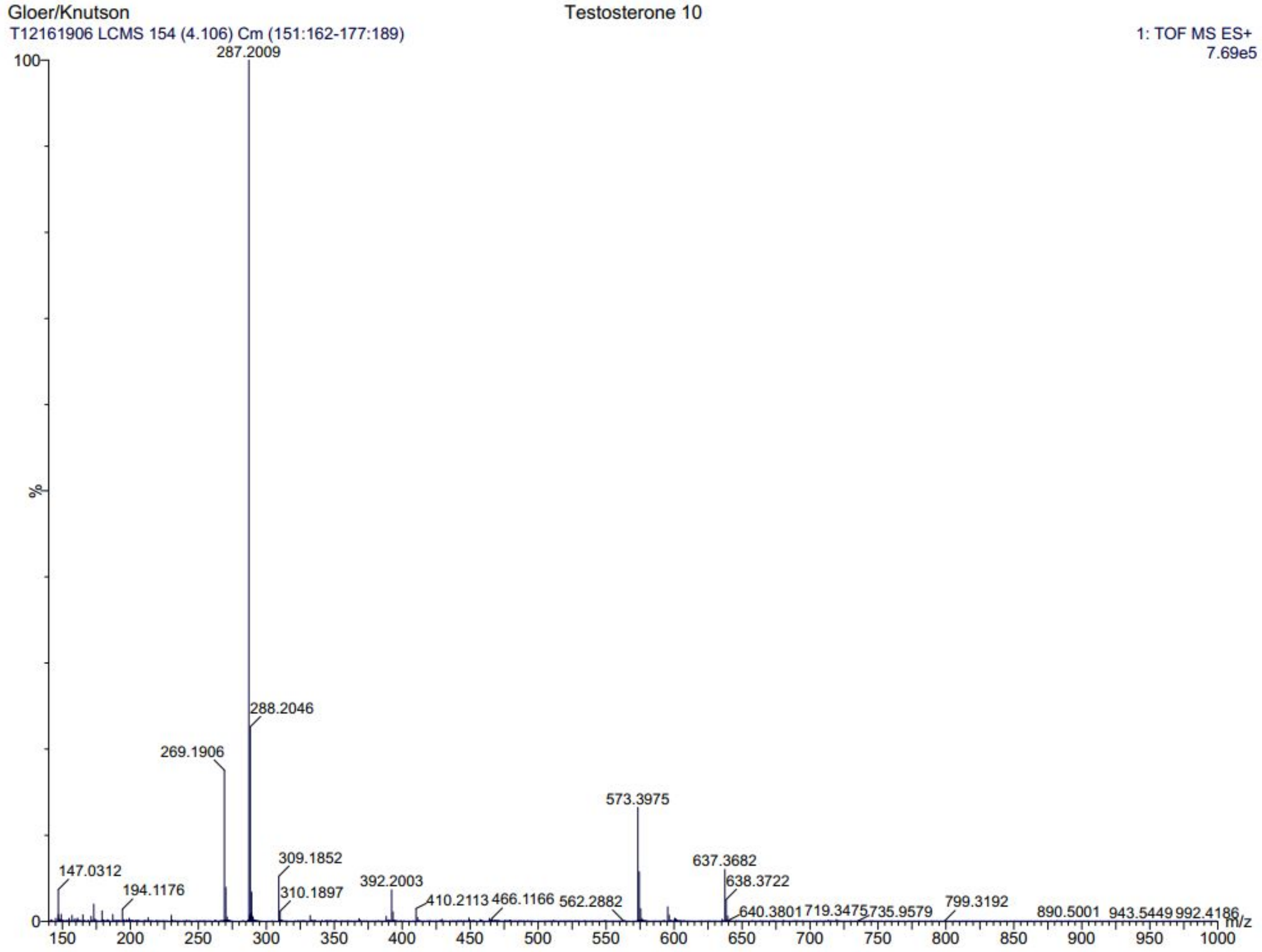

Figure S3. HRESIMS of testosterone with an $M+H^+$ ion at *m*/*z* 287.2009.

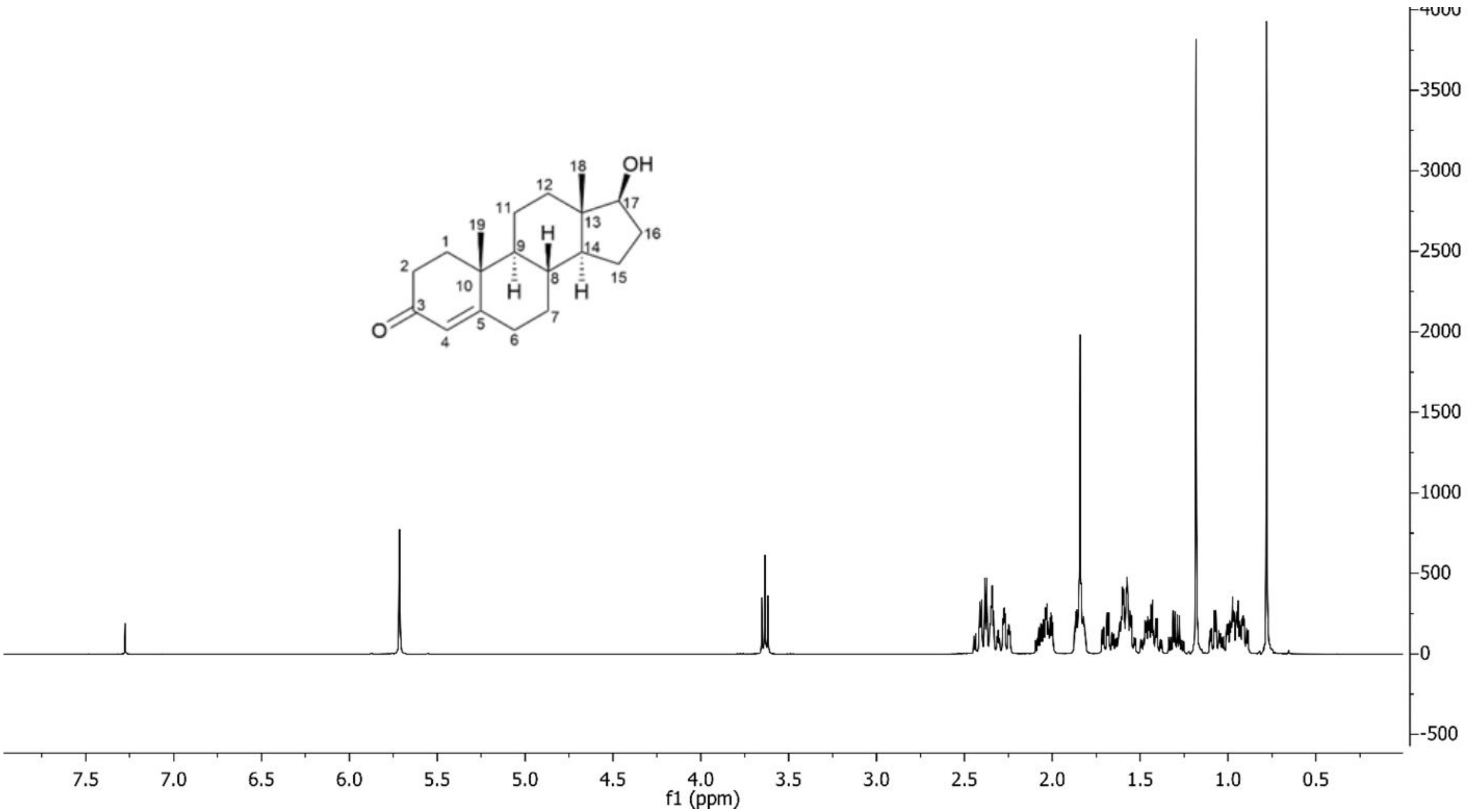

Figure S4. ^{1}H NMR spectrum of testosterone standard in $CDCl_3$ (600 MHz).

Product 1 (2,2-Dichloro-4β,5β-epoxy-17β-hydroxyandrost-3-one) Data.

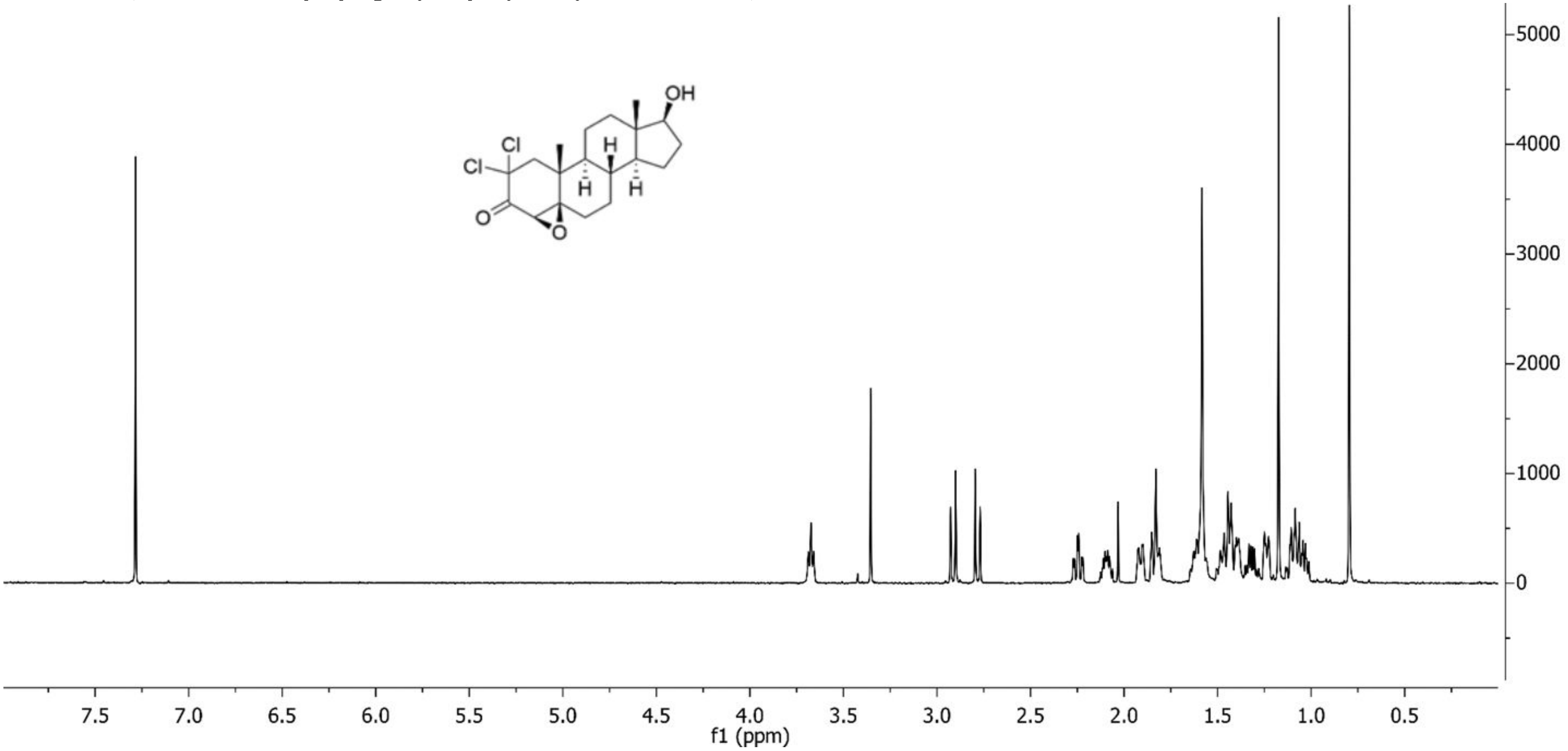

Figure S5. ^{1}H NMR spectrum of product **1** (2,2-dichloro-4β,5β-epoxy-17β-hydroxyandrost-3-one) in $CDCl_3$ (600 MHz).

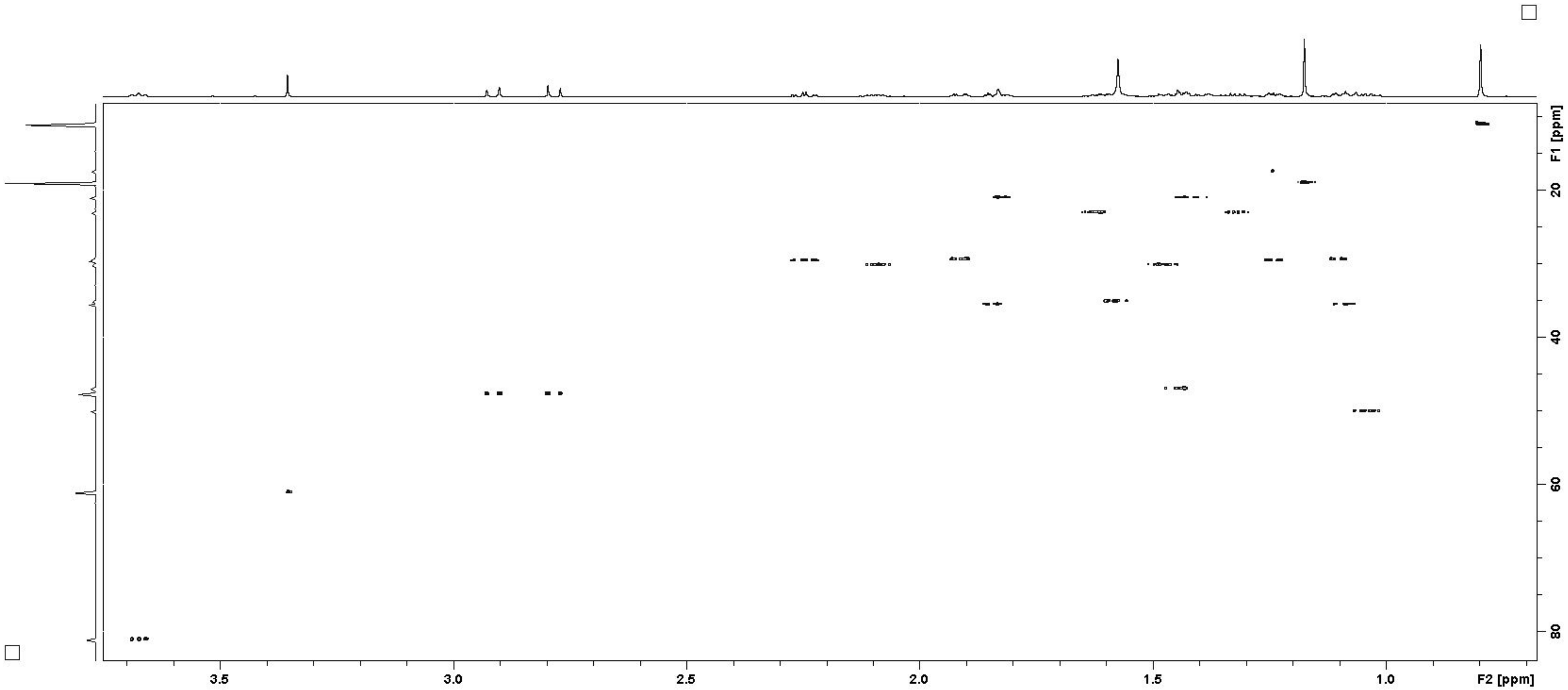

Figure S6. HSQC of product **1** (2,2-dichloro-4β,5β-epoxy-17β-hydroxyandrost-3-one) in $CDCl_3$.

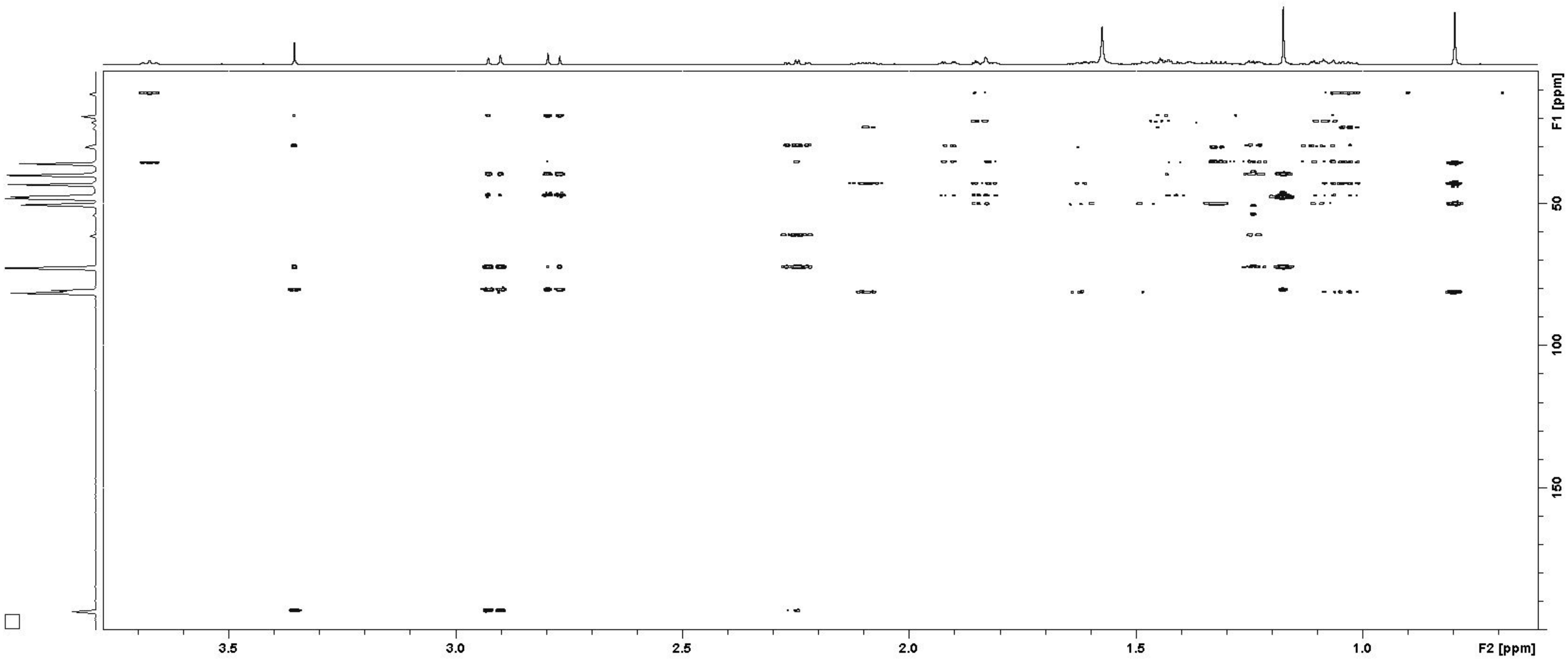

Figure S7. HMBC of product **1** (2,2-dichloro-4β,5β-epoxy-17β-hydroxyandrost-3-one) in $CDCl_3$.

Proton	δ_H mult. (*J* in Hz)	HMBC (H# → C#)	Carbon	δ_C
1α	2.78 *d (*15.9)	2,5,10,19	1	47.8
β	2.91 *d* (15.9)	2,3,5,10,19		
			2	80.5
			3	193.8
4	3.35 *s*	2,3,5,6	4	61.2
			5	72.5
6α	2.25 *td (*13.4, 4.3)	3,4,5,7,8	6	29.6
β	1.24 *m*	4,5,7,8,10,14		
7α	1.08 *m*	9,11,12,14	7	29.4
β	1.91 *dd (*13.4, 3.1)	6,8,9		
8 β	1.59 *m*	13,14	8	35.2
9α	1.45 *m*	11,12,19	9	47.1
			10	39.8
11α	1.81 *m*	12,13,9	11	21.2
β	1.45 *m*	9,12		
12α	1.08 *m*	7,9,11,14,17	12	35.6
β	1.81 *m*	11,13,9,14		
			13	43.1
14α	1.08 *m*	18,15,12,13,17	14	50.3
15α	1.59 *m*	13,14,17	15	23.1
β	1.32 *ddt* (12.1, 6.0, 6.0)	8,14,16		
16α	1.45 *m*	14,15,17	16	30.1
β	2.09 *m*	15,13,17		
17α	3.67 *t (*8.6)	12,18	17	81.2
18-CH_3	0.80 *s*	12,13,14,17	18	11.1
19-CH_3	1.17 *s*	1,2,5,9,10	19	13.1

Table S1. NMR data for product **1** (2,2-dichloro-4β,5β-epoxy-17β-hydroxyandrost-3-one) in $CDCl_3$ (600 MHz).

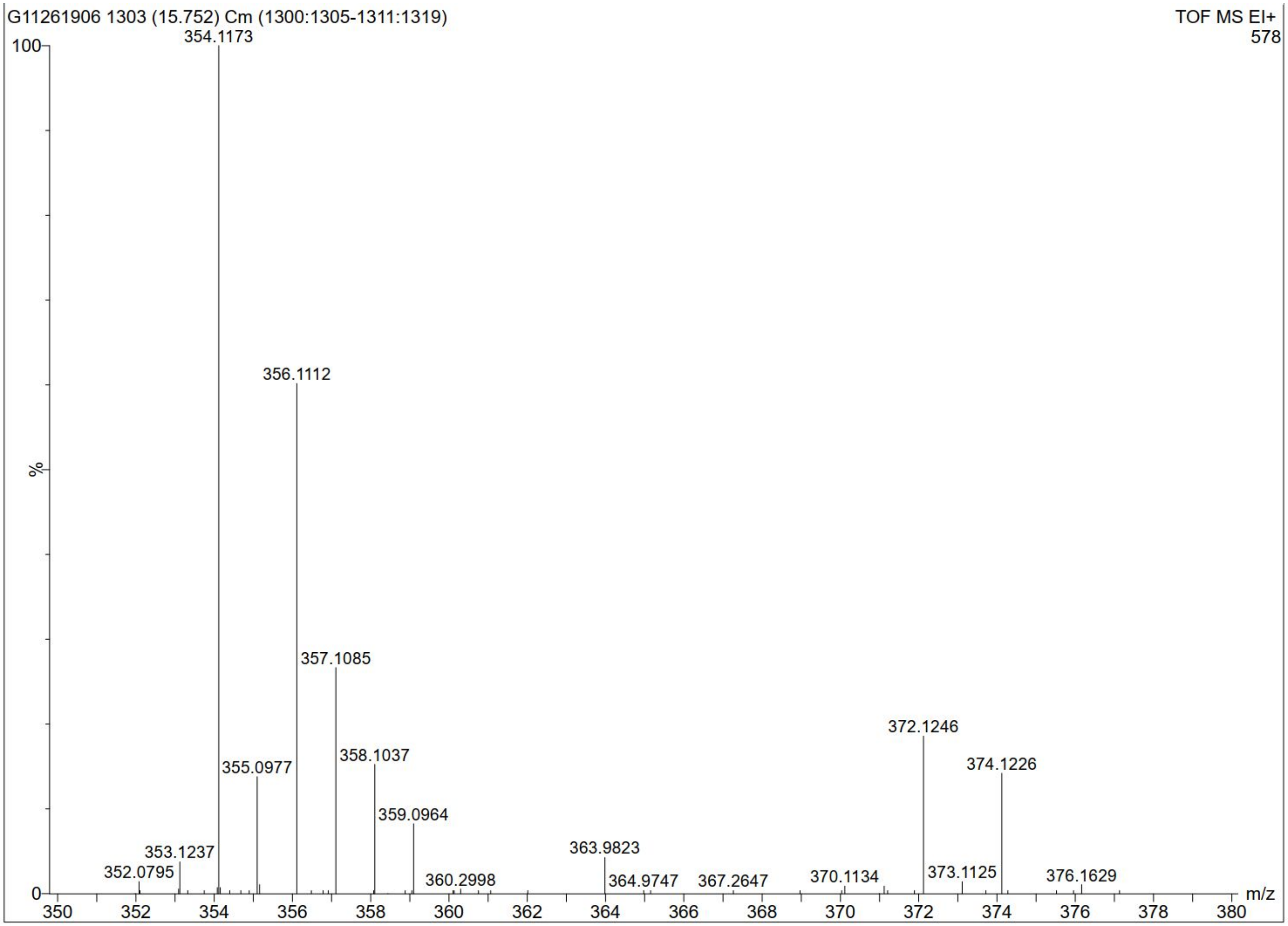

Figure S8. HREIMS of product **1** (2,2-dichloro-4β,5β-epoxy-17β-hydroxyandrost-3-one) produced an $M^{+\bullet}$ ion at *m*/z 372.1246, indicating a formula of $C_{19}H_{26}O_3Cl_2$. An expansion of the peak at *m/z* 372.1246 and the accompanying characteristic chlorine-37 isotope peak has been inset for reference.

Product 2 (2,2-dichloro-4β,5β-epoxyandrostan-3,17-dione) Data.

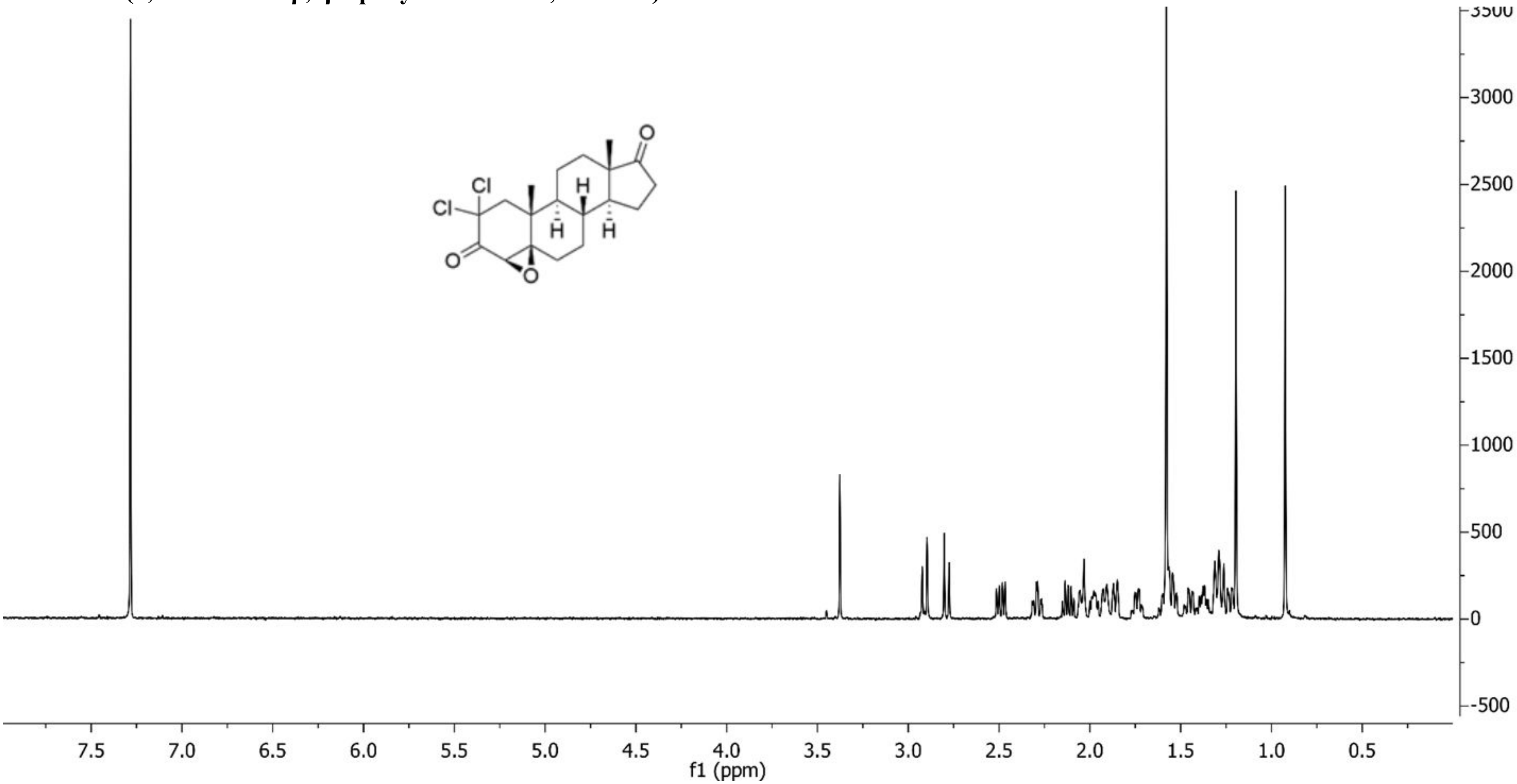

Figure S9. ^{1}H NMR spectrum of product **2** (2,2-dichloro-4β,5β-epoxyandrostan-3,17-dione) in $CDCl_3$ (600 MHz).

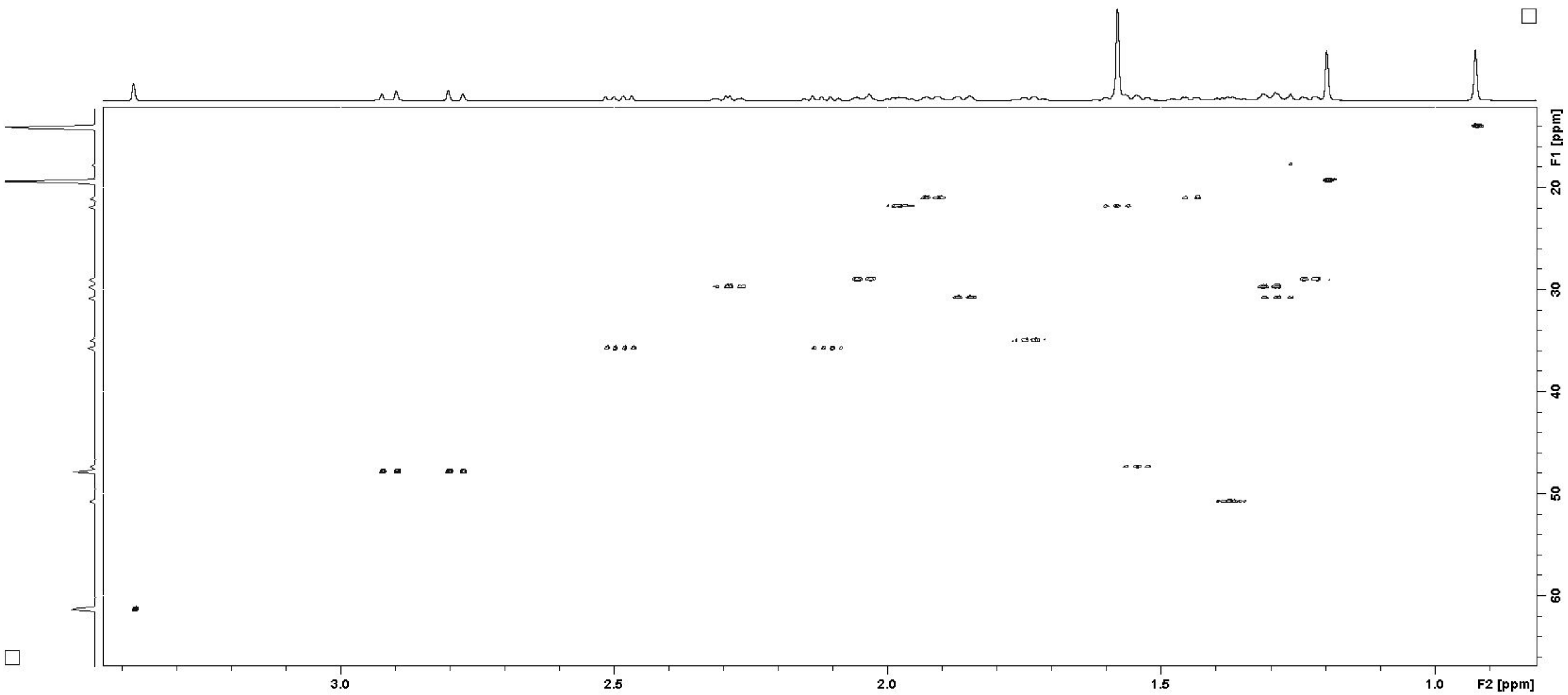

Figure S10. HSQC of product **2** **(**2,2-dichloro-4β,5β-epoxyandrostan-3,17-dione) in $CDCl_3$.

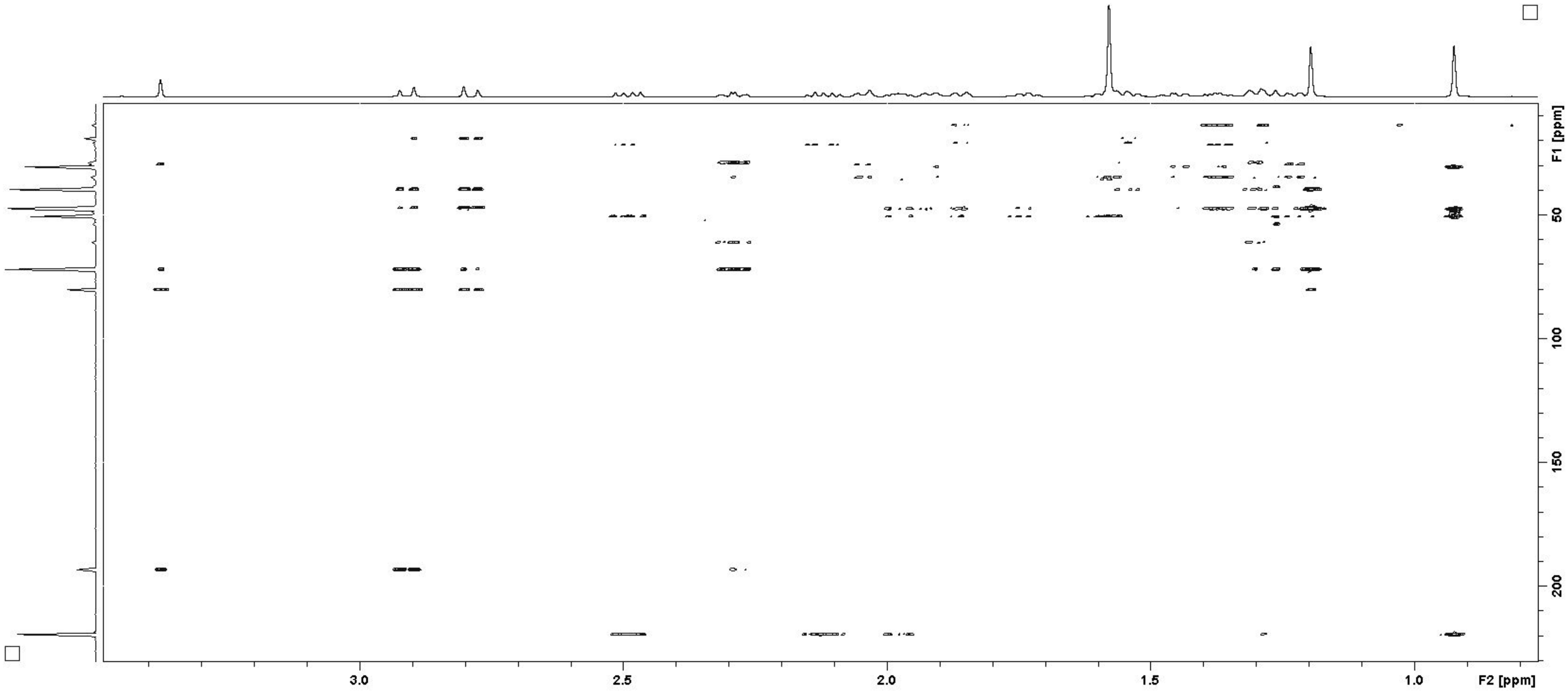

Figure S11. HMBC of product **2** (2,2-dichloro-4β,5β-epoxyandrostan-3,17-dione) in $CDCl_3$.

Proton	δ_H mult. (*J* in Hz)	HMBC (H# → C#)	Carbon	δ_C
1α	2.79 d (16.0)	2,5,9,10,19	1	47.8
β	2.91 d (16.0)	2,3,5,9,10,19		
2			2	80.1
3α			3	193.1
β				
4	3.37 s	2,3,5,6	4	61.3
			5	71.7
6α	2.29 td (13.4, 4.2)	3,4,5,7,8	6	29.3
β	1.29 m	4,7,10		
7α	1.23 dd (12.7, 3.5)	6,8,14	7	28.9
β	2.04 d (14.0)	6,8,10		
8β	1.74 qd (10.9, 3.7)	9,14	8	34.5
9α	1.57 m	10,11,19	9	47.4
10α			10	39.3
11α	1.92 d (12.9)	8,12,13	11	20.9
β	1.46 qd (12.5, 3.4)	12		
12α	1.29 m	5,9,17,18	12	30.5
β	1.86 d (13.3)	5,9,17,18		
13			13	47.2
14α	1.37 td (11.7, 5.6)	13,15,16,18	14	50.4
15α	1.57 m	13,14,16,17		
β	1.98 dt (14.3, 7.7)	8,14	15	21.6
16α	2.12 dt (19.1, 9.0)	15,17	16	35.6
β	2.49 dd (19.1, 9.0)	14,15,17		
17			17	219.1
18	0.92 s	12,13,14,17	18	13.6
19	1.20 s	2,5,9,10	19	19.2

Table S2. NMR data for product **2** (2,2-dichloro-4β,5β-epoxyandrostan-3,17-dione) in $CDCl_3$ (600 MHz).

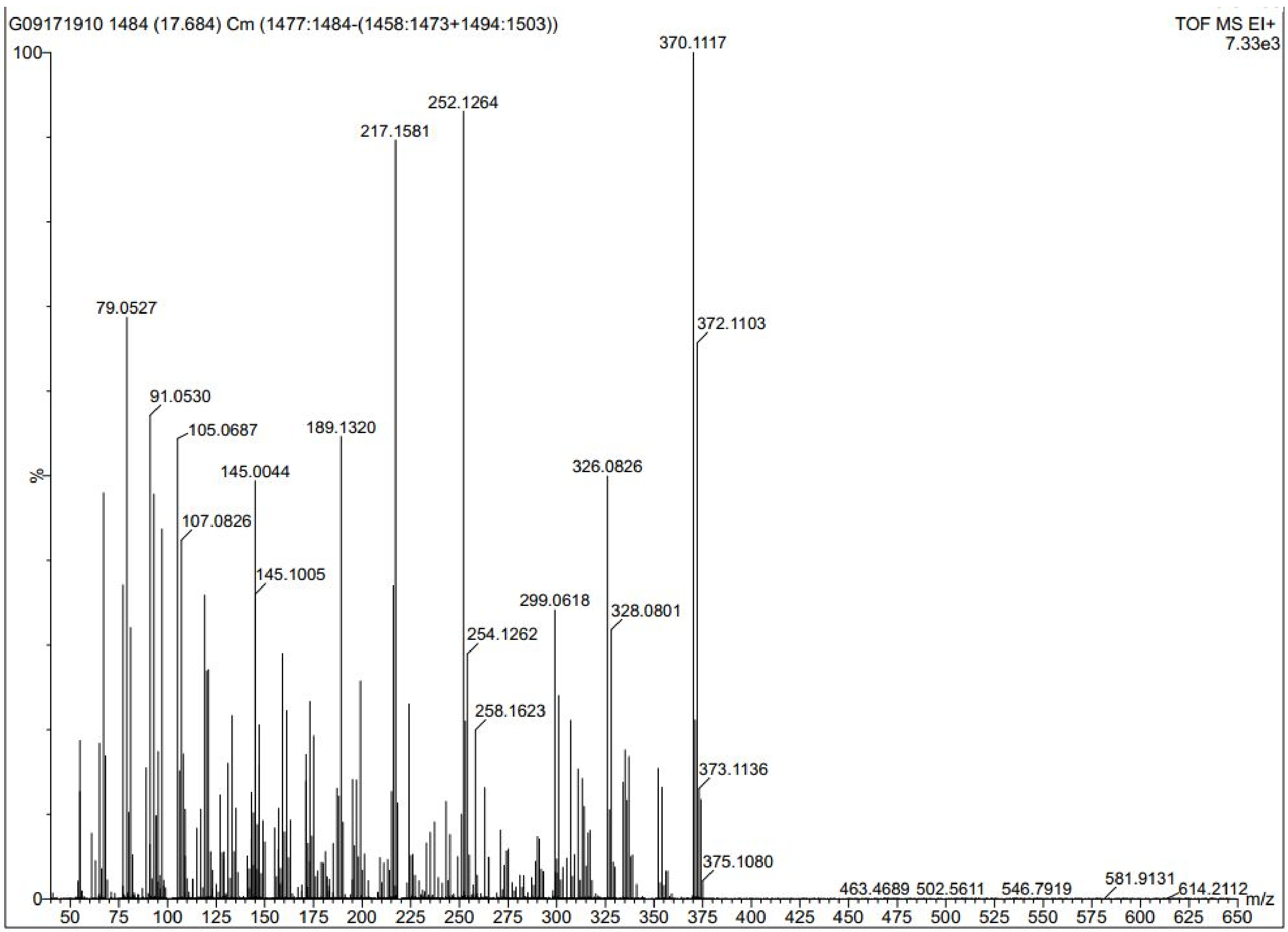

Figure S12. HREIMS of product **2** (2,2-dichloro-4β,5β-epoxyandrostan-3,17-dione) produced an $M^{+\bullet}$ ion at *m*/z 370.1117, indicating a formula of $C_{19}H_{24}O_3Cl_2$.

Product 2 (2,2-dichloro-4β,5β-epoxyandrostan-3,17-dione) ECD Study

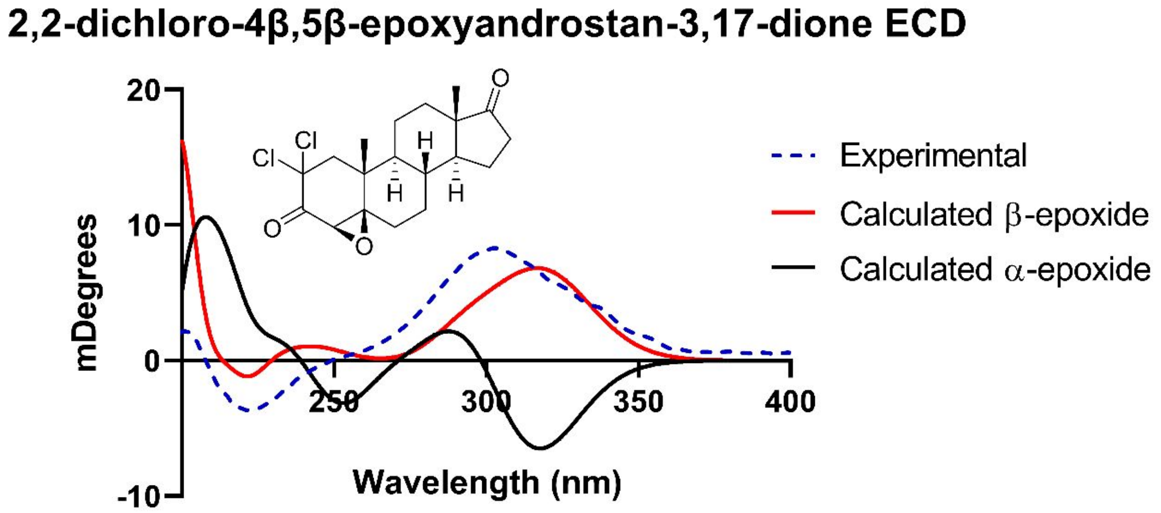

Figure S13. Comparison of the ECD spectrum of 2,2-dichloro-4β,5β-epoxyandrostan-3,17-dione and the calculated spectra of the α and β epoxide isomers

ECD Studies of product ***2*** *(2,2-dichloro-4β,5β-epoxyandrostan-3,17-dione):* The *in silico* portion of the study began with two geometry optimization stages for each molecule. Both were conducted in the gas phase using the resolution-of-identity (RI) approximation. The first optimization was conducted using a BP functional and SV(P) basis set and the corresponding default auxiliary basis set followed by optimization using the larger TZVP basis set and the corresponding default auxiliary basis set in order to increase the optimization accuracy.[2,3] No symmetry constraints were used during the optimization.

ECD spectra for 2,2-dichloro-4β,5β-epoxyandrostan-3,17-dione were calculated for geometries obtained from the RI-BP/TZVP calculations. Time-dependent density functional theory (TDDFT) calculations were used with B3LYP functional [4,5] using the RIJCOSX approximation [6] and TZVP basis set [2] with the corresponding default auxiliary basis set. [7] A total of 80 excited states were calculated, and only singlet excited states were considered. All of the quantum chemical calculations were performed with ORCA version 4.2.1.[8]

ECD data for 2,2-dichloro-4β,5β-epoxyandrostan-3,17-dione were collected using a Jasco J-815 spectropolarimeter running Jasco Spectra Manager Version 2.06.04. 2.7 mmol 2,2-dichloro-4β,5β-epoxyandrostan-3,17-dione in MeOH (0.6 mg in 600 μL MeOH) was used for analysis. Instrument access was courtesy of the Dr. Ernesto Fuentes lab, Department of Biochemistry, University of Iowa. ECD spectra were plotted using SpecDis version 1.71 software.[9] A broadening factor of 0.25 was used to match the resolution level of the experimental data as closely as possible.

**α-epoxide**				**β-epoxide**			
Cl1	-0.98919	5.62869	-0.07591	Cl1	-2.30467	3.09703	-0.90577
Cl2	-2.73593	3.34557	0.37475	Cl2	-1.15273	5.42421	0.47789
O3	-1.29818	1.6282	2.26666	O3	0.78575	2.94294	2.6541
O4	-0.61369	5.06314	2.74922	O4	-2.76131	3.10162	2.04021
O5	-1.52262	-3.56135	-3.2187	O5	-1.81033	-3.35435	-3.086
C6	1.21876	-2.27149	-2.21245	C6	1.06091	-2.34254	-2.1335
C7	1.9801	1.73061	0.26942	C7	2.26858	1.70042	0.11012
C8	-0.2428	-3.427	0.18603	C8	-0.4861	-3.29355	0.30414
C9	-0.94061	-4.23422	-0.92951	C9	-1.28264	-4.03964	-0.78781
C10	-0.98934	-3.30672	-2.15769	C10	-1.24328	-3.13639	-2.03439
C11	-0.7776	-0.72369	-2.415	C11	-0.76406	-0.59454	-2.34423
C12	-0.24977	-2.01451	-1.79596	C12	-0.36662	-1.92339	-1.70611
C13	-0.45919	-1.97222	-0.26711	C13	-0.54724	-1.83232	-0.17522
C14	-0.09975	0.49587	-1.76472	C14	0.05499	0.55261	-1.7292
C15	0.74876	0.38084	2.61555	C15	1.00109	0.42224	2.60779
C16	0.1713	-0.86686	1.92392	C16	0.19979	-0.7276	1.98338
C17	0.29952	-0.82459	0.39345	C17	0.3391	-0.76287	0.45666
C18	-0.22234	0.50774	-0.2291	C18	-0.03613	0.60004	-0.19237
C19	0.44547	1.74396	0.46129	C19	0.76095	1.78412	0.43158
C20	-1.05307	3.96628	0.60855	C20	-1.10347	3.61678	0.33631
C21	-0.46715	2.69665	2.75486	C21	-0.56801	2.43926	2.57933
C22	-0.72405	4.02845	2.12724	C22	-1.5925	3.08593	1.7126
C23	0.10672	1.57632	1.94952	C23	0.5698	1.70868	1.94973
C24	-0.00629	3.12146	-0.11932	C24	0.31201	3.18481	-0.05117
H25	1.62167	-3.19604	-1.77707	H25	1.36726	-3.29866	-1.68738
H26	1.27099	-2.36556	-3.30587	H26	1.08996	-2.45784	-3.22581
H27	1.87732	-1.44808	-1.90801	H27	1.80712	-1.58987	-1.84845
H28	2.45193	2.48349	0.91779	H28	2.82654	2.42491	0.72002
H29	2.23004	1.98559	-0.76941	H29	2.45139	1.94051	-0.94554

H30	2.43552	0.75996	0.49096	H30	2.68266	0.70336	0.30231
H31	-0.66978	-3.62695	1.17713	H31	-0.92033	-3.42761	1.3034
H32	0.83024	-3.6658	0.23972	H32	0.55493	-3.64797	0.35103
H33	-1.98016	-4.48324	-0.6652	H33	-2.3402	-4.16584	-0.50765
H34	-0.45116	-5.18306	-1.18991	H34	-0.90464	-5.04193	-1.0333
H35	-1.86638	-0.66785	-2.25283	H35	-1.83711	-0.4163	-2.16545
H36	-0.61994	-0.716	-3.50347	H36	-0.62606	-0.62557	-3.43496
H37	-0.55746	1.40266	-2.18434	H37	-0.30363	1.5035	-2.1461
H38	0.96224	0.5271	-2.05639	H38	1.10883	0.45723	-2.03383
H39	0.4967	0.37673	3.68527	H39	0.83357	0.48765	3.69208
H40	1.84391	0.41631	2.53403	H40	2.08072	0.26684	2.45498
H41	-0.89593	-0.93839	2.19057	H41	-0.86674	-0.60621	2.24354
H42	0.65979	-1.77279	2.31166	H42	0.52315	-1.68715	2.413
H43	-0.30605	2.73097	3.83725	H43	-0.97901	2.07465	3.5259
H44	-0.32677	3.01975	-1.16262	H44	0.42118	3.27152	-1.1388
H45	0.88179	3.77003	-0.14201	H45	0.99888	3.91091	0.40021
H46	-1.53729	-1.75887	-0.11622	H46	-1.59372	-1.49902	-0.01666
H47	-1.29806	0.57265	0.01437	H47	-1.10063	0.7657	0.0603
H48	1.3678	-0.93376	0.13962	H48	1.39194	-0.99631	0.21904

Table S3. Cartesian coordinates of the optimized models of α and β epoxide isomers of product **2** at RI-BP/TZVP level.

Dienogest data

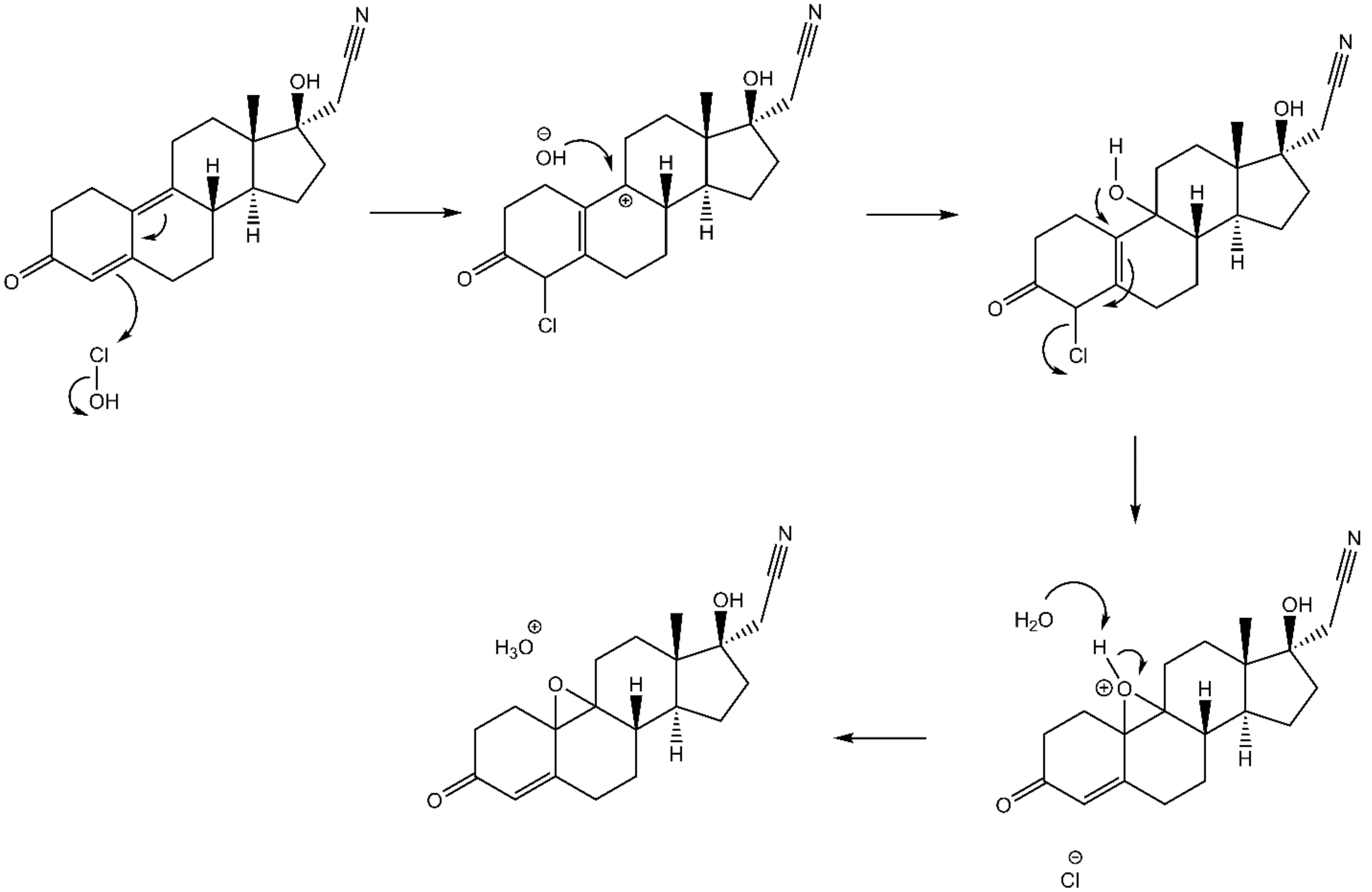

Figure S14. Proposed mechanism of dienogest to product 3 (9,10-epoxydienogest) transformation.

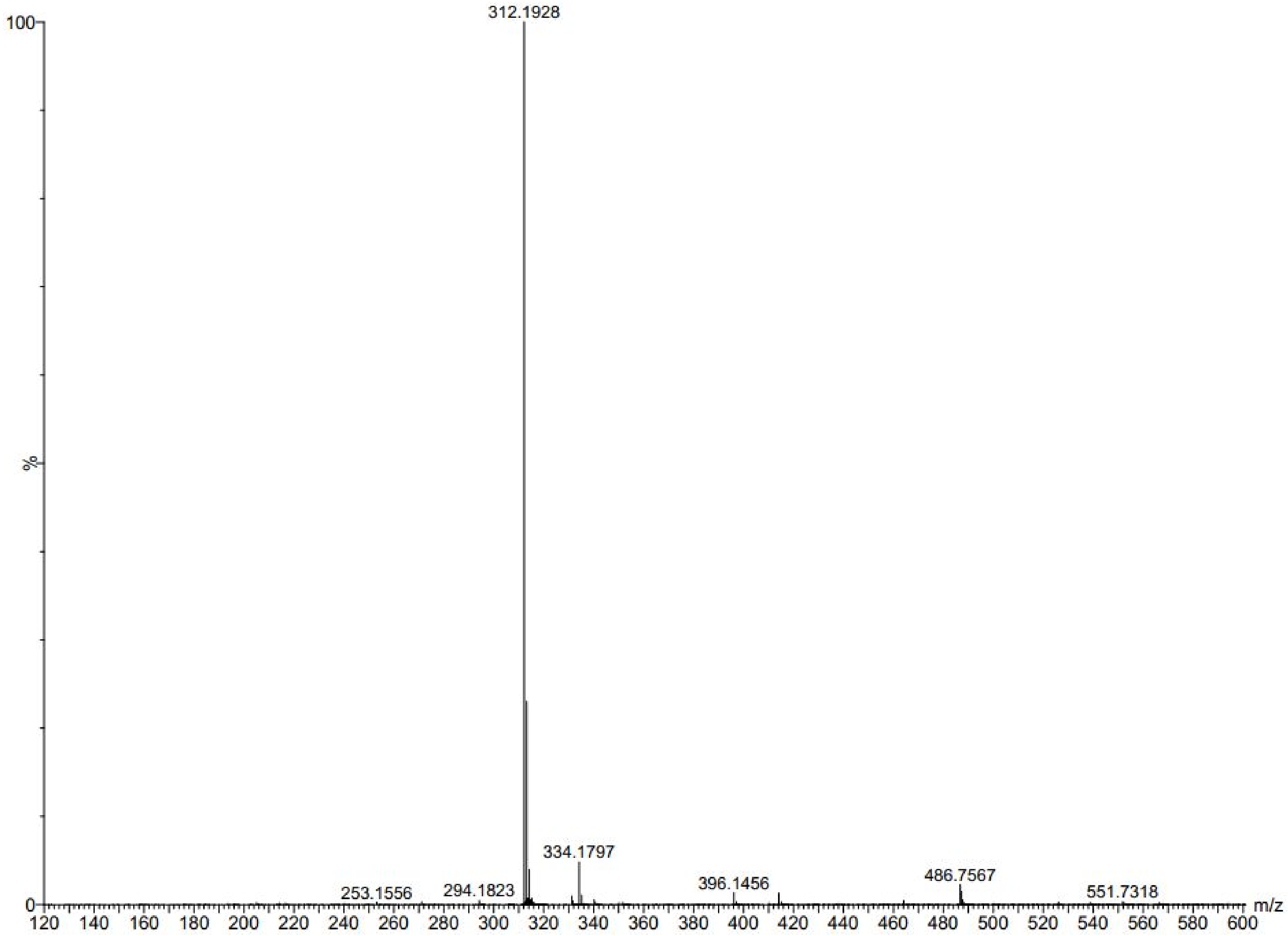

Figure S15. HRESIMS of dienogest standard with an $(M+H)^+$ ion at *m*/*z* 312.1928.

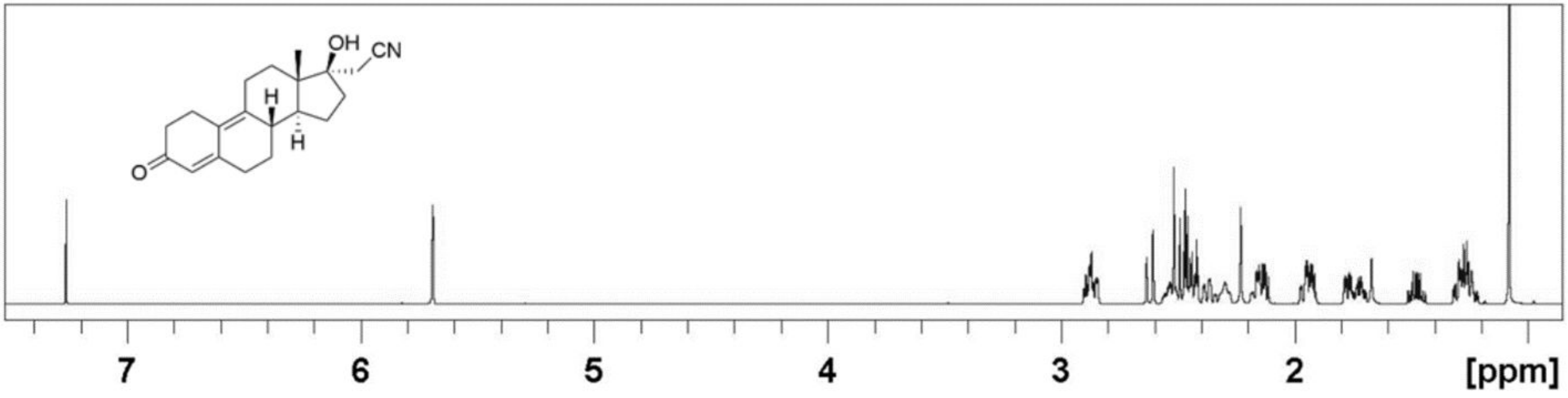

Figure S16. ^{1}H NMR of dienogest standard in $CDCl_3$ (600 MHz).

Product 3 (9,10-epoxy-dienogest) Data

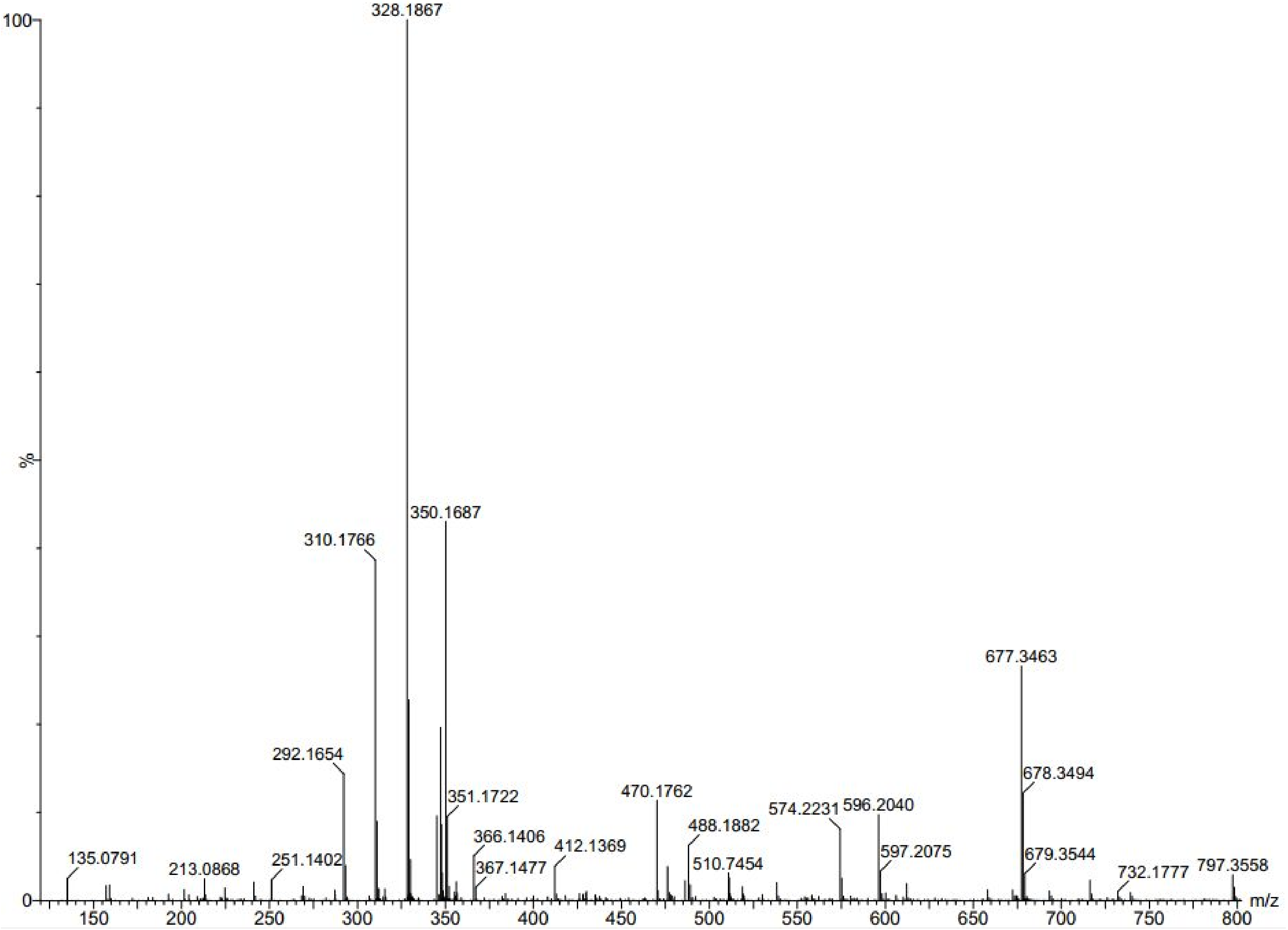

Figure S17. HRESIMS of product **3** (9,10-epoxy-dienogest) with an $(M+H)^+$ ion at *m*/*z* 328.1867, corresponding to the formula $C_{20}H_{25}NO_3$ along with minor impurities.

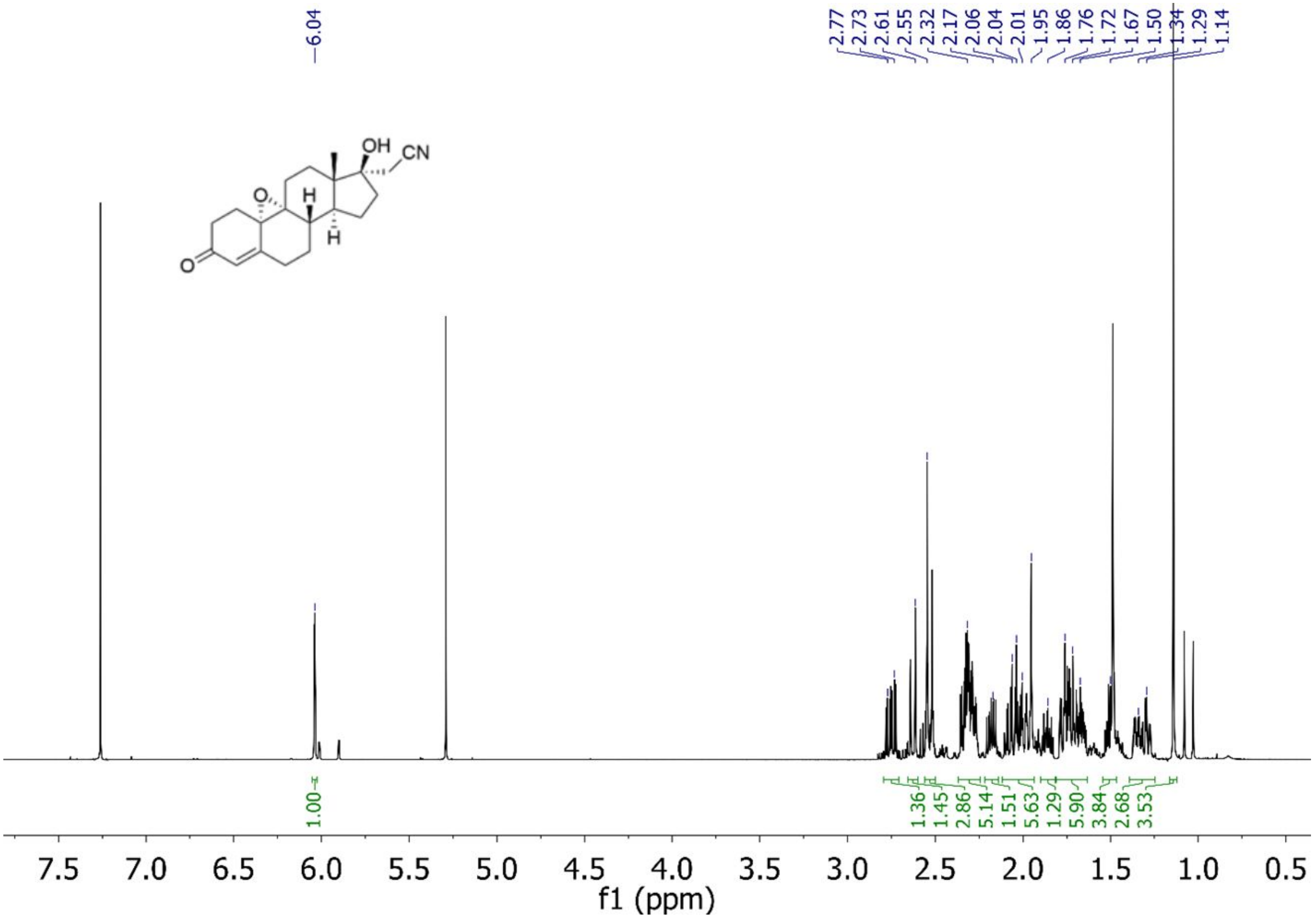

Figure S18. ^{1}H NMR spectrum of product **3** (9,10-epoxy-dienogest) with minor impurities in $CDCl_3$ (600 MHz).

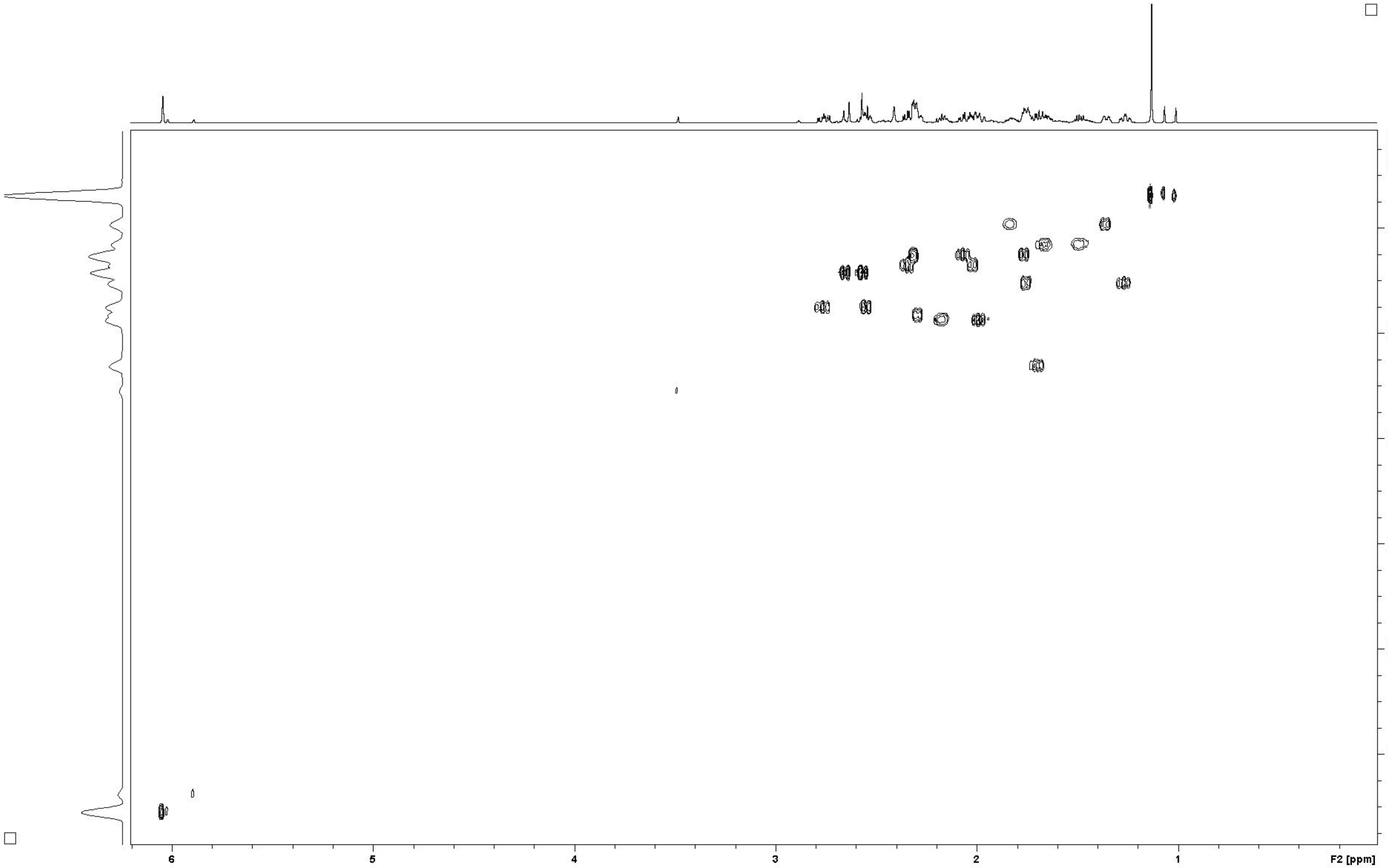

Figure S19. HSQC spectrum of product **3** (9,10-epoxy-dienogest) in $CDCl_3$ (600 MHz).

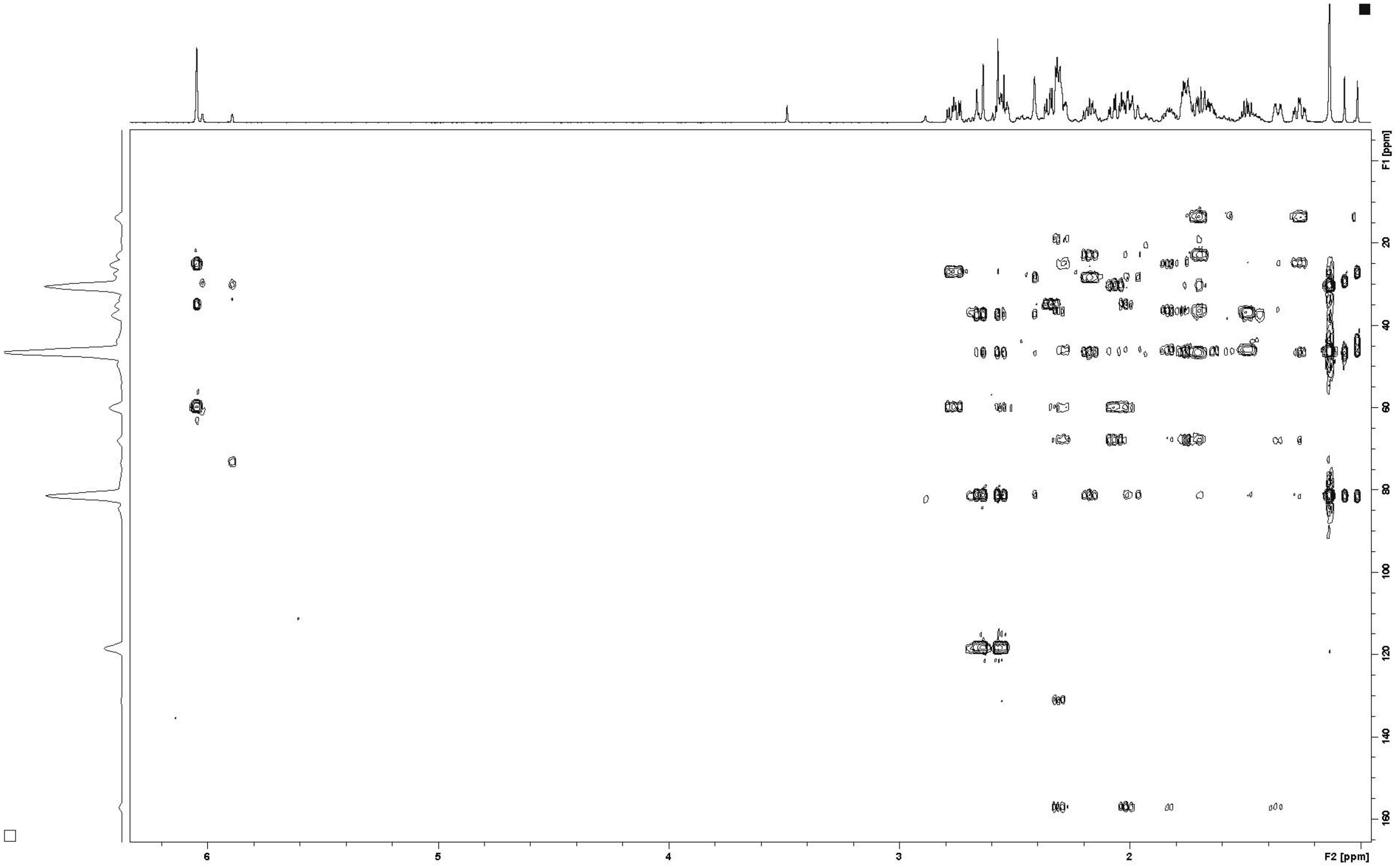

Figure S20. HMBC spectrum of product **3** (9,10-epoxy-dienogest) in $CDCl_3$ (600 MHz).

Proton	δ_H mult. (*J* in Hz)	HMBC (H# → C#)	Carbon	δ_C
1	1.99 m	2,3,5,9,10	1	27.0
	2.33 dt (13.6, 4.7)		2	35.3
2	2.52 m	1,3,10	3	198.9
	2.75 ddd (17.6, 13.2, 5.3)		4	131.0
4	6.03 s	2,6,10	5	157.3
6	2.29 m	4,5,7,8,10	6	25.2
	2.31 m		7	19.1
7	1.34 m	5,6,8,9,14	8	36.6
	1.81 m		9	68.1
8	2.26 m	6,9,10,14	10	60.0
11	1.75 m	8,9,10,12,13	11	25.1
	2.05 dt (13.9, 4.2)		12	30.5
12	1.25 dt (13.9, 4.7)	9,11,13,17,18	13	46.9
	1.73 m		14	46.2
14	1.68 m	7,8,9,12,13,15,17,18	15	23.2
15	1.49 m	8,13,14,16,17	16	37.5
	1.63 m		17	81.6
16	1.95 m	13,14,15,17,20	18	13.8
	2.16 ddd (15.4, 9.3, 7.0)		20	28.6
18	1.12 s	12,13,14,17	21	118.6
20	2.54 d (16.2)	13,16,17,21		
	2.64 d (16.2)			

Table S4. NMR data for product **3** (9,10-epoxy-dienogest) in $CDCl_3$ (600 MHz).

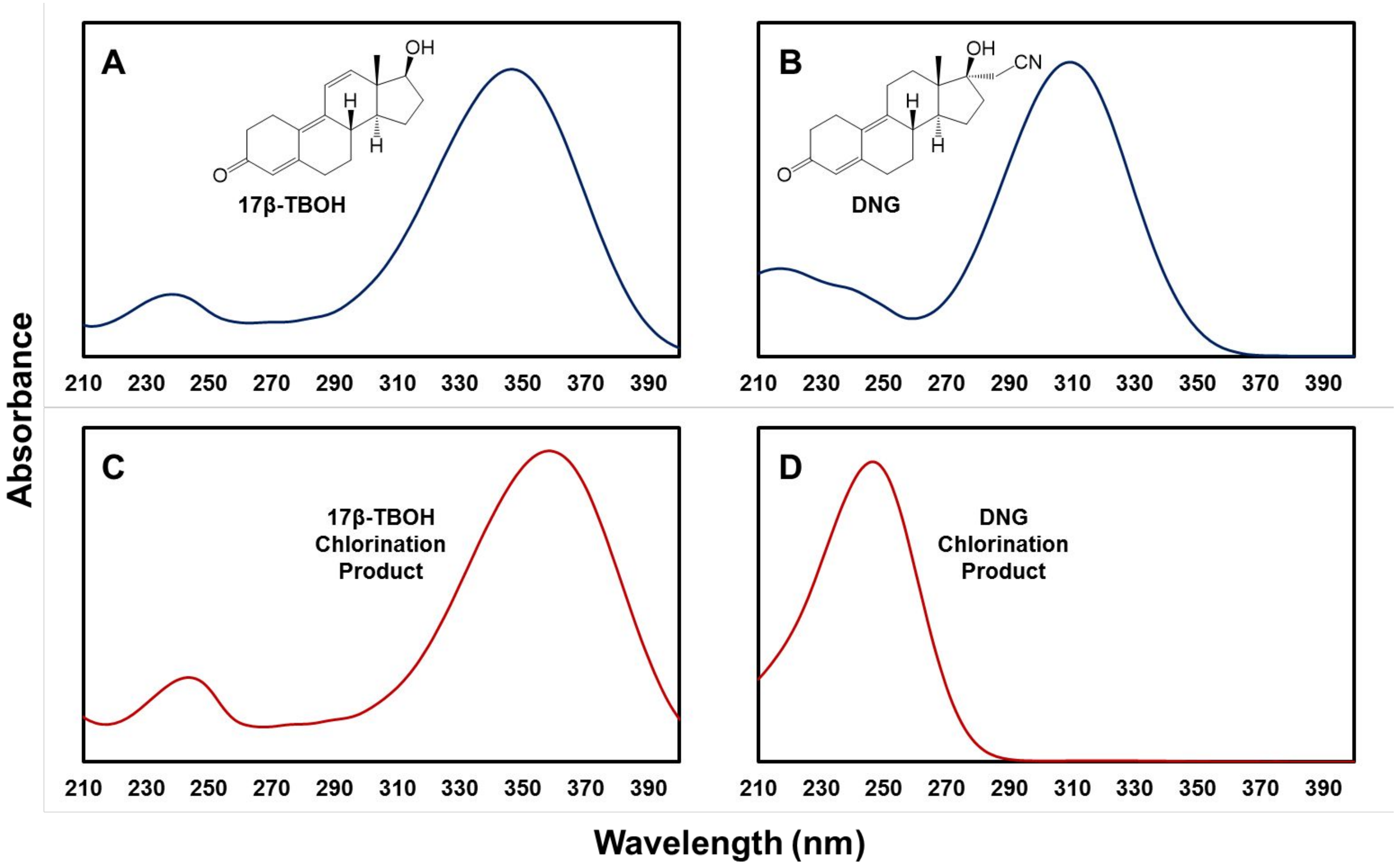

Figure S21. UV-VIS spectrum of (A) 17β-trenbolone standard, (B) dienogest standard, (C) 17β-trenbolone chlorination product (**4**), and (D) dienogest chlorination product (**3**).

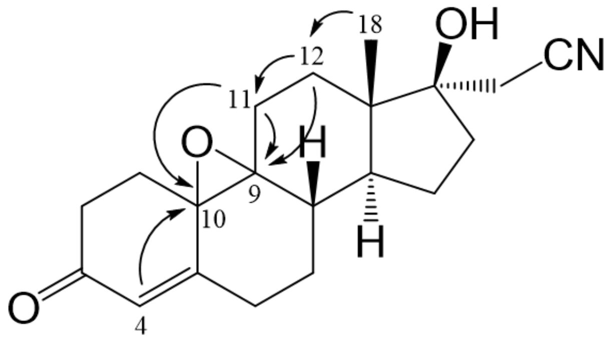

9,10-epoxy-dienogest
Calc'd (M+H)$^{+}$: 328.1912
Exp (M+H)$^{+}$: 328.1867

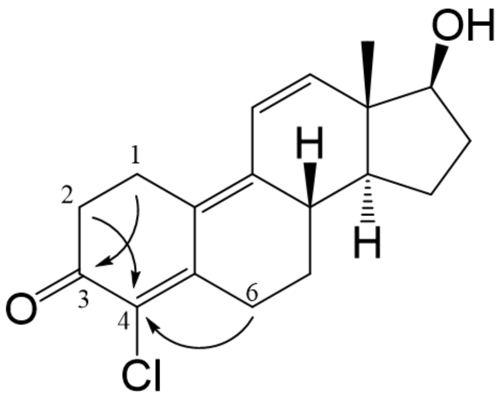

4-chloro-17β-trenbolone
Calc'd (M+H)$^{+}$: 305.1308
Exp (M+H)$^{+}$: 305.1312

Figure S22. Key HMBC correlations and HRESIMS data for product **3** (9,10-epoxy-dienogest) and product **4** (4-chloro-17β-trenbolone).

Dienogest Product 3 (9,10-epoxy-dienogest) as a Function of pH

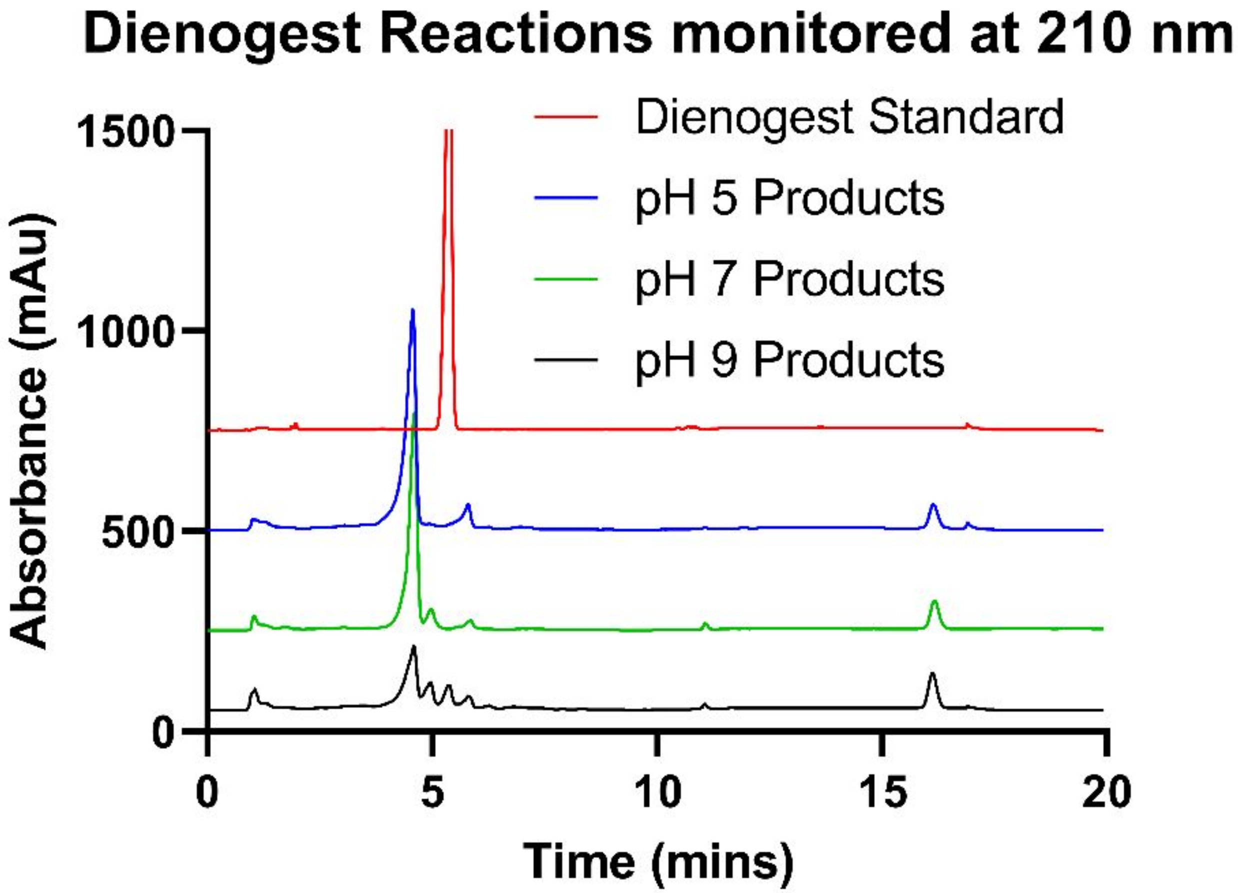

Figure S23. Dienogest product **3** (9,10-epoxy-dienogest) distribution changes as a function of pH.

17β-trenbolone data

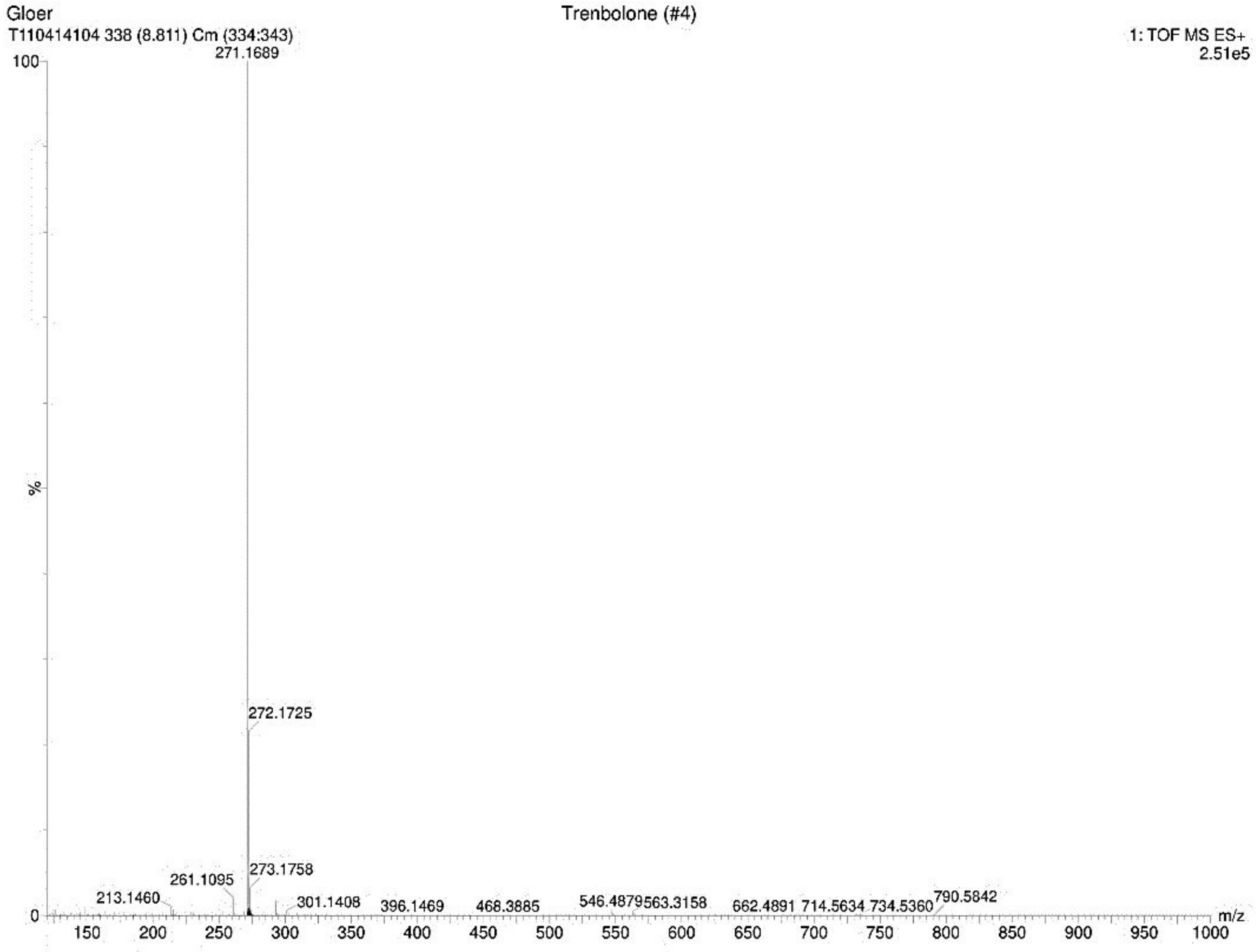

Figure S24. HRESIMS data for 17β-trenbolone standard gave an $(M+H)^+$ ion at *m/z* 271.1689.

Product 4 (4-Chloro-17β-Trenbolone) Data

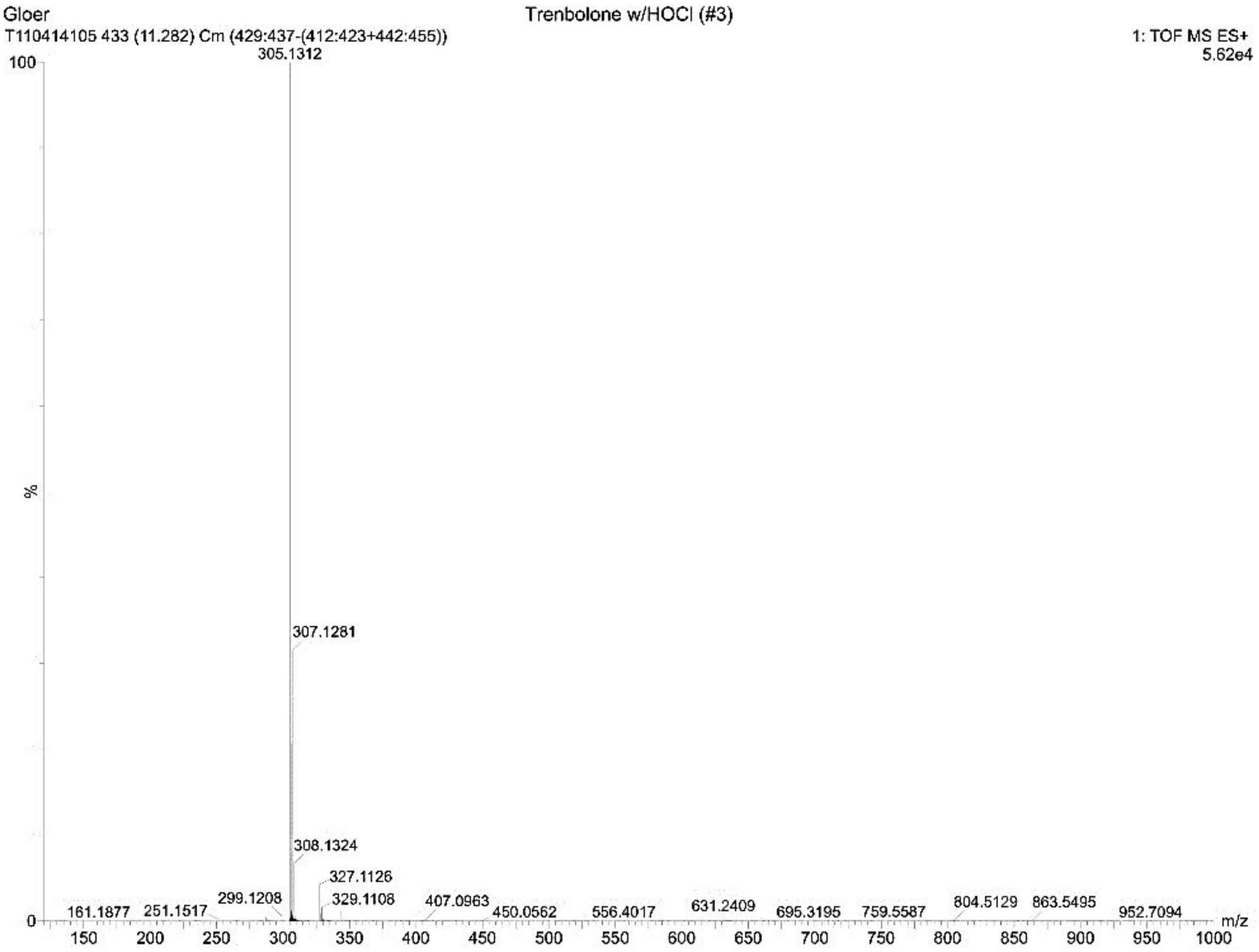

Figure S25. HRESIMS data for product **4** (4-chloro-17β-trenbolone) gave an $(M+H)^+$ ion at *m*/*z* 305.1312.

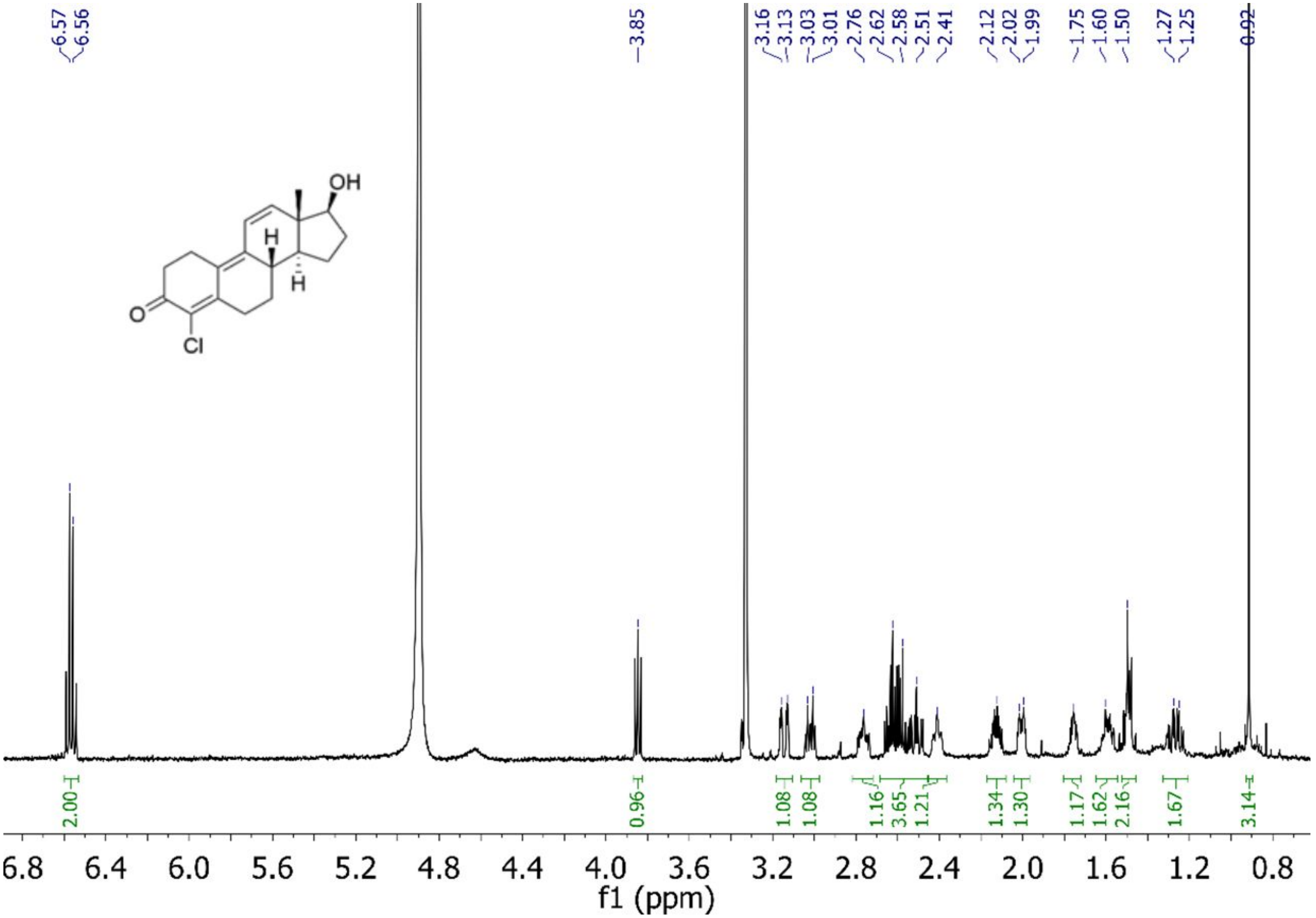

Figure S26. ^{1}H NMR spectrum of product **4** (4-chloro-17β-trenbolone) in CD_3OD (600 MHz).

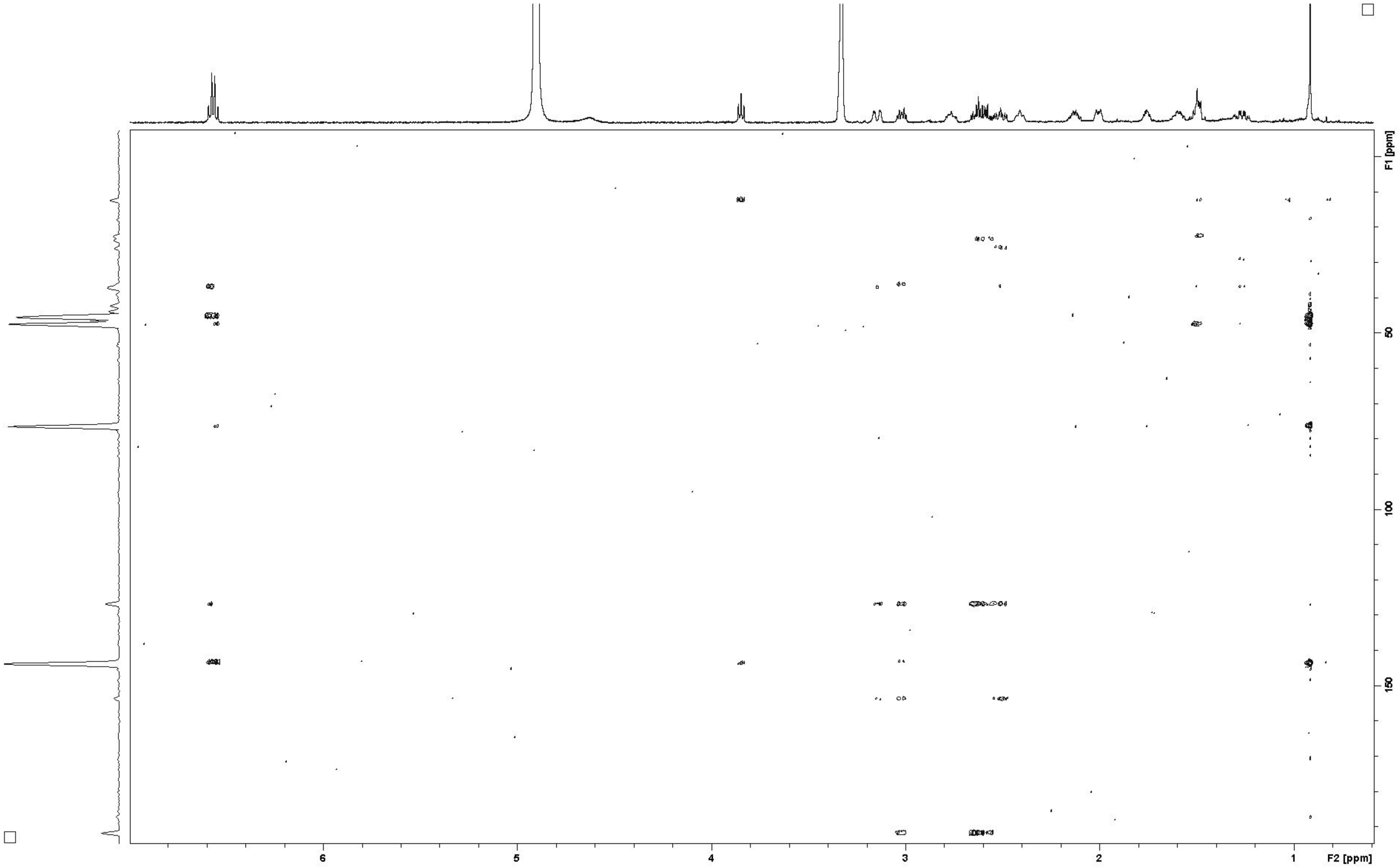

Figure S27. HMBC spectrum of product **4** (4-chloro-17β-trenbolone) in CD_3OD (600 MHz).

Proton	δ_H mult. (J in Hz)	HMBC (H# → C#)	Carbon	δ_C
1	2.75 m	2,3,5,9,10	1	23.4
	3.00 dt (15.0, 5.7)		2	36.3
2	2.57 m	1,3,4,10	3	191.7
	2.61 m		4	127.0
6	2.49 ddd (18.6, 13.9, 4.9)	4,5,7,8,10	5	153.6
	3.13 m		6	29.2
7	1.25 ddd (17.3, 12.9, 5.2)	6,8,14	7	25.8
	1.99 m		8	36.9
8	2.40 bt (10.0)	11	9	143.4
11	6.56 d (9.8)	8,9,10,13	10	126.7
12	6.53 d (9.8)	9,13,14,17	11	122.7
14	1.48 m	8,15,16,18	12	143.7
15	1.46 m	8,13,14,17	13	45.2
	1.74 m		14	47.3
16	1.57 m	13,17	15	22.5
	2.11 m		16	26.3
17	3.83 t (8.5)	12,18	17	76.4
18	0.90 s	12,13,14,17	18	12.2
17-OH	4.62 s			

Table S5. NMR data for product **4** (4-chloro-17β-trenbolone) in CD_3OD (600 MHz).

Altrenogest Data

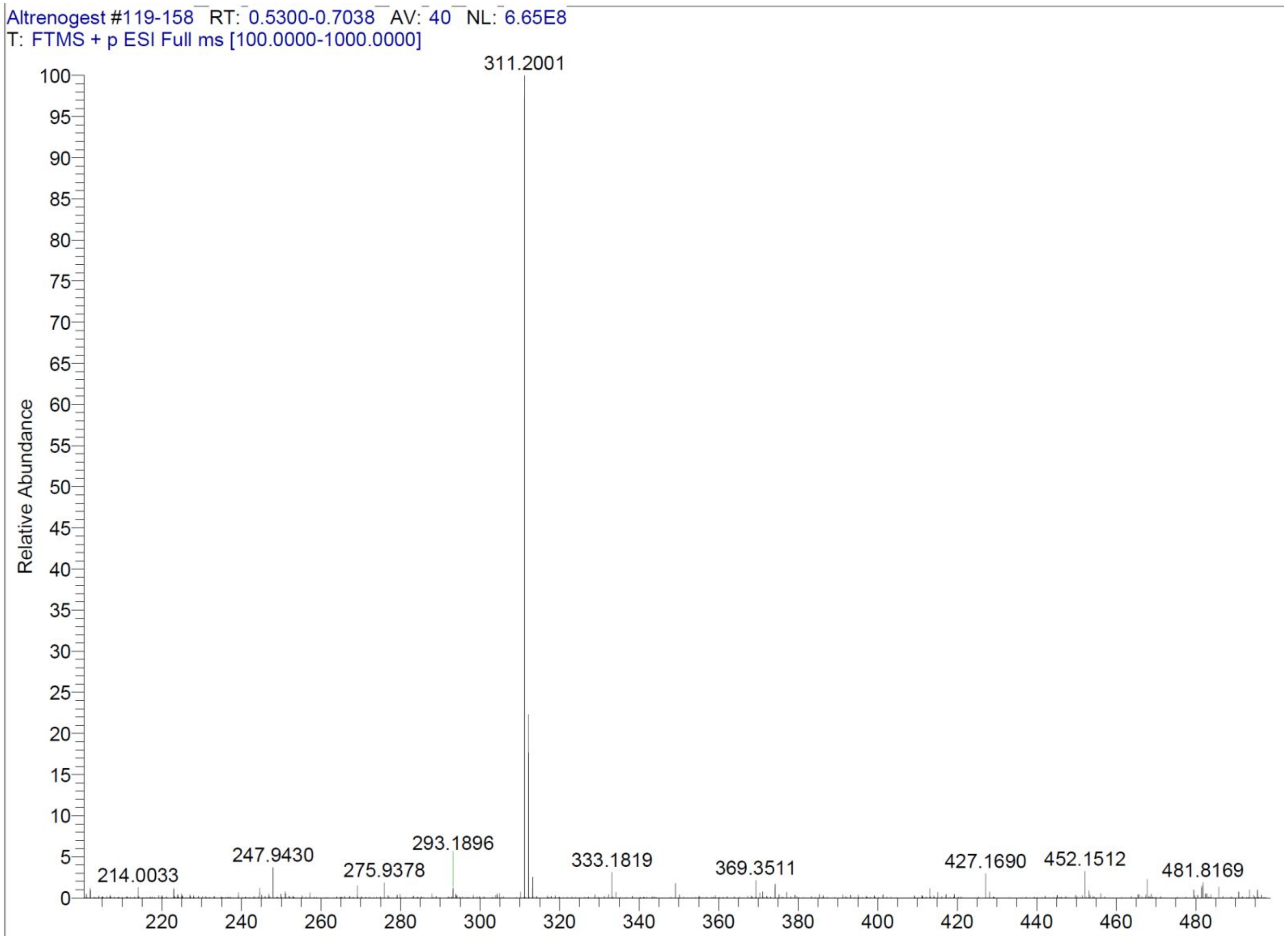

Figure S28. HRESIMS data for altrenogest gave an $(M+H)^+$ ion at *m*/*z* 311.2001.

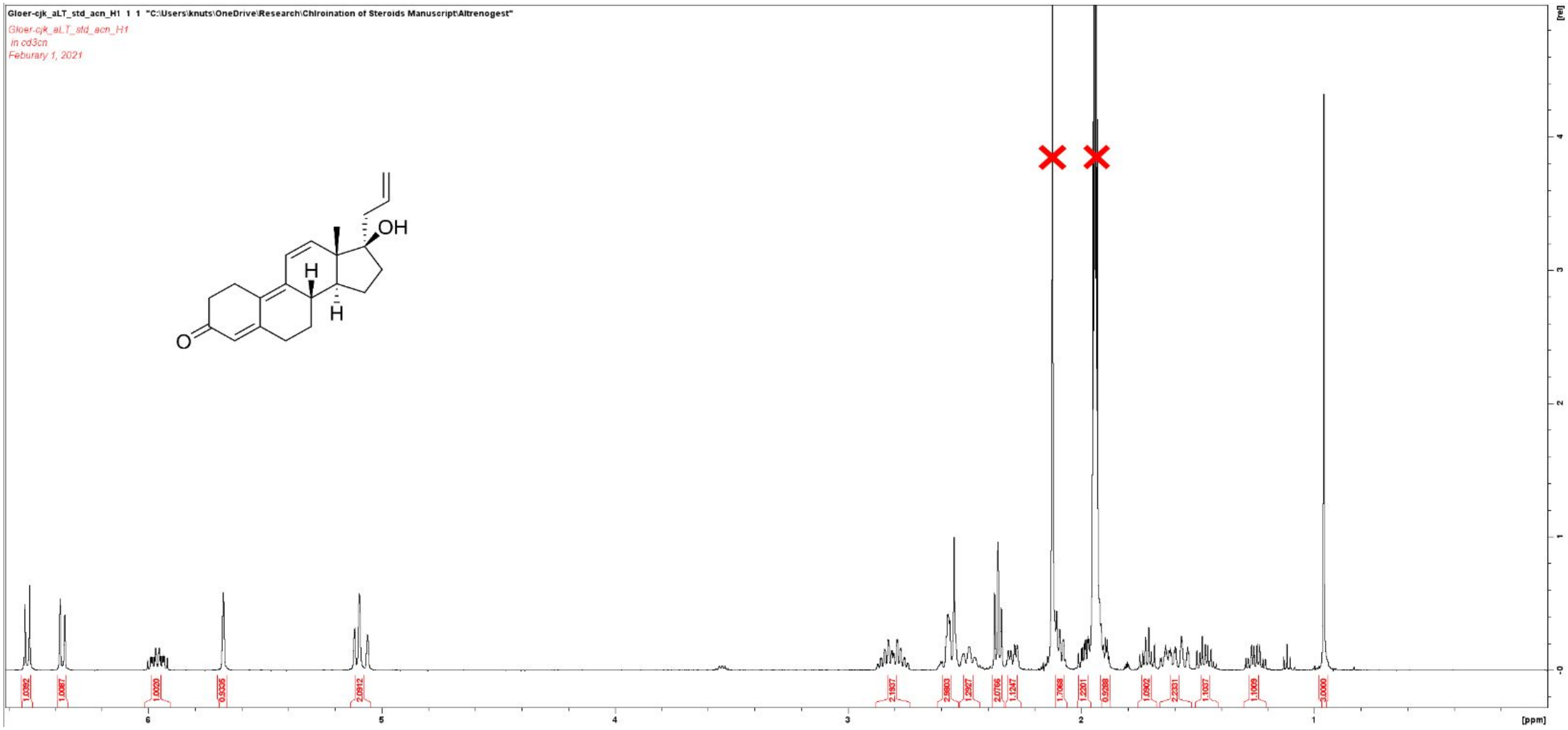

Figure S29. ^{1}H NMR spectrum of altrenogest in CD_3CN (500 MHz).

Proton	δ_H mult. (*J* in Hz)[a]
1	2.35 t (7.5)
	2.35 t (7.5)
2	2.77 m
	2.84 m
4	5.67 s
6	2.57 m
	2.54 m
7	1.89 m
	1.25 dt (5.1,12)
8	2.48 br t (7.3)
11	6.52 d (10)
12	6.37 d (10)
14	1.71 dt (6.8,12)
15	1.98 m
	1.57 m
16	1.63 m
	1.47 dq (6.7,12)
18	0.95 *s*
20	2.29 ddd (14,6.3,1.1)
	2.09 m
21	5.96 dddd (17,10,8.0,6.3)
22	5.10 dd (10,1.4)
	5.07 dd (17,1.4)

Table S6. ^{1}H NMR data for altrenogest in CD_3CN (500 MHz).

[a]Assignments made in reference to Wammer et.al.[10]

Product 5 (4-chloro-altrenogest) Data

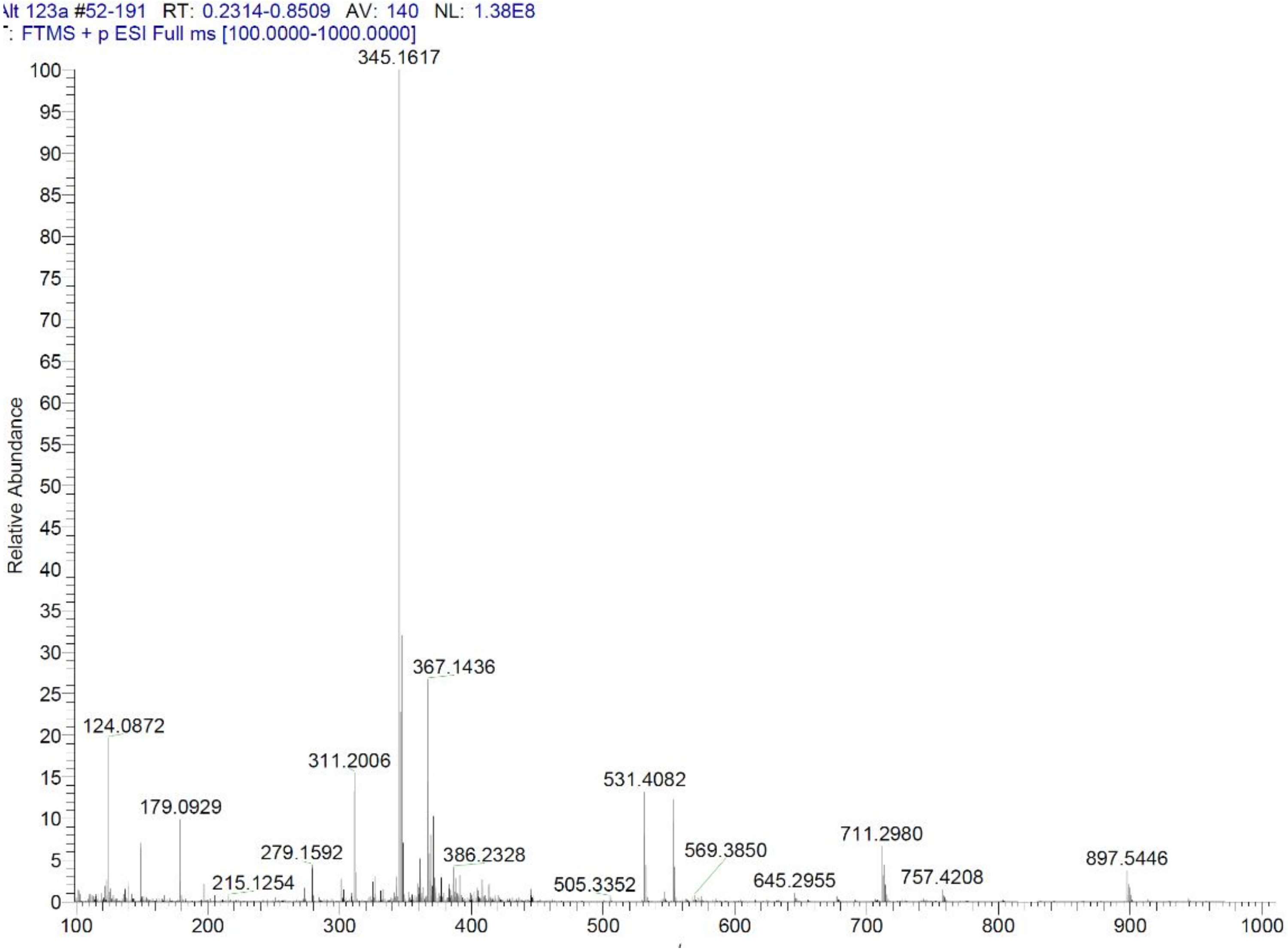

Figure S30. HRESIMS data for product **5** (4-chloro-altrenogest) yielded an $(M+H)^+$ ion at *m*/*z* 345.1617.

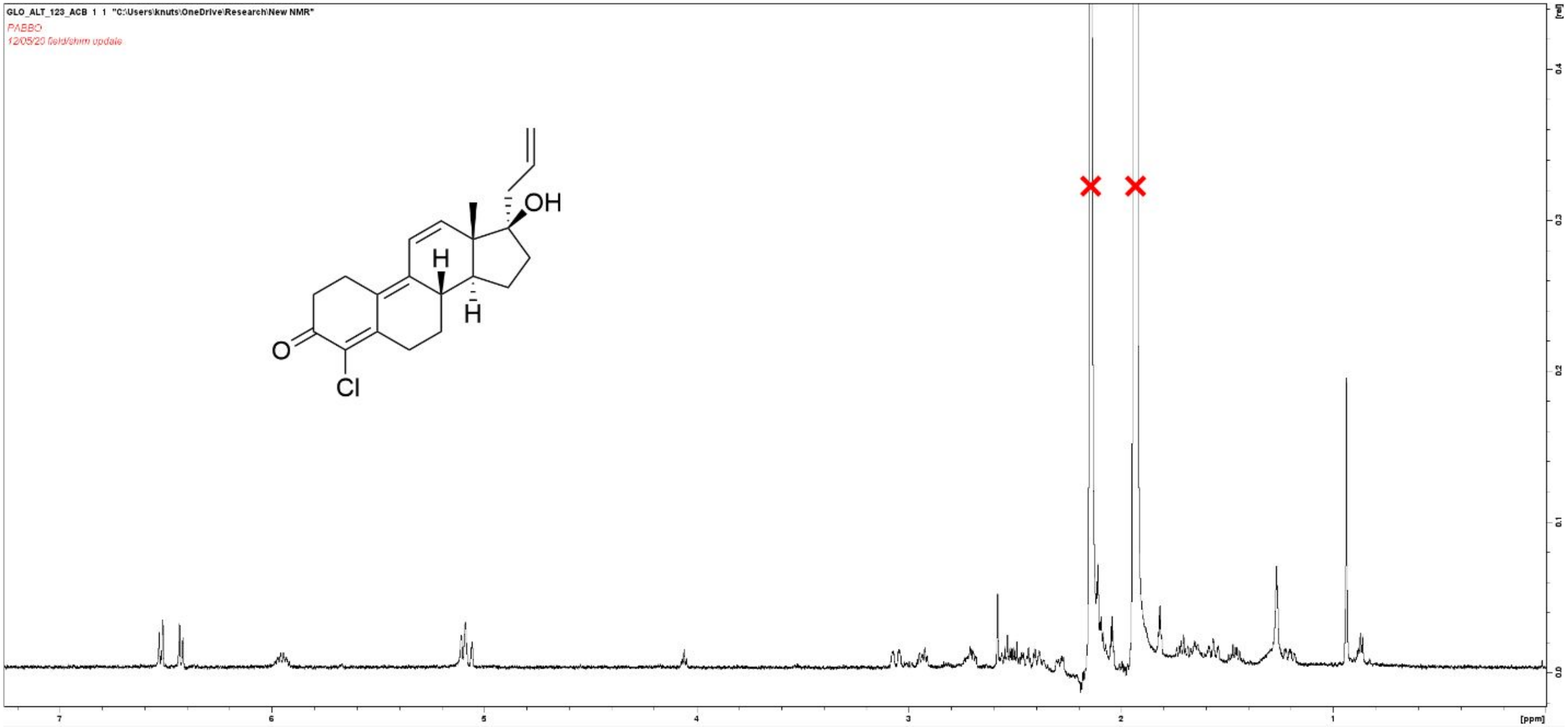

Figure S31. ^{1}H NMR spectrum of product **5** (4-chloro-altrenogest) in CD_3CN (600 MHz).

Product 6 (11,12-epoxy-altrenogest) Data

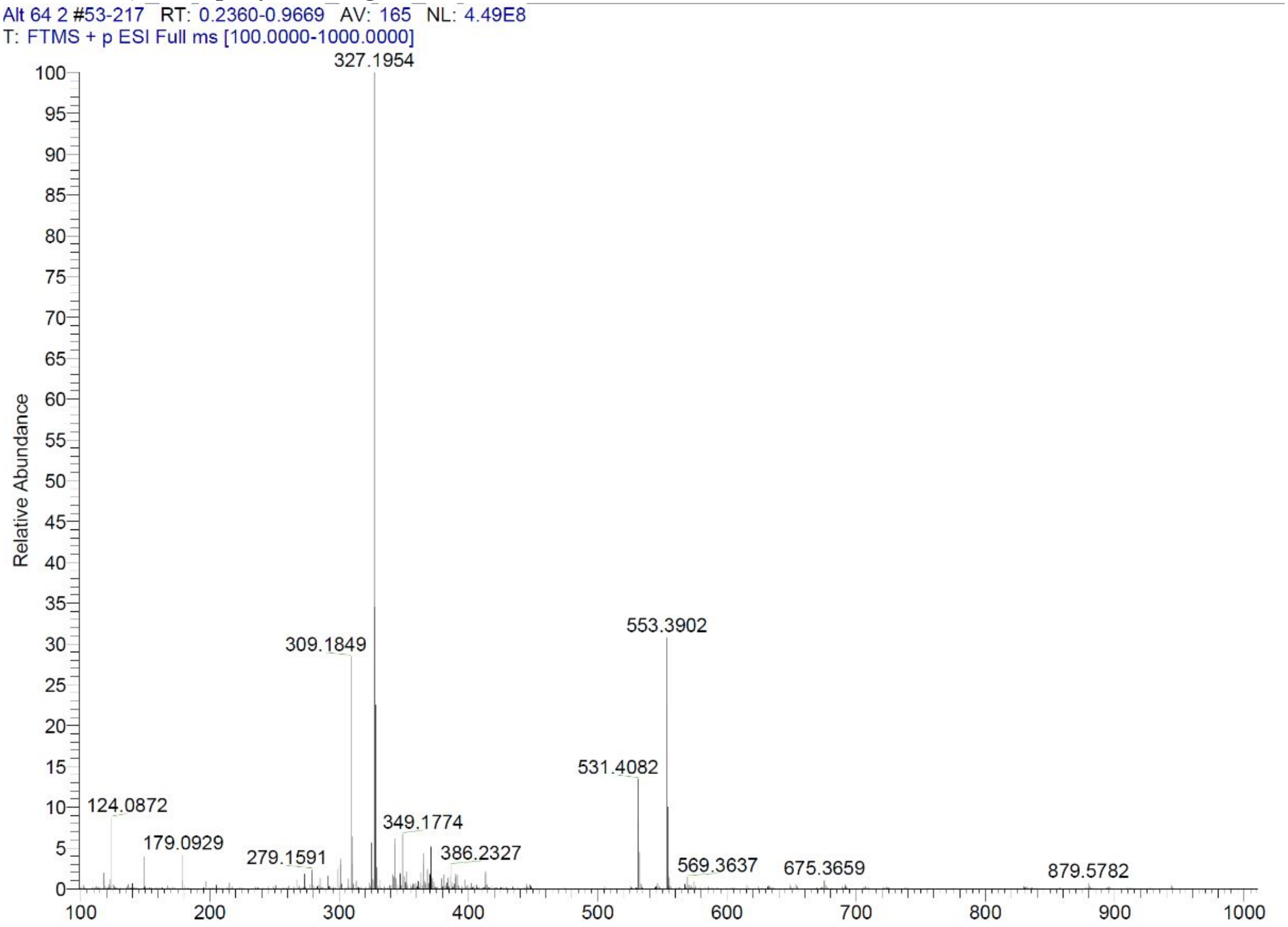

Figure S32. HRESIMS data for product **6** (11,12-epoxy-altrenogest) yielded an $(M+H)^+$ ion at *m/z* 327.1954.

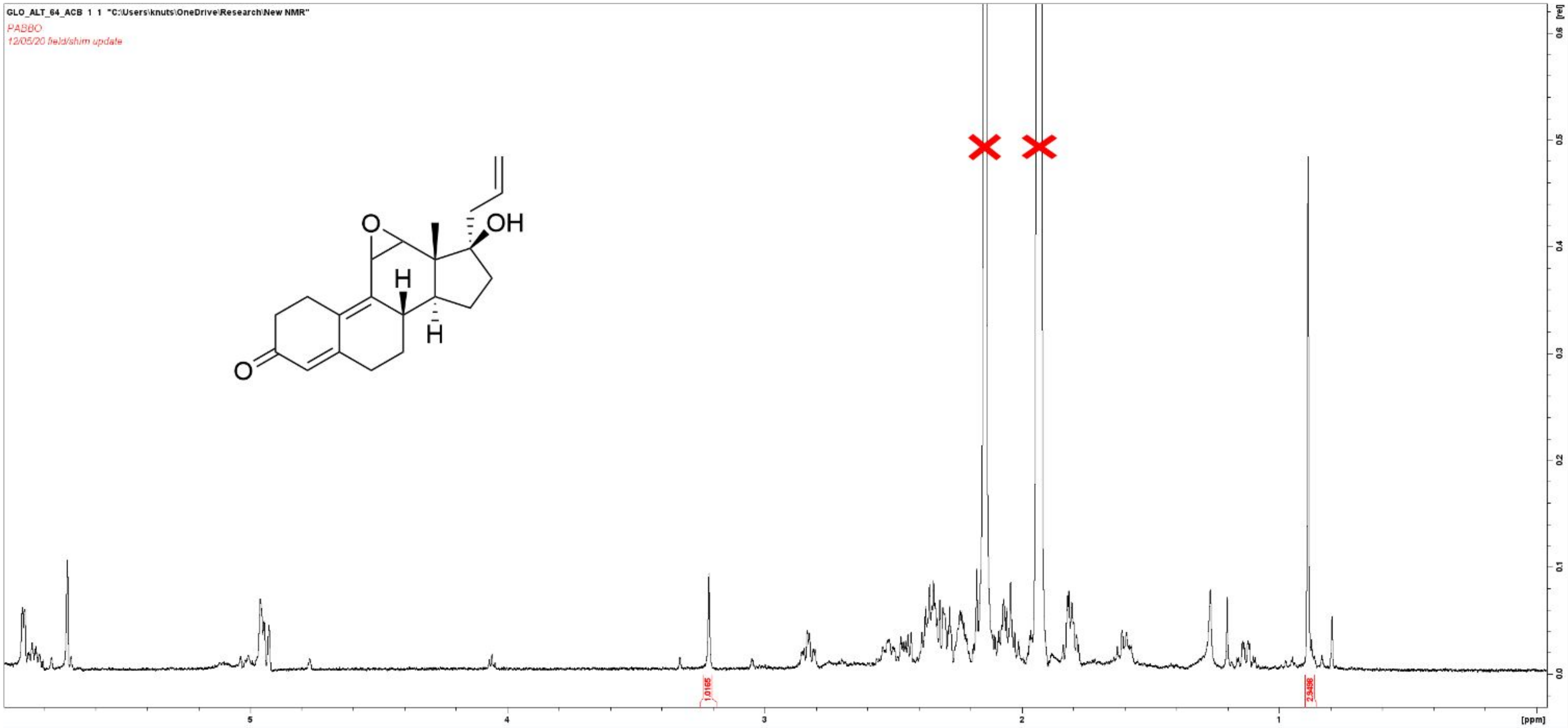

Figure S33. ^{1}H NMR spectrum of product **6** (11,12-epoxy-altrenogest) in CD_3CN (600 MHz).

Dienedione Data

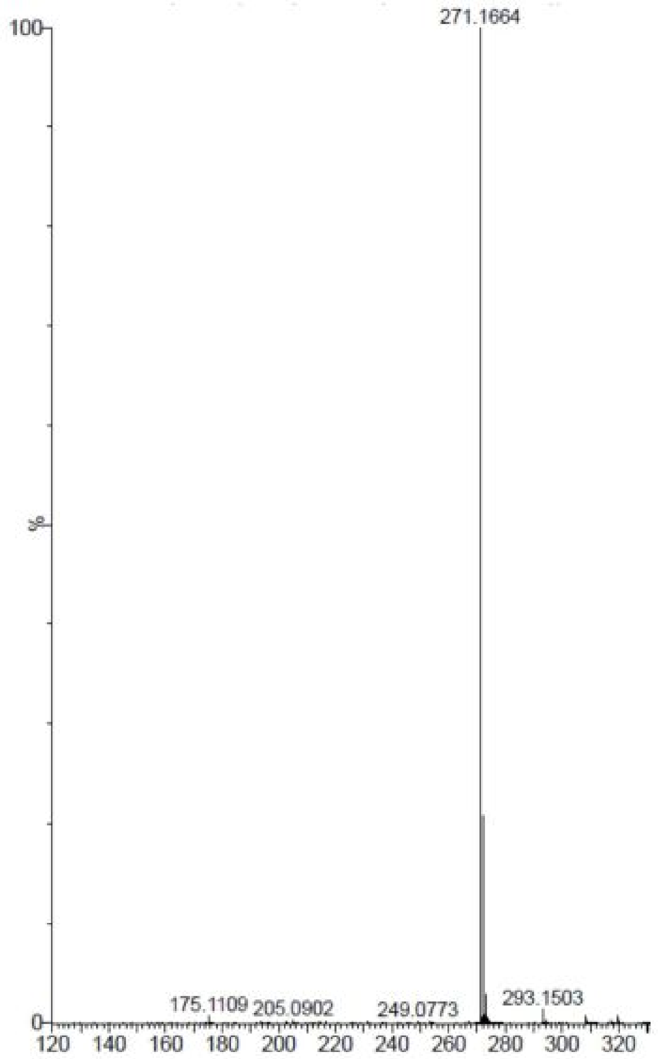

Figure S34. HRESIMS data of dienedione standard yielded an $(M+H)^+$ ion at *m*/*z* 271.1664.

Product 7 (9,10-epoxy-dienedione) data

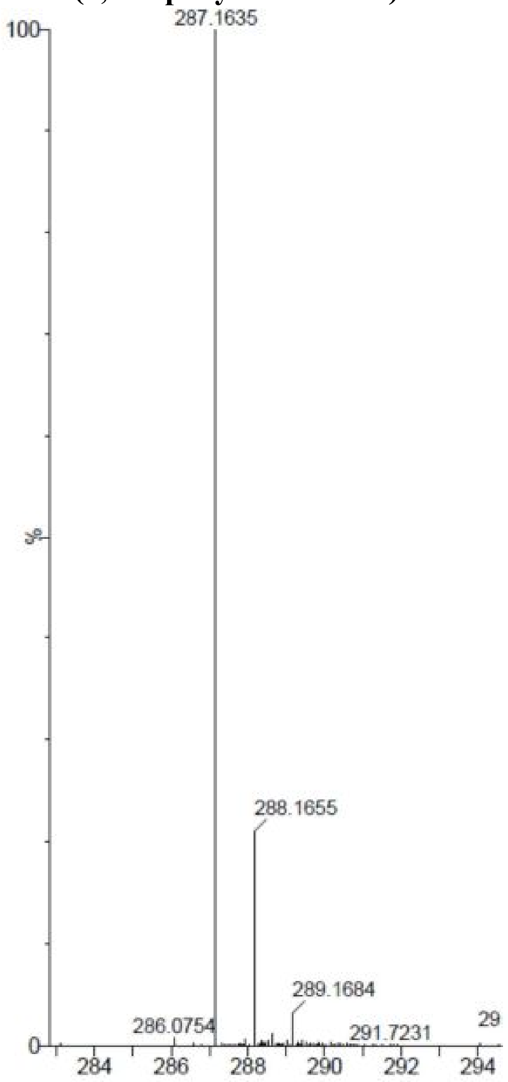

Figure S35. HRESIMS data of product **7** (9,10-epoxy-dienedione) yielded an $(M+H)^+$ ion at *m/z* 287.1635.

Methyldienolone Data

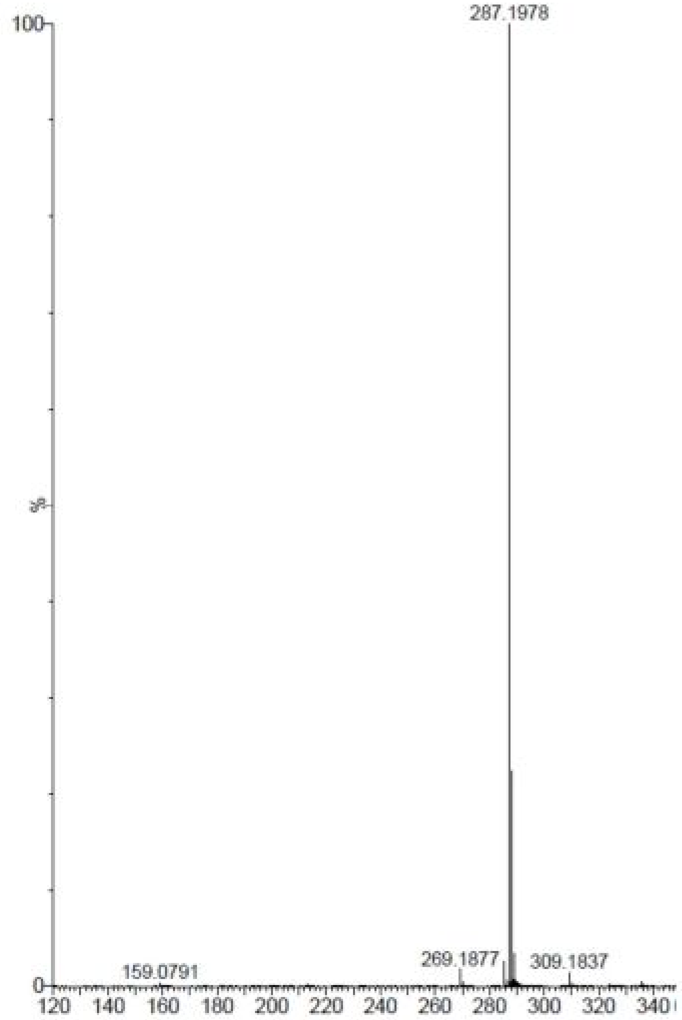

Figure S36. HRESIMS data of methyldioenolone standard yielded an $(M+H)^+$ ion at *m*/*z* 287.1978.

Product 8 (9,10-epoxy-methyldienolone) Data

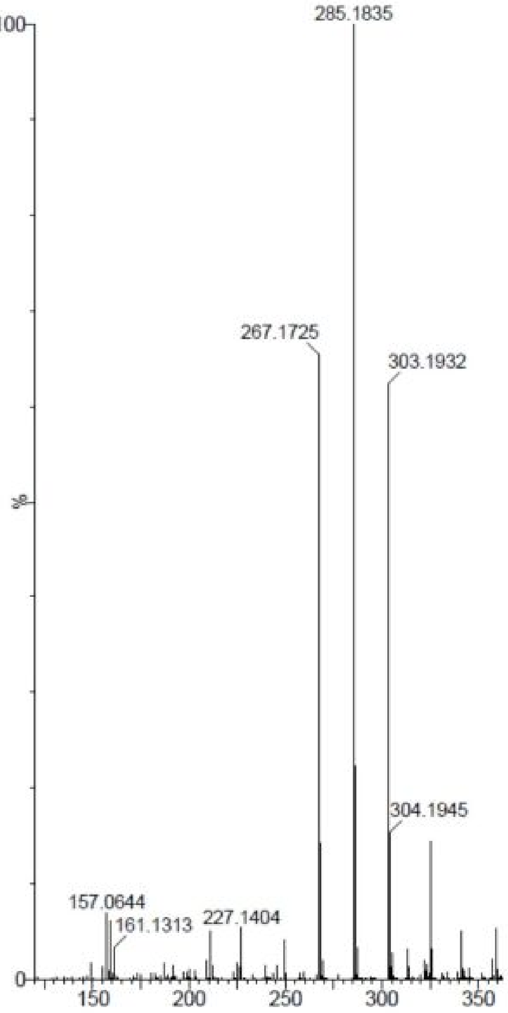

Figure S37. HRESIMS data of product **8** (9,10-epoxy-methyldienolone) yielded an $(M+H)^+$ ion at *m*/*z* 303.1932.

Methyl Trenbolone Data

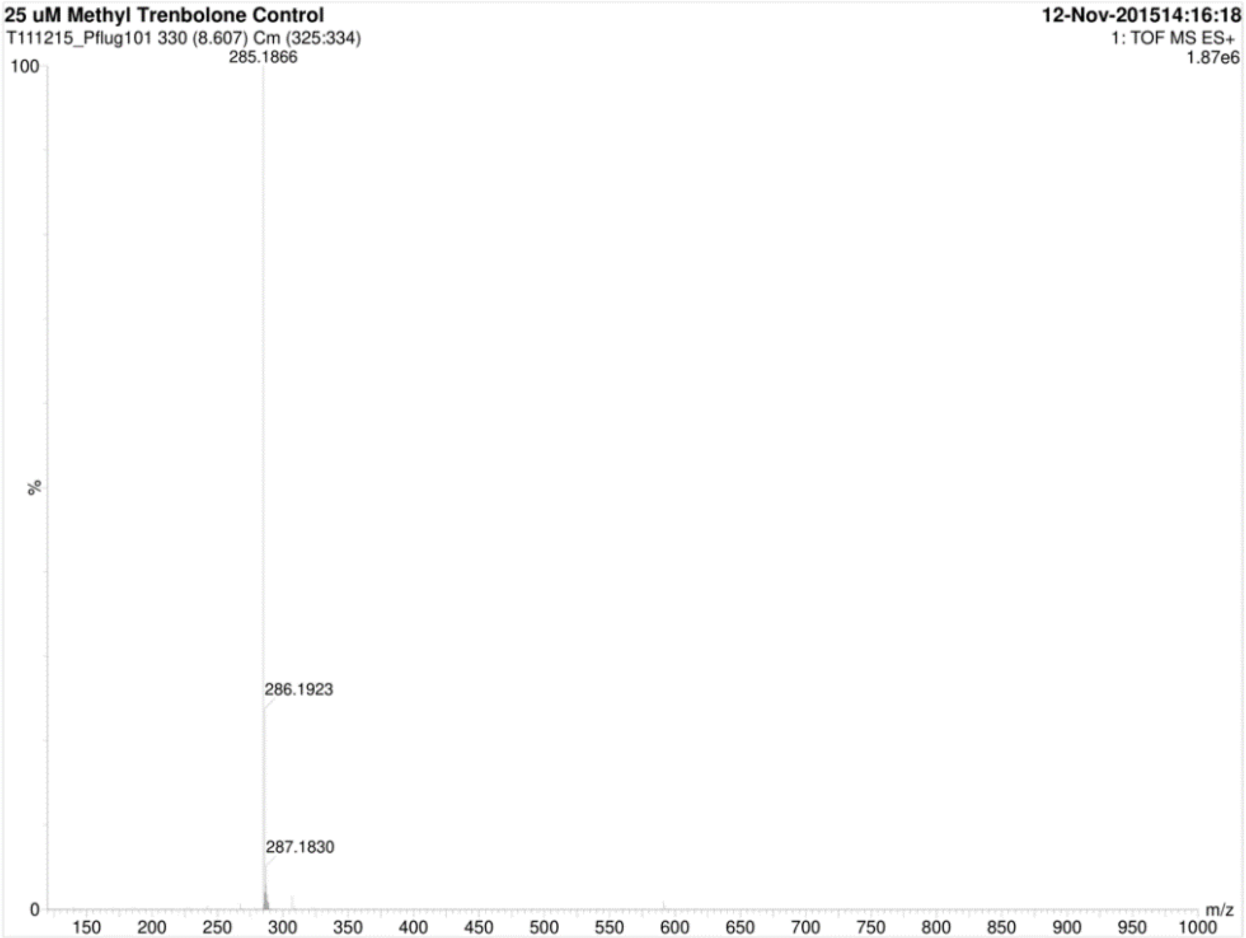

Figure S38. HRESIMS of methyl trenbolone standard yielded an $(M+H)^+$ ion at *m/z* 285.1866.

Product 9 (4-chloro-methyl-trenbolone) data

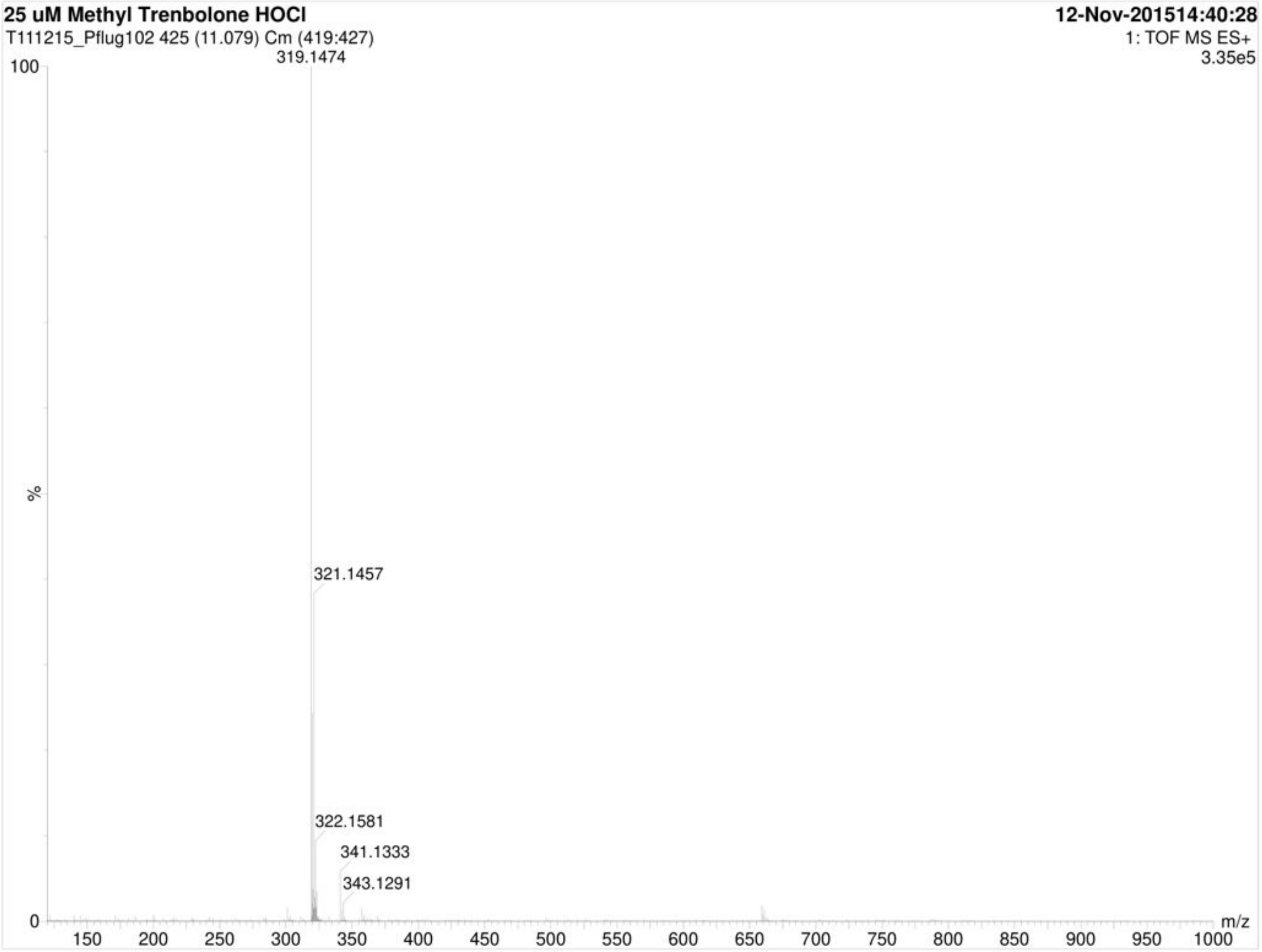

Figure S39. HRESIMS of product **9** (4-chloro-methyl-trenbolone) yielded an $(M+H)^+$ ion at *m/z* 319.1474.

17α-trenbolone data

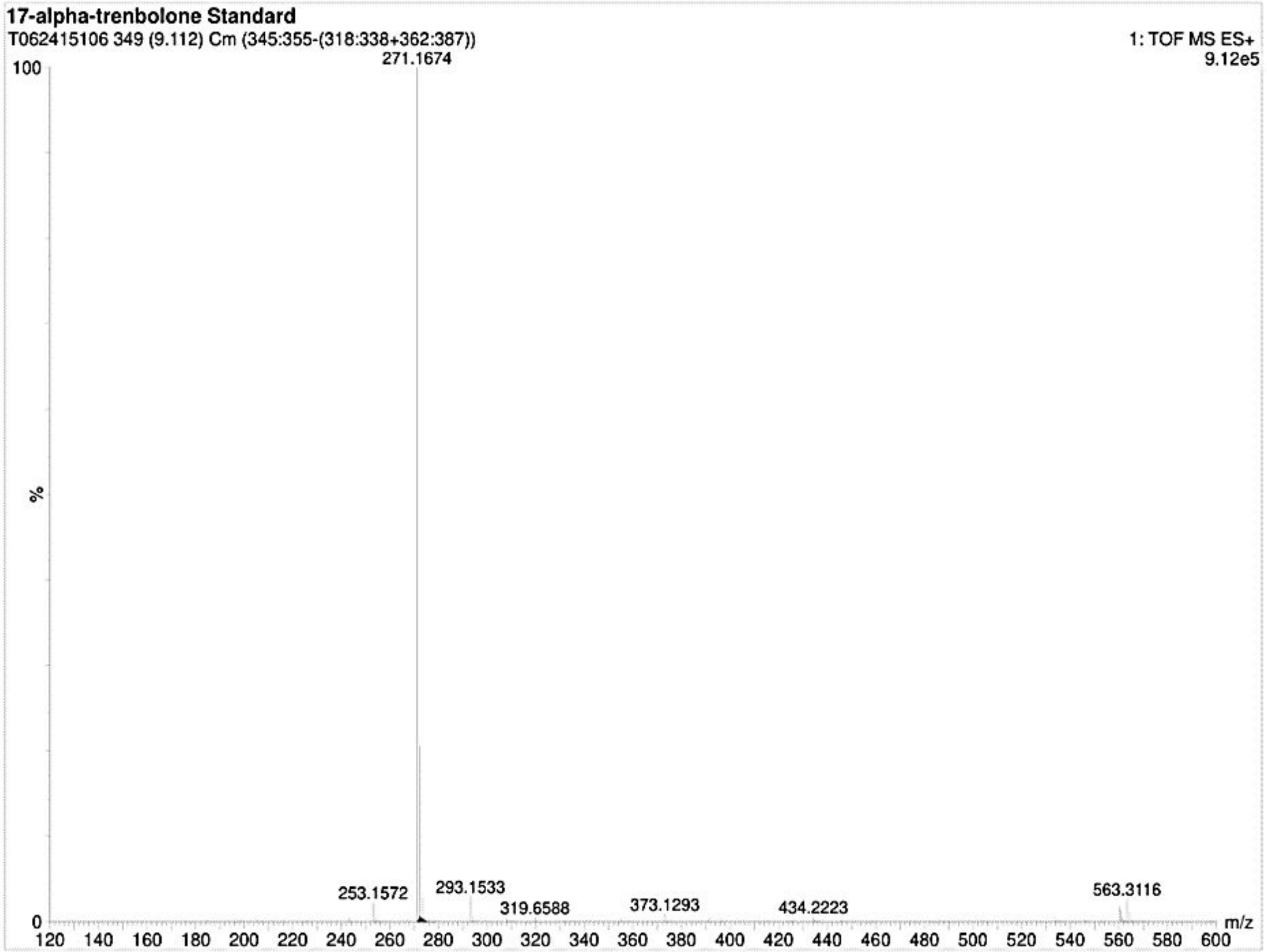

Figure S40. HRESIMS of 17α -trenbolone standard yielded an $(M+H)^+$ ion at *m*/*z* 271.1674.

Product 10 (4-chloro-17α-trenbolone) data

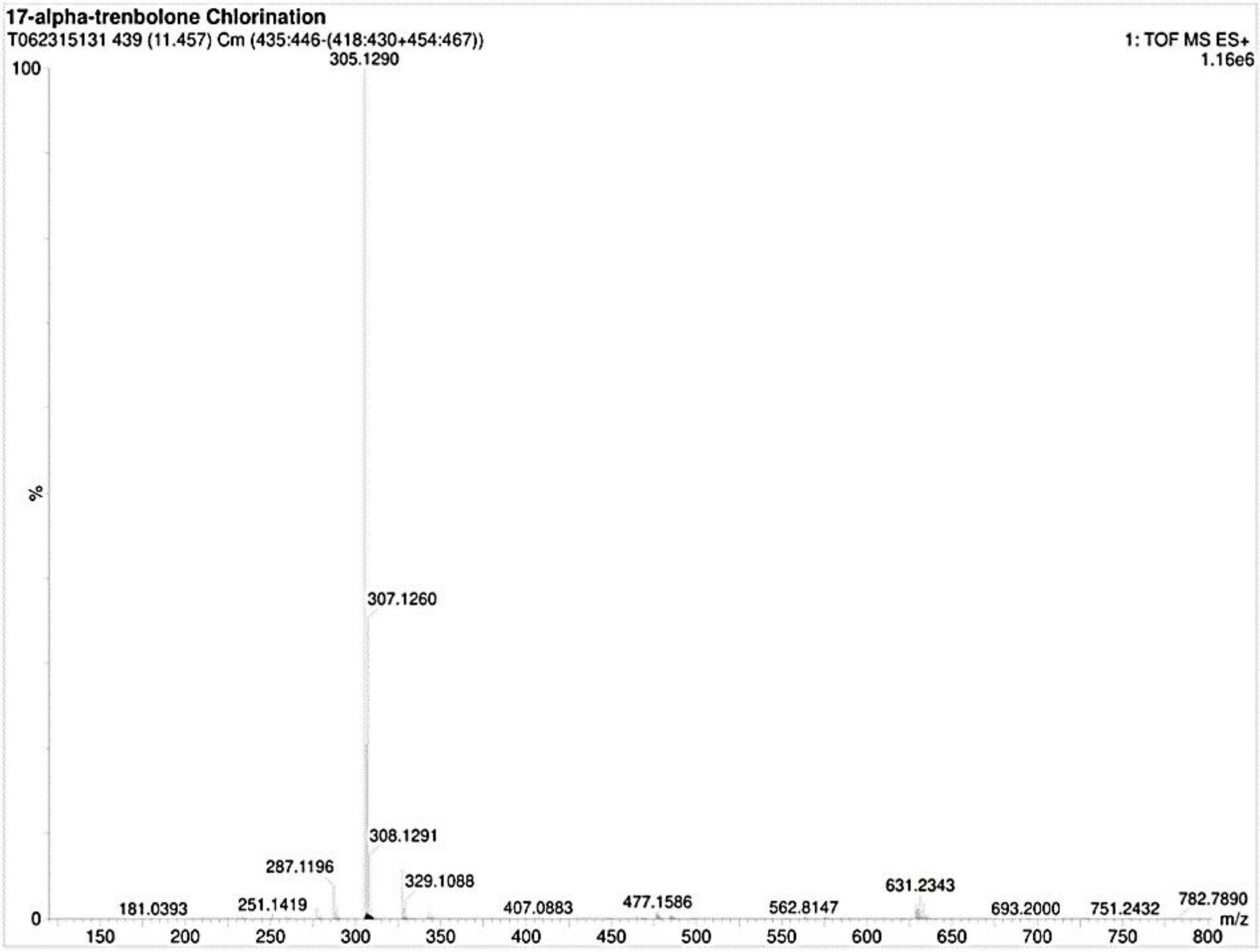

Figure S41. HRESIMS of product **10** (4-chloro-17α -trenbolone) yielded an $(M+H)^+$ ion at *m*/*z* 305.1290.

Gestrinone Data

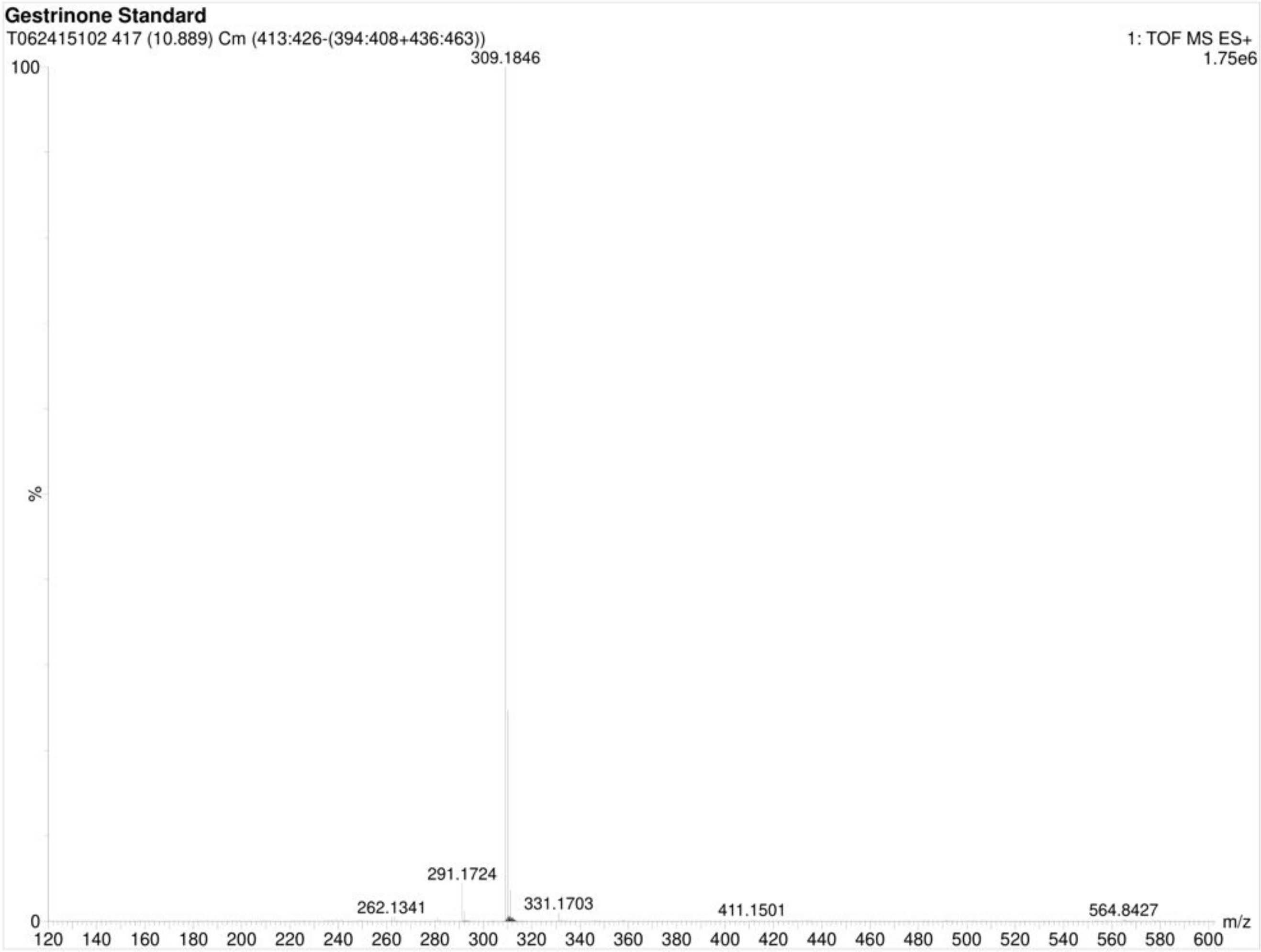

Figure S42. HRESIMS of gestrinone standard yielded an $(M+H)^+$ ion at *m*/*z* 309.1846.

Product 11 (4-chloro-gestrinone) data

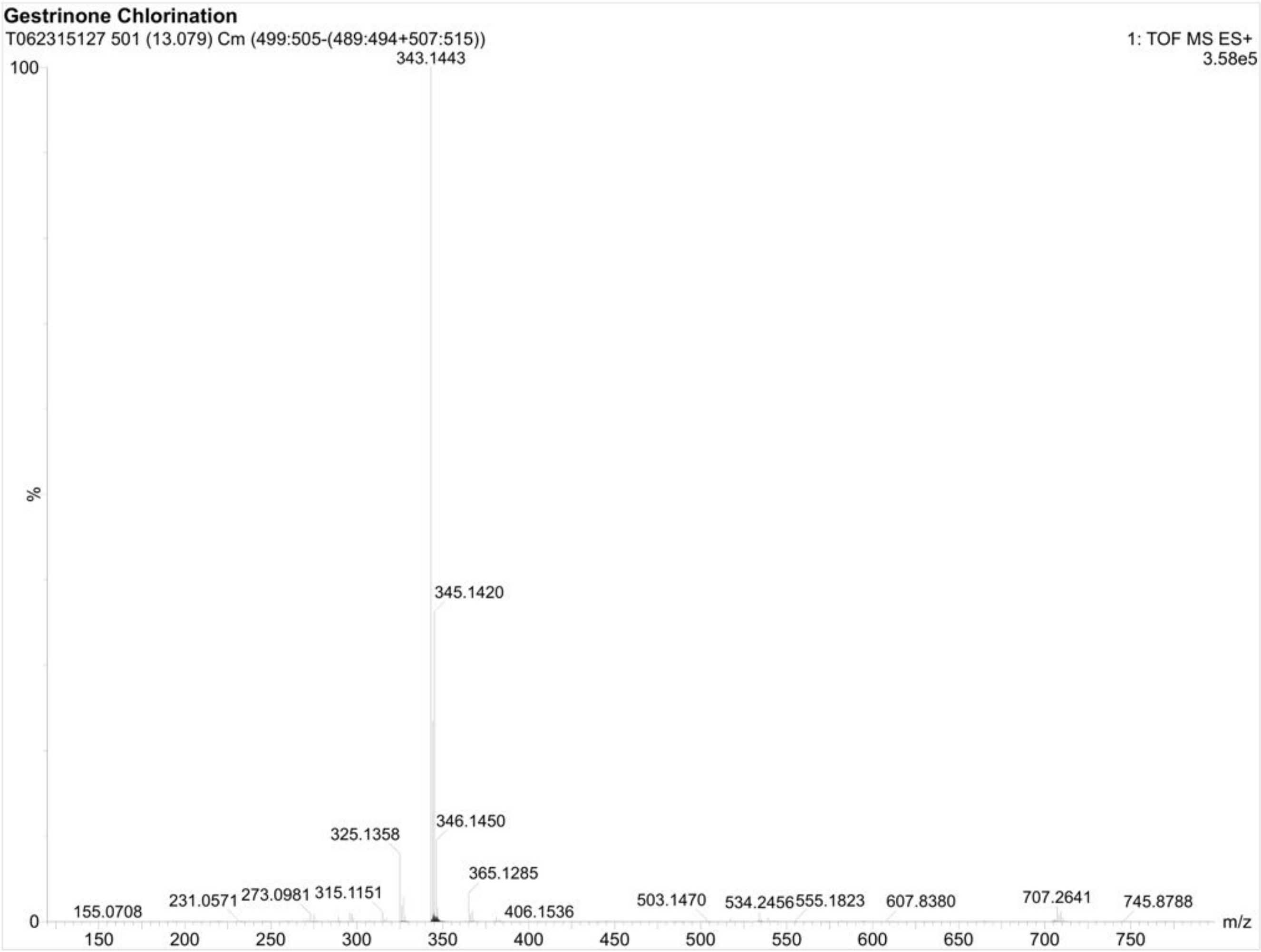

Figure S43. HRESIMS of product **11** (4-chloro-gestrinone) yielded an $(M+H)^+$ ion at *m/z* 343.1443.

Computational Data

Species	enone	dienone	trienone
E4/D4/T5	0	0	0
D9/T10		10	23
T10			9
T12			3

Table S7. Relative free energies (kcal mol-1) for bare cationic intermediates (MN15/6-31+G(d,p) + SMD water)

Species	Testosterone	Dienogest	β-trenbolone
reactant	0	0	0
TS for chlorination	18	14	9
intermediate	13	-6	-14

Table S8. Relative free energies (kcal mol^{-1}) for initial chlorination at C4 via HOCl (MN15/6-31+G(d,p) + SMD water).

Species	testosterone	dienogest		beta-trenbolone	
With hydroxide	chlorohydrin pathway	deprotonation pathway	chlorohydrin pathway	deprotonation pathway	chlorohydrin pathway
TS for deprotonation / chlorohydrin formation[1]	2	-1	7	4	9
4-Cl or chlorohydrin product	-57	-40	-33	-33	-4
TS for epoxide formation	-35	N/A	-21	N/A	ND
epoxide product	-56	N/A	-38	N/A	ND
With water					
TS for deprotonation / chlorohydrin formation[1]	5	9	4	9	8
4-Cl or chlorohydrin product	-31	-10	-5	-9	-5
TS for epoxide formation	ND	N/A	ND	N/A	ND
epoxide product	ND	N/A	ND	N/A	ND

Table S9. Relative free energies (kcal/mol) for deprotonation and chlorohydrin formation, with either hydroxide or water. Energies are relative to common cationic intermediate. (MN15/6-31+G(d,p) + SMD water). [1]C4-C9 chlorohydrin for dienogest, C4-C12 chlorohydrin for beta-trenbolone.

**Species**	**dienogest**		**beta-trenbolone**	
	forward barrier	*reverse barrier*	*forward barrier*	*reverse barrier*
With hydroxide				
4-Cl product	-1	+38	+4	+37
chlorohydrin product	+7	+40	+9	+13
With water				
4-Cl product	+9	+19	+9	+18
chlorohydrin product	+4	+9	+8	+13

Table S10. Forward and reverse free energies of activation (kcal/mol) for 4-Cl and chlorohydrin formation from the common intermediate, using either hydroxide or water. (MN15/6-31+G(d,p) + SMD water).

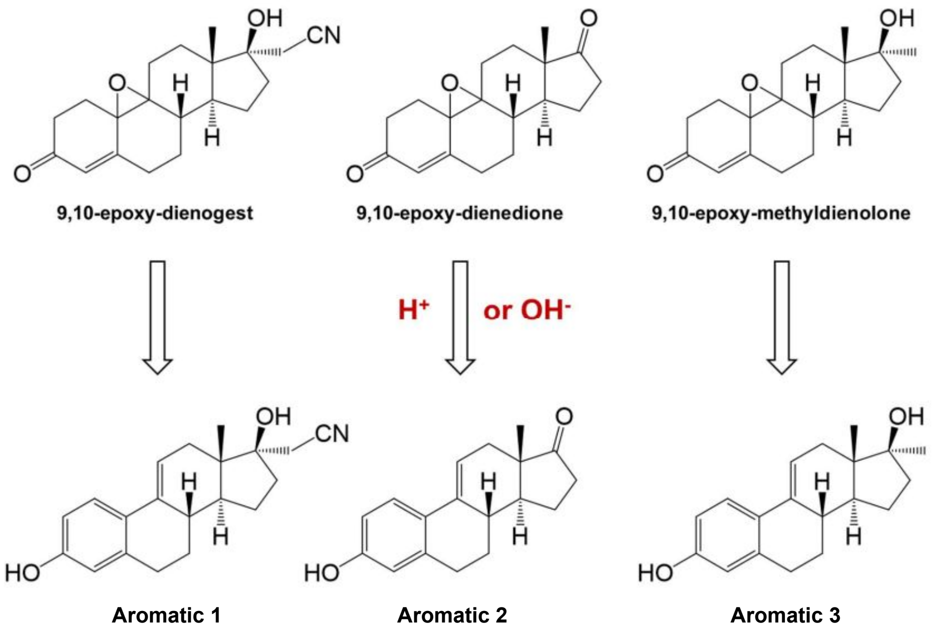

Figure S44. Reaction of dienones with aqueous chlorine results in exclusive 9,10-epoxidation. Subsequent treatment of epoxy products with aqueous acid or base results in epoxide ring opening and aromatization to form known estrogenic products.

Coordinates, Electronic Energies and Free Energies for Bare Cationic Intermediates; MN15/6-31+G(d,p) + SMD water

E4

SCF Done: E(RMN15) = -1196.508098

Sum of electronic and thermal Free Energies= -1196.175774

C,0,-0.0191029256,-0.0275724902,0.0351648336

C,0,-0.0061362616,-0.0158982593,1.4902337642

C,0,1.2274458697,-0.0048239639,2.2784727993

C,0,2.5418690042,-0.2599368877,1.5205269197

C,0,2.3198863238,-0.8916562157,0.1412177325

C,0,1.3432808596,-0.0193940077,-0.6430154785

H,0,1.7328344262,1.0070345712,-0.691130347

H,0,1.2236922144,-0.3756753516,-1.671161499

H,0,1.8737933172,-1.8955645205,0.2731920792

C,0,3.6757325785,-1.0563240319,-0.5301421233

H,0,4.1327587369,-0.052817159,-0.6254647653

C,0,4.6190112029,-1.9083751345,0.3215811832

C,0,4.8486682741,-1.286524225,1.6923674557

C,0,3.5000447762,-1.080464072,2.3909457914

H,0,3.0375872985,-2.059537809,2.5911799783

H,0,3.6316955331,-0.580961993,3.3605133121

H,0,5.5053766316,-1.9121374015,2.3098607999

H,0,5.3504617745,-0.3142946659,1.5624650402

C,0,5.8190902913,-2.0780617743,-0.6082987448

C,0,5.185602355,-2.2687183154,-2.0097947131

C,0,3.7226715378,-1.7603864322,-1.8884641287

H,0,3.4300030367,-1.1107998234,-2.7204397295

H,0,3.0200903659,-2.6062786231,-1.8718243308

H,0,5.7408102896,-1.7137054539,-2.773212566

H,0,5.2046325235,-3.3224334354,-2.3081904673

H,0,6.4166584718,-1.1552801623,-0.5792603574

H,0,6.4777091562,-2.9050198621,-0.3207001828

H,0,4.1306942752,-2.8911126214,0.4578854988

H,0,3.0105476593,0.7215610789,1.3334334846

C,0,1.2223623882,1.436102719,2.9151637142

C,0,0.0393142567,1.6501101864,3.8437536881

C,0,-1.2540556092,1.3936944584,3.1294435895

C,0,-1.2550395809,0.0593659786,2.270867704

Cl,0,-2.751126016,-0.1114091597,1.3569876429

H,0,-1.2266664956,-0.7250158141,3.0422464893

O,0,-2.2549302398,2.0622701969,3.1830484667

H,0,0.0853930587,0.9405749058,4.6820113595

H,0,0.0093152247,2.6671559622,4.2407248816

H,0,1.2480249565,2.1884927879,2.1182055565

H,0,2.1638891946,1.4975441615,3.4697848746

H,0,1.1055056261,-0.6980143141,3.1261785049

H,0,-0.5645787737,-0.970172799,-0.1893018403

H,0,-0.7278321523,0.7369520411,-0.3235463438

D4

SCF Done: E(RMN15) = -1195.31489439

Sum of electronic and thermal Free Energies=   -1195.004553

C,0,-0.0082420506,-0.1917236377,0.0403782649

C,0,0.0064266716,0.0118857344,1.515632607

C,0,1.1698943909,-0.0242697204,2.2627799593

C,0,2.4166066155,-0.0907064377,1.5745406594

C,0,2.4787614562,-0.2615910641,0.0934297069

C,0,1.2609423115,0.318940652,-0.6149102622

H,0,1.3000532942,1.4152860325,-0.5598928572

H,0,1.2835194926,0.0337933725,-1.6707415991

H,0,2.4333376419,-1.3716595523,-0.0050297026

C,0,3.8045394028,0.1893460939,-0.5082451316

H,0,3.845676518,1.2915893247,-0.4678030134

C,0,4.9994058681,-0.3444371955,0.2719591977

C,0,4.9806420118,0.2342003349,1.6731022062

C,0,3.6640606065,-0.1099220676,2.3772543911

H,0,3.6941103127,-1.1597818205,2.7376875467

H,0,3.5271856213,0.4815441106,3.2892029127

H,0,5.8096437046,-0.1315917348,2.2879103557

H,0,5.0809876393,1.3264206346,1.605163047

C,0,6.1634778818,0.0280455152,-0.6430968133

C,0,5.6257713962,-0.2623989028,-2.0651165212

C,0,4.077190705,-0.2808877031,-1.9405005917

H,0,3.5832297272,0.3408630832,-2.6940896848

H,0,3.6903998365,-1.3035652742,-2.0557959885

H,0,5.9658216339,0.4928411963,-2.7806557092

H,0,5.9899503685,-1.2299828099,-2.4264371504

H,0,6.3697563971,1.1012994027,-0.5226904969

H,0,7.0867616032,-0.5144518577,-0.414437014

H,0,4.9082252628,-1.4440494167,0.3223609234

C,0,1.1374469701,-0.0218429699,3.7759335644

C,0,-0.2106270313,-0.5149533836,4.3148536783

C,0,-1.2997816606,0.2976526034,3.6990976811

C,0,-1.3411014399,0.1851730704,2.1784232846

Cl,0,-2.1986723605,1.5794328433,1.4590528929

H,0,-1.9376378781,-0.7102080643,1.9450025964

O,0,-2.0955635635,0.9832507139,4.318512764

H,0,-0.3545924634,-1.5631462484,4.0183998129

H,0,-0.2622737871,-0.436474313,5.4024218554

H,0,1.3607479692,0.9813739815,4.1599711715

H,0,1.9136555476,-0.6901750867,4.1579413882

H,0,-0.0804861056,-1.2870455175,-0.092923402

H,0,-0.9073922239,0.2386121639,-0.4052197178

D9

SCF Done: E(RMN15) = -1195.30144475

Sum of electronic and thermal Free Energies= -1194.989174

C,0,-0.0194801121,-0.031358263,0.0143830156

C,0,-0.0109491147,0.0143847721,1.5185242708

C,0,1.2117636239,0.0034860232,2.2703432423

C,0,2.5053189512,0.3635457574,1.5711952422

C,0,2.483145101,0.071287,0.0599140812

C,0,1.2200799135,0.5917261485,-0.6157036918

H,0,1.1851661186,1.6868798797,-0.5532713527

H,0,1.2516405842,0.3300197784,-1.6780523146

H,0,2.482578939,-1.0325986988,-0.001240189

C,0,3.7721710391,0.5711993753,-0.582128764

H,0,3.8237137557,1.6683835396,-0.4652484358

C,0,5.0082789698,-0.0268466513,0.0848628041

C,0,5.0561553835,0.3457300934,1.5562827475

C,0,3.7798064832,-0.134836307,2.2546637243

H,0,3.7331917997,-1.2331967379,2.2174567446

H,0,3.7912054348,0.1614491148,3.3075306414

H,0,5.9188668261,-0.1008343525,2.0634937754

H,0,5.151552178,1.4377120007,1.6541723275

C,0,6.1277042634,0.4630763516,-0.8306610325

C,0,5.523090999,0.3426230403,-2.2529442946

C,0,3.9869552386,0.2205702551,-2.056802173

H,0,3.4206123257,0.8629714465,-2.738852856

H,0,3.6550319434,-0.8138189458,-2.2267484764

H,0,5.7789752121,1.2135233316,-2.8649833748

H,0,5.9155256883,-0.5375627829,-2.7729124204

H,0,6.3421436638,1.5143894606,-0.5910714424

H,0,7.060189519,-0.099063227,-0.7129482872

H,0,4.926723352,-1.1254345476,-0.0021765018

Cl,0,2.351251213,2.182692896,1.8710234307

C,0,1.25097238,-0.3073560295,3.7055381931

C,0,-0.0797716663,-0.5804467652,4.3941449568

C,0,-1.2199373523,0.1077997474,3.724503984

C,0,-1.1661427472,0.1449267495,2.2395933273

H,0,-2.1110273866,0.3249792714,1.7279928455

O,0,-2.1996507577,0.5632654579,4.3050503185

H,0,-0.3138676961,-1.6532080772,4.3252820898

H,0,-0.0438826871,-0.3174958324,5.4529851414

H,0,1.7980926282,0.5053836223,4.2088363853

H,0,1.9322573285,-1.1697744071,3.7993340142

H,0,-0.0592068885,-1.09515847,-0.2597852908

H,0,-0.9423952872,0.4271097755,-0.3513497654

T4

SCF Done: E(RMN15) = -1194.10610791

Sum of electronic and thermal Free Energies= -1193.818787

C,0,0.0121444468,-0.0810473344,0.0081692225

C,0,0.0102943506,-0.0058658317,1.5007440044

C,0,1.1596395647,-0.0176203905,2.2552706199

C,0,2.4298536949,0.0235762988,1.5777054804

C,0,2.4999043556,-0.0701354171,0.0781093661

C,0,1.2672728187,0.5303599499,-0.5852772619

H,0,1.2667239162,1.6178270963,-0.4273766277

H,0,1.3092895961,0.3474635457,-1.6633810278

H,0,2.5113931349,-1.162696994,-0.1284905383

C,0,3.8077146253,0.5118370965,-0.4324403799

H,0,3.8282970855,1.5871507746,-0.1849675852

C,0,4.9877898491,-0.1441518126,0.271194732

C,0,4.8623036151,0.0122265537,1.7323133017

C,0,3.6312364346,0.1142758174,2.3335804791

H,0,3.5817490894,0.2182530888,3.4115979426

H,0,5.7547036235,0.0148314632,2.3578330211

C,0,6.2005455771,0.4263072646,-0.4684663045

C,0,5.7074830164,0.5024800472,-1.9388091871

C,0,4.1638100246,0.3219768785,-1.9041507438

H,0,3.641615952,1.0209294513,-2.5643812913

H,0,3.8861214459,-0.6967570434,-2.2085567518

H,0,5.9817144391,1.4624480413,-2.3863054

H,0,6.1711777309,-0.2797936312,-2.5475826491

H,0,6.4190872186,1.4282024322,-0.0803332051

H,0,7.0989731347,-0.1852721856,-0.3483307727

H,0,4.932376864,-1.2349459755,0.0742023854

C,0,1.1009043396,-0.1095107498,3.7651213691

C,0,-0.1903526336,-0.7913253888,4.2337731622

C,0,-1.3622026789,-0.0599396516,3.6711870842

C,0,-1.3536636034,0.0325280757,2.1486758191

Cl,0,-2.224796909,1.5062503573,1.610146688

H,0,-1.9333184579,-0.8239482239,1.7746021611

O,0,-2.2664803439,0.4184060468,4.336987159

H,0,-0.2113481893,-1.8191093261,3.8447233578

H,0,-0.2640080386,-0.8179648048,5.3228532026

H,0,1.1963342272,0.8861186399,4.2158285666

H,0,1.9374424268,-0.7082962436,4.1325342397

H,0,-0.0380988612,-1.1558143201,-0.2393450669

H,0,-0.8940941165,0.3742721272,-0.3993302957

T9

SCF Done: E(RMN15) = -1194.07075741

Sum of electronic and thermal Free Energies=   -1193.782840

C,0,-0.0284919173,-0.062601608,0.0191678014

C,0,-0.0130111012,0.0101553256,1.5255462995

C,0,1.2059330327,-0.0180876239,2.2856621042

C,0,2.5032095179,0.2607309792,1.5640729771

C,0,2.4642839228,-0.1623391148,0.0876530284

C,0,1.2602296425,0.4079723093,-0.6480056048

H,0,1.3172362241,1.5021201218,-0.6830957944

H,0,1.2764212747,0.0501289973,-1.6823465079

H,0,2.3512561874,-1.2622078763,0.124110159

C,0,3.8215564154,0.1420620407,-0.5240314563

H,0,4.067097458,1.2011152103,-0.3294150413

C,0,4.890400915,-0.7130062931,0.144522072

C,0,4.8623431833,-0.5397977911,1.6273783554

C,0,3.7705172577,-0.1014512965,2.2765794612

H,0,3.7985413172,0.0408605767,3.3536370953

H,0,5.7476032981,-0.7935997473,2.2095143294

C,0,6.1437727395,-0.332465159,-0.6463407873

C,0,5.6126791913,-0.2243845385,-2.102706738

C,0,4.0620450872,-0.1520927066,-2.0026073296

H,0,3.6281809277,0.6001108465,-2.6689389444

H,0,3.6104469623,-1.1217441073,-2.2551658801

H,0,6.0165269939,0.6641170759,-2.5980712107

H,0,5.9207098654,-1.0900107859,-2.6977496029

H,0,6.5128241252,0.6396214657,-0.2947155635

H,0,6.9566140863,-1.0575266328,-0.540988866

H,0,4.6571763722,-1.77039072,-0.0792476546

Cl,0,2.3055917735,2.1273908161,1.7231145748

C,0,1.2347177103,-0.2744042561,3.7322865981

C,0,-0.1019384331,-0.5201259524,4.4186928499

C,0,-1.2369100951,0.1484596062,3.7211287747

C,0,-1.1720752102,0.1544854225,2.2368182822

H,0,-2.1133317758,0.3240706403,1.7154347458

O,0,-2.2224886196,0.6148483333,4.2832129334

H,0,-0.3371486508,-1.5941846562,4.3815449412

H,0,-0.0735545037,-0.2243631456,5.4690582652

H,0,1.7730968414,0.5649457214,4.2031481375

H,0,1.9162235732,-1.1291535282,3.8721028011

H,0,-0.1820557963,-1.1231508279,-0.2255234293

H,0,-0.9038592819,0.4770637555,-0.3527432501

T10

SCF Done: E(RMN15) = -1194.09286365

Sum of electronic and thermal Free Energies=   -1193.805064

C,0,-0.0005556694,-0.0894057661,0.0072717464

C,0,-0.0103188103,-0.0048360992,1.5021000798

C,0,1.3348463912,-0.0179635033,2.2029433002

C,0,2.4337217134,0.7120264283,1.4315174729

C,0,2.3411301493,0.8314987163,-0.0590395738

C,0,0.9097597465,0.9837567877,-0.5761155691

H,0,0.5388446874,1.983599534,-0.3150904009

H,0,0.9321699376,0.9118264064,-1.667754943

H,0,2.738614788,-0.1415400464,-0.4269308569

C,0,3.2715216904,1.927693975,-0.5576992383

H,0,2.908321234,2.8941973248,-0.1696554484

C,0,4.677570073,1.7080806594,-0.0198920973

C,0,4.6762613057,1.6126762353,1.4373780353

C,0,3.5571625476,1.1605153975,2.1286308489

H,0,3.6167465448,1.0715705169,3.2066780943

H,0,5.579624486,1.8417637074,2.0030161673

C,0,5.5334673943,2.747616186,-0.7518204919

C,0,4.8397992906,2.8618627612,-2.1384697016

C,0,3.5250825468,2.042186066,-2.0569087049

H,0,2.7002203647,2.5128047535,-2.5995002191

H,0,3.6701001547,1.0338410433,-2.4687381598

H,0,4.6241097989,3.9100991558,-2.3656981537

H,0,5.4839274417,2.4838422168,-2.9376163063

H,0,5.4861462332,3.6998433818,-0.2129682036

H,0,6.5825558205,2.4495754444,-0.8197501722

H,0,4.9997610631,0.6902359223,-0.3427609512

C,0,1.2431793862,0.3928422346,3.670459272

C,0,0.0622241394,-0.254083162,4.383732264

C,0,-1.2269340587,0.0128965257,3.6666501871

C,0,-1.1616626315,0.0636216493,2.2007658294

H,0,-2.1118587532,0.1032295942,1.671591097

O,0,-2.3052879166,0.1236102072,4.2592988472

H,0,0.189321817,-1.346954108,4.4119328282

H,0,-0.0114500545,0.0961592581,5.4161968514

H,0,1.1388686239,1.485772546,3.6884361495

H,0,2.1641657877,0.1344864806,4.1974454493

Cl,0,1.9588092752,-1.7522089137,2.0946690933

H,0,0.3767704883,-1.0800775231,-0.2886783134

H,0,-1.0226556672,0.0018056207,-0.3695298423

T12

SCF Done: E(RMN15) = -1194.10132372

Sum of electronic and thermal Free Energies= -1193.813657

C,0,-0.0248510716,-0.010518824,0.0142940263

C,0,-0.0147125715,0.0290585492,1.520669601

C,0,1.2383596605,-0.0004339702,2.2591778381

C,0,2.4612271118,-0.130623114,1.5244885999

C,0,2.4380479522,-0.2770330853,0.0086748842

C,0,1.2741015164,0.5149930156,-0.5750397351

H,0,1.4020923791,1.5817638436,-0.3426995148

H,0,1.2636577114,0.4078464893,-1.6647240431

H,0,2.2724343908,-1.343774137,-0.2203663813

C,0,3.7873903547,0.1175924854,-0.5736415825

H,0,3.9356595432,1.2012438893,-0.4208602922

C,0,4.9250107374,-0.6204054703,0.130817408

C,0,4.9883708562,-0.126959848,1.5577566579

C,0,3.6507663396,-0.0796387907,2.2136071273

H,0,3.6678395842,0.0420479186,3.2930324794

H,0,5.4022331108,0.8926864315,1.5896460485

Cl,0,6.0950792536,-1.127299764,2.5794018943

C,0,6.1297941436,-0.328643106,-0.7580392955

C,0,5.5532506301,-0.4184152094,-2.1899800863

C,0,4.0176830458,-0.2348938358,-2.0483954374

H,0,3.6173586515,0.5261584659,-2.7251555749

H,0,3.4955132865,-1.1760538693,-2.2691411111

H,0,5.9963546547,0.3418496744,-2.8402020228

H,0,5.7800731735,-1.3920157012,-2.636256055

H,0,6.478018485,0.6918857907,-0.5451196537

H,0,6.9689878584,-1.0106051299,-0.5885947712

H,0,4.692646983,-1.6973481488,0.1307753507

C,0,1.1958569502,0.2092471165,3.7362024017

C,0,-0.0688381056,-0.3121411342,4.4174874942

C,0,-1.3041169173,0.0710338637,3.6750601132

C,0,-1.1920420004,0.1231640798,2.2021629618

H,0,-2.1271990077,0.2329395251,1.6551110281

O,0,-2.3876334778,0.2935427434,4.2139027721

H,0,-0.044014437,-1.4111893667,4.4310187052

H,0,-0.1302078844,0.0360759229,5.4504593982

H,0,1.2538246023,1.3013771686,3.8736923878

H,0,2.0667068175,-0.2139663746,4.2350852471

H,0,-0.1603833087,-1.0570079406,-0.2941708272

H,0,-0.8975864129,0.5441507338,-0.3429396794

Coordinates, Electronic Energies and Free Energies for Reactions with HOCl; MN15/6-31+G(d,p) + SMD water

Testosterone

Reaction complex

SCF Done: E(RMN15) = -2266.22240153

Sum of electronic and thermal Free Energies= -2265.592891

C,0,1.054039122,1.096198835,-1.4047084019

C,0,1.4558015263,0.9235026015,0.030669361

C,0,0.3549123539,0.8984370757,1.0727960341

C,0,-0.7825391611,-0.0529704055,0.5836611108

C,0,-1.2233998912,0.2100417367,-0.8698804108

C,0,-0.0282657495,0.0944983244,-1.8100040689

H,0,0.3685203284,-0.9333559619,-1.7670400358

H,0,-0.3327294566,0.2851459233,-2.8462871523

H,0,-1.6279655926,1.2309010526,-0.9573707611

C,0,-2.3216459409,-0.781230046,-1.2269962517

H,0,-1.9126378525,-1.7934526725,-1.0406615552

C,0,-3.557798879,-0.6574565093,-0.3248465459

C,0,-3.1325757586,-0.9674513841,1.1098569367

C,0,-1.9760143202,-0.0589942687,1.5548903782

H,0,-2.3431558549,0.9705775995,1.661658941

C,0,-4.4450776548,-1.7118295448,-0.994761802

C,0,-4.2842629592,-1.4353888936,-2.504409491

C,0,-2.8925921565,-0.7665377965,-2.6532479984

H,0,-2.9872512375,0.2684450089,-3.0113908021

H,0,-2.2450263822,-1.2946930782,-3.3608023687

H,0,-4.3764875755,-2.3550365255,-3.0909063382

H,0,-5.0891246686,-0.7613509489,-2.8232410304

O,0,-5.7914080294,-1.6475576459,-0.5309678081

H,0,-6.2963116019,-2.3750459123,-0.9259528648

H,0,-4.0297293753,-2.7035675742,-0.7526416738

C,0,-4.2302543271,0.7198811997,-0.415928759

H,0,-5.1621397586,0.7188378075,0.1639447517

H,0,-3.5887989267,1.514126649,-0.0189703277

H,0,-4.4838069388,0.9887584755,-1.4486223659

C,0,0.9088630965,0.3756895068,2.4123498144

C,0,2.241182492,1.0085251075,2.8109262715

C,0,3.2604768456,0.81266224,1.7289021399

C,0,2.772281315,0.8444790394,0.3551006213

H,0,3.5249479794,0.8449273336,-0.4305523302

O,0,4.4722611551,0.6689676072,1.9751459546

H,0,2.126434901,2.0947597169,2.9471483544

H,0,2.6261976095,0.5994374406,3.7496598277

H,0,1.0449020751,-0.7141044448,2.3332797816

H,0,0.1761319371,0.5544463475,3.2061059817

H,0,0.6379263695,2.1122493377,-1.5149960903

H,0,1.9397926161,1.0352320906,-2.0482247613

Cl,0,2.7717068788,-2.0758893738,0.0806752663

O,0,3.2529829682,-3.6985272882,-0.0659348588

H,0,2.6498709727,-4.095138252,-0.7258384459

H,0,5.1224756355,-3.2998732243,-0.7566087237

O,0,5.8830114726,-2.7769022589,-1.0706513033

H,0,6.6687898759,-3.1963595034,-0.6884447758

O,0,3.0094826976,3.7443173865,-1.1204086531

H,0,3.5645158957,4.5215054106,-0.9459517317

O,0,0.35546811,4.905842222,-1.1354071467

H,0,0.1616148114,4.9766533147,-0.1870122373

O,0,-2.2320803255,3.7234619036,-1.7155212176

H,0,-2.600991137,3.7244764882,-0.8078801243

H,0,-1.3107639122,4.0392868075,-1.6119581695

H,0,2.9535525987,3.2678123358,-0.2738639784

H,0,1.225458162,4.4549635749,-1.1862159543

O,0,6.0017943286,0.0108701692,-0.4059608563

H,0,5.514848252,0.1831231414,0.4271746378

H,0,5.8953561774,-0.9452721365,-0.599846379

H,0,-3.9787516092,-0.8545641047,1.8049559823

O,0,-0.1524827537,-3.8408008523,-0.8042114123

H,0,0.390896726,-4.5995603482,-1.0676276153

H,0,-1.0127293839,-4.216755832,-0.5213995127

O,0,-0.2345531873,-3.3967095726,2.0890739182

H,0,-0.4546905815,-2.4909301494,2.3617209627

H,0,0.0433143513,-3.3331403454,1.15207223

H,0,-3.229395914,-4.2603936966,0.7034186465

O,0,-2.3912345164,-4.7463867843,0.7471835034

H,0,-1.8481397013,-4.2811695369,1.4175650938

O,0,4.9872665517,1.8555264548,-2.3900732765

H,0,5.3090256108,1.2354989199,-1.7011650569

H,0,4.3334390895,2.4372579146,-1.9570439674

O,0,-3.1449185723,4.0698158913,1.021147212

H,0,-3.5627949919,3.3991369634,1.6062159526

H,0,-2.279767943,4.2589248373,1.417609448

O,0,-4.3999338469,2.2111348087,2.7935758649

H,0,-4.6263415109,1.4373736493,2.2507279763

H,0,-3.682573401,1.9080470197,3.3733367184

H,0,-2.8068363943,-2.0198753478,1.1574073826

H,0,-1.6449229663,-0.3767408918,2.5525408841

H,0,-0.3413460301,-1.0690715918,0.5702454909

C,0,-0.1374874425,2.3559244957,1.2350748856

H,0,-0.7690352151,2.4493288817,2.1268293722

H,0,0.7120446645,3.0431602869,1.348080274

H,0,-0.7168694106,2.6809657308,0.3640959681

Chlorination TS at C4

SCF Done: E(RMN15) = -2266.19827039

Sum of electronic and thermal Free Energies=   -2265.564512

C,0,-1.0531184081,-0.9908711452,-1.3948574529

C,0,-1.4504641671,-0.7310056918,0.0108690139

C,0,-0.4062511116,-0.6333580441,1.0788283741

C,0,0.8625357657,0.1155674276,0.5774230985

C,0,1.2739832182,-0.2376044413,-0.8657762033

C,0,0.1037941849,-0.0856742869,-1.8275049979

H,0,-0.2304109478,0.9635460135,-1.8480083637

H,0,0.4028325209,-0.3579075147,-2.8456504693

H,0,1.6081935733,-1.2873472845,-0.9034255137

C,0,2.4412611529,0.6681186885,-1.2381712999

H,0,2.1039382474,1.7080076909,-1.0492650131

C,0,3.6716877601,0.448635134,-0.3442336795

C,0,3.2822957312,0.7577173401,1.1021080986

C,0,2.0562077005,-0.0644832883,1.5313015611

H,0,2.3219881189,-1.1306514473,1.5621510393

C,0,4.6243634857,1.4515120453,-1.0045924964

C,0,4.4297916369,1.2133476744,-2.5182361989

C,0,3.002646491,0.622491325,-2.6663634777

H,0,3.0385471806,-0.4143232077,-3.0280212972

H,0,2.3827957597,1.1884136854,-3.3693194516

H,0,4.5631613223,2.138648385,-3.0875931942

H,0,5.194082786,0.5048952329,-2.8610179412

O,0,5.9676185806,1.2809986882,-0.5574552811

H,0,6.5105376082,2.0021513944,-0.9111966634

H,0,4.2879998894,2.4651481452,-0.7361856044

C,0,4.2566191306,-0.9668110667,-0.4664650384

H,0,5.1921669369,-1.0293552497,0.1028217676

H,0,3.5761052726,-1.7307786092,-0.0753961441

H,0,4.4856112065,-1.229931889,-1.5060023237

C,0,-0.9697337087,0.0469943978,2.3411461922

C,0,-2.2809524637,-0.5677494096,2.8290248784

C,0,-3.3113608185,-0.6516704876,1.747268408

C,0,-2.8323905541,-0.6525100489,0.3318911564

H,0,-3.5489383289,-1.0386649403,-0.390125014

O,0,-4.5202001664,-0.7351031352,1.9636626002

H,0,-2.1191412198,-1.601087762,3.1711251043

H,0,-2.7029212555,-0.0181758381,3.6755858507

H,0,-1.1105305752,1.1163994661,2.1335614042

H,0,-0.2275452069,-0.0301665076,3.1415705735

H,0,-0.7039514181,-2.0396000169,-1.4140050586

H,0,-1.923851587,-0.9258771374,-2.0552969189

Cl,0,-2.7173579938,1.3194039723,-0.2058350368

O,0,-2.9772888091,3.3871812058,-0.7538036182

H,0,-2.997931965,3.3275236599,-1.72464868

H,0,-4.6578632244,3.2052457965,-0.3772799284

O,0,-5.6203621273,3.0028118007,-0.22254332

H,0,-5.8264821843,3.3210536927,0.6690398693

O,0,-3.4556938087,-3.3350729499,-1.6726258462

H,0,-3.8744296747,-4.0337482889,-2.2009900101

O,0,-0.9969098282,-4.6669334533,-0.8913697058

H,0,-1.03600773,-4.6695845984,0.0778363748

O,0,1.791389334,-3.9676187169,-1.3269814239

H,0,2.1704807411,-3.9007526417,-0.4264293379

H,0,0.834663069,-4.1364336061,-1.2018582304

H,0,-3.9272703809,-3.3383267678,-0.8234678387

H,0,-1.7799246919,-4.1521429068,-1.1816861054

O,0,-6.0830280884,0.2328652287,-0.4093779272

H,0,-5.6790642363,-0.1751816249,0.3794226528

H,0,-5.8976702913,1.1984849692,-0.3335550799

H,0,4.1215010618,0.5567382104,1.7847770307

O,0,-0.3577824025,3.6441886373,-0.1838975732

H,0,-1.3318084817,3.6278810369,-0.401693207

H,0,-0.0364460597,4.5356547357,-0.3892430692

O,0,0.7594212471,3.3391824863,2.3479916047

H,0,0.9500729054,2.409178598,2.5503990359

H,0,0.2232949698,3.3302865843,1.5202103983

H,0,2.5449348883,3.8832790225,-0.2854956434

O,0,2.66205444,4.4871951036,0.465637844

H,0,2.1243070387,4.0941321066,1.1839697254

O,0,-4.757616833,-0.841559378,-2.7098085654

H,0,-5.2011406351,-0.4740703817,-1.9133182317

H,0,-4.3056619183,-1.6555182017,-2.4145892649

O,0,2.8515102615,-3.9981847049,1.3876531632

H,0,3.4345834125,-3.3476141536,1.8381458675

H,0,2.0416387547,-4.0327433626,1.9200215908

O,0,4.575864271,-2.231045105,2.8295624438

H,0,5.2228878726,-1.8777834015,2.1978805011

H,0,4.058175991,-1.4603031891,3.1139541062

H,0,3.0439698682,1.8307193317,1.1813977138

H,0,1.7776802701,0.2161882636,2.5558362632

H,0,0.589252323,1.1866253674,0.5627937059

C,0,-0.1034134054,-2.1341315135,1.3780823277

H,0,0.4306206473,-2.1907690753,2.334011443

H,0,-1.0202970216,-2.7299894659,1.4647430347

H,0,0.5169561012,-2.5698016073,0.5893649231

Cationic intermediate

SCF Done: E(RMN15) = -2266.20373591

Sum of electronic and thermal Free Energies= -2265.572153

C,0,1.028724187,0.9499168122,-1.4185933076

C,0,1.3931753223,0.74742636,-0.0058120433

C,0,0.3715862064,0.6522263644,1.0500564758

C,0,-0.8978218582,-0.1079960305,0.5763288211

C,0,-1.3028447036,0.1999612035,-0.8781802633

C,0,-0.1282317856,0.0312719862,-1.8314035482

H,0,0.217564491,-1.0142219149,-1.8244867151

H,0,-0.4205013894,0.2793236455,-2.8569061005

H,0,-1.6501309243,1.2430815041,-0.9485884469

C,0,-2.4576895347,-0.7311420624,-1.2248123457

H,0,-2.1052817922,-1.7591317253,-1.0046215348

C,0,-3.6915050051,-0.4997441455,-0.3377899852

C,0,-3.302119458,-0.7526447478,1.119440068

C,0,-2.0886193744,0.1000170063,1.5243292047

H,0,-2.3613419212,1.164626595,1.5183653315

C,0,-4.6268932991,-1.539620141,-0.9651197329

C,0,-4.4349055417,-1.3479560295,-2.4859529437

C,0,-3.0183418521,-0.7369617383,-2.6534087648

H,0,-3.0720666755,0.287889608,-3.0454739971

H,0,-2.3885496692,-1.3131053309,-3.3389064089

H,0,-4.5508121962,-2.2938794782,-3.0243007519

H,0,-5.2113716606,-0.6650885195,-2.8522748793

O,0,-5.9726928574,-1.3757366726,-0.5243868771

H,0,-6.5049331621,-2.1145154543,-0.8575384683

H,0,-4.2734890863,-2.5383120824,-0.6633887117

C,0,-4.2972109755,0.9022170259,-0.5073421467

H,0,-5.2313538263,0.9722851725,0.0639922409

H,0,-3.6254942554,1.688910016,-0.1466660017

H,0,-4.5353277639,1.1259802086,-1.5538973359

C,0,0.9332198344,0.0589430291,2.3543556887

C,0,2.2126491732,0.7421701062,2.83769952

C,0,3.2716876571,0.7906579281,1.7849718854

C,0,2.8266372238,0.6539056009,0.3359456359

H,0,3.5054928075,1.1582749287,-0.3531103607

O,0,4.4677054344,0.9244815,2.0023965255

H,0,2.0116633839,1.788371385,3.1134404115

H,0,2.6252540381,0.2572805175,3.726984311

H,0,1.1047156116,-1.0158514755,2.2071563002

H,0,0.1723286119,0.1594266625,3.1332985499

H,0,0.6856321773,2.0021727651,-1.4723389496

H,0,1.9110034403,0.8700504057,-2.0611174201

Cl,0,2.7957821867,-1.1226431701,-0.0784888321

O,0,3.0722257875,-3.8002674863,-0.7847758124

H,0,3.0368944395,-3.5982075754,-1.7326467718

H,0,4.5289238426,-3.3106703254,-0.3722652358

O,0,5.479868705,-3.0030638045,-0.1614632626

H,0,5.6868910839,-3.3300557777,0.7264068869

O,0,3.4135179974,3.3116106039,-1.8056413723

H,0,3.819202945,3.9508596851,-2.4136617166

O,0,0.9292155293,4.6377971354,-1.1161659042

H,0,0.9753877483,4.7255817304,-0.1513298334

O,0,-1.8424663683,3.864564679,-1.511349865

H,0,-2.2192777401,3.838941716,-0.6077480669

H,0,-0.8898869757,4.062018726,-1.3970293904

H,0,3.9092647048,3.3982077565,-0.9748086786

H,0,1.7305816472,4.1319957084,-1.3702369678

O,0,6.0249732261,-0.2771237255,-0.3780913124

H,0,5.7015005009,0.1699163207,0.4239911901

H,0,5.8002896472,-1.2343543522,-0.2708518653

H,0,-4.146118133,-0.5410960398,1.7923635383

O,0,0.6216808738,-3.5758255839,-0.0121649628

H,0,1.600848448,-3.6757259686,-0.3140188406

H,0,0.1641529145,-4.3938508215,-0.2605872863

O,0,-0.6505522117,-3.2722272879,2.4261538851

H,0,-0.8698892786,-2.3459853676,2.6161398053

H,0,-0.0617590069,-3.2579235387,1.6325242512

H,0,-2.4203013482,-3.8937079257,-0.228762419

O,0,-2.4817067215,-4.4850555188,0.5383716443

H,0,-1.9494113731,-4.0486929856,1.2365734361

O,0,4.6895730473,0.7395100004,-2.7083124638

H,0,5.1270925182,0.4021880167,-1.8958156369

H,0,4.257155497,1.5775024933,-2.4550181663

O,0,-2.8808357707,4.019172257,1.207246762

H,0,-3.4362674484,3.3819255012,1.7088458767

H,0,-2.0651102589,4.1125939763,1.7233823692

O,0,-4.4893261515,2.272454405,2.8052218855

H,0,-4.9476904419,1.6664850707,2.2001719158

H,0,-3.8567829654,1.7136991969,3.2852269359

H,0,-3.0465830548,-1.8182481994,1.2373982956

H,0,-1.8083165919,-0.1474774726,2.5564367923

H,0,-0.6181753399,-1.176973928,0.6004793357

C,0,0.0923264685,2.1917396028,1.2439277226

H,0,-0.3855871705,2.2986828305,2.2250912424

H,0,1.0062236979,2.7988862642,1.2440218748

H,0,-0.5702691379,2.5593443901,0.4568930882

C4-C5 chlorohydrin formation TS

SCF Done: E(RMN15) = -2266.20147256

Sum of electronic and thermal Free Energies=   -2265.568488

C,0,0.8237405267,0.455904677,-1.2192650876

C,0,1.1044537736,0.4308144067,0.1966593622

C,0,0.0443173267,0.4567025836,1.2239700392

C,0,-1.2212434738,-0.3123037377,0.7647675126

C,0,-1.6388871569,0.0516125045,-0.6787056742

C,0,-0.4984610441,-0.1400317472,-1.6816149012

H,0,-0.3490344217,-1.2125666747,-1.8699152846

H,0,-0.7687278095,0.3108711451,-2.6430099107

H,0,-1.9591217914,1.1055519816,-0.7180164429

C,0,-2.820431273,-0.8366140902,-1.0523553681

H,0,-2.4586172086,-1.8775165761,-0.9517055812

C,0,-4.0211704148,-0.7074427044,-0.1019726495

C,0,-3.5734323899,-1.0619884191,1.3149575367

C,0,-2.3960537304,-0.176442493,1.7461360309

H,0,-2.7189872409,0.8724401699,1.8117247281

C,0,-4.9590544897,-1.7154842124,-0.7728507779

C,0,-4.8180514802,-1.4160521795,-2.2844488648

C,0,-3.4462529075,-0.7088324283,-2.4458522422

H,0,-3.5744340329,0.3516942597,-2.7040953043

H,0,-2.8256308172,-1.1625178192,-3.2254437205

H,0,-4.8845565873,-2.3341501936,-2.8766758345

H,0,-5.646001198,-0.7666001494,-2.5939022618

O,0,-6.2910737249,-1.5977384335,-0.2796849171

H,0,-6.8369172314,-2.2945450539,-0.6754023051

H,0,-4.5840471248,-2.7286843308,-0.5519474941

C,0,-4.6615705877,0.6897016273,-0.1354743985

H,0,-5.4083769208,0.7782143748,0.6640133844

H,0,-3.9206742536,1.4818524856,0.009684144

H,0,-5.1698242625,0.8864078372,-1.0866407999

C,0,0.6503460015,-0.0312521358,2.5599968098

C,0,1.7387893566,0.9156390113,3.0837607408

C,0,2.5625148902,1.511485461,1.980029162

C,0,2.5155893415,0.7954578362,0.6286155805

H,0,2.9831388272,1.411497784,-0.1471565006

O,0,3.2672664335,2.5029535247,2.1023396705

H,0,1.3226460721,1.7516543059,3.6558506693

H,0,2.4231635937,0.3839664401,3.7570707327

H,0,1.0544396011,-1.0378086254,2.4121257875

H,0,-0.1447572903,-0.0991371474,3.3073730874

H,0,0.7896568788,1.5763086184,-1.334463885

H,0,1.704260396,0.1680536302,-1.8087412629

Cl,0,3.5544021725,-0.6410462668,0.8517339291

O,0,3.0712172699,-3.2406841633,-1.1081129043

H,0,2.80613424,-4.1247171494,-1.4031380711

H,0,4.4500027604,-3.2753726751,-0.4939381555

O,0,5.4203989341,-3.2600161292,-0.1287879081

H,0,5.3672630505,-3.3060306384,0.8371495692

O,0,3.2171526317,2.5821537959,-2.5874640989

H,0,3.5816669698,1.6674057771,-2.5235105068

O,0,2.1527628609,4.0532234514,-0.4915586906

H,0,2.4814579448,3.7710123443,0.3776098333

O,0,-0.2835179577,3.5231844599,-1.9161899999

H,0,-1.1406087352,3.6283922215,-1.4489054534

H,0,0.4194062684,3.7656900704,-1.279839988

H,0,3.8948056981,3.1095970775,-3.0368155908

H,0,2.61846755,3.4943690958,-1.1572187508

O,0,6.3955412276,-0.9399580836,-1.1801808094

H,0,6.5818956267,-0.3087434798,-0.4686203587

H,0,6.0700348241,-1.7676391431,-0.7373576273

H,0,-4.4056729256,-0.9586160971,2.0243948669

O,0,1.1324358898,-2.1981861577,0.1348220833

H,0,1.9579908061,-2.6179101508,-0.3600335743

H,0,0.3664765877,-2.4703271663,-0.3981540198

O,0,-0.2780793572,-3.3951063594,2.3038534753

H,0,-0.7521498329,-2.6489144136,2.7072954167

H,0,0.3542946751,-2.985727827,1.6689171147

H,0,-2.6275772186,-3.8949407995,0.0029350132

O,0,-1.7877189215,-4.3745435673,0.0789298327

H,0,-1.352892867,-4.02319208,0.8861749322

O,0,4.1395485522,-0.0340156535,-2.5408161104

H,0,4.9366295349,-0.3431218968,-2.0458163387

H,0,4.2679308732,-0.3178970927,-3.4586928695

O,0,-2.8586494223,3.8752976934,-0.8166717789

H,0,-3.0663767273,3.7431449563,0.1357798864

H,0,-3.117337611,4.7868788608,-1.0207225437

O,0,-3.5447236544,3.5174696937,1.9023879682

H,0,-4.1460630127,2.755441192,1.9305440944

H,0,-2.7864430609,3.2653388462,2.4532328163

H,0,-3.2505457639,-2.1165854427,1.3363046514

H,0,-2.0784992926,-0.473233608,2.753299277

H,0,-0.9501632117,-1.3772247115,0.7524590659

C,0,-0.2976077608,1.9911576983,1.3571584059

H,0,-0.7768740835,2.1138719161,2.3349842472

H,0,0.5700263158,2.6585903847,1.3221255052

H,0,-0.9795580032,2.3037627042,0.5632626544

C4-C5 chlorohydrin

SCF Done: E(RMN15) = -2266.30353138

Sum of electronic and thermal Free Energies=   -2265.663268

C,0,0.598794946,-0.5021936893,-0.9132480442

C,0,0.6347492934,-0.8324795774,0.5832283044

C,0,-0.0249545362,0.2706776552,1.4659110009

C,0,-1.4516306837,0.560723439,0.9164333624

C,0,-1.4256505983,0.9642560938,-0.5735330073

C,0,-0.8095595865,-0.1734928334,-1.3909639746

H,0,-1.4599474593,-1.0611396133,-1.2966810129

H,0,-0.7873664194,0.0816548545,-2.4586379524

H,0,-0.8058713658,1.8689986214,-0.7030987157

C,0,-2.8433514135,1.2580466851,-1.0408888236

H,0,-3.4085975195,0.3156827989,-0.9164143139

C,0,-3.577526421,2.3036306382,-0.1896127382

C,0,-3.6625163902,1.789072515,1.2462499925

C,0,-2.2581755708,1.5262957254,1.8047612801

H,0,-1.7301303855,2.4818979875,1.9161881534

C,0,-4.9048344521,2.342215482,-0.952911869

C,0,-4.4870722933,2.3600541184,-2.4410496805

C,0,-3.0683660813,1.7335051777,-2.4826607778

H,0,-2.3139958662,2.4876514487,-2.7522741189

H,0,-2.9882750544,0.918461069,-3.2090669656

H,0,-5.2042164713,1.813423177,-3.0615774806

H,0,-4.4784230717,3.3999269917,-2.7902864023

O,0,-5.7040245553,3.4555522146,-0.5610208114

H,0,-6.5573643653,3.4093121761,-1.0190680334

H,0,-5.4517534248,1.4114013739,-0.7252752826

C,0,-2.9098637751,3.6867375828,-0.2365009883

H,0,-3.4234362114,4.3744881537,0.4463877644

H,0,-1.8580404274,3.6431129168,0.0681110197

H,0,-2.9457167352,4.1263443775,-1.2403630439

C,0,-0.1208317405,-0.2436136215,2.9218606538

C,0,1.0853691941,-1.0889541897,3.3902706069

C,0,2.300570998,-0.910511806,2.5427668402

C,0,2.0930777337,-0.9724743789,1.0309358749

H,0,2.6695435224,-0.1591294335,0.5790910829

O,0,3.4318758493,-0.7396068638,2.9902082614

H,0,1.3478708394,-0.9044176484,4.4344263143

H,0,0.8375608687,-2.1559231633,3.2864700125

H,0,-1.0316543335,-0.840648466,3.0451463242

H,0,-0.2192554167,0.6271955018,3.5798835971

H,0,1.2657926315,0.351299162,-1.1061707209

H,0,1.0134712007,-1.3576239293,-1.460484313

Cl,0,2.8540342672,-2.4904497112,0.4441389368

O,0,0.3013861656,-4.0286971827,-1.2721561224

H,0,-0.5623743108,-4.4640974557,-1.3361749445

H,0,1.615971497,-5.1805136106,-0.8762573608

O,0,2.3848402421,-5.7816091472,-0.7278000513

H,0,2.523718616,-5.8097926634,0.2311147034

O,0,4.3082728994,0.1088748887,-1.6594910643

H,0,3.8443260746,-0.6526960882,-2.0751951825

O,0,5.0334994749,0.8699608111,1.0279140839

H,0,4.643015596,0.2942236478,1.7120665703

O,0,3.4013262083,2.6538304808,-0.7156877075

H,0,2.6430711784,3.2392340005,-0.5120443153

H,0,3.7204567823,2.2829877703,0.1285324515

H,0,3.6788816256,0.8534933543,-1.6171389625

H,0,4.87966589,0.4134395801,0.1738529344

O,0,4.4335935309,-4.4036387249,-2.0001879484

H,0,5.0942095847,-4.1427995114,-1.3405410448

H,0,3.7424971009,-4.9116405575,-1.5109884926

H,0,-4.1964211974,2.5038579467,1.8878496872

O,0,-0.0115635381,-2.0906149762,0.8220890988

H,0,0.2226602577,-3.370431355,-0.5478063626

H,0,-0.976250181,-1.9836046192,0.7447600258

O,0,-3.5668926182,-1.919303762,2.5806003736

H,0,-3.3687758629,-0.9996853632,2.825012547

H,0,-2.7050561335,-2.3132092885,2.3659266528

H,0,-5.0700317114,-0.84919279,-0.1350347424

O,0,-4.8833802428,-1.7814418086,0.0585597641

H,0,-4.440341559,-1.7831269721,0.934787371

O,0,3.1917497426,-2.1119910378,-3.00566403

H,0,3.6228270204,-2.91793464,-2.6335759154

H,0,3.4741192171,-2.0652604706,-3.931763593

O,0,1.0331883943,4.3445595864,-0.6443434467

H,0,0.7916267746,3.8049878314,-1.4307760886

H,0,0.4319070825,4.0642364643,0.0639777694

O,0,0.7856367831,2.6941444465,-2.9099259658

H,0,1.4570325749,2.0311891195,-2.6765710688

H,0,-0.0544924574,2.2080803093,-2.953950256

H,0,-4.2390117681,0.8480713597,1.2505074406

H,0,-2.345755956,1.103286784,2.8140980681

H,0,-2.0174554531,-0.3928663015,0.9515494327

C,0,0.8399916554,1.5460412074,1.4487671717
H,0,0.2912689579,2.3807183301,1.8982417487
H,0,1.7624420207,1.4337065708,2.0353391137
H,0,1.1281683227,1.8353048238,0.4347973457

TS for epoxide formation

SCF Done: E(RMN15) = -2266.27098930

Sum of electronic and thermal Free Energies= -2265.628180

C,0,0.2710762228,-1.0128943802,0.3741347996
C,0,0.4651135547,0.0564427733,-0.6904631265
C,0,-0.6622020478,0.0885063584,-1.748517628
C,0,-1.9851976289,0.3101095173,-0.9541312526
C,0,-2.2366651988,-0.8397081769,0.0470120128
C,0,-1.0878711286,-0.8853369896,1.0578580764
H,0,-1.1167837892,0.0448651653,1.6489168133
H,0,-1.2277387825,-1.7155214911,1.7620143209
H,0,-2.2733919836,-1.8019695715,-0.4899461277
C,0,-3.5714532052,-0.6262306658,0.7462589615
H,0,-3.5043875159,0.3436648324,1.2781105711
C,0,-4.7532999833,-0.4781641381,-0.222390307
C,0,-4.5075544054,0.7583186607,-1.0831986185
C,0,-3.1902372981,0.6276633808,-1.8599530733
H,0,-3.3023073127,-0.1427622255,-2.6347274661
C,0,-5.8891005572,-0.3499950329,0.7973255751
C,0,-5.578214084,-1.4333243418,1.8532879533
C,0,-4.0488366797,-1.6741081198,1.7622337316
H,0,-3.8303384096,-2.6837890524,1.3873127731
H,0,-3.5476644046,-1.5727882889,2.7303819955

H,0,-5.8963564512,-1.1184059253,2.8523170537

H,0,-6.1432086704,-2.3394492882,1.6014439108

O,0,-7.1686619239,-0.49021094,0.1833158334

H,0,-7.8565010322,-0.3106037636,0.842687693

H,0,-5.8199535049,0.6505428697,1.2534348113

C,0,-4.9736005985,-1.7221034863,-1.0952576473

H,0,-5.7971445868,-1.5444048631,-1.7976894773

H,0,-4.0840704772,-1.9711176847,-1.6839755466

H,0,-5.2309291468,-2.6032856623,-0.4952793156

C,0,-0.4224422668,1.2804202222,-2.6947496062

C,0,1.0040436121,1.3654698383,-3.2492216203

C,0,2.0938004223,1.2208766578,-2.2314450328

C,0,1.8704058231,0.2160470542,-1.140242581

O,0,3.1580249835,1.8399570702,-2.3075727624

H,0,1.1801201072,0.5390737,-3.9554584299

H,0,1.1692730861,2.2953224355,-3.8010143294

H,0,-0.6638698686,2.2161339536,-2.1755660312

H,0,-1.1084610469,1.2025442732,-3.5468353336

H,0,0.3656334096,-2.0030139113,-0.0892286712

H,0,1.090441826,-0.9157594767,1.1011400154

O,0,0.8642292344,1.7456592854,2.456653587

H,0,-0.0537539712,1.940893172,2.7379889428

H,0,1.74739249,3.2767206015,1.906357302

O,0,2.4401624753,3.7953018665,1.4327761402

O,0,4.943171914,-1.1513297061,0.8871994989

H,0,4.1872505708,-0.9678240714,1.4724267753

O,0,5.2214413177,1.1745468152,-0.5381900217

H,0,4.5349807423,1.3591531071,-1.2206361292

O,0,5.1223357839,-3.9073672105,0.362690227

H,0,4.1683329961,-4.0359596222,0.1504553988

H,0,5.6021044753,-4.0229863226,-0.471724187

H,0,4.9739450921,-2.1229392006,0.7308775111

H,0,5.1500270591,0.2202120455,-0.298828783

O,0,4.3203970775,2.0680999794,1.6368622155

H,0,4.6991001665,1.7498475183,0.7142148268

H,0,3.615772974,2.8083763344,1.5229012566

H,0,-5.3405674839,0.9244513493,-1.7809984263

O,0,0.7042749345,1.3467207091,-0.1573876643

H,0,0.7889464551,1.4990607448,1.4872593322

O,0,-0.7609424218,3.6521236398,0.1378361582

H,0,-1.4471577986,3.7250826703,-0.5445003497

H,0,-0.2581559533,2.8276691957,-0.080428634

H,0,-2.4039643969,1.8688537386,2.256391697

O,0,-1.8739213641,2.6421650943,2.5073425548

H,0,-1.5701124563,3.0381695298,1.6568137159

O,0,2.9962235614,0.0729022794,2.8654847788

H,0,2.1312931533,0.5544367043,2.8809061388

H,0,3.314093565,0.0437295453,3.7831832547

O,0,2.3634392606,-3.8501363325,-0.0045569213

H,0,2.262698731,-3.405534302,0.8677772503

H,0,2.3377457088,-3.1367216181,-0.6725209322

O,0,2.4313779014,-2.7895537326,2.6095790215

H,0,3.2734047733,-3.1821232594,2.8895647961

H,0,2.553062602,-1.8215444524,2.6911755584

H,0,-4.4550634233,1.6367898253,-0.4186680471

H,0,-3.0003472062,1.5696068605,-2.3889527355

H,0,-1.8238100678,1.2109534566,-0.3291633107

C,0,-0.7099973056,-1.206798238,-2.5768804511

H,0,-1.6419426922,-1.2504390333,-3.1532514575

H,0,0.1219819865,-1.2514636415,-3.2861081068

H,0,-0.6557160038,-2.1107183347,-1.9622565997

Cl,0,2.7270646121,-1.5064856506,-2.2879795161

H,0,2.6345263805,0.1197189437,-0.3723222547

H,0,2.5818515077,4.6118367248,1.9371978543

H,0,3.8852729845,1.2922957046,2.1040308175

C4-C5 epoxide

SCF Done: E(RMN15) = -2266.30266050

Sum of electronic and thermal Free Energies= -2265.661523

C,0,0.2742686923,-0.8856745166,0.6480806104

C,0,0.3313540301,0.3431137567,-0.2261671063

C,0,-0.6698619031,0.4076524914,-1.3749303154

C,0,-2.0784156699,0.2465777862,-0.7314120447

C,0,-2.1940060868,-1.0258163436,0.140041788

C,0,-1.128600897,-1.0159726725,1.2385409053

H,0,-1.3223392668,-0.1614996662,1.9103798749

H,0,-1.1971932227,-1.9264357439,1.8461419705

H,0,-2.0362352001,-1.9213753058,-0.4832847258

C,0,-3.5948846974,-1.0838826261,0.7346755948

H,0,-3.7205351478,-0.1559544563,1.3249175885

C,0,-4.7113836584,-1.0675757605,-0.3210396295

C,0,-4.6067638901,0.2339587049,-1.1131611257

C,0,-3.2192518944,0.3674150806,-1.7562161978

H,0,-3.0960635284,-0.3963746065,-2.5372329267

C,0,-5.9248178324,-1.180255371,0.607544844

C,0,-5.5174981498,-2.2583928379,1.6369845638

C,0,-3.9665817603,-2.2599539462,1.6485961984

H,0,-3.5694678966,-3.1974794172,1.2351006792

H,0,-3.5533618826,-2.14954737,2.6564301346

H,0,-5.9441651821,-2.0468881624,2.6226682694

H,0,-5.9185639966,-3.2252497984,1.3084044655

O,0,-7.1152481461,-1.4905962505,-0.1131767214

H,0,-7.8608520471,-1.5122201394,0.5064006132

H,0,-6.0585325286,-0.2091917025,1.1127314532

C,0,-4.6679142212,-2.2828664592,-1.259691087

H,0,-5.4530107352,-2.1942404547,-2.0203910154

H,0,-3.7092253746,-2.3638120205,-1.782314602

H,0,-4.8317931887,-3.2233745539,-0.7202917293

C,0,-0.567434295,1.7800118855,-2.0715577034

C,0,0.856530839,2.1737376151,-2.4711095044

C,0,1.8522494355,2.1127571396,-1.3537305134

C,0,1.5649124776,1.1527859112,-0.233551855

O,0,2.8690807508,2.8021281354,-1.31881854

H,0,1.2481839864,1.4660513522,-3.2189896101

H,0,0.8926463651,3.1696350988,-2.9234658788

H,0,-0.9939441925,2.5528597321,-1.4195723661

H,0,-1.1770595437,1.757576872,-2.9810581261

H,0,0.5159259097,-1.7690561518,0.0441095754

H,0,1.0357052064,-0.8021695422,1.4338234529

O,0,1.2800467596,1.4273799212,3.1668062821

H,0,0.6620086343,1.3655944211,3.9114469129

H,0,2.1063779069,3.0022234227,2.4688800912

O,0,2.6261964405,3.5533502958,1.8441002864

O,0,6.0998776114,-1.3285646538,0.6610866785

H,0,5.4929384665,-1.2395424401,1.4137847486

O,0,5.4479250941,0.9945245592,-0.6639284835

H,0,4.779371131,0.7224097798,-1.3385640237

O,0,5.3059488491,-3.5862575857,-0.7803705294

H,0,4.3876646819,-3.4430028612,-1.1027409499

H,0,5.8590306633,-3.6954088412,-1.5689306138

H,0,5.8035215715,-2.1276549569,0.1626545767

H,0,5.7835550903,0.1427425276,-0.2846551751

O,0,4.6301265703,1.9392447609,1.510221138

H,0,4.8995087367,1.635507084,0.5452490123

H,0,3.8899228424,2.6381468328,1.5344497707

H,0,-5.3882396199,0.2883072912,-1.8840678883

O,0,0.4135002957,1.5690251527,0.5363117978

H,0,0.7488486604,1.3685196059,2.3420560398

O,0,-3.1189176653,3.6518024266,0.0003244626

H,0,-3.2756204341,3.0755734594,-0.7666016508

H,0,-2.1954035002,3.4845892167,0.2515466485

H,0,-4.9514408646,1.6579194248,1.8500144238

O,0,-4.4638469289,2.4250661156,2.188404585

H,0,-4.0261455857,2.8220150504,1.4041649448

O,0,3.5356108595,-0.1043091856,2.7371986443

H,0,2.680428783,0.3220427707,2.9960796778

H,0,3.9887838206,-0.3455669109,3.561722551

O,0,2.5936931011,-2.9322342559,-1.326622223

H,0,2.5909582214,-2.714388095,-0.3687027994

H,0,2.799945979,-2.0879651387,-1.7903386273

O,0,3.0885338743,-2.621306617,1.4378753626

H,0,3.9656564999,-3.0355896698,1.4050674403

H,0,3.2214259141,-1.7429349512,1.855219104

H,0,-4.766635755,1.0795893256,-0.4222660608

H,0,-3.1571105381,1.3385463787,-2.2644983297

H,0,-2.184646391,1.09420288,-0.0241473635

C,0,-0.3430561065,-0.7069262039,-2.3839864606

H,0,-0.9254106108,-0.5673823317,-3.3022931093

H,0,0.7215103104,-0.68706913,-2.6507962898

H,0,-0.5656222277,-1.7048146587,-1.9914203279

Cl,0,3.5711605932,-0.3307661734,-2.7789557869

H,0,2.4267941521,0.8379053609,0.3618709266

H,0,2.8740524547,4.3582861238,2.3257654721

H,0,4.2806270019,1.1269287867,2.005647857

Dienogest

Reaction complex

SCF Done: E(RMN15) = -2739.05673850

Sum of electronic and thermal Free Energies= -2738.338099

C,0,-1.0695774961,-1.5798900284,-2.1473187749

C,0,-1.5096947914,-1.2168479461,-0.7554705557

C,0,-0.5427745404,-0.6891776662,0.1959654463

C,0,0.7863024871,-0.6459872297,-0.1091850074

C,0,1.2722221397,-1.2121340364,-1.4346169683

C,0,0.2444010427,-0.9161793733,-2.5255302654

H,0,0.1161947583,0.1737758902,-2.6164393093

H,0,0.5974156556,-1.2962415567,-3.4912212438

H,0,1.3177950134,-2.3119427918,-1.3278794684

C,0,2.671906087,-0.7351372429,-1.7982875913

H,0,2.597580817,0.3336916635,-2.0687280597

C,0,3.6422863454,-0.8380252099,-0.6096182458

C,0,3.1868240257,0.2106246324,0.4021569471

C,0,1.7679011563,-0.0873248902,0.9013412959

H,0,1.8250394405,-0.8009584559,1.734101736

C,0,5.0113336825,-0.6106011681,-1.3025759308

C,0,4.8732225596,-1.382325633,-2.6384799455

C,0,3.3608599385,-1.498133876,-2.9389272675

H,0,3.0426606728,-2.549245382,-2.9235189761

H,0,3.0986348903,-1.094607613,-3.9215762909

H,0,5.4258205185,-0.8776315491,-3.4382212643

H,0,5.3275008655,-2.3696990747,-2.5008473484

C,0,3.6349409609,-2.2493446993,0.0044968701

H,0,4.3583302534,-2.3186114697,0.8223100746

H,0,2.651639977,-2.5071312743,0.414679195

H,0,3.8932232158,-3.0124464128,-0.7403099706

C,0,-1.1121676903,-0.1523477174,1.4906127671

C,0,-2.2688150452,-1.0120083949,1.9972555316

C,0,-3.3030904717,-1.1955721044,0.9262752104

C,0,-2.8329028133,-1.3703321578,-0.4211231628

H,0,-3.5577413834,-1.6621039262,-1.179367564

O,0,-4.5273039922,-1.23603439,1.21600371

H,0,-1.9000765155,-2.0194397553,2.2563293401

H,0,-2.7385392869,-0.5848038928,2.8883359335

H,0,-1.4769401329,0.8762506109,1.3219852049

H,0,-0.3473790071,-0.0993220051,2.2709741449

H,0,-0.9313730897,-2.6731344588,-2.1653755748

H,0,-1.8791830678,-1.3534111506,-2.8518073362

Cl,0,-3.1390368797,1.4044706165,-1.5397626726

O,0,-3.743764438,2.8782779719,-2.1237193939

H,0,-4.5114072674,3.0515462301,-1.496649113

H,0,-6.2449329867,3.6304394163,-0.1311808112

O,0,-5.6414120157,2.8886027017,-0.290946343

H,0,-5.2670627959,2.6124426902,0.57946094

O,0,-5.3255466263,-3.4556977297,-0.4614461594

H,0,-6.1307899153,-3.943345056,-0.2273429914

O,0,-3.0570892589,-4.5002200005,1.0165202318

H,0,-3.1636748769,-4.0204351482,1.8533622956

O,0,-0.2464606308,-4.1674357219,0.3860692646

H,0,0.1821444133,-3.7501840903,1.1612480806

H,0,-1.205697959,-4.1987672461,0.5838303918

H,0,-5.3177891125,-2.6555999402,0.1041516824

H,0,-3.7892657223,-4.1841879209,0.444670091

O,0,-6.3775273918,0.0646441192,-0.610938553

H,0,-6.1932024428,1.0243969084,-0.5988150406

H,0,-5.7308803039,-0.343622307,0.0005889066

H,0,3.8575654578,0.2789220283,1.2685247805

O,0,-1.6758574417,4.4711488545,-1.1008239466

H,0,-2.4128634448,4.1151601125,-1.6394698073

H,0,-1.5695590851,5.4056608658,-1.3344631003

O,0,0.4183420767,2.6880750582,-0.488339612

H,0,0.384476634,2.6676781044,0.4892234045

H,0,-0.2838832965,3.3192665619,-0.7597866018

H,0,3.4722584583,3.3078730049,-1.4511805283

O,0,2.8978711073,3.8802480874,-0.9201627767

H,0,2.0217654036,3.4280048325,-0.8820748696

O,0,-5.6586273948,-1.6324995785,-2.7813744709

H,0,-5.9076071536,-0.9215911337,-2.1523197743

H,0,-5.508613653,-2.4033263838,-2.2012744113

O,0,0.9652688654,-3.0436252256,2.7710981598

H,0,1.8809692452,-2.7107002636,2.8773240918

H,0,0.3939728888,-2.2734238892,2.9258711616

O,0,3.5241496573,-1.7690212233,3.4867809301

H,0,4.3406302675,-1.4825761754,3.0200320183

H,0,3.0890169556,-0.9461837145,3.7600068123

H,0,3.1838268012,1.1944433918,-0.0892968499

H,0,1.3537025057,0.8277784006,1.3431031013

O,0,6.0610735897,-1.1311492906,-0.4853049932

H,0,6.9143177613,-1.0262521801,-0.9395132127

C,0,5.3412586441,0.869502644,-1.6021462589

H,0,6.2126059275,0.9006972501,-2.2685780264

H,0,4.5154961813,1.3648511699,-2.1261228161

C,0,5.6911293564,1.6325592142,-0.4033776592

N,0,5.9999966654,2.2312937475,0.5467337659

O,0,0.1415345502,2.9807426117,2.497438383

H,0,0.1049205261,2.0901836465,2.8799334917

H,0,-0.7827707915,3.1986218812,2.2205474282

O,0,-2.3657035224,3.6471814935,1.5394404154

H,0,-2.2044876025,3.9289639519,0.6136240877

H,0,-3.0114756436,2.9131662962,1.5221065196

H,0,3.0788958997,3.5089639833,0.9728542353

O,0,2.9597344883,3.3364396544,1.9293759013

H,0,1.9927985393,3.2888001651,2.0650491007

H,0,6.0104278232,-0.8716991743,1.3671211532

O,0,5.9903575794,-0.7786395807,2.3450054697

H,0,5.9115950847,0.1758127443,2.503723793

O,0,-4.4910843362,1.7132035804,1.9442281423

H,0,-4.8325791113,1.9584829743,2.8188380339

H,0,-4.6366177732,0.7521802244,1.8560013025

Chlorination TS at C4

SCF Done: E(RMN15) = -2739.03558251

Sum of electronic and thermal Free Energies= -2738.316047

C,0,-1.109310771,-1.4009514294,-2.1370385204

C,0,-1.5131647589,-1.0528945688,-0.7446136227

C,0,-0.5665936092,-0.6342118967,0.2304750528

C,0,0.7759343856,-0.5805916834,-0.0968090465

C,0,1.2466092607,-1.0771820433,-1.4457409129

C,0,0.2124987663,-0.7579822568,-2.5227290173

H,0,0.0946352944,0.3324709061,-2.6069398917

H,0,0.5534256012,-1.1385399253,-3.4913381483

H,0,1.2864236991,-2.1803994017,-1.3616859575

C,0,2.6477507321,-0.5939828112,-1.7953327964

H,0,2.5793586639,0.4868558156,-2.0102461345

C,0,3.625887027,-0.7701630558,-0.6215859113

C,0,3.1813953008,0.216203605,0.4552887475

C,0,1.7595277097,-0.0976913499,0.932297608

H,0,1.7934602838,-0.8693151413,1.7156584043

C,0,4.9888950562,-0.5048880117,-1.3130896055

C,0,4.8358577244,-1.2025149336,-2.6882087361

C,0,3.3202091299,-1.3036382258,-2.9785987259

H,0,3.0001707785,-2.3537435942,-3.009360922

H,0,3.0475085276,-0.8527704492,-3.9373446463

H,0,5.3782754478,-0.6534903263,-3.4652149717

H,0,5.2921490037,-2.1953711768,-2.6105513355

C,0,3.6229394231,-2.2147982211,-0.0907328636

H,0,4.3650472077,-2.3328996898,0.7039813051

H,0,2.6494881093,-2.4938687231,0.3289511577

H,0,3.8598085564,-2.9351532262,-0.8834400146

C,0,-1.1101172018,-0.2196472423,1.5804339646

C,0,-2.2588975407,-1.1309332287,2.0111534335

C,0,-3.3317177608,-1.1761114504,0.9674266977

C,0,-2.8995084065,-1.0731553319,-0.4331806031

H,0,-3.5837436213,-1.488911524,-1.1713079802

O,0,-4.5314287951,-1.3208122028,1.2599646276

H,0,-1.8909513528,-2.1664357782,2.1144187718

H,0,-2.693589242,-0.8301789507,2.9685424624

H,0,-1.4715870514,0.8193947682,1.5238210891

H,0,-0.3327993386,-0.2473325622,2.3485055892

H,0,-1.006121316,-2.501047369,-2.1558545307

H,0,-1.9265045923,-1.1527963189,-2.8241992099

Cl,0,-3.2310732438,1.0397544506,-0.8925889232

O,0,-3.7054834417,2.9474078999,-1.2907844682

H,0,-4.1119676894,2.8944482798,-2.1745593347

H,0,-5.159002252,2.9288360995,-0.2022174837

O,0,-5.9039370909,2.7551346164,0.4199547221

H,0,-5.4814793172,2.4165055209,1.236890841

O,0,-5.2778076392,-3.3484817975,-0.7865310203

H,0,-6.0052626471,-3.9819586799,-0.6834551971

O,0,-2.8695945294,-4.357867966,0.4229562978

H,0,-3.0190230917,-4.1085508633,1.3487211654

O,0,-0.0081029727,-4.1236235511,0.1413111578

H,0,0.3308558502,-3.7500365613,0.9814780217

H,0,-0.9849066831,-4.1107065766,0.2057481583

H,0,-5.3937646695,-2.6837009644,-0.0809031454

H,0,-3.6531158473,-4.0239026353,-0.0651516277

O,0,-6.5979210431,0.0832130254,-0.2911851909

H,0,-6.4031896982,1.0332247099,-0.1257364783

H,0,-5.9182042625,-0.4076322513,0.2110370433

H,0,3.8488034887,0.2202200528,1.3258933874

O,0,-1.3210578384,4.2147804569,-1.7353035239

H,0,-2.1995549811,3.7762898882,-1.6011835282

H,0,-1.3420504238,5.0228317733,-1.1999715182

O,0,0.6168645637,2.5771133909,-0.6244991072

H,0,0.4565510117,2.6156057245,0.3417296525

H,0,-0.0783443901,3.1507471195,-1.0288066344

H,0,3.6534548159,3.5713610528,-1.3927177604

O,0,3.0124938547,3.984090267,-0.7946122538

H,0,2.1855001255,3.4479243293,-0.8549558346

O,0,-5.7675029302,-1.197144833,-2.7187156781

H,0,-6.0555957436,-0.6105299912,-1.9870211849

H,0,-5.5684734404,-2.0406948281,-2.2676756129

O,0,0.9842970553,-3.1560912071,2.6656831781

H,0,1.8879209847,-2.7983432746,2.7997367153

H,0,0.3824056796,-2.430734204,2.8986127741

O,0,3.535794372,-1.9696451645,3.3298789645

H,0,4.3542811348,-1.6045166087,2.9248403094

H,0,3.7796504013,-2.2286943743,4.231321248

H,0,3.1856575747,1.2300190073,0.0310865487

H,0,1.3417842305,0.7847772283,1.4350077711

O,0,6.0444053525,-1.0698107208,-0.5346580729

H,0,6.8953344869,-0.9309848921,-0.9841344698

C,0,5.3166242861,0.9893351973,-1.5335979483

H,0,6.1811563218,1.0571393566,-2.2060391411

H,0,4.4859324694,1.5127157386,-2.0210396798

C,0,5.6778713422,1.6844669031,-0.2974421507

N,0,5.9952491371,2.2286732256,0.6821404645

O,0,-0.0046748611,3.0394042716,2.209444236

H,0,-0.000201064,2.2210740703,2.7308810441

H,0,-0.9474959498,3.1904705241,1.9589101342

O,0,-2.6870432758,3.4290183552,1.5071195881

H,0,-2.9115569905,3.4969246189,0.5620206548

H,0,-3.2326387082,2.6933709964,1.8549024576

H,0,3.1174909924,3.5321115823,1.0735362848

O,0,2.9088618573,3.3264659197,2.0082628018

H,0,1.9339136296,3.317867195,2.0503532756

H,0,6.0048841099,-0.8927218357,1.3337204814

O,0,5.9862854224,-0.8463904741,2.3149117283

H,0,5.9095287544,0.0994664452,2.5195204148

O,0,-4.5220596518,1.4500442991,2.5674957048

H,0,-4.7977278818,1.6082427922,3.4839972288

H,0,-4.6971333868,0.5089548763,2.3867397059

Cationic intermediate

SCF Done: E(RMN15) = -2739.06070574

Sum of electronic and thermal Free Energies=   -2738.346979

C,0,-0.0000464924,0.0005973353,0.0000122818

C,0,-0.0001043573,0.0001751229,1.4839422422

C,0,1.1350412738,0.0000307982,2.2683141733

C,0,2.3982208801,0.0478615676,1.6222240346

C,0,2.4798234172,-0.1066701761,0.1366195503

C,0,1.2960329629,0.5594457465,-0.5640351912

H,0,1.339852163,1.6456681915,-0.4034234781

H,0,1.3576950286,0.3713271155,-1.6400355638

H,0,2.3511244359,-1.2020705278,-0.0032024162

C,0,3.8348024426,0.2873458414,-0.4358479105

H,0,3.8980362118,1.388135055,-0.3901574043

C,0,5.002469249,-0.2800649099,0.3887390497

C,0,4.9471126447,0.4155695416,1.7463682935

C,0,3.6248600499,0.1117423402,2.4516677497

H,0,3.6638278478,-0.8801528447,2.9392341417

H,0,3.4605000884,0.8038043625,3.288986776

H,0,5.7709837399,0.1209830219,2.4071584329

H,0,5.0086810673,1.502167052,1.6001309001

C,0,6.2076408275,0.0386434241,-0.5344840387

C,0,5.6564364505,-0.2510679628,-1.9554270468

C,0,4.1139421672,-0.1738436676,-1.8711497854

H,0,3.6644814019,-1.1621282397,-2.0353606228

H,0,3.6875348985,0.5065094415,-2.6138971196

H,0,6.0706178278,0.4558449914,-2.681924427

H,0,5.9897667391,-1.2527843695,-2.2471096861

O,0,7.3069673375,-0.8059399182,-0.1961217281

H,0,8.0494243215,-0.6402338507,-0.8016795512

C,0,6.6995543339,1.5021086455,-0.4687858345

H,0,7.4049148862,1.6657284872,-1.2931806576

H,0,5.8716772121,2.2057559097,-0.6078087808

C,0,7.405867903,1.817175124,0.7732540384

N,0,7.9956490252,2.0554678461,1.7488185799

C,0,4.9079355437,-1.8085651958,0.5463725057

H,0,5.7597904944,-2.1840766296,1.1209260454

H,0,4.0000455861,-2.1133132039,1.0785165749

H,0,4.9069458851,-2.3148830133,-0.4261267562

C,0,0.9806256535,0.0060905335,3.7734572429

C,0,-0.1624969916,-0.9186709042,4.1992275272

C,0,-1.4449211033,-0.5609355169,3.521306149

C,0,-1.3369534473,0.1082823743,2.154134044

H,0,-2.1607669249,-0.2082039957,1.509644085

Cl,0,-1.5247614229,1.8866271254,2.4300273077

O,0,-2.5550277675,-0.7567373149,4.005266721

H,0,0.0696348616,-1.9503164996,3.8848277636

H,0,-0.3184125058,-0.923811791,5.2812991714

H,0,0.7861886248,1.0330410003,4.1181551122

H,0,1.8965844939,-0.327427248,4.2667476094

H,0,-0.1249770983,-1.0589473444,-0.2961860545

H,0,-0.8861855003,0.5298474844,-0.3684155539

O,0,-1.6130355621,4.9697860632,2.9652918537

H,0,-2.1928935335,4.6374054887,2.2621073343

H,0,-2.6479321507,4.2786284697,4.1567284254

O,0,-3.2521641704,3.8375628459,4.821880588

H,0,-2.6751536966,3.2563779865,5.3552863241

O,0,-4.0484187557,-1.8144605299,1.6798111676

H,0,-4.8056630758,-2.4209838838,1.6943175077

H,0,-3.9135633456,-1.5228253809,2.6011119145

O,0,-1.4060377604,-2.9021217985,1.7016564492

H,0,-1.4100908324,-3.3355044005,2.5697805845

H,0,-2.3283405134,-2.5982819468,1.5577608671

O,0,1.4091647465,-3.3180366806,1.0715653285

H,0,1.9045848022,-3.2304553558,1.9130826451

H,0,0.4866945617,-3.0620639966,1.2619978692

O,0,-4.61413136,1.5307942965,3.8859308425

H,0,-4.1430493166,2.3608286328,4.1325972411

H,0,-3.9789632775,0.8087314117,4.0380773491

O,0,0.5014660688,4.7704686553,1.3984708772

H,0,-0.2852562946,4.874496797,2.0295479249

H,0,0.900330623,5.6485074519,1.3066563721

O,0,2.5065137783,2.9392433276,1.8366004184

H,0,2.6071564055,2.8666683366,2.809046462

H,0,1.7613431968,3.5759654108,1.6965811531

H,0,5.2986796051,4.0866107487,0.4762930586

O,0,4.8895626681,4.3055483312,1.3268569877

H,0,4.03499086,3.8136616577,1.3657069215

O,0,-4.7058962797,0.9620238017,1.0535115967

H,0,-4.6591276839,1.270201384,1.9832931187

H,0,-4.5082094033,0.0065124635,1.1048479162

O,0,2.8877974644,-3.2318970821,3.530885018

H,0,3.8331557928,-2.9705183177,3.5673119747

H,0,2.4230643337,-2.6064615187,4.1098764361

O,0,5.5927756714,-2.2774464882,3.9871701491

H,0,6.343437969,-2.002787839,3.4139691231

H,0,5.3485471445,-1.4778279775,4.4796448909

O,0,2.6752501298,2.9639592243,4.7610076339

H,0,2.6374942507,2.0603463737,5.1124940719

H,0,1.7403403259,3.288765694,4.7463282563

O,0,0.0349398533,3.7651093154,4.7479628041

H,0,-0.5417471037,4.1366989167,4.0230805442

H,0,-0.4563257449,3.0055726179,5.1154383403

H,0,5.4276475109,3.4723819215,2.9874314683

O,0,5.4353496289,3.1053852035,3.8950553462

H,0,4.5049710789,3.1475722805,4.1904660703

H,0,7.7010711563,-1.1294587025,1.6084253113

O,0,7.9060851507,-1.3470504057,2.5441290477

H,0,8.0613788126,-0.4900227924,2.9728225537

O,0,-1.6424845547,1.7071592876,6.0109093084

H,0,-1.6156981069,1.6470975961,6.9787957266

H,0,-2.2377394615,0.9973578239,5.7167786418

TS for deprotonation at C4

SCF Done: E(RMN15) = -2739.06531589

Sum of electronic and thermal Free Energies=   -2738.349518

C,0,1.3225273581,-0.1275152309,-2.6083948246

C,0,1.7824439407,0.0630498663,-1.2030129021

C,0,3.2406383365,0.2645110011,-1.0038645374

C,0,3.6991976548,0.3637034487,0.4198289915

C,0,2.7802209431,1.1874700896,1.2588304938

C,0,1.3801861925,0.5663231594,1.2553738629

C,0,0.8949135408,0.2536499391,-0.1463204892

C,0,-0.4936262631,0.1926634542,-0.4030125526

C,0,-1.0119963986,0.2044641299,-1.8075038242

C,0,-0.1106007509,-0.6098769968,-2.7279915659

H,0,-0.185084422,-1.6716654825,-2.4583731312

H,0,-0.4530003838,-0.5020744169,-3.7617358243

H,0,-0.8932795786,1.2691733414,-2.1030737607

C,0,-2.4846312295,-0.1659970788,-1.9306932317

H,0,-2.5503303793,-1.2600394231,-1.8011549298

C,0,-3.3622204254,0.4762427115,-0.8449333787

C,0,-2.8891047758,-0.0954862365,0.4885062882

C,0,-1.4338321871,0.3051267902,0.7451234003

H,0,-1.3793229982,1.3737023301,1.0380771248

H,0,-1.022563064,-0.2349170402,1.6099494267

H,0,-3.5022121079,0.243511118,1.3315179002

H,0,-2.9437554137,-1.192223313,0.4570363888

C,0,-4.7800861191,0.0882868114,-1.3390339637

C,0,-4.6760669588,0.2416034984,-2.8822455311

C,0,-3.172111476,0.2206352988,-3.2432538836

H,0,-2.830810198,1.2167041913,-3.5552633465

H,0,-2.9461843128,-0.4748709586,-4.0567908587

H,0,-5.2312464544,-0.5571717535,-3.3853492112

H,0,-5.1442682466,1.1911248397,-3.1629277612

O,0,-5.7417949318,0.9742726023,-0.7669013344

H,0,-6.6259942668,0.7731453564,-1.1175646362

C,0,-5.209527243,-1.3571273345,-1.0019164496

H,0,-6.1231028654,-1.5856858033,-1.5655931034

H,0,-4.4469796627,-2.0787778781,-1.3147388091

C,0,-5.5192809856,-1.553524979,0.4143741877

N,0,-5.8014952739,-1.6992149853,1.5348417215

C,0,-3.2641741448,2.0134312579,-0.8458748006

H,0,-3.7778653047,2.4221324781,0.0331129643

H,0,-2.2271316921,2.3632614392,-0.8109839153

H,0,-3.7280186448,2.4481312342,-1.7381457481

H,0,1.3713508789,-0.3416788441,1.8718843147

H,0,0.6989496241,1.2816385678,1.7285146604

H,0,2.7383996533,2.1879393173,0.8004227812

H,0,3.1799912444,1.2758216127,2.2723549066

O,0,4.7545600127,-0.1188251039,0.8213445881

Cl,0,4.311119251,-0.755288756,-1.9887625435

H,0,1.4242988811,0.8833272765,-3.0531391665

H,0,2.0158716831,-0.7699296399,-3.1579307392

H,0,3.3492630574,1.3710462294,-1.3812552254

O,0,3.2907759012,2.9850454661,-1.6188185969

H,0,3.4317885281,3.2660602147,-2.5361454871

O,0,4.0638860186,-4.5396332368,-0.759298165

H,0,4.5495013693,-4.7867960643,-1.5609255139

H,0,4.6558924805,-3.9342693302,-0.2538992084

O,0,5.6852628548,-2.8342301868,0.7472118744

H,0,5.3793579867,-2.9766100166,1.6579628628

H,0,5.4358151667,-1.9120020476,0.5421007322

O,0,3.1910335683,4.8015616077,0.3600588689

H,0,3.2343549032,4.1775186414,-0.4212603781

H,0,4.0549182646,4.7308772452,0.795259195

O,0,1.2892925373,4.0351869975,2.2756535092

H,0,1.3312585492,4.7305469306,2.9496974753

H,0,1.9392645336,4.2976679576,1.5800332894

O,0,0.649585993,3.3693639956,-1.0341032411

H,0,0.5454043817,3.1353395438,-0.0969618367

H,0,1.5994961901,3.1694184842,-1.2636760645

O,0,5.7076036846,2.8199488752,-0.3776933061

H,0,6.0269110151,1.9088030237,-0.4646210583

H,0,4.8488203334,2.8592454731,-0.8815086779

O,0,1.7189137803,-3.2101024761,-1.3451278332

H,0,2.554071885,-3.7030272607,-1.1515938308

H,0,1.7752647816,-2.9393428873,-2.2741470547

O,0,-0.7171489825,-2.6870404275,0.0098441721

H,0,-0.516211817,-2.4946019867,0.9458962521

H,0,0.138126242,-2.9089624373,-0.4191113881

H,0,-3.7566123429,-3.9668435054,-0.2627078679

O,0,-3.0588092626,-4.1634992037,0.3800019498

H,0,-2.2518632133,-3.6914002606,0.0734854131

O,0,-1.4436429368,4.0717833926,1.4264866892

H,0,-1.9900900453,3.4711016213,1.9760225671

H,0,-0.524598989,4.0174915795,1.7634496657

O,0,-3.1244146326,2.38472976,3.0197681542

H,0,-3.9887475264,2.1318182646,2.6213053109

H,0,-2.6405520848,1.5515555574,3.1337735457

O,0,0.0621695231,-2.450505219,2.9494116895

H,0,0.0909205245,-1.5440421101,3.2942672956

H,0,0.9174979367,-2.5875499825,2.4759708155

O,0,2.425373966,-2.7329305411,1.4867752143

H,0,2.3203377822,-2.2906380644,0.6249656276

H,0,3.0240579225,-2.177364863,2.0357809466

H,0,-3.05515888,-3.1650067894,2.0307127919

O,0,-2.8063811949,-2.6952459774,2.853285539

H,0,-1.829101207,-2.6878715078,2.8536626867

H,0,-5.6512845291,1.3880727378,1.054504508

O,0,-5.6567875346,1.5817936595,2.0178711092

H,0,-5.761861588,0.7138732191,2.4410623675

O,0,4.0325750209,-1.3154559009,3.3381130367

H,0,3.4312034195,-0.7373099578,3.8342347388

H,0,4.5115766292,-0.7280001428,2.7233505926

C4-Cl product complex

SCF Done: E(RMN15) = -2739.12818196

Sum of electronic and thermal Free Energies= -2738.410653

C,0,1.9367259978,1.1770909263,0.3217731486

C,0,2.2992559275,0.1854063479,-0.7433097546

C,0,3.5945635223,0.0912833801,-1.1816386289

C,0,4.1027922027,-1.0653029931,-1.9081320579

C,0,3.0822627885,-2.0962298531,-2.3044845125

C,0,1.6972346148,-1.4886634615,-2.5077402189

C,0,1.2870378953,-0.7282437663,-1.2695833823

C,0,0.0886602476,-0.8831287723,-0.6323606407

C,0,-0.1792808882,-0.1430267846,0.6698137707

C,0,0.4540079514,1.2443602883,0.6363731844

H,0,-0.0744336124,1.857528562,-0.105511218

H,0,0.3224360016,1.7288453531,1.6115568185

H,0,0.3004871849,-0.7126921127,1.4870974705

C,0,-1.6666935811,-0.0462728088,0.9735433643

H,0,-2.0979217367,0.6323168279,0.2181080675

C,0,-2.396548598,-1.389566091,0.8301094951

C,0,-2.3724431115,-1.7280227552,-0.6601783087

C,0,-0.9325763337,-1.8649209071,-1.1767373598

H,0,-0.5627680976,-2.8763432255,-0.9559342348

H,0,-0.9504654008,-1.795394655,-2.2700982685

H,0,-2.915678823,-2.6551090932,-0.883843354

H,0,-2.8624674171,-0.9160049122,-1.2179177521

C,0,-3.774451102,-1.0521464121,1.4616962961

C,0,-3.4133602567,-0.151009752,2.6686029374

C,0,-2.0323082039,0.4728151509,2.3692228879

H,0,-1.2841127568,0.136658756,3.0997874495

H,0,-2.0535984873,1.5683500368,2.4054416428

H,0,-4.1898161901,0.6031933807,2.8365726939

H,0,-3.3769034591,-0.7869733048,3.5599469678

O,0,-4.4312974811,-2.2516926149,1.8725489777

H,0,-5.2619881208,-2.0337388527,2.3286578827

C,0,-4.7246650629,-0.2818551972,0.5157597415

H,0,-5.5437224103,0.145189891,1.109495294

H,0,-4.2095093068,0.5537791412,0.0251602465

C,0,-5.3347067571,-1.1369797355,-0.5019461433

N,0,-5.8449441101,-1.8292398452,-1.2877839822

C,0,-1.7263884292,-2.4956878012,1.6609946918

H,0,-2.2828618479,-3.4342158372,1.5739832209

H,0,-0.7017748777,-2.6877916495,1.3200845294

H,0,-1.6774192611,-2.2281589277,2.7240359583

H,0,1.7267077633,-0.7999436311,-3.3641671353

H,0,0.9917749493,-2.2854499414,-2.7472941963

H,0,3.050418601,-2.8365121007,-1.4883918538

H,0,3.4452891186,-2.6095443783,-3.1994856123

O,0,5.3141917431,-1.2368164052,-2.1219232328

Cl,0,4.7784904835,1.2859737761,-0.7178713372

H,0,2.4896039084,0.8720808456,1.2228949735

H,0,2.3287002916,2.1621805499,0.0491375475

H,0,3.7097926773,-0.9864426505,2.1263433891

O,0,4.4639327568,-0.4216189808,2.3941767949

H,0,5.1776353192,-0.6262153338,1.7552704864

H,0,4.1414967909,1.4095238442,2.5934699058

O,0,3.9909518677,2.3722914156,2.7320300163

H,0,4.2927352402,2.565919841,3.6320810047

O,0,-0.6452844099,4.4049175517,1.439722215

H,0,-1.0718139591,3.5687545853,1.6889471512

H,0,0.3214436475,4.2934984331,1.6070291114

O,0,2.1345044908,4.3054039972,1.6450792051

H,0,2.3701994232,4.362999108,0.69535376

H,0,2.6832630129,3.5819001853,2.0156425459

O,0,6.4730227439,-1.3614762534,0.5273439404

H,0,7.4290202535,-1.3932837053,0.6858032148

H,0,6.3477359951,-1.0955567448,-0.404896314

O,0,4.8164527672,-3.6206490734,0.5408591532

H,0,5.1050259634,-4.3102742876,1.1579189937

H,0,5.4913172711,-2.9063951828,0.5889621033

O,0,2.500208494,-2.3883306874,1.4402969973

H,0,2.1270868283,-1.954913854,0.6537536311

H,0,3.297269933,-2.875040042,1.1135195373

O,0,-2.6833363432,5.7527334904,0.0225617963

H,0,-1.9006853849,5.5192453214,0.5631870361

H,0,-2.3315932805,6.0426282775,-0.8334530658

O,0,-3.3050151765,2.985533068,-0.1030969441

H,0,-2.769113213,2.5640200927,-0.8031514535

H,0,-3.1155963645,3.9497418014,-0.1510524554

H,0,-6.3877965035,1.9108433887,-0.4387696682

O,0,-5.9153141258,2.5116252393,-1.035478633

H,0,-5.0576255161,2.7143363336,-0.5947429018

O,0,1.0666390767,-4.7204157353,0.109997201

H,0,0.0921197719,-4.7923519073,0.0571783536

H,0,1.2657345504,-3.9706425977,0.6987157037

O,0,-1.7289504489,-5.2630671321,-0.4346486299

H,0,-2.6391806137,-4.9883314675,-0.1817486935

H,0,-1.7901127018,-6.2016521853,-0.6677778457

O,0,-1.5778032572,1.8064843084,-2.3345651586

H,0,-1.1399840021,1.0202983044,-1.9671192889

H,0,-1.0143859582,2.5783860395,-2.0842052361

O,0,-0.2206673748,4.0896885003,-1.4544685818

H,0,-0.445559641,4.1568810384,-0.5027450786

H,0,0.7584544866,4.1415554286,-1.4830240397

H,0,-4.9011700602,1.4781621622,-2.3009475511

O,0,-4.288848625,1.0275948348,-2.9185530601

H,0,-3.396391766,1.3645398019,-2.7044279345

H,0,-4.4296280663,-3.7349485215,0.7561686001

O,0,-4.4210411003,-4.5330925761,0.182634557

H,0,-4.7865063419,-4.2338678347,-0.6654068958

O,0,2.612614374,4.4159911874,-1.2054727625

H,0,2.9620304943,5.2439325978,-1.5715914261

H,0,3.2028925324,3.7140859396,-1.5242828602

C4-C9 chlorohydrin formation TS

SCF Done: E(RMN15) = -2739.05851443

Sum of electronic and thermal Free Energies= -2738.336333

O,0,0.003999589,-0.0144879261,0.0038181338

C,0,-0.0015811129,-0.0128396491,2.2043639873

C,0,2.888006517,-0.0095528115,2.5859918073

C,0,2.0436178773,-1.2403486234,2.5867449455

C,0,0.6914473186,-1.2675408623,2.4148337144

C,0,0.7507471141,1.2604493131,2.4502241769

C,0,2.1892340541,1.1835266477,1.9498383541

H,0,2.193726045,1.0949340256,0.8559467756

H,0,2.7137278964,2.1080004203,2.2104373784

H,0,0.8050002927,1.2734668166,3.5592649441

C,0,-0.013065607,2.5024834564,2.011500137

H,0,-0.0514133005,2.4923565581,0.908856153

C,0,-1.4645984137,2.5075096657,2.5282485294

C,0,-2.1667614755,1.3253576983,1.8622995548

C,0,-1.4903576138,0.0099244359,2.2500614449

H,0,-1.7115480729,-0.2483019428,3.3004929031

H,0,-1.9029982525,-0.8207096513,1.6665325376

H,0,-3.2288390542,1.2617382842,2.1277132445

H,0,-2.0998962412,1.4295793757,0.7716532509

C,0,-1.918063924,3.9378594529,2.1301425507

C,0,-0.6573631538,4.8048400872,2.40038409

C,0,0.5545992956,3.8474548847,2.4725604997

H,0,0.9257705468,3.760207069,3.5022483364

H,0,1.3893263619,4.1832325925,1.850432118

H,0,-0.5373654895,5.5601545568,1.6164830623

H,0,-0.8069799477,5.3370053281,3.3459160526

O,0,-3.0273685739,4.334588957,2.9375447931

H,0,-3.2779365293,5.2492074445,2.7224279137

C,0,-2.3367973456,4.0932876996,0.6518755493

H,0,-2.4465150785,5.1632645726,0.4347733324

H,0,-1.5657471831,3.7032757747,-0.0206805337

C,0,-3.6170756084,3.4550850971,0.344684205

N,0,-4.6513416523,2.9721584682,0.1130480931

C,0,-1.5438181358,2.4008321796,4.0627827097

H,0,-2.5901296561,2.3770159297,4.3837408675

H,0,-1.0678857724,1.4911404656,4.4432464337

H,0,-1.0634025662,3.2553140106,4.5533033875

C,0,-0.0398494428,-2.5911329138,2.4588705961

C,0,0.5547595159,-3.5042199489,3.5347889682

C,0,2.023879222,-3.6928529135,3.3482576999

C,0,2.7921284695,-2.5357147938,2.7180699827

H,0,3.756483101,-2.4135998893,3.2196488448

Cl,0,3.1411935592,-3.0421968057,1.0183617671

O,0,2.6253288095,-4.7190036705,3.653230627

H,0,0.4339940624,-3.024168893,4.5209312204

H,0,0.0689913297,-4.4831217537,3.5725228407

H,0,0.0184385801,-3.081596192,1.4758897987

H,0,-1.1003990919,-2.4516168808,2.6815310448

H,0,3.1253460641,0.1988402285,3.6450381186

H,0,3.8455036058,-0.2312414155,2.0979511788

O,0,3.0449691585,-3.175633591,-2.1042633126

H,0,3.9291781956,-3.0132124114,-1.7401061587

H,0,3.1695065981,-4.8287013323,-1.426734576

O,0,3.2389719742,-5.7299986685,-1.008075853

H,0,2.4102963749,-5.8464837617,-0.50230244

O,0,5.0465972714,-3.6754439784,4.9502940997

H,0,5.5438480825,-4.1218371166,5.6537999476

H,0,4.4091835425,-4.3310402059,4.6083298639

O,0,2.7967042418,-2.1449362097,5.8703449658

H,0,2.2347841843,-2.8819049631,6.1577945622

H,0,3.6429358958,-2.5577156085,5.5941406135

O,0,0.7871036506,-0.0599256514,5.7335848445

H,0,-0.0881484967,-0.4882497117,5.624502544

H,0,1.4589054828,-0.7646859459,5.6562152227

O,0,4.5433901718,-6.0500732001,1.5575069678

H,0,4.1724459499,-5.8719651133,0.6648387919

H,0,3.8713190097,-5.7395991423,2.1897748956

O,0,2.2896090802,-0.7186580705,-1.330544618

H,0,2.5332585048,-1.6553527723,-1.6193391494

H,0,2.2265309852,-0.1962397876,-2.1447280834

H,0,-0.6182652939,-0.759794398,-0.188025156

H,0,0.8711123528,-0.3032790794,-0.3919663607

H,0,-1.0695186225,2.6093748529,-1.9020914477

O,0,-1.2791864544,1.6635959196,-1.8955516391

H,0,-0.730902717,1.2567059532,-1.1899674347

O,0,6.3707490663,-3.9373222046,2.3578270694

H,0,5.7771228003,-4.6283204525,1.9963731859

H,0,6.0136209267,-3.744044575,3.2466297131

O,0,-1.8221059045,-1.2522213471,5.5707683875

H,0,-2.5893170295,-0.7321879161,5.2455457055

H,0,-1.7248932641,-1.9876305788,4.944094038

O,0,-4.170106978,-0.0478254661,4.4150460747

H,0,-4.4200523826,0.8633996399,4.1420628661

H,0,-4.1190616079,-0.5490522464,3.585446184

O,0,-1.583356906,-2.2579913842,-0.6197439109

H,0,-1.9272691821,-2.6378099232,0.2045381744

H,0,-0.7868477857,-2.8009221697,-0.856993312

O,0,0.6089791823,-3.7938808332,-1.1588408258

H,0,1.5304337815,-3.5135324522,-1.4265429879

H,0,0.7185958216,-4.3848259966,-0.3894459273

H,0,-2.8676731446,0.6549790718,-1.3657977682

O,0,-3.43919382,-0.0979174068,-1.1158600137

H,0,-2.828380095,-0.8571260698,-1.0455127538

H,0,-4.4117513793,3.1174004663,3.2429833784

O,0,-5.1363171355,2.475157492,3.4103384187

H,0,-5.430382963,2.202562396,2.5257283087

O,0,1.0168493474,-5.8548182533,0.8945874629

H,0,0.4328820462,-6.6292740365,0.8763356467

H,0,1.530156457,-5.9287894013,1.7162403854

C4-C9 chlorohydrin

SCF Done: E(RMN15) = -2739.12351858

Sum of electronic and thermal Free Energies=   -2738.400483

C,0,-1.0236830924,-1.10068036,-2.2090896683

C,0,-1.4511559973,-0.8297796686,-0.7901028336

C,0,-0.6452287935,-0.4003013249,0.1988568697

C,0,0.7952880246,-0.0132583712,-0.1193948732

C,0,1.3064560665,-0.8249694803,-1.3291890524

C,0,0.3892149117,-0.6136837971,-2.5296751726

H,0,0.3839425106,0.452060306,-2.7986493533

H,0,0.7778631801,-1.159150932,-3.3973357326

H,0,1.2378170879,-1.8848744372,-1.0423037793

C,0,2.7647840366,-0.4894552371,-1.6151447685

H,0,2.8125557287,0.5901970333,-1.8321142221

C,0,3.6836731875,-0.7394674583,-0.4037415205

C,0,3.1940436714,0.1532044478,0.7357009763

C,0,1.7368131961,-0.1645255478,1.0800930759

H,0,1.6352139885,-1.1866074365,1.4648474836

C,0,5.0702608897,-0.4163035871,-1.0177431848

C,0,4.9699835146,-0.9878468632,-2.4610260914

C,0,3.4697433268,-1.2186494515,-2.7608674161

H,0,3.2201916995,-2.2878525427,-2.7369906613

H,0,3.180315855,-0.8361814147,-3.744535616

H,0,5.4246220925,-0.2951794627,-3.1779760381

H,0,5.5431047371,-1.9206402059,-2.5011600753

C,0,3.6929596219,-2.2186125865,0.0275241305

H,0,4.2762613109,-2.3392534964,0.9467281414

H,0,2.6851132611,-2.5960034243,0.2267456291

H,0,4.1438171497,-2.8617271958,-0.7369673408

C,0,-1.2081459232,-0.17030006,1.5836736813

C,0,-2.2662745513,-1.2190203951,1.9326228156

C,0,-3.3287200702,-1.3078183295,0.8899206582

C,0,-2.9216446877,-0.9769037841,-0.5406403532

H,0,-3.3974549069,-1.6863760864,-1.2247940796

O,0,-4.4900695695,-1.6297373879,1.1394690567

H,0,-1.788889985,-2.2141701008,1.9501727622

H,0,-2.7327948301,-1.0520691646,2.9080419116

H,0,-1.6459981403,0.8372785994,1.6495000093
H,0,-0.4244249205,-0.2158387835,2.344317172
H,0,-1.0922323188,-2.1901229537,-2.3660168475
H,0,-1.7572475029,-0.6516702178,-2.8942561169
Cl,0,-3.7147690855,0.6318844811,-0.8866042252
O,0,-3.4786816692,3.8417862714,-1.2192822889
H,0,-3.4311613983,4.7798549402,-1.4589037228
H,0,-5.0396805944,3.2058676447,-0.1892345082
O,0,-5.7367143064,2.9466663886,0.4453539374
H,0,-5.2598695516,2.599113299,1.2279549477
O,0,-5.2684671137,-3.3634654497,-1.0358455062
H,0,-5.9187491938,-4.0685152813,-0.8898302871
O,0,-2.6769998353,-4.2171338835,-0.0309887371
H,0,-2.9190554054,-4.2380019098,0.9085492662
O,0,0.200839836,-3.9473541562,0.3082136489
H,0,0.4006128954,-3.600082302,1.2030219883
H,0,-0.76549543,-3.8789050136,0.184408726
H,0,-5.2919207431,-2.8009151189,-0.236126725
H,0,-3.4990989274,-3.9632324837,-0.500694259
O,0,-6.8591388493,0.2625382586,0.3451081226
H,0,-6.483176115,1.1652319101,0.2627479062
H,0,-6.1508125423,-0.2783508095,0.7365488238
H,0,3.8174052135,0.0459009957,1.6322483233
O,0,-1.208574918,2.5764442789,-2.2449593737
H,0,-2.7006898033,3.4031620481,-1.6399906917
H,0,-0.5449424162,3.1796390085,-2.6172782833
O,0,0.7682715975,1.3813931673,-0.5076032367
H,0,0.6079060449,1.9412620859,0.2916701471

H,0,-0.7095862779,2.0248778085,-1.6043598556

H,0,2.8346748638,2.7833289843,-2.6941097921

O,0,2.0722750784,3.1559915918,-2.2241220218

H,0,1.6458686565,2.4016638174,-1.759705747

O,0,-6.6594949484,-1.0696037735,-2.2303370168

H,0,-6.7186888745,-0.550962972,-1.4007620433

H,0,-6.1736105954,-1.8798740064,-1.9822302588

O,0,0.8929224255,-3.1019254495,2.9742522527

H,0,1.7935953372,-2.734371073,3.1048081124

H,0,0.289414495,-2.3575318365,3.132119988

O,0,3.3724939554,-1.7697743745,3.6599185752

H,0,4.2424756618,-1.6331781725,3.2233031633

H,0,2.9622078454,-0.8899852784,3.6724095268

H,0,3.2430068334,1.2070938903,0.424884505

H,0,1.414507455,0.5094110097,1.8844132846

O,0,6.0966274256,-1.0559877905,-0.2549576274

H,0,6.9624134936,-0.8964105555,-0.6673909409

C,0,5.4038523394,1.0937649392,-1.0734131517

H,0,6.2711054172,1.2375162896,-1.7301428686

H,0,4.5746506089,1.6774420573,-1.4936700533

C,0,5.7600590318,1.6399752531,0.2365990559

N,0,6.0737540887,2.0635972462,1.2754142136

O,0,0.2508590053,3.0227858135,1.6914468578

H,0,0.3513783793,2.468115787,2.481543474

H,0,-0.7211903513,3.1768635848,1.5849776673

O,0,-2.4691100454,3.4238961173,1.4034792017

H,0,-2.8268689271,3.5954320437,0.5041584859

H,0,-3.0167777982,2.7083706136,1.7813203459

H,0,2.6273087311,3.7586513124,-0.4618500852

O,0,2.7675548384,3.9765767673,0.482149662

H,0,1.9399247042,3.7107190328,0.9270360586

H,0,6.0098027361,-1.0592423801,1.6187157406

O,0,5.9809898398,-1.0902393726,2.6003346315

H,0,6.0118012843,-0.1607400441,2.8786429345

O,0,-4.3899571137,1.5470553598,2.5642015353

H,0,-4.6510074892,1.7809816084,3.4690283229

H,0,-4.6903715582,0.6329409398,2.4273402985

TS for epoxide formation

SCF Done: E(RMN15) = -2739.10019818

Sum of electronic and thermal Free Energies= -2738.381623

C,0,0.0118397843,-0.0868045093,-0.0019119667

C,0,-0.0008718365,-0.0725008275,1.5006967224

C,0,1.2682468862,-0.0727712008,2.2466153154

C,0,2.5205975733,0.2032303457,1.5095553654

C,0,2.5143167896,-0.0039000636,-0.0053068454

C,0,1.2407066985,0.5576344818,-0.6335669855

H,0,1.2094311131,1.650144351,-0.5251279395

H,0,1.2456322539,0.340349396,-1.7069354595

H,0,2.5177336626,-1.0918368629,-0.1670667434

C,0,3.7842801566,0.6004028225,-0.5953472312

H,0,3.760871955,1.6798635138,-0.3669610339

C,0,5.0714342884,0.0420103478,0.039850378

C,0,5.0484415522,0.4333516615,1.5189009062

C,0,3.8182575758,-0.1690201753,2.2007964031

H,0,3.8641142283,-1.2644704205,2.2010447542

H,0,3.7885305141,0.1453350823,3.2497042073

H,0,5.9509493219,0.1045808609,2.0474734116

H,0,4.9877875392,1.5290293089,1.6131414285

C,0,6.1554299992,0.6971108663,-0.8573879605

C,0,5.5220634314,0.6679062232,-2.2768443688

C,0,4.0045341204,0.4314698633,-2.0997465532

H,0,3.725356819,-0.5863188191,-2.4032746306

H,0,3.401757447,1.1271539899,-2.6915320639

H,0,5.7295813503,1.6037677719,-2.8069274896

H,0,5.9947099511,-0.1403728381,-2.845301663

O,0,7.359871299,-0.069193845,-0.7897168823

H,0,8.0226934917,0.3074365589,-1.393102571

C,0,6.5074666004,2.1537604905,-0.4751342867

H,0,7.0900146355,2.5977098659,-1.2924045875

H,0,5.6086169461,2.768521565,-0.3410398906

C,0,7.3306175931,2.2317812973,0.7321143678

N,0,8.0115218262,2.2827193126,1.6757930491

C,0,5.1836047837,-1.4854084317,-0.1249022573

H,0,6.0514440941,-1.8636442171,0.4259003344

H,0,4.3009078079,-2.0085757047,0.2558145369

H,0,5.30725908,-1.7682186986,-1.1764830042

O,0,2.0328678974,1.5467985999,1.8341524563

H,0,2.5165419895,1.9681830484,2.6460861693

C,0,1.2958333667,-0.3031278517,3.7285365411

C,0,-0.0094332074,-0.8330756887,4.3207688053

C,0,-1.2381081657,-0.327946929,3.63943085

C,0,-1.1630656666,-0.1150968537,2.1883145305

H,0,-2.1102021312,-0.0341674857,1.6563200951

Cl,0,-1.3889480917,3.2729166302,1.0040786519

O,0,-2.3173656888,-0.1913964659,4.2353088192

H,0,-0.0432065517,-1.9253034923,4.1853726381

H,0,-0.0698902259,-0.6300592717,5.3924143518

H,0,1.5601406668,0.6468800843,4.2113980083

H,0,2.1080750378,-1.0035571293,3.9490153651

H,0,-0.0069152984,-1.1500437795,-0.2899691985

H,0,-0.9192963494,0.3607188688,-0.3652670566

O,0,0.7527969104,6.5843915476,3.2508533884

H,0,0.9673725944,7.5248640935,3.3404251944

H,0,0.7098171283,6.3788187686,2.2825927231

H,0,-0.8930595721,5.9685911752,3.9062565066

O,0,-1.7145104465,5.5417486548,4.231411232

H,0,-1.5126711865,4.5903824148,4.2428425176

O,0,-3.7261037906,-2.0474076934,2.3312254707

H,0,-4.4554503692,-2.6477536182,2.553125315

H,0,-3.6350691914,-1.4428987985,3.0907987435

O,0,-1.2480830192,-3.5221969897,2.3428382413

H,0,-1.1920552198,-3.8284182411,3.2613447054

H,0,-2.0660919395,-2.9805483078,2.3004208887

O,0,1.4451199269,-2.6194810617,1.7566363066

H,0,2.0835760529,-2.9411981392,2.428613815

H,0,0.5521238343,-2.9254059768,2.0219624508

O,0,-3.749230197,2.006225968,2.804325376

H,0,-3.0190096055,2.4073044217,2.2843672151

H,0,-3.3380906938,1.2948217395,3.3333641822

O,0,0.5065190676,5.7989546469,0.6383080059

H,0,1.3103477894,5.275690913,0.4257134551

H,0,-0.1966047708,5.1189366312,0.7305608161

H,0,2.7821269493,3.7827531112,-0.7503920943

O,0,2.6505516771,4.0202364891,0.1821344593

H,0,2.3234815624,3.2077476709,0.6147260364

O,0,-4.5227168014,0.2074260771,0.6521043303

H,0,-4.3290548215,0.880761608,1.3370745913

H,0,-4.2976616945,-0.6409371499,1.0827625683

O,0,3.4970364892,-3.4719310655,3.6005750157

H,0,4.3906172886,-3.1269806655,3.3965343212

H,0,3.2519714307,-3.0650833564,4.446486679

O,0,6.1880157149,-2.2954612702,3.3628481255

H,0,6.837740525,-1.9753619551,2.6990879733

H,0,5.9155984218,-1.4955425993,3.8404311151

O,0,3.2451790139,2.7326756897,3.7544429141

H,0,3.5743535916,2.1288976417,4.4404637109

H,0,2.6084543589,3.3674583604,4.1949502821

O,0,1.4212154254,4.358191313,4.8346777685

H,0,1.298060582,5.1806475363,4.3112566355

H,0,0.6348025707,3.8029318374,4.638772663

H,0,4.2305971543,4.2933341717,1.2486914197

O,0,4.8825468832,4.3220147582,1.9776076555

H,0,4.4718139832,3.7788079378,2.6790015726

H,0,7.9564433659,-0.707963899,0.8812810184

O,0,8.2674135296,-1.0938943163,1.7291803941

H,0,8.4473288332,-0.3300104462,2.3005579529

O,0,-0.5956751549,2.607633812,3.9897505343

H,0,-1.4291993532,2.3064598661,4.3876477541

H,0,-0.784431439,2.7512597072,3.0324151153

C9-C10 epoxide

SCF Done: E(RMN15) = -2739.12676386

Sum of electronic and thermal Free Energies= -2738.407801

C,0,-0.8955934995,-0.6054959892,-2.4703897951

C,0,-1.345848108,-0.8463910036,-1.0606451457

C,0,-0.5473748054,-0.2341008753,0.0330464985

C,0,0.9290755572,-0.2938590711,-0.1447650478

C,0,1.461909951,-0.884063075,-1.4516894395

C,0,0.6252131924,-0.409540355,-2.643284959

H,0,0.851022089,0.6526851461,-2.8084931178

H,0,0.9569075186,-0.9459061829,-3.5386001276

H,0,1.3946008088,-1.9790913234,-1.3730139412

C,0,2.9219223644,-0.4740501124,-1.6370139948

H,0,2.9184623097,0.6231296845,-1.7667221607

C,0,3.8146174603,-0.7738440916,-0.4199628212

C,0,3.2814657941,0.0449218476,0.7568642553

C,0,1.8314467731,-0.3434947899,1.0712860484

H,0,1.7854213034,-1.3643638959,1.479093533

C,0,5.2122759955,-0.3908498574,-0.976260545

C,0,5.1656369734,-0.8799462075,-2.4505370488

C,0,3.6785834675,-1.0900361524,-2.8160270491

H,0,3.4376765132,-2.1590312364,-2.8904858155

H,0,3.4141996078,-0.6265874528,-3.7713749642

H,0,5.6506958338,-0.152201894,-3.1099727863

H,0,5.7360222057,-1.8126612232,-2.5188696328

C,0,3.8354930965,-2.274260232,-0.0752379418

H,0,4.3730697954,-2.4398036579,0.8661888615

H,0,2.8257368789,-2.6778687426,0.04329143

H,0,4.337700214,-2.8602826036,-0.8534960204

C,0,-1.2428985459,-0.1073202393,1.366456104

C,0,-2.0452804455,-1.3603303203,1.7109840993

C,0,-2.9496763806,-1.7455435966,0.5758085806

C,0,-2.4482733754,-1.5833490499,-0.7799629855

H,0,-3.032925128,-2.0233781396,-1.587035537

O,0,-4.0882400698,-2.2126193001,0.7923641964

H,0,-1.3682753499,-2.211185025,1.8825185246

H,0,-2.6455051004,-1.218822459,2.6148350405

H,0,-1.9267003132,0.7457350594,1.2702080046

H,0,-0.5358963082,0.1262835841,2.1651644862

H,0,-1.2701847051,-1.4243675626,-3.0936055465

H,0,-1.4180547468,0.3029564791,-2.8082178349

Cl,0,-3.4409728052,1.9910165511,-1.1036442342

O,0,-3.9521794661,5.1992949449,1.7192688521

H,0,-3.8313437594,6.0128933432,2.2337012265

H,0,-5.5692497075,4.1526785203,1.5217517321

O,0,-6.2590939781,3.4693455314,1.4159676436

H,0,-5.8036615807,2.6256254122,1.594558145

O,0,-4.3587783708,-4.313142831,-1.0919131715

H,0,-5.0817945089,-4.9428392786,-0.9449789791

O,0,-2.2049969777,-4.9271423595,0.781057995

H,0,-2.569436862,-4.4842297614,1.5639127833

O,0,0.3494701255,-3.7165169637,0.3058618641

H,0,0.9356474999,-3.8419806381,1.0816143827

H,0,-0.5105348925,-4.1358036942,0.5174782257

H,0,-4.5242487877,-3.5603430631,-0.4839874205

H,0,-2.8658339279,-4.7768975015,0.0724849839

O,0,-5.5633789856,-0.5119361883,-1.2432357577

H,0,-4.892875652,0.200381866,-1.2903658937

H,0,-5.2551490152,-1.1063391395,-0.5332832327

H,0,3.8978760877,-0.0864570899,1.6550217705

O,0,-2.7855935462,5.1038824673,-0.7361444104

H,0,-3.5334149161,5.3308440069,0.8282872646

H,0,-1.8196770455,5.0999620846,-0.6344969711

O,0,0.198611098,0.9390659587,-0.353195878

H,0,-0.2265607423,2.419168339,0.6549402409

H,0,-3.0292658996,4.165200747,-0.8958565371

H,0,-0.5462142135,2.4736849332,-3.2320573791

O,0,-0.7321724052,3.0939654548,-2.5084392033

H,0,-1.4565485923,2.6751711434,-1.9970257377

O,0,-5.0447932388,-2.4967866402,-3.2824225979

H,0,-5.2320581751,-1.7459100832,-2.6809880024

H,0,-4.7801673846,-3.2201789067,-2.6802779603

O,0,2.2288610195,-4.0886384882,2.4252260344

H,0,2.7403818761,-3.34196416,2.8146229262

H,0,1.8666758294,-4.5821341087,3.1763512209

O,0,3.6443416434,-1.9881750224,3.6483742601

H,0,4.5015406729,-1.7150639205,3.2459399776

H,0,3.0607082123,-1.2178075218,3.563056806

H,0,3.3021741267,1.1151369354,0.5003409765

H,0,1.437934966,0.3279962201,1.8406090058

O,0,6.2271975093,-1.056992566,-0.2216418775

H,0,7.1012450661,-0.8684275401,-0.603737751

C,0,5.5238165819,1.1247257089,-0.943025315

H,0,6.4087568079,1.3117558777,-1.5644109096

H,0,4.7012135753,1.7190435261,-1.358928217

C,0,5.835168507,1.6070974132,0.4025238287

N,0,6.112237417,1.9825313517,1.4697672295

O,0,-0.5896437775,3.2192430089,1.0957246662

H,0,0.1396392777,3.615036722,1.5990011388

H,0,-1.8971064629,3.0397155728,1.9436657087

O,0,-2.7619361076,3.081104923,2.4908269372

H,0,-3.2499580269,3.9575172649,2.2464776009

H,0,-3.3677274597,2.2995748736,2.2817104456

H,0,0.9477648094,3.1911498616,-1.6714759976

O,0,1.8623064379,3.2681909657,-1.320791727

H,0,1.9804943113,2.4804137174,-0.7662895039

H,0,6.1420685333,-1.1175526877,1.6344031216

O,0,6.138211025,-1.1368814098,2.6173090457

H,0,6.1934944189,-0.2043494345,2.8823024305

O,0,-4.4673749777,1.1896639151,1.7788472689

H,0,-4.4602088108,0.2976832101,2.1636109827

H,0,-4.1995821982,1.1095055433,0.8363695379

β-trenbolone

Reactant complex

SCF Done: E(RMN15) = -2224.53643585

Sum of electronic and thermal Free Energies= -2223.981001

C,0,-0.5746593636,0.1798948222,1.8064515614

C,0,-0.926818951,0.4956061739,0.380311625

C,0,0.1261072028,0.5749301741,-0.6259870223

C,0,1.4435122775,0.4500294993,-0.256428352

C,0,1.8270716003,0.145117051,1.1881413731

C,0,0.714588406,-0.6181000724,1.8983906484

H,0,0.586732982,-1.6042174826,1.4238061143

H,0,0.9872337605,-0.7860772193,2.9462757254

H,0,1.9658165019,1.1027805876,1.7213169221

C,0,3.1459855077,-0.6144808079,1.1897097448

H,0,2.9905082638,-1.5279551892,0.5860662956

C,0,4.2652214888,0.1705833216,0.4919030118

C,0,3.8415930382,0.4385965817,-0.9182583034

C,0,2.5328618354,0.569274082,-1.2340589352

H,0,2.274249534,0.7933571619,-2.2651940098

C,0,5.4650017626,-0.760528411,0.70905489

C,0,5.2655220753,-1.2677107505,2.1570999637

C,0,3.7742126934,-1.0273190319,2.5200292403

H,0,3.6740679878,-0.2159310489,3.2538885542

H,0,3.2998451301,-1.9151285051,2.9491645361

H,0,5.5339638545,-2.3294180321,2.2013023094

H,0,5.9349703292,-0.7380082086,2.8427593859

O,0,5.3890529322,-1.827658631,-0.2435299301

H,0,6.0553486656,-2.5004711216,-0.0314842228

H,0,6.4194245288,-0.2339615017,0.5767696291

C,0,4.5979458993,1.5138954663,1.1730941893

H,0,5.3987722655,2.0158772259,0.6156629074

H,0,3.7294455287,2.1813656811,1.1893227301

H,0,4.9447400502,1.369753855,2.2033961251

C,0,-0.3556668589,0.7091699333,-2.0553784008

C,0,-1.568397004,1.6318977568,-2.1761593672

C,0,-2.6583257837,1.2209447318,-1.2321429806

C,0,-2.2424217131,0.7109940268,0.0589774281

H,0,-3.0114167734,0.5878822438,0.8196824313

O,0,-3.8632532663,1.3762739816,-1.5217964836

H,0,-1.2891430319,2.6600353568,-1.8945825029

H,0,-1.9592039605,1.65794626,-3.1970425534

H,0,-0.6405371105,-0.2959041741,-2.4128379797

H,0,0.429390896,1.0698661749,-2.7211060708

H,0,-0.4414943928,1.1402834269,2.3305598817

H,0,-1.420764003,-0.3280416442,2.2837456422

Cl,0,-2.5737561463,-2.049105786,-0.3965802191

O,0,-3.1137129317,-3.6362683188,-0.7212310675

H,0,-2.672605446,-4.2093910529,-0.0628102215

H,0,-5.003787979,-3.176410688,0.2718718365

O,0,-5.5124925944,-2.5566438719,0.8238679138

H,0,-6.3461363669,-3.0021750081,1.0398314792

O,0,-4.6029946658,1.7249265148,2.3060813852

H,0,-5.2457770794,2.1276568596,2.9101223845

O,0,-3.1827425191,3.7510018875,0.8504043064

H,0,-3.5100218684,3.6737299347,-0.0596192051

O,0,-0.277287291,3.7404358822,0.8813604843

H,0,0.1183389143,3.6928286119,-0.0147021922

H,0,-1.2478816787,3.6600505202,0.7762260821

H,0,-5.1253563015,1.2651887591,1.6033472553

H,0,-3.6358813445,3.0361493521,1.35120514

O,0,-5.7460731618,0.2693218953,0.2423519192

H,0,-5.6170558101,-0.694356862,0.3590244592

H,0,-5.0843160297,0.5731615529,-0.4197620575

H,0,4.6016139734,0.5771661811,-1.688742362
O,0,-1.1419186589,-3.6359591061,-3.0121470549
H,0,-1.9177539687,-3.843558789,-2.4604977124
H,0,-1.4093438387,-2.8915281782,-3.5739168088
O,0,0.855219161,-2.792537329,-1.209980694
H,0,0.6322112976,-1.8995292519,-0.897187282
H,0,0.1569826607,-3.0418809706,-1.8579693955
H,0,4.0769484724,-2.4155286391,-1.487122995
O,0,3.5163026095,-2.827880327,-2.174322293
H,0,2.5969516631,-2.7859383,-1.833224144
O,0,-4.0538795065,-1.1478448,2.9368680915
H,0,-4.5238583206,-1.5956313089,2.2058540769
H,0,-4.163992522,-0.1900401676,2.7826431314
O,0,1.1136788046,3.7583335211,-1.6317545284
H,0,2.075808544,3.8811258297,-1.46496551
H,0,0.8235881633,4.548479162,-2.1124057827
O,0,3.8780789144,3.9866055885,-1.1714498109
H,0,4.0133524605,4.0887571545,-0.2157901507
H,0,4.1707136053,3.0813728034,-1.3700252091

Chlorination TS at C4

SCF Done: E(RMN15) = -2224.52471717

Sum of electronic and thermal Free Energies= -2223.966353

C,0,-0.6713934817,0.2311784953,1.7461745287
C,0,-0.971102422,0.428671628,0.2976683936
C,0,0.052890299,0.4620623695,-0.6902061115
C,0,1.3833122261,0.3555773784,-0.2934862529
C,0,1.744939141,0.1806361297,1.1717034752

C,0,0.6345376134,-0.5157911858,1.9485870469

H,0,0.532382838,-1.5531132398,1.5938533268

H,0,0.8950340274,-0.552891698,3.0114296418

H,0,1.8636124807,1.1953108281,1.5956714895

C,0,3.0721095856,-0.5564833797,1.2610974773

H,0,2.9345918153,-1.5229813513,0.742664504

C,0,4.1861325538,0.178855116,0.5056662964

C,0,3.7744579907,0.3182639959,-0.9212500445

C,0,2.4639940261,0.4075275304,-1.2651263808

H,0,2.2145061553,0.5367774924,-2.3134449896

C,0,5.3935865659,-0.716282575,0.8098118652

C,0,5.1900189411,-1.0886178926,2.2973345456

C,0,3.6923054135,-0.8374907453,2.6282181482

H,0,3.5751180639,0.0343419421,3.2855553828

H,0,3.2256920005,-1.6904185857,3.1298339149

H,0,5.4735773238,-2.1372122017,2.4418060865

H,0,5.8460605908,-0.4862037239,2.9339231288

O,0,5.3197899289,-1.8633952498,-0.0433364804

H,0,5.9962088319,-2.5084593423,0.2177626447

H,0,6.3441646448,-0.1973473721,0.6313826283

C,0,4.4928341411,1.5874308744,1.0576751693

H,0,5.3131378913,2.0328916115,0.4816999526

H,0,3.6233627988,2.249865933,0.9789066652

H,0,4.8036544222,1.5435514919,2.1078657783

C,0,-0.4055571068,0.5656635563,-2.1299091602

C,0,-1.5978907603,1.5131237294,-2.2768055227

C,0,-2.7169974603,1.1523494196,-1.3506937738

C,0,-2.3354772558,0.5290545496,-0.0737951184

H,0,-3.0649220687,0.6198804546,0.7281949183

O,0,-3.9038672902,1.4016354825,-1.6077537599

H,0,-1.2948303031,2.5339084327,-1.9890062599

H,0,-1.9687598081,1.5528277371,-3.3043443766

H,0,-0.6893486095,-0.4393367469,-2.4818473983

H,0,0.3926971719,0.9163055858,-2.7843005272

H,0,-0.6050160689,1.2393286971,2.1918986197

H,0,-1.5234558292,-0.2654516554,2.2253582878

Cl,0,-2.6843947737,-1.5849526457,-0.4891491258

O,0,-3.2604443164,-3.4638302128,-0.8404310507

H,0,-2.7659741115,-3.964351821,-0.1655870099

H,0,-4.7972442565,-2.9847899279,0.0701320428

O,0,-5.4618895482,-2.5180583579,0.6294978944

H,0,-6.2200864478,-3.1124579321,0.7317781449

O,0,-4.4540828303,1.6498710959,2.3421363398

H,0,-5.0414713194,2.0687519501,2.9900764378

O,0,-2.8230446989,3.5672698441,0.968858777

H,0,-3.2106248913,3.6236527131,0.0813101029

O,0,0.0676715085,3.6364570407,0.780262022

H,0,0.3938729641,3.5402761383,-0.1399919551

H,0,-0.9027380716,3.50679848,0.7611907912

H,0,-5.0362854404,1.2489929732,1.6512595002

H,0,-3.358631946,2.8940007755,1.4450604268

O,0,-5.7784991803,0.3236259587,0.2849068014

H,0,-5.6649871545,-0.6495630985,0.3374676715

H,0,-5.1895411418,0.6393875033,-0.4329381313

H,0,4.539593329,0.3987992433,-1.6948052132

O,0,-1.3860071731,-3.4970637343,-2.9117568628

H,0,-2.1249943191,-3.5437833544,-2.2578268418

H,0,-1.5246561297,-2.6786202616,-3.413585014

O,0,0.6592766825,-2.9978964957,-1.0481826289

H,0,0.3986318455,-2.1816376644,-0.5922106245

H,0,-0.0226542207,-3.1493949184,-1.7444565729

H,0,3.938101024,-2.5769437677,-1.1748682899

O,0,3.3909777516,-3.0418126789,-1.8381667411

H,0,2.4608182174,-2.9648212619,-1.5367903685

O,0,-4.0825679274,-1.270774108,2.8782399

H,0,-4.5367066327,-1.6709863862,2.1090935496

H,0,-4.1123164119,-0.3053758251,2.7366181444

O,0,1.2393938913,3.5180919706,-1.8225102058

H,0,2.2154666088,3.6250932926,-1.7524518572

H,0,0.9194319166,4.3027246758,-2.2933665866

O,0,4.0319762476,3.7760086345,-1.6280657277

H,0,4.2415625159,3.8797461551,-0.6859477447

H,0,4.3858464219,2.9045314911,-1.869997754

Cationic intermediate

SCF Done: E(RMN15) = -2224.56020314

Sum of electronic and thermal Free Energies= -2224.003733

C,0,-0.961675293,-0.5922298809,1.1353470254

C,0,-1.1291913425,0.1640408157,-0.133276678

C,0,-0.0919767929,0.6810804866,-0.875879222

C,0,1.2485679128,0.4394571839,-0.4292129792

C,0,1.4845400049,-0.2473051691,0.8928142856

C,0,0.3946298774,-1.2718833419,1.1977259978

H,0,0.4390550684,-2.0881714742,0.4610803794

H,0,0.5661127688,-1.7015158348,2.1902175014
H,0,1.3985900299,0.555184146,1.6520540886
C,0,2.8883663285,-0.8226376149,0.9345644149
H,0,2.9476967072,-1.5876150401,0.1411227522
C,0,3.9367931595,0.2429532355,0.5893057853
C,0,3.6497323034,0.7482545682,-0.7756835319
C,0,2.3588312001,0.8478951821,-1.2279887912
H,0,2.1832883862,1.2779850027,-2.20845457
C,0,5.2416480102,-0.5321016993,0.7992586063
C,0,4.9581249008,-1.3496726763,2.0787458683
C,0,3.4130438572,-1.4328445758,2.2330996795
H,0,3.07239959,-0.8441760014,3.0946905668
H,0,3.0612015377,-2.4581568786,2.3775174516
H,0,5.4196847252,-2.3382373776,1.9792710316
H,0,5.4170893466,-0.8640375673,2.9458199042
O,0,5.4324651758,-1.3731365189,-0.3416520091
H,0,6.167508979,-1.9844706962,-0.1748211619
H,0,6.1051852722,0.1361207891,0.9051975752
C,0,3.934480392,1.4638052739,1.5413743816
H,0,4.7684848929,2.1272101012,1.2858823446
H,0,3.004076347,2.0390596602,1.4691882006
H,0,4.0668816231,1.1399225372,2.5796937175
C,0,-0.4111370598,1.4161775539,-2.1606414538
C,0,-1.6688336857,2.2752995127,-2.0080740843
C,0,-2.8434445465,1.5008255706,-1.5000431118
C,0,-2.5325832337,0.2668070528,-0.6563423283
H,0,-3.2790670857,0.1557170559,0.1372196086
O,0,-4.0104803362,1.7919352967,-1.7188864197

H,0,-1.4752648859,3.0567622395,-1.2559462412

H,0,-1.9454751065,2.7749380841,-2.9401106472

H,0,-0.5375302648,0.6908375922,-2.9777673726

H,0,0.4071190679,2.0780562053,-2.4469140536

H,0,-1.0662120222,0.1411986825,1.9560257285

H,0,-1.7922228159,-1.2964494357,1.2491805298

Cl,0,-2.7351903213,-1.1608982335,-1.7599235491

O,0,-2.1133288635,-3.8720364167,1.1312486015

H,0,-1.484017261,-3.5492435503,1.7948883916

H,0,-2.9405686078,-3.2799862637,1.2085947178

O,0,-4.2502989177,-2.3867084119,1.34951652

H,0,-4.8768844754,-2.9474652245,1.8328816656

O,0,-5.296350993,1.6913443978,1.2024871332

H,0,-6.0194016604,1.8847715405,1.8180011962

O,0,-2.6494950387,2.5952418481,1.2890970992

H,0,-2.6581373071,3.4028784759,0.751869576

O,0,0.0916935824,2.7485810487,2.1837951923

H,0,0.5092120483,3.0582497474,1.3513349131

H,0,-0.8384790876,2.5611575402,1.9509020591

H,0,-5.524180066,0.843201385,0.7392022254

H,0,-3.5805879668,2.2782654624,1.308729196

O,0,-5.7799140825,-0.732098535,0.0331443029

H,0,-5.1663625836,-1.4052507663,0.4947147969

H,0,-5.5359970026,-0.7127774272,-0.9051218927

H,0,4.4646614571,1.1068433191,-1.4060016357

O,0,-0.6991669471,-4.1487180927,-1.2161893343

H,0,-1.2221470726,-3.9859997833,-0.3912451751

H,0,-1.3520392338,-4.3296871725,-1.9096088043

O,0,0.9707969265,-2.2362946805,-2.3467370279

H,0,0.4402418646,-1.4739712209,-2.6256809412

H,0,0.3546928016,-2.8568638558,-1.8890076435

H,0,4.2544697344,-1.9073423132,-1.7585344493

O,0,3.8013375422,-2.1838247215,-2.5788413288

H,0,2.8408407908,-2.1699369906,-2.3883682434

O,0,-3.8724809899,-0.2745294612,2.9655816352

H,0,-3.9929022974,-1.0762305861,2.373587739

H,0,-4.2946172046,0.4620448704,2.487050346

O,0,1.1196154752,3.8080082627,-0.2382846139

H,0,2.0688600063,3.8829926428,-0.4886840538

H,0,0.7771418909,4.7148896061,-0.2257482997

O,0,3.7946860997,4.0779797844,-1.0598696614

H,0,4.3933944542,3.6537354489,-0.4240232803

H,0,3.847556313,3.5346562816,-1.8626931423

TS for deprotonation at C4

SCF Done: E(RMN15) = -2224.55266174

Sum of electronic and thermal Free Energies=   -2223.996215

C,0,0.1844021905,-1.3097283612,-0.7958470506

C,0,0.6165544026,-0.37982316,0.2883797088

C,0,-0.2530671316,0.5320252204,0.8736126087

C,0,-1.6340742018,0.4679585337,0.5398173106

C,0,-2.1121271558,-0.3691987636,-0.619371017

C,0,-1.2915156033,-1.64908298,-0.7357872457

H,0,-1.499536455,-2.2892357754,0.1337766817

H,0,-1.5917026857,-2.1967079838,-1.6349478328

H,0,-1.9118667092,0.2454895721,-1.5214084068

C,0,-3.6006336733,-0.6425154086,-0.4918414426

H,0,-3.7294426048,-1.2533744236,0.4184949817

C,0,-4.4173792628,0.6325487186,-0.2606416041

C,0,-3.9003771902,1.3130681387,0.9541571591

C,0,-2.5815519319,1.229915915,1.3048642628

H,0,-2.2343628252,1.7744618789,2.1773194309

C,0,-5.8402516408,0.0656905752,-0.188994699

C,0,-5.8409473051,-1.0082670512,-1.3051291688

C,0,-4.3548455664,-1.3382231457,-1.6220590134

H,0,-4.0561271296,-0.9201056816,-2.5922325752

H,0,-4.1622594578,-2.4144297667,-1.6529485518

H,0,-6.386830254,-1.8872904308,-0.9451692889

H,0,-6.3668522729,-0.6409392844,-2.1920394732

O,0,-6.012119321,-0.5051684126,1.10953829

H,0,-6.8704407156,-0.9560593734,1.14783984

H,0,-6.5978330891,0.8431694377,-0.3517886339

C,0,-4.3608423155,1.6437843331,-1.4270352607

H,0,-4.9006187384,2.5565148043,-1.1509041733

H,0,-3.3318807924,1.9169369035,-1.6801940982

H,0,-4.8401369263,1.2259792714,-2.319388012

C,0,0.2567882138,1.5599116659,1.8636450742

C,0,1.7362823462,1.8783972134,1.6180305269

C,0,2.5301469088,0.610954033,1.6342778917

C,0,2.070155657,-0.3651638219,0.5963384736

H,0,2.5302915166,0.0242098554,-0.4232582502

O,0,3.4922515354,0.4032699741,2.3658453956

H,0,1.8432335317,2.3215110392,0.6151090208

H,0,2.1333432057,2.5761398259,2.3591070644

H,0,0.1018987524,1.2101053124,2.8930974885

H,0,-0.3054663284,2.4925442023,1.7520401676

H,0,0.4061032923,-0.7596226722,-1.7308801442

H,0,0.8141464517,-2.2009498817,-0.8126587135

Cl,0,2.770872993,-1.989498232,0.8569438339

O,0,2.6635457732,-3.9418098332,-2.0282870106

H,0,2.4422430014,-4.0908127008,-2.9597336577

H,0,3.2360695592,-3.1370632428,-2.0100202918

O,0,4.3665780283,-1.7180485431,-2.0703729022

H,0,4.8834419285,-1.7161720714,-2.8903786086

O,0,4.8098845169,2.4389043374,0.1156646884

H,0,5.651453719,2.9151118378,0.0452460592

O,0,2.8909854982,3.9768521612,-1.2037267099

H,0,3.3732685885,4.559222679,-1.8097989395

O,0,0.8573065446,2.1121727056,-2.0369541491

H,0,1.5345021091,1.376170938,-2.0060411097

H,0,1.4035892445,2.9001187709,-1.8356266379

H,0,5.0376637704,1.482170275,0.0510259446

H,0,3.5614291928,3.3890434709,-0.7829907127

O,0,5.5830100613,-0.2682286792,0.1035706732

H,0,5.2245951705,-0.8393470726,-0.6121258693

H,0,5.1412379374,-0.5505543312,0.9223625939

H,0,-4.5699418805,1.9389133476,1.5442123218

O,0,0.4746019479,-4.6847055161,-0.4210315637

H,0,1.2590590247,-4.3971036207,-0.9427453156

H,0,0.6069064573,-5.6280556615,-0.2414085737

O,0,-0.0110232692,-3.5114882813,2.0881633501

H,0,0.8351891617,-3.1680913408,2.4135111559

H,0,0.1691267083,-3.8595552165,1.1835767135

H,0,-2.6399989132,-1.4959735442,2.3972145155

O,0,-1.9674822464,-1.7228321018,3.0579518599

H,0,-1.3325226459,-2.3187801022,2.6032614479

O,0,2.955344987,0.4246947786,-1.90743347

H,0,3.7880609018,-0.8706616877,-2.0787732148

H,0,3.6072829107,1.1309543079,-1.7610907778

O,0,0.8280688142,5.0002165253,0.4900655188

H,0,0.0599609011,4.5270082366,0.1060683631

H,0,1.5942636092,4.7004189557,-0.0419448243

O,0,-1.2347348321,3.5437696006,-0.8434508255

H,0,-0.6678942348,2.809810362,-1.1684491887

H,0,-1.9256947593,3.1443534435,-0.2917734086

C4-Cl product complex

SCF Done: E(RMN15) = -2224.61955889

Sum of electronic and thermal Free Energies= -2224.055865

C,0,0.5270350403,-1.2121611722,-1.0397138071

C,0,0.8882249418,-0.471242842,0.2162113607

C,0,-0.1002757343,0.4271831125,0.8117428821

C,0,-1.3560161486,0.5436855371,0.2695568083

C,0,-1.8146685205,-0.2864914666,-0.9229817866

C,0,-0.954606874,-1.5290261642,-1.1120282191

H,0,-1.2105163715,-2.2482954121,-0.3194924225

H,0,-1.1902166235,-2.0042633192,-2.0706754039

H,0,-1.7683839834,0.3436566234,-1.829963122

C,0,-3.252021742,-0.7047314488,-0.6338811933

H,0,-3.2047105582,-1.2048821224,0.3484797601

C,0,-4.2224504294,0.464805869,-0.4629087862
C,0,-3.6724611306,1.4195154332,0.5468570836
C,0,-2.3588279854,1.4405168448,0.8637611536
H,0,-2.0243614128,2.1750000922,1.5909056451
C,0,-5.4959228503,-0.2990172491,-0.0732162694
C,0,-5.4684398387,-1.5274264285,-1.0195384469
C,0,-4.0133685318,-1.65609277,-1.5531658196
H,0,-3.9421265432,-1.3301086991,-2.5993256425
H,0,-3.6391586442,-2.6831273301,-1.5002277523
H,0,-5.7703326461,-2.4151205907,-0.4517285294
H,0,-6.1899236572,-1.4044152612,-1.8336386897
O,0,-5.3757846294,-0.6779991692,1.3041954801
H,0,-6.076651187,-1.3115940661,1.5260398864
H,0,-6.3998477798,0.311230889,-0.2010858329
C,0,-4.5115464645,1.2314270793,-1.7683562125
H,0,-5.087217169,2.1376919431,-1.5440595971
H,0,-3.5839649965,1.5329283668,-2.2681272654
H,0,-5.0987878245,0.6292065286,-2.4712633783
C,0,0.300728263,1.0554641916,2.1265397331
C,0,1.7586859301,1.4908713158,2.1109573094
C,0,2.6627207011,0.3432989076,1.7675673811
C,0,2.1254534039,-0.6088725709,0.7979654266
H,0,2.6723910373,1.0319341652,-1.3806479595
O,0,3.8169944151,0.261872688,2.2136363925
H,0,1.9122729749,2.2570623501,1.3331274304
H,0,2.078378542,1.9150285082,3.0669183659
H,0,0.1566494892,0.3050058105,2.9210879073
H,0,-0.3242282191,1.9098536915,2.3848987502

H,0,0.8175109589,-0.5679080667,-1.8882870217

H,0,1.1264686583,-2.1179276273,-1.1357564325

Cl,0,3.2379184618,-1.8559460256,0.3011828843

O,0,2.3227268263,-4.239808057,-1.9519781304

H,0,1.9221850075,-4.7155474736,-2.6952311448

H,0,2.6308456974,-3.3795632611,-2.3173868315

O,0,3.3028337386,-1.8334436232,-3.0997861663

H,0,2.9669134232,-1.6726314818,-3.9945255114

O,0,4.5678657939,3.1310907654,0.7518486689

H,0,4.2635431387,2.9496140852,1.655770096

O,0,2.3960959118,3.9203225105,-0.7437630655

H,0,2.6792406475,4.7081133606,-1.2328337715

O,0,0.5145581773,2.4771022348,-2.256012692

H,0,0.3949656616,1.6015659127,-1.8544827021

H,0,1.21854881,2.9194048489,-1.7286758519

H,0,4.9956000578,2.3001721765,0.4489304781

H,0,3.1826290129,3.6065120329,-0.2321564251

O,0,5.5731933526,0.6322132524,-0.0438001618

H,0,6.4700517449,0.3369307026,-0.2642520014

H,0,5.2724798895,0.1069837562,0.7217122391

H,0,-4.3480751749,2.1433873507,1.0038839957

O,0,0.5490916793,-4.3985852071,0.2700793775

H,0,1.1816730942,-4.2740616631,-0.473111813

H,0,0.8831273701,-5.1559195034,0.7745715828

O,0,-0.4334422649,-2.6519731614,2.3116135813

H,0,0.2412386529,-1.9858141735,2.5204545163

H,0,-0.0760563268,-3.1544044699,1.546259027

H,0,-3.596017121,-1.0150586223,2.3606375168

O,0,-2.9064346873,-1.2172248287,3.021414052

H,0,-2.1750751511,-1.6591211058,2.5463435369

O,0,3.4427591292,0.7806931268,-1.9175934285

H,0,3.2804751489,-0.963705567,-2.6420942258

H,0,4.1911856714,0.7033028816,-1.2852691772

O,0,-0.0358455912,4.2791796217,0.7259531511

H,0,-0.7161760247,4.1867808015,0.0285954892

H,0,0.8216998574,4.1781970775,0.2624022781

O,0,-1.8444853767,3.7980226966,-1.5027618119

H,0,-1.0759413587,3.2855532724,-1.8461943965

H,0,-2.41342474,3.1482755859,-1.0567083287

C4-C5 chlorohydrin formation TS

SCF Done: E(RMN15) = -2224.53872397

Sum of electronic and thermal Free Energies= -2223.976223

O,0,0.0404864119,0.0323849726,-0.0167664286

C,0,0.0277266367,0.0153238107,1.9821453613

C,0,1.5000289271,0.0259132496,2.2045462979

C,0,-0.7627526432,1.1963751287,2.1651068804

C,0,-0.1396438998,2.3819716131,2.5049474707

C,0,1.3634587635,2.4313692761,2.7232068168

C,0,2.0882292372,1.3874034257,1.8814138221

H,0,1.9635421948,1.6187665716,0.8126291886

H,0,3.159748734,1.4012113454,2.108230349

H,0,1.527798605,2.1681385782,3.7845862528

C,0,1.8548186701,3.8498910206,2.4958259003

H,0,1.5966463267,4.1203031288,1.4573904813

C,0,1.1219153059,4.8506372027,3.4013550915

C,0,-0.3417894441,4.7668625335,3.1178188688

C,0,-0.9087195278,3.5995819078,2.7237890264

H,0,-1.9838685178,3.5656898642,2.5730119162

H,0,-0.9714680171,5.6408235623,3.2885175373

C,0,1.8410121561,6.1566838468,3.0445160139

C,0,3.3207745809,5.7229004633,2.9199585107

C,0,3.3283633368,4.1819350798,2.7190090949

H,0,3.7019541706,3.6688695247,3.6154643068

H,0,3.9573372104,3.8723161006,1.8791213479

H,0,3.7762892289,6.2492829158,2.0737240611

H,0,3.8786243011,6.0114757464,3.8166600587

O,0,1.312589897,6.6273901978,1.8001140537

H,0,1.8530111358,7.3651875785,1.475081216

H,0,1.6925300276,6.9289846405,3.8101521613

C,0,1.3274341978,4.5996613884,4.911643309

H,0,0.7949077495,5.3712347143,5.4814528353

H,0,0.9370696941,3.6232683402,5.2197515049

H,0,2.3881462227,4.6530531974,5.1836850971

C,0,-2.2476549701,0.9632751578,2.0066019695

C,0,-2.7248786129,0.0235771659,3.1290980945

C,0,-1.6540041745,-0.9698355064,3.4845765525

C,0,-0.6431160184,-1.3039400761,2.3777419719

H,0,0.1134467053,-1.9944881845,2.7628835073

Cl,0,-1.548953325,-2.1981721445,1.1152316289

O,0,-1.5386975726,-1.4880145415,4.5859787525

H,0,-2.9640950916,0.5797586035,4.0412387296

H,0,-3.6238577364,-0.5347770324,2.837151364

H,0,-2.4256423904,0.5066796916,1.0232354402

H,0,-2.8293758344,1.8850496669,2.0278074299

H,0,1.6334356038,-0.1933373927,3.2815089269

H,0,1.9556025187,-0.810974932,1.6593801635

O,0,2.3186664266,-0.5364350394,-1.0441319719

H,0,3.0250043799,-0.1103515126,-0.5316276403

H,0,2.3135606767,-1.5398860493,-0.763293558

O,0,2.2070908668,-2.926014417,-0.2416976969

H,0,2.4395213294,-3.5753772699,-0.9222687411

O,0,3.0834848266,-2.6919891948,3.7421600157

H,0,3.7032770242,-3.2753863364,4.2071104115

H,0,2.5468462767,-3.2762001691,3.1421316071

O,0,1.2239861812,-1.8428823707,5.641230551

H,0,0.3439067983,-1.8219371103,5.2222961311

H,0,1.8552466161,-2.1466808964,4.9480838672

O,0,1.9228580506,0.8622699791,6.1435909326

H,0,1.0408126969,1.2674017231,6.0130953454

H,0,1.760698622,-0.0932247634,5.9811546503

O,0,1.6748339256,-4.1746069164,1.9596358212

H,0,1.8233204662,-3.6930216381,1.0718590733

H,0,0.7184048164,-4.2395094244,2.1003021705

H,0,0.9655188452,-0.176107067,-0.4204989818

H,0,-0.5947775458,-0.5823195004,-0.425883076

O,0,-0.8691138388,2.7241490046,-0.7178376464

H,0,-1.6537700473,2.8353998837,-0.1562851953

H,0,-0.491126288,1.8578077437,-0.4619863326

H,0,0.4721207081,5.6996765773,0.361044524

O,0,0.0524142741,5.4338676434,-0.4813172993

H,0,-0.1205129595,4.4727759679,-0.4265007752

O,0,4.3652118485,-2.005556526,1.1804878966

H,0,3.6216664796,-2.3827512575,0.6379039889

H,0,4.0672430743,-2.1061337657,2.1049876758

O,0,-0.7922821931,1.5603862508,5.6514826514

H,0,-1.278148339,2.3965459012,5.8309315289

H,0,-1.3999045628,0.8311555059,5.8517184175

O,0,-2.0483101196,4.0489804557,6.0974440965

H,0,-1.2996137784,4.6246653513,6.3237350227

H,0,-2.3288990714,4.3371552054,5.213715379

C4-C9 chlorohydrin formation TS

SCF Done: E(RMN15) = -2224.54425276

Sum of electronic and thermal Free Energies= -2223.981990

C,0,-0.9638631025,-0.7818836978,0.9940005227

C,0,-1.135660461,-0.0876118282,-0.3236900382

C,0,-0.1347427219,0.3107972883,-1.1439818057

C,0,1.255126523,-0.0383313146,-0.7707521319

C,0,1.4766730948,-0.3904417914,0.6830596105

C,0,0.4287312739,-1.3621684135,1.2100159661

H,0,0.533445702,-2.3377818725,0.7176914924

H,0,0.6016686646,-1.5239468546,2.2793005913

H,0,1.2968694487,0.5747367328,1.1933078081

C,0,2.9227805376,-0.7901652556,0.9069512932

H,0,3.1410837035,-1.6356043451,0.2346992987

C,0,3.8750878085,0.344083072,0.5023745123

C,0,3.6132334006,0.7081884499,-0.9241011252

C,0,2.3939224606,0.5592333366,-1.4878641072

H,0,2.239018697,0.8767707484,-2.5150518118

C,0,5.2365828004,-0.2661785638,0.8606578588

C,0,4.9481929552,-1.0007769889,2.1943805987

C,0,3.4070419318,-1.1701507979,2.3035815034

H,0,2.9862371104,-0.4882033373,3.0544135804

H,0,3.1171579739,-2.1868028206,2.5849697168

H,0,5.4631230716,-1.9679137531,2.1819645134

H,0,5.3500061109,-0.4326002541,3.0392982633

O,0,5.6272767731,-1.1688755334,-0.1786573055

H,0,6.4093737697,-1.6695264175,0.1044761815

H,0,6.0103287585,0.5047195536,0.9673241747

C,0,3.7344094782,1.6322206517,1.3437575405

H,0,4.4662936171,2.3700629962,0.9922846384

H,0,2.738860352,2.0774604376,1.2546633786

H,0,3.9386770674,1.4402600463,2.4032913101

C,0,-0.4532202921,1.0319601809,-2.4334873742

C,0,-1.6256297351,2.0012945353,-2.2579924434

C,0,-2.8064010438,1.4109921397,-1.5534598053

C,0,-2.559879322,0.1459318599,-0.7410321806

H,0,-3.2523740133,0.1039762184,0.1062110683

O,0,-3.9328345024,1.8888319253,-1.6012667912

H,0,-1.3051322154,2.8385349487,-1.618866788

H,0,-1.9568734836,2.4301479936,-3.2080369971

H,0,-0.6756368158,0.300482444,-3.223915417

H,0,0.4044927037,1.6146212633,-2.7761336175

H,0,-1.1833384948,-0.0307637548,1.7708081555

H,0,-1.740033676,-1.5491808343,1.0960160293

Cl,0,-3.0700311214,-1.1912940263,-1.874493094

O,0,-2.4187364005,-3.9221786855,0.7126095065

H,0,-1.8693445041,-3.9958630157,1.5081995394

H,0,-3.1710871509,-3.2759941335,0.9520281753

O,0,-4.3544308345,-2.2852165253,1.3581962839

H,0,-4.9887788144,-2.8312978955,1.8480225802

O,0,-5.0527600451,2.0226599654,1.2965331346

H,0,-5.5087731121,2.1120714985,2.1472569379

O,0,-2.3029765721,2.5340397484,1.2527722355

H,0,-2.112976509,3.2681232988,0.6474001001

O,0,0.1722519882,2.3910066742,2.7041167564

H,0,0.5882539857,2.7070826787,1.8756262834

H,0,-0.7674078046,2.3009266876,2.4512844098

H,0,-5.3439320623,1.1570019286,0.9108169508

H,0,-3.2790185885,2.4029357704,1.243341132

O,0,-5.8186025343,-0.4156937584,0.2558210703

H,0,-5.243173321,-1.1662695792,0.6359467963

H,0,-5.6554587051,-0.3897040151,-0.6999430408

H,0,4.4065718509,1.1859982604,-1.5009690317

O,0,-0.5963525554,-3.4970193084,-1.3068807943

H,0,-1.2615921076,-3.552591411,-0.5717959558

H,0,-1.107925301,-3.4872081156,-2.130889099

O,0,1.345391555,-1.7504347015,-1.6233016521

H,0,1.2898236253,-1.5487142007,-2.578072322

H,0,0.5604488464,-2.3636546944,-1.4257947367

H,0,4.6392336208,-2.1083742995,-1.4363223528

O,0,4.1521231597,-2.6281351812,-2.1095327417

H,0,3.2130922364,-2.456198412,-1.9183702045

O,0,-3.7320722363,-0.2830939848,3.0136495215

H,0,-3.943670738,-1.0488012308,2.3963954903

H,0,-3.860553757,0.5207773398,2.4828794013

O,0,0.7997063018,3.2933233532,0.0614786635

H,0,1.6107300051,3.5980481535,-0.4056648764

H,0,0.1025583044,3.9321298027,-0.153861824

O,0,3.1272882188,4.0869958984,-1.2954726332

H,0,3.3172504668,5.0265955737,-1.1475492929

H,0,3.8804131464,3.6058716756,-0.9162087244

C4-C12 chlorohydrin formation TS

SCF Done: E(RMN15) = -2224.55052645

Sum of electronic and thermal Free Energies= -2223.988427

C,0,-1.0207258462,-0.695108731,1.0950918704

C,0,-1.1575685489,0.2200970694,-0.0790791094

C,0,-0.1112650953,0.8082751446,-0.7225779282

C,0,1.2507985015,0.4687062742,-0.3258631121

C,0,1.4507635035,-0.3779004257,0.9146958944

C,0,0.3251492023,-1.3979813426,1.0788784057

H,0,0.3593298355,-2.1099221067,0.2392469196

H,0,0.4792584149,-1.9645608361,2.0042273028

H,0,1.3912768609,0.3206289782,1.7700074456

C,0,2.8176394661,-1.0427772771,0.910737415

H,0,2.8073790001,-1.8146908446,0.1222143751

C,0,3.9363523975,-0.0649924792,0.5458591312

C,0,3.6569302272,0.5377676235,-0.785769777

C,0,2.3230575626,0.8965496371,-1.0887842499

H,0,2.163098035,1.4935712859,-1.9809182501

C,0,5.2047917613,-0.8923517137,0.8176530272

C,0,4.8534778621,-1.6210681637,2.1318919217

C,0,3.3036615609,-1.6704469635,2.2216277509

H,0,2.9446716108,-1.0754671997,3.0717799206

H,0,2.9259576308,-2.6879927647,2.3539631101

H,0,5.3032724305,-2.6193876153,2.113562118

H,0,5.2843109165,-1.0912421064,2.9871644956

O,0,5.4015767327,-1.832508206,-0.2413947352

H,0,6.0984892039,-2.4636480019,-0.0014530916

H,0,6.0930463891,-0.2550381723,0.9059136485

C,0,4.0048395788,1.1707447092,1.4896280035

H,0,4.849927881,1.8076841739,1.2052678461

H,0,3.0890563574,1.7707147774,1.4548776165

H,0,4.1646743354,0.8403643957,2.5214111768

C,0,-0.3909839532,1.7156833029,-1.9017731831

C,0,-1.6476275439,2.5559407848,-1.6611731451

C,0,-2.8365658875,1.7273140036,-1.2911137748

C,0,-2.5455462971,0.3897748911,-0.6198569355

H,0,-3.314759762,0.1769907981,0.1298965203

O,0,-3.9975131565,2.0618669349,-1.4871466327

H,0,-1.4657516252,3.2181643985,-0.7981198217

H,0,-1.9014910979,3.1881777817,-2.5161617823

H,0,-0.495550549,1.1160832099,-2.8185749387

H,0,0.4377074046,2.4060301282,-2.0686966895

H,0,-1.1309294642,-0.0893516928,2.0108911037

H,0,-1.8504027001,-1.4101849545,1.0973062181

Cl,0,-2.7599023419,-0.8657346586,-1.9269160436

O,0,-2.1162000487,-3.9530353328,0.4645265881

H,0,-1.4825639983,-3.7400786523,1.1671306709

H,0,-2.9536681026,-3.4028950957,0.6615858423

O,0,-4.2681383175,-2.5862274839,1.0046024222

H,0,-4.8872373293,-3.2366281335,1.3708493376

O,0,-5.3866576133,1.4598552202,1.3410727774

H,0,-6.1308543186,1.5663185035,1.9527006537

O,0,-2.8063007768,2.5169284724,1.6906866781

H,0,-2.8156984119,3.3279664387,1.1584167008

O,0,0.0408783798,2.6075092801,2.3070801576

H,0,0.4778892138,2.9708988287,1.5079422199

H,0,-0.8786163442,2.4072905116,2.0467946484

H,0,-5.5853982058,0.6693014495,0.7736689176

H,0,-3.6998906146,2.1204961785,1.5832754527

O,0,-5.7945944849,-0.8054006535,-0.1297826063

H,0,-5.1813552961,-1.5211481315,0.2653912498

H,0,-5.5136417708,-0.6629955527,-1.0468877659

H,0,4.4658132809,1.0210507152,-1.3302198613

O,0,-0.6504218256,-3.8829379381,-1.8720521772

H,0,-1.1926200073,-3.8404448171,-1.0449588912

H,0,-1.2864239714,-3.86391127,-2.6036386097

O,0,1.1605063962,-1.8882840548,-2.5791699646

H,0,0.6467046869,-1.0675306715,-2.652830528

H,0,0.5281183945,-2.5728452523,-2.2536581779

H,0,4.4211774454,-1.6419676593,-1.6624331347

O,0,3.8640221467,-1.0678169214,-2.2365081994

H,0,2.9559904172,-1.4505111227,-2.2556617528

O,0,-3.9809807152,-0.691044557,2.8923272816

H,0,-4.0670767112,-1.4113587777,2.1995191149

H,0,-4.3730928942,0.1034259979,2.4861154914

O,0,1.2767343312,3.9311406795,0.111416276

H,0,2.1951775695,3.8639442823,-0.2351832685

H,0,1.1642716104,4.8571643962,0.3756272666

O,0,3.884527141,3.9150723731,-0.9783067808

H,0,4.4958069381,3.4390074072,-0.3934703871

H,0,3.8436840123,3.3847922957,-1.7902846791

C4-C12 chlorohydrin

SCF Done: E(RMN15) = -2224.57393342

Sum of electronic and thermal Free Energies= -2224.009311

C,0,-1.0245477452,-0.6209884137,1.1996001883

C,0,-1.1600529034,0.2656636662,-0.0048018124

C,0,-0.1115213137,0.816775282,-0.6615913792

C,0,1.2713046258,0.4559260347,-0.2875868392

C,0,1.4695771948,-0.3456920935,0.9915841224

C,0,0.3194461585,-1.3307490211,1.2012531425

H,0,0.3411873938,-2.0676283719,0.3829223055

H,0,0.4695664395,-1.8769976441,2.1406309985

H,0,1.4662795263,0.3681356936,1.8340198753

C,0,2.8023131356,-1.0785650613,0.9657115573

H,0,2.7050052754,-1.890442549,0.2228203908

C,0,3.9542239276,-0.1785767073,0.5039105932

C,0,3.6964904304,0.2519221788,-0.9355988782

C,0,2.296711439,0.7640249776,-1.1129693723

H,0,2.1245099058,1.325209722,-2.0288988211

C,0,5.1747314666,-1.0707496633,0.7818708267

C,0,4.8585661284,-1.6867979856,2.1532452501

C,0,3.310493125,-1.6807808643,2.2846470312

H,0,2.9946873028,-1.0507549111,3.1266674943

H,0,2.9052620207,-2.6815158523,2.4597382764

H,0,5.2873465825,-2.6924897132,2.2040416155

H,0,5.3269231608,-1.0934945715,2.945270972

O,0,5.2226027309,-2.1133028973,-0.2241826466

H,0,6.1290031399,-2.4455918205,-0.3251272683

H,0,6.1176760011,-0.5154081372,0.7525692172

C,0,4.0955055926,1.0936066995,1.3553448819

H,0,5.012138814,1.6283294602,1.0748245181

H,0,3.2442029416,1.7703873549,1.2128540723

H,0,4.1577096884,0.854195899,2.4219412371

C,0,-0.3805780405,1.7109572829,-1.8522823292

C,0,-1.6258834826,2.5711245052,-1.6217427069

C,0,-2.8228887609,1.7641385219,-1.2300893475

C,0,-2.5401085715,0.4281513724,-0.5573111771

H,0,-3.3223340197,0.2102138586,0.177446229

O,0,-3.9804987687,2.1245754812,-1.4087404476

H,0,-1.4342150157,3.2433269667,-0.7680616282

H,0,-1.8776906425,3.1950220122,-2.4838019725

H,0,-0.4945843715,1.1044759574,-2.7634924887

H,0,0.4598926156,2.3865503246,-2.0278903435

H,0,-1.1381088221,-0.005813593,2.1075355387

H,0,-1.8479973041,-1.3438871392,1.214133569

Cl,0,-2.779849338,-0.8123547259,-1.8960705271

O,0,-2.024620188,-3.8503660823,0.4226292691

H,0,-1.4524691068,-3.6949131977,1.1904379599

H,0,-2.8836773034,-3.3358042172,0.6126645893

O,0,-4.2408277789,-2.5682365656,0.9741672243

H,0,-4.8414868579,-3.2459070794,1.3211288414

O,0,-5.4253824015,1.4657814845,1.376313984

H,0,-6.1759466636,1.547032393,1.9839187052

O,0,-2.8614697487,2.5672780502,1.7924345277

H,0,-2.8524318258,3.347330955,1.2153788465

O,0,0.041720422,2.7180410122,2.2805149428

H,0,0.478363992,3.0547805091,1.4693917703

H,0,-0.8664953086,2.4735798548,2.0213498476

H,0,-5.6034984804,0.6796410906,0.7966166969

H,0,-3.7389464368,2.1473367039,1.6504851799

O,0,-5.7812705056,-0.7863927761,-0.1379750548

H,0,-5.164092188,-1.5018368553,0.25355748

H,0,-5.4666864438,-0.6078183502,-1.0379861243

H,0,4.4402529416,0.9778440906,-1.2795157474

O,0,-0.1426040957,-3.6986072132,-1.6367362072

H,0,-0.8437567982,-3.664139752,-0.9419370406

H,0,-0.5539346508,-4.1053829551,-2.41435209

O,0,1.6312166246,-1.8474124527,-2.662112042

H,0,1.0747146802,-1.0674252194,-2.832070686

H,0,1.0551309011,-2.480620689,-2.1647284794

H,0,4.482825705,-1.567207379,-1.3971602196

O,0,3.8487211743,-0.8989498974,-1.8656955162

H,0,2.9376767929,-1.3430379107,-2.1221039091

O,0,-4.0471906899,-0.7134615707,2.9185155538

H,0,-4.0994212663,-1.4161648385,2.2048403515

H,0,-4.4126876171,0.0932421935,2.5117738355

O,0,1.3023649896,4.0007774491,0.0787230868

H,0,2.1951155613,3.8520129773,-0.3068849621

H,0,1.2966842536,4.9232801109,0.3767175762

O,0,3.8595338843,3.7423405355,-1.1064140517

H,0,4.4275766702,3.2237396233,-0.5138566146

H,0,3.7529280996,3.1929544532,-1.8995594409

C11-C12 epoxide product complex

SCF Done: E(RMN15) = -2224.61198430

Sum of electronic and thermal Free Energies= -2224.046404

C,0,0.7749468003,0.5380018816,-1.4573410891

C,0,1.1612820562,0.6895726161,-0.0165378713

C,0,0.1182494545,1.1242790846,0.9209622172

C,0,-1.1715269688,1.2229373354,0.4962798722

C,0,-1.6319589931,0.7811009697,-0.8862287626

C,0,-0.5951385838,-0.1187789893,-1.5468705004

H,0,-0.576372042,-1.08907753,-1.0218270339

H,0,-0.8715878038,-0.3102863358,-2.5906210313

H,0,-1.7706805607,1.6688016997,-1.5288025485

C,0,-2.9763127567,0.0767329804,-0.7447115303

H,0,-2.8353811269,-0.7576333935,-0.041500132

C,0,-4.0696412278,0.9718035215,-0.147808127

C,0,-3.6609083844,1.4527073988,1.2198305128

C,0,-2.2220838898,1.6546616405,1.4749525248

H,0,-1.9422705701,2.4341257378,2.1799129113

C,0,-5.2914267683,0.04995528,-0.2410734351

C,0,-5.1309457507,-0.6098056063,-1.6285454769

C,0,-3.6303513832,-0.4891815054,-2.0084112274

H,0,-3.4917571354,0.202551401,-2.8504383864

H,0,-3.1928582517,-1.4496665388,-2.3030324971

H,0,-5.4617152144,-1.6534662989,-1.5696226371

H,0,-5.7700139186,-0.1093781859,-2.3639130302

O,0,-5.2042565313,-0.9121991827,0.8143499636

H,0,-5.8952155127,-1.5805097222,0.68378073

H,0,-6.2336216909,0.6069422461,-0.147661306

C,0,-4.3393217514,2.2397745171,-0.9732783594

H,0,-5.2000919534,2.7769189105,-0.5571212791

H,0,-3.4781878931,2.9194460705,-0.9516787173

H,0,-4.5628169159,2.0045665059,-2.0195403985

C,0,0.5109936501,1.2786529979,2.3761008912

C,0,1.9958335725,1.5610873189,2.59772815

C,0,2.902169078,0.7325226512,1.7309189914

C,0,2.4295908263,0.4210201685,0.3908668145

H,0,3.1568761494,0.0195783824,-0.3136881436

O,0,4.0545034912,0.4339540677,2.0978325671

H,0,2.2249029361,2.606258611,2.3380978491

H,0,2.2739329218,1.4181485356,3.6462393897

H,0,0.256596881,0.3358264289,2.8811432547

H,0,-0.0736515066,2.0637923251,2.8625274383

H,0,0.7196162168,1.5451017668,-1.90555959

H,0,1.5472517182,-0.0175954987,-1.9970694529

Cl,0,1.497065591,-2.236474942,2.9365542403

H,0,0.7892772414,-2.9367019361,-2.2169415624

O,0,1.7507652462,-2.8265220682,-2.0166390395

H,0,2.1729961814,-3.6363199396,-2.3448464649

O,0,5.4402751996,-1.3654381881,-0.5254519827

H,0,6.236925822,-1.9161809243,-0.4945945895

O,0,5.5216964659,1.3669761985,-0.3899680748

H,0,5.2810863403,1.5151809434,0.5428579759

O,0,3.7469276397,2.3952909398,-1.8166950409

H,0,3.1054932676,2.9934421978,-1.2453652489

H,0,4.5051465196,1.9564399923,-1.2196545051

H,0,4.7120256548,-1.8667617911,-0.0775137296

H,0,5.5264125463,0.3794371259,-0.4834592377

O,0,3.2950498732,-2.8034201373,0.4041299344

H,0,2.6660038038,-2.7526812692,-0.3472160551

H,0,2.779789501,-2.5465589885,1.2039297378

H,0,-4.3639748623,2.1057874086,1.7393650512

O,0,-2.3921078536,-3.410411445,-0.3123309956

H,0,-1.4368420565,-3.3205529385,-1.8338674426

H,0,-2.4249043221,-4.3341866083,-0.0200971988

O,0,-1.4863448704,-1.9866088157,1.9007193172

H,0,-0.5465733163,-1.9386439848,2.1910859793

H,0,-2.0244760999,-2.8994317439,0.4473947633

O,0,-2.9572004226,0.5666809982,2.0984955848

H,0,-1.8638310895,-1.0870986124,1.9475681511

O,0,3.874231661,-1.0252079898,-2.8987250117

H,0,3.0864024771,-1.5476493787,-2.6474724033

H,0,4.4947909587,-1.1277701885,-2.1480768898

O,0,2.2323586842,3.8160995858,-0.3935625825

H,0,1.292650858,3.9090100616,-0.6946398966

H,0,2.5847564568,4.715602368,-0.2988091972

O,0,-0.3934720798,4.1327811363,-1.1804941133

H,0,-0.537265973,3.5757497194,-1.9630834832

H,0,-0.9504470823,3.7416987952,-0.486044398

H,0,4.144930207,2.9305235314,-2.5264082019

O,0,-0.9251147989,-3.3139086315,-2.6784752144

H,0,-1.253013216,-2.5452042643,-3.1717731696

Coordinates, Electronic Energies and Free Energies for Reactions with Cl_2; MN15/6-31+G(d,p) + SMD water

Cationic intermediate following chlorination at C4

SCF Done: E(RMN15) = -2650.57108452

Sum of electronic and thermal Free Energies=   -2649.953057

C,0,0.6423138132,1.0626184262,-1.3101600115

C,0,1.0387461036,0.8300373473,0.0780327431

C,0,0.0549616031,0.6783652913,1.1611293936

C,0,-1.2110170757,-0.0916128816,0.6895168033

C,0,-1.6940925348,0.3244834961,-0.7146805479

C,0,-0.5650841203,0.2171278921,-1.7320021904

H,0,-0.2459879976,-0.8322979348,-1.8238035817

H,0,-0.8966132952,0.5516356699,-2.7199859784

H,0,-2.0404510262,1.371210092,-0.6975339195

C,0,-2.862886513,-0.583896913,-1.0814746118

H,0,-2.4806080781,-1.6223455308,-1.0079304105

C,0,-4.0373404587,-0.4883630751,-0.0940891965

C,0,-3.5435360299,-0.8969750995,1.2922495121

C,0,-2.3494651628,-0.0315814577,1.7202382359

H,0,-2.671423657,1.0113929221,1.8505405421

C,0,-5.0005470517,-1.47013272,-0.769377202

C,0,-4.9010560478,-1.1253230652,-2.2736542297

C,0,-3.5255292937,-0.4313962705,-2.4559574957

H,0,-3.6488371793,0.6338651047,-2.695393184

H,0,-2.9290933839,-0.87883003,-3.2574931495

H,0,-5.000422988,-2.0226939469,-2.8924433814

H,0,-5.7280869247,-0.454139175,-2.5356427703

O,0,-6.3179924741,-1.3595909004,-0.2361298772

H,0,-6.8741096068,-2.051861855,-0.6254056197

H,0,-4.6298325842,-2.4918055525,-0.5902024684

C,0,-4.6762691028,0.9088782682,-0.0559427742

H,0,-5.4380412574,0.9473381031,0.7329823757

H,0,-3.9409394335,1.6918423395,0.1536720855

H,0,-5.1662601037,1.1625271164,-1.0034230521

C,0,0.6741609908,0.0469072807,2.4217753999

C,0,1.9470494669,0.747605801,2.895781955

C,0,2.9555858423,0.9532288585,1.8114965295

C,0,2.4922582901,0.7605403763,0.3725394584

H,0,3.1254541157,1.2982255657,-0.3388315454

O,0,4.1212342021,1.270690821,2.0012158804

H,0,1.7166257669,1.7521089075,3.2808606677

H,0,2.426999967,0.2064147914,3.7170327474

H,0,0.8647320043,-1.0166339377,2.2275219621

H,0,-0.0625800667,0.0992607644,3.2276379662

H,0,0.3542586686,2.1388125853,-1.310605472

H,0,1.5025478891,0.9777188595,-1.9823041277

Cl,0,2.5745523767,-1.0156690264,-0.0074785095

H,0,4.9774649746,-2.999735298,-0.1091958792

O,0,5.7980655037,-2.5315298803,0.1705914738

H,0,5.9271586621,-2.7567866847,1.1046156123

O,0,3.2171286628,3.0749975031,-2.4282387069

H,0,3.6206135196,2.1764897357,-2.5028593252

O,0,2.3941663482,4.1307717259,-0.003005689

H,0,2.7754196854,3.7264121548,0.7909558627

O,0,-0.1147916718,4.4593498586,-1.3611832756

H,0,-0.9736897571,4.3295202139,-0.9054521414

H,0,0.5933552005,4.3277997243,-0.7004636147

H,0,3.8633465758,3.6908833171,-2.8058754741

H,0,2.7828131913,3.6672789527,-0.7820749448

O,0,5.8363194769,0.2203674721,-0.2777483268

H,0,5.430989316,0.6800131782,0.4791286718

H,0,5.792302875,-0.7435198272,-0.0721507551

H,0,-4.3495645834,-0.8163476878,2.034285246

O,0,0.1755445696,-3.4196535174,-0.4072671441

H,0,1.1210383523,-3.6606139275,-0.5467917231

H,0,-0.3435921337,-4.2066965767,-0.6408934288

O,0,-0.8076903514,-3.3965572627,2.2268759904

H,0,-1.0143255005,-2.5034101905,2.5459380305

H,0,-0.3044369711,-3.2738886497,1.3900355

H,0,-2.7432825695,-3.8128397847,-0.4059476149

O,0,-2.690468641,-4.5064267215,0.2712359984

H,0,-2.1750702129,-4.1076715238,1.0019640807

O,0,4.3239124582,0.5357900966,-2.5941406439

H,0,4.866530527,0.410013765,-1.7782449393

H,0,4.9433199365,0.487590091,-3.3383861999

O,0,-2.7677152118,4.1676742212,-0.4155812972

H,0,-3.0255587902,3.9655480361,0.5120145591

H,0,-3.2366168199,4.9823273755,-0.6519831118

O,0,-3.5470141519,3.5757553876,2.2365294655

H,0,-4.1601639046,2.8248516768,2.1752937881

H,0,-2.7797896934,3.2389071584,2.7267956777

H,0,-3.2283768298,-1.9525321624,1.2588857529

H,0,-1.9951723005,-0.3750056676,2.699873515

H,0,-0.8917017887,-1.1459422254,0.6023965579

C,0,-0.268772627,2.1982307099,1.4439122536

H,0,-0.6983798639,2.230180233,2.4519787265

H,0,0.6175676651,2.8425886899,1.4315954667

H,0,-0.9832534746,2.5794339232,0.7095710564

Cl,0,3.2272543363,-4.1131695201,-0.7826353783

C4-C5 chlorohydrin formation TS

SCF Done: E(RMN15) = -2650.56392515

Sum of electronic and thermal Free Energies= -2649.944947

C,0,0.7040652683,0.5364419599,-1.1975052321

C,0,0.953980421,0.6532519771,0.2080923168

C,0,-0.104226214,0.5942273336,1.2311038389

C,0,-1.3170237756,-0.2597134916,0.7781388639

C,0,-1.7301767957,0.0268341172,-0.6818830651

C,0,-0.5650570267,-0.1656482919,-1.6524659508

H,0,-0.3544079294,-1.2379454903,-1.7690862535

H,0,-0.8352399628,0.2131431154,-2.6443181071

H,0,-2.0846233738,1.0674066641,-0.7699552508

C,0,-2.8710597098,-0.9208714599,-1.0360258051

H,0,-2.4734521635,-1.94423523,-0.8899522531

C,0,-4.0898506941,-0.7995164632,-0.1081548691

C,0,-3.6486097176,-1.0888397952,1.3256869858

C,0,-2.5139151302,-0.1408215887,1.7377907232

H,0,-2.8851637191,0.893505653,1.7564744834

C,0,-4.9883755268,-1.8572751392,-0.7563114755

C,0,-4.8313237236,-1.608613659,-2.2754795246

C,0,-3.4789667363,-0.8664490548,-2.4424426252

H,0,-3.6354140584,0.1792069162,-2.7424101044

H,0,-2.8324315828,-1.3291413103,-3.1951852627

H,0,-4.8612564539,-2.5490951188,-2.8345895634

H,0,-5.6728235097,-0.9958626019,-2.6211650964

O,0,-6.3309659299,-1.757613864,-0.2879147691

H,0,-6.8531758991,-2.4798392746,-0.6698449537

H,0,-4.592876323,-2.8523092059,-0.4942393803

C,0,-4.7728209202,0.5744883471,-0.1992606079

H,0,-5.5301494141,0.6695574879,0.5896961141

H,0,-4.0581496901,1.3934173815,-0.076745513

H,0,-5.2769854835,0.7195708656,-1.1618093595

C,0,0.5301784588,0.1246959369,2.5633611878

C,0,1.5870993528,1.1023533385,3.093964087

C,0,2.3894644242,1.7486532798,2.0024021923

C,0,2.3421302809,1.0780786047,0.6325130263

H,0,2.7986216337,1.7151573472,-0.1326057159

O,0,3.0679771382,2.7544528746,2.1495539766

H,0,1.1418994561,1.9130262623,3.6806907892

H,0,2.2910759673,0.5847544273,3.7580827609

H,0,0.9546139415,-0.8753934437,2.4200894425

H,0,-0.2666281761,0.0340073059,3.3070052029

H,0,0.5714371552,1.6558964905,-1.3494582854

H,0,1.616296476,0.3228627125,-1.7692334216

Cl,0,3.3486201546,-0.3979509772,0.7711270408

H,0,5.3792809893,-2.8072480324,-0.1259091742

O,0,6.1994231691,-2.3425174785,0.1690196433

H,0,6.1443053536,-2.2848719251,1.1353054383

O,0,2.8907388236,2.8152250813,-2.5766730591

H,0,3.2855812908,1.9104855697,-2.5503835185

O,0,1.77422547,4.2903119943,-0.5028666344

H,0,2.1017730899,4.0482730771,0.3782689681

O,0,-0.5544156877,3.4594692302,-1.9490518657

H,0,-1.4333348501,3.5548855092,-1.5206647777

H,0,0.109145201,3.7803733868,-1.304922626

H,0,3.525650407,3.3707432611,-3.0538281223

H,0,2.2755270981,3.7393648506,-1.1480865162

O,0,6.3059085085,0.1163298542,-1.111956659

H,0,6.2142415735,0.8071069031,-0.4378301041

H,0,6.2703917812,-0.7414158266,-0.6235364626

H,0,-4.4932800596,-0.9921898488,2.0212503766

O,0,1.0256295148,-2.382649394,0.214803516

H,0,1.8680438617,-2.7830275274,-0.1100035402

H,0,0.3381124896,-2.6855142217,-0.4011288658

O,0,-0.3663185323,-3.3052143411,2.5015416763

H,0,-0.8339086586,-2.4914483746,2.7523024366

H,0,0.2233000813,-3.0507196825,1.7542916775

H,0,-2.6820308152,-3.9719460972,0.1439473076

O,0,-1.8584906126,-4.4581378605,0.3057265586

H,0,-1.472736736,-4.0627896674,1.1153714136

O,0,3.9502923272,0.2442974196,-2.5900544529

H,0,4.7946560298,0.1728099085,-2.0824023343

H,0,4.1678272946,0.0284450365,-3.5098490494

O,0,-3.1632371325,3.8009661703,-0.9368215059

H,0,-3.3918360089,3.687549775,0.0134143657

H,0,-3.4260931229,4.7049357806,-1.1677657973

O,0,-3.8860828165,3.4671789997,1.7773324759

H,0,-4.4850404137,2.7027030502,1.7901406976

H,0,-3.1142733776,3.1929231934,2.2980317995

H,0,-3.2864770105,-2.1285940599,1.3883571028

H,0,-2.1991391145,-0.3828263809,2.7605251354

H,0,-1.0054577645,-1.31554516,0.8101965039

C,0,-0.5313996235,2.1053052469,1.3789475515

H,0,-1.0084058298,2.1986543067,2.3613285612

H,0,0.3015046803,2.8160085361,1.3400029389

H,0,-1.2345559323,2.3796890798,0.5881110591

Cl,0,3.6223295758,-3.7800302811,-0.8611277252

C4-C5 chlorohydrin

SCF Done: E(RMN15) = -2650.62830592

Sum of electronic and thermal Free Energies= -2650.002481

C,0,0.747634584,0.157198736,-0.9925685003

C,0,1.0100266018,-0.0185076369,0.5041402872

C,0,-0.2226708455,0.45005284,1.3460562483

C,0,-1.4602174265,-0.3900497827,0.9006728517

C,0,-1.7293310752,-0.2846415193,-0.6151111755

C,0,-0.4867322608,-0.6244939368,-1.4333324739

H,0,-0.2829987987,-1.7038254599,-1.3409277571

H,0,-0.6754399868,-0.4299138802,-2.4973720021

H,0,-2.0283407486,0.7475906367,-0.8499853538

C,0,-2.8847552468,-1.2136536987,-0.9653184018

H,0,-2.5880246266,-2.2241104441,-0.6203757082

C,0,-4.1857821057,-0.8799790029,-0.2201916009

C,0,-3.9168949372,-0.9812381994,1.2800573157

C,0,-2.7433508324,-0.0787681695,1.6954573687

H,0,-3.0274308,0.9743006711,1.5475192953

C,0,-5.0842210045,-1.9718204765,-0.8096722907

C,0,-4.7477940297,-1.961091899,-2.319556159

C,0,-3.3286247837,-1.343111946,-2.4280047896

H,0,-3.3624297725,-0.3497124648,-2.8987988788

H,0,-2.643816301,-1.9592416555,-3.0198689048

H,0,-4.7966180265,-2.9695726147,-2.7427122672

H,0,-5.495238746,-1.3517457048,-2.8424761948

O,0,-6.4609217079,-1.735209814,-0.5216936464

H,0,-6.985903958,-2.4755035363,-0.8629570667

H,0,-4.7869374407,-2.9341737702,-0.3637390909

C,0,-4.7543712014,0.499199843,-0.5870019144

H,0,-5.6160502421,0.7302725734,0.0512136186

H,0,-4.0167479154,1.2950781575,-0.4516235826

H,0,-5.0938375754,0.539326001,-1.6288023431

C,0,0.0820851776,0.208734956,2.8356820147

C,0,1.3658737878,0.9079884132,3.3030214042

C,0,2.5351613669,0.5489363262,2.4348432178

C,0,2.2822228758,0.7423603541,0.9493472444

H,0,2.146487662,1.8119201313,0.7647486136

O,0,3.6044119872,0.1552264515,2.883693123

H,0,1.2441771142,1.9993914525,3.2357141558

H,0,1.6099796904,0.6572798432,4.3386959438

H,0,0.1685787059,-0.8663052145,3.0327760599

H,0,-0.7482307277,0.5816053768,3.4444944954

H,0,0.6334656775,1.2317029125,-1.2029347602

H,0,1.6242288338,-0.1879386906,-1.5532816822

Cl,0,3.7186038958,0.2622251405,0.000469132

H,0,4.417385707,-2.4430478379,-1.6711449101

O,0,5.3471007893,-2.1594628501,-1.8408383824

H,0,5.7602350839,-2.0831160836,-0.9666794904

O,0,2.6826872091,3.5991918767,-0.943393116

H,0,2.8605693765,2.9162261138,-1.6281341746

O,0,3.6287932825,3.802102809,1.6982103671

H,0,4.1385805665,3.0073469424,1.9185806434

O,0,0.0332451512,4.4861956532,-1.2276365506

H,0,-0.5402011582,3.8504602051,-1.7110502418

H,0,-0.3154036273,4.5055874781,-0.3220683955

H,0,1.7405257682,3.8608213521,-1.0500787152

H,0,3.3408104683,3.6856702517,0.7644697478

O,0,5.5156912504,0.3660747174,-3.0127641303

H,0,6.117750008,0.8719231036,-2.4459428365

H,0,5.427485725,-0.5200024408,-2.5876935401

H,0,-4.8145011113,-0.7227104827,1.8589001699

O,0,1.2520035359,-1.4044876289,0.7963351729

H,0,1.7050435825,-1.8681247663,0.0400179332

H,0,0.6130449261,-2.5351775186,1.6289466608

O,0,-1.6845872643,-2.8804135261,3.609868114

H,0,-2.5141438641,-2.867693212,3.1019968007

H,0,-1.6046093456,-1.9976435539,4.0116224971

H,0,0.0840098276,-4.0750145996,1.4932405616

O,0,0.2918176069,-3.3690669501,2.1333618202

H,0,-0.5444959779,-3.1582133641,2.7304354864

O,0,3.1327690816,1.8208522784,-3.1007946035

H,0,3.9596984398,1.286771516,-3.0369692469

H,0,3.2379477453,2.3768527955,-3.8875245794

O,0,-1.8757536572,2.7505687153,-2.4478060396

H,0,-2.481131312,3.0144436826,-1.7195458334
H,0,-2.2101273759,3.1866326972,-3.2467118861
O,0,-3.2225175384,3.7476614646,-0.1325551876
H,0,-3.1063313078,3.0773934477,0.5614519215
H,0,-2.6341085064,4.4783622427,0.1173800571
H,0,-3.670146369,-2.0317017236,1.5097455003
H,0,-2.566238832,-0.2058217395,2.7704845378
H,0,-1.2175514663,-1.4488119805,1.099853116
C,0,-0.5018769776,1.9512211174,1.1326974259
H,0,-1.2507881722,2.2856251816,1.8613050788
H,0,0.3822272465,2.583673596,1.2739151723
H,0,-0.8896362627,2.1585128055,0.1312707532
Cl,0,2.4200366705,-3.2136615448,-1.3847677929

Dienogest

Cationic intermediate following chlorination at C4

SCF Done: E(RMN15) = -3123.42482886

Sum of electronic and thermal Free Energies= -3122.720263

C,0,-1.0849608014,-1.3645323248,-2.0598638929
C,0,-1.4615086475,-0.9718797672,-0.6789127895
C,0,-0.5741070795,-0.5608051167,0.2917161144
C,0,0.8044756572,-0.4788571206,-0.0471295616
C,0,1.2811459969,-1.0287459077,-1.3539598305
C,0,0.2639172799,-0.7965571109,-2.4703731821
H,0,0.1767550775,0.2810285338,-2.6629939333
H,0,0.6165829625,-1.2776480544,-3.3874936335
H,0,1.2971845134,-2.1237372146,-1.1629286771

C,0,2.6958054694,-0.59078184,-1.7109792382

H,0,2.6436804804,0.4737515355,-1.9966722826

C,0,3.6619356797,-0.7047728606,-0.5190693056

C,0,3.1909208753,0.3142616063,0.51575191

C,0,1.7666109022,-0.0009688123,0.9734812483

H,0,1.7695132662,-0.8093413163,1.7284104398

H,0,1.3305724133,0.8543523574,1.5063920023

H,0,3.8401690348,0.3533773129,1.3985943618

H,0,3.1839535749,1.3143134782,0.0621229165

C,0,5.0271130988,-0.4483054229,-1.211106552

C,0,4.8891470913,-1.192515043,-2.5661024571

C,0,3.3775014235,-1.3768838569,-2.8360502041

H,0,3.0949609854,-2.4358844538,-2.7706248955

H,0,3.0830981879,-1.0205512864,-3.8273897698

H,0,5.3884080478,-0.6341596125,-3.3649992069

H,0,5.3990874148,-2.1574475352,-2.4740642172

O,0,6.079368418,-0.979917562,-0.4067172297

H,0,6.9320631348,-0.8604711464,-0.8583593704

C,0,5.3470919145,1.0400962571,-1.4778132793

H,0,6.2261350117,1.0901437562,-2.1324883482

H,0,4.5240226023,1.5349026289,-2.0052106155

C,0,5.6686818041,1.7889908942,-0.2623176004

N,0,5.952440882,2.3757447799,0.7028756388

C,0,3.6835794218,-2.1254662685,0.0736296531

H,0,4.3857149277,-2.1760957381,0.9110698566

H,0,2.7024443945,-2.4287524324,0.4551593903

H,0,3.9931449413,-2.8688795657,-0.6704195334

C,0,-1.1094294126,-0.1979271592,1.6598037912

C,0,-2.1950613874,-1.1893174041,2.0877754886

C,0,-3.2970452923,-1.2594179493,1.0813192093

C,0,-2.9283124769,-0.9556265498,-0.3669953824

H,0,-3.5078691339,-1.5881153516,-1.0446504046

Cl,0,-3.4261631525,0.7538053583,-0.6860233363

O,0,-4.4651848114,-1.512881851,1.3595901123

H,0,-1.7620512803,-2.2019372084,2.1424408203

H,0,-2.6196972594,-0.9505027262,3.0667635928

H,0,-1.5209844994,0.8218759207,1.6391922842

H,0,-0.3157936832,-0.2060755452,2.4103065828

H,0,-1.0559497214,-2.4713835451,-2.0530122773

H,0,-1.8891538228,-1.0843176241,-2.7512732903

H,0,-5.5061999777,2.9887617825,-0.0606427875

O,0,-6.1168447285,2.5506377008,0.5701168852

H,0,-5.5588058398,2.2532250712,1.3196051351

O,0,-5.131051834,-3.4390334515,-0.8653360143

H,0,-5.7748848855,-4.1650349697,-0.8610863211

H,0,-5.3479263873,-2.8771110035,-0.0985988409

O,0,-2.5401366304,-3.9723540714,0.1479024399

H,0,-2.7435355148,-4.1272963668,1.0839598177

H,0,-3.4109847964,-3.8773458007,-0.2970985861

O,0,0.349827141,-3.962982183,0.3308002022

H,0,0.5873258714,-3.5661292614,1.195285729

H,0,-0.6158626123,-3.8595001482,0.2292760767

O,0,-6.7034626779,-0.1420530857,-0.1462105568

H,0,-6.5139554596,0.8091794324,0.0200954457

H,0,-6.0225813532,-0.631229286,0.3513491909

O,0,-1.3961213185,2.6944457217,-2.6693844647

H,0,-2.210259368,3.0735636092,-2.2681623921

H,0,-1.0637660473,3.3748466005,-3.2753278147

O,0,0.6373458695,2.2299125867,-0.7830297589

H,0,0.3423345923,2.4942262327,0.1159635106

H,0,-0.1143421386,2.4005220637,-1.3966866952

H,0,3.5254701347,3.4440019105,-1.7347839721

O,0,2.8628520672,3.8479154547,-1.1547175195

H,0,2.0853584434,3.2395243675,-1.1484397928

O,0,-5.7404749127,-1.2086303029,-2.6447637085

H,0,-6.0724347993,-0.6929526636,-1.8794722582

H,0,-5.4841775057,-2.0632814511,-2.2474247358

O,0,1.1276110545,-2.9618994077,2.9088667582

H,0,2.0083186056,-2.5517068156,3.0490885964

H,0,0.4819586559,-2.2854319655,3.1696669571

O,0,3.5342781993,-1.4839464131,3.5638975202

H,0,4.3747069332,-1.2688854557,3.1000533029

H,0,3.093512218,-0.6286174228,3.6891898208

O,0,-0.0995886038,3.11209875,1.8765912595

H,0,-0.0343532719,2.4002257934,2.5330191066

H,0,-1.0691320837,3.2578672391,1.7414119837

O,0,-2.8166833957,3.4393974794,1.5394602682

H,0,-3.1345217881,3.6169398214,0.626293337

H,0,-3.318294013,2.6626872472,1.8586131736

H,0,2.9847757725,3.6555454532,0.7601055323

O,0,2.7708618742,3.5683444464,1.7117620203

H,0,1.7957764667,3.5371871187,1.7459781219

H,0,6.0468510702,-0.7277267386,1.4404795282

O,0,6.0336587121,-0.6343938307,2.4185344608

H,0,5.9978549334,0.32257788,2.5776922008

O,0,-4.5340748836,1.379343763,2.6731644303

H,0,-4.8316971639,1.6394300415,3.5593371327

H,0,-4.7432835535,0.4337397966,2.5861961089

Cl,0,-3.9943873236,4.0318828656,-1.3684756104

TS for deprotonation at C4

SCF Done: E(RMN15) = -3123.40944596

Sum of electronic and thermal Free Energies= -3122.706533

C,0,1.2162868655,-0.2292511553,-2.7060778471

C,0,1.685611032,0.1713834463,-1.3457487

C,0,3.0972538816,0.4411404803,-1.1686231673

C,0,3.5910468138,0.7450165375,0.1972390048

C,0,2.6187960181,1.5395883007,1.0133114028

C,0,1.2709092394,0.8211960224,1.0791388282

C,0,0.7736676623,0.4136300158,-0.2923019712

C,0,-0.5930103913,0.2356371943,-0.5132345582

C,0,-1.12534064,0.0612193039,-1.9105060387

C,0,-0.1796358564,-0.8212217252,-2.7171302767

H,0,-0.1676102976,-1.830376434,-2.2808598569

H,0,-0.5395298138,-0.9059114908,-3.7474132126

H,0,-1.085763036,1.0774713565,-2.3539748372

C,0,-2.5662414133,-0.4292869739,-1.9627295943

H,0,-2.54553922,-1.5070201777,-1.7235455281

C,0,-3.4740808024,0.2466501489,-0.9238828574

C,0,-2.9505014468,-0.178797256,0.4452464813

C,0,-1.5261798908,0.3465590056,0.6504571846

H,0,-1.5549285084,1.423245479,0.8977100035

H,0,-1.066896528,-0.1252606261,1.5303452804

H,0,-3.5810918234,0.183455493,1.2660888215

H,0,-2.9288394194,-1.2763548576,0.5019885655

C,0,-4.8682697202,-0.2732925295,-1.3617618725

C,0,-4.7931440888,-0.245083412,-2.9126164924

C,0,-3.2951433838,-0.22721869,-3.2950524577

H,0,-3.013595222,0.7450723051,-3.720870793

H,0,-3.040930391,-0.9948688822,-4.0318162067

H,0,-5.320175609,-1.1055028395,-3.3383547878

H,0,-5.306933298,0.6573480384,-3.2607774634

O,0,-5.8829256738,0.5921767649,-0.8525294142

H,0,-6.7554499265,0.288632262,-1.1556622401

C,0,-5.1968625082,-1.7107649829,-0.9002961478

H,0,-6.0964471677,-2.046141335,-1.4319879069

H,0,-4.3892076405,-2.404078769,-1.1616491956

C,0,-5.4844200969,-1.8081602588,0.5310211313

N,0,-5.7499476802,-1.8798328462,1.6626709502

C,0,-3.4721859752,1.7812505963,-1.0553157911

H,0,-4.026781828,2.2300597986,-0.2222380382

H,0,-2.4580248312,2.1953742561,-1.0354878293

H,0,-3.9432553269,2.1085817252,-1.989000485

H,0,1.3512605553,-0.0638003072,1.7240276171

H,0,0.5496704962,1.4975153296,1.5498883802

H,0,2.4895201674,2.5088680216,0.5063491778

H,0,3.0347655789,1.7192064621,2.0085179157

O,0,4.726046379,0.4541916912,0.5771470043

Cl,0,4.2543624088,-0.460279312,-2.1580192584

H,0,1.2353312342,0.7100106584,-3.2917660217

H,0,1.9431124094,-0.8952803087,-3.1793994125

H,0,3.1243968068,1.6497974154,-1.670346142

O,0,3.2272585916,2.9007887215,-2.1148133104

H,0,3.6466989682,2.9162597883,-2.9931653091

H,0,3.8476147998,3.3499865239,-1.473392775

Cl,0,4.3357660553,-4.8960277893,-0.3415077147

H,0,5.3121300119,-3.0316797306,0.3405146339

O,0,5.8462051763,-2.2749225209,0.6688273438

H,0,5.3675048143,-1.4640199207,0.4207954468

O,0,2.1111058889,5.1429770017,0.2823962849

H,0,1.5222236756,4.6191048924,-0.305161096

H,0,3.0134527241,4.7864724486,0.1617788888

O,0,0.9962589159,4.1140327479,2.6238373697

H,0,0.9594647767,4.7999789212,3.3077906206

H,0,1.4682769529,4.5185387886,1.8569238886

O,0,0.1116245686,3.6914017128,-1.1217316026

H,0,-0.5176108341,3.5869211815,-0.3708448485

H,0,0.3319507139,2.8002118046,-1.4358885081

O,0,4.7347426863,3.9873096021,-0.1639592232

H,0,5.0698406099,3.3046900521,0.4409976284

H,0,5.4970594963,4.5338712002,-0.4131026491

O,0,2.0185838895,-2.9039772076,-0.7844585619

H,0,2.7231325287,-3.586531053,-0.6463247532

H,0,1.9582051652,-2.7810387964,-1.7451068148

O,0,-0.5202312806,-2.7342063141,0.4237289796

H,0,-0.3671969136,-2.3622323758,1.3141441191

H,0,0.3669384283,-2.8618171223,0.0200098294

H,0,-3.5175452422,-4.0561658567,0.1779926384

O,0,-2.8419820845,-4.2045371346,0.8565975672

H,0,-2.0232858993,-3.7616651065,0.5379719279

O,0,-1.4392046269,3.9671328872,1.1836434685

H,0,-2.1469252545,3.4637318143,1.642536871

H,0,-0.6715220921,4.0001510608,1.79706814

O,0,-3.3644828751,2.5185732211,2.6906024256

H,0,-4.2186661223,2.1795601626,2.3349173075

H,0,-2.858416601,1.7261871502,2.9309930557

O,0,0.1536806788,-1.9304924815,3.2543492371

H,0,0.0938367118,-0.9785178912,3.431735482

H,0,1.0527347399,-2.0787609993,2.8718181985

O,0,2.6204186082,-2.3064888524,2.016257106

H,0,2.5327184232,-2.0858619603,1.0701030144

H,0,3.2359143143,-1.6482294875,2.4126495304

H,0,-2.8932327281,-3.0156265833,2.3788688907

O,0,-2.6977642513,-2.4288080093,3.1382046343

H,0,-1.7235500854,-2.3634443107,3.1666861304

H,0,-5.8240798289,1.1609053525,0.9121221563

O,0,-5.8383451198,1.4472880773,1.8524693551

H,0,-5.8660039317,0.6211808773,2.362565286

O,0,4.3269114173,-0.4534330233,3.2983554019

H,0,3.7404430279,0.1858487402,3.7330572605

H,0,4.6710843185,0.0066968566,2.5083787003

C4-Cl product complex

SCF Done: E(RMN15) = -3123.43554358

Sum of electronic and thermal Free Energies=   -3122.735845

C,0,2.1116447819,1.0471442683,0.5757344214

C,0,2.4791738722,0.0900489564,-0.5221683621
C,0,3.7860376307,-0.0304700522,-0.923594768
C,0,4.267840221,-1.1546161929,-1.7117274344
C,0,3.2242360537,-2.1186113797,-2.2010915248
C,0,1.8642218814,-1.4569700223,-2.4001938758
C,0,1.445846411,-0.7316073684,-1.1438228668
C,0,0.1990941006,-0.8135857289,-0.5943659748
C,0,-0.0933212736,-0.0923427362,0.7120483273
C,0,0.6157659135,1.259301835,0.7170828821
H,0,0.2277091952,1.8804969175,-0.1067263476
H,0,0.4137668013,1.7873833026,1.6569783784
H,0,0.3475425394,-0.6905738335,1.5316319579
C,0,-1.5830698978,0.0286257895,0.9973552108
H,0,-2.0013258626,0.7636560904,0.2896592872
C,0,-2.3292894635,-1.2956785426,0.7651588621
C,0,-2.301407884,-1.5432952972,-0.743041171
C,0,-0.859475887,-1.6749519351,-1.2593262564
H,0,-0.5381202658,-2.7206448571,-1.1629051175
H,0,-0.8531024689,-1.4728812492,-2.3375363741
H,0,-2.8577170646,-2.4475695225,-1.0222847188
H,0,-2.7805921936,-0.6928271487,-1.2507711545
C,0,-3.7102521088,-0.9910891888,1.408236621
C,0,-3.3627395828,-0.131805985,2.6513321905
C,0,-1.9542041061,0.4581037484,2.421126238
H,0,-1.2328036649,0.0392777348,3.1356197203
H,0,-1.9369284395,1.5473178376,2.5413786886
H,0,-4.1199787712,0.6444482086,2.8074597895
H,0,-3.3816842922,-0.7881645104,3.5283246137

O,0,-4.3576590284,-2.2113568673,1.7738328063

H,0,-5.1931289953,-2.0151033838,2.2303564104

C,0,-4.6679014145,-0.1992631824,0.4886770355

H,0,-5.4845544345,0.2125175537,1.0964903181

H,0,-4.1574610134,0.6478103656,0.0146282682

C,0,-5.2815551127,-1.0276219149,-0.5488453571

N,0,-5.7936340633,-1.6974159236,-1.352726557

C,0,-1.6748000336,-2.4592564745,1.5279456978

H,0,-2.2393817776,-3.3843839926,1.3758881634

H,0,-0.6500179504,-2.6407542675,1.1832807025

H,0,-1.6335551264,-2.2612781827,2.606710461

H,0,1.931388286,-0.7373681646,-3.2292458128

H,0,1.1368014038,-2.2194426577,-2.6853212797

H,0,3.1508215807,-2.9068382629,-1.4354656313

H,0,3.5950361125,-2.5860896796,-3.1178402984

O,0,5.4792537701,-1.3599796963,-1.9065829417

Cl,0,5.0042688117,1.090123456,-0.3682924544

H,0,2.5059017848,0.6120400808,1.511238703

H,0,2.646654875,1.9900528834,0.4335944129

H,0,5.682902073,-1.0635683892,1.8300875842

H,0,4.0843235029,2.1091065176,2.4725641522

O,0,4.0053079522,3.0697562506,2.5934921818

H,0,4.0351027311,3.2159476305,3.5516708609

O,0,-0.7519533413,4.4431933809,1.5300707467

H,0,-1.0721462628,3.5562736544,1.7619625467

H,0,0.2360371843,4.3975850194,1.5062050482

O,0,1.9750275085,4.4958137729,1.1528797056

H,0,2.1120811406,4.2308078891,0.2202787004

H,0,2.619231597,3.9746223785,1.6784827337

O,0,6.6110504079,-1.7038817345,0.6636710175

H,0,7.564291447,-1.6314118544,0.8330979104

H,0,6.4417421772,-1.3714760587,-0.2444183499

O,0,4.7321766305,-3.8372707719,0.6323262473

H,0,4.9489222558,-4.5719371015,1.2267436384

H,0,5.4963538012,-3.2259289039,0.6678168292

O,0,2.2992543541,-2.7860593635,1.5434463805

H,0,2.3192355693,-1.8154652625,1.5359354722

H,0,3.1922342846,-3.0794106792,1.2456306147

O,0,-2.9294377624,5.7860482306,0.3338765611

H,0,-2.1052835436,5.5307461336,0.7986792378

H,0,-2.6483420163,6.1254583536,-0.5300634565

O,0,-3.5325497771,3.0228042919,0.0921389481

H,0,-2.9839470948,2.6448958825,-0.6239998675

H,0,-3.3615666878,3.9912945423,0.0920145515

H,0,-6.5627567481,1.8307590848,-0.430270363

O,0,-6.1031880453,2.5077408574,-0.9507513506

H,0,-5.2693680477,2.7072072568,-0.4656252481

O,0,1.0658080395,-4.578395126,-0.3168843935

H,0,0.0909073507,-4.6557773652,-0.3266504772

H,0,1.2961936303,-3.8583582907,0.3054821332

O,0,-1.7442306199,-5.1184515374,-0.766960471

H,0,-2.6416546188,-4.8389114258,-0.4747798008

H,0,-1.7833382639,-6.0827180453,-0.8550411436

O,0,-1.8010455498,1.9664461789,-2.1491019416

H,0,-1.2756703414,1.2214938604,-1.8150634953

H,0,-1.2912570728,2.7882231625,-1.9345095186

O,0,-0.5405864462,4.2890378282,-1.397974804

H,0,-0.6899443401,4.3281095342,-0.430677051

H,0,0.4205785495,4.1213859214,-1.5096737671

H,0,-5.0657592254,1.6221160928,-2.2933797456

O,0,-4.4405786429,1.2332593651,-2.9396857789

H,0,-3.5498265374,1.5183716404,-2.6518322011

H,0,-4.3835564352,-3.6332479627,0.5855604592

O,0,-4.3966150232,-4.385606913,-0.0470767338

H,0,-4.7694094543,-4.0130589367,-0.8627652552

O,0,2.2271459268,3.6990198857,-1.6272491884

H,0,2.7220367148,4.2128395746,-2.2848150019

H,0,2.3903429403,2.7643429287,-1.8345506864

Cl,0,4.9030552801,-0.671748206,2.8474659398

TS for C4-C9 chlorohydrin formation

SCF Done: E(RMN15) = -3123.42312174

Sum of electronic and thermal Free Energies=   -3122.714662

C,0,-1.0029755889,-1.0052304088,-2.2559926866

C,0,-1.3678029066,-0.8888454072,-0.8135561863

C,0,-0.503736597,-0.5970650576,0.1984904008

C,0,0.8867503853,-0.353950784,-0.1308713723

C,0,1.365129829,-0.7301772012,-1.502339255

C,0,0.3488501632,-0.3865689293,-2.5859758212

H,0,0.2646826454,0.7036774292,-2.6719732918

H,0,0.7072299834,-0.7654337914,-3.5481148476

H,0,1.3917803978,-1.8372838567,-1.4293714168

C,0,2.7802256803,-0.2478009765,-1.7934267791

H,0,2.7389304584,0.8518814482,-1.8761288461

C,0,3.7624216398,-0.5925749827,-0.6582175404

C,0,3.296606542,0.1826311896,0.572581219

C,0,1.8835818236,-0.243872963,0.9704795104

H,0,1.8902054498,-1.2600974615,1.4031274036

H,0,1.4991262715,0.3982669346,1.7700318386

H,0,3.9594849029,0.0409739779,1.4346082571

H,0,3.2719465721,1.255484555,0.3418725556

C,0,5.1155634554,-0.1861275343,-1.3004504206

C,0,4.9600075245,-0.6315927015,-2.7806077067

C,0,3.4477879133,-0.8113208847,-3.0510110873

H,0,3.1923885843,-1.873428341,-3.162205903

H,0,3.122576932,-0.2988587782,-3.9610617915

H,0,5.4126264739,0.1062520208,-3.4516246093

H,0,5.5068912487,-1.5713414612,-2.9126778216

O,0,6.1832016037,-0.8662020072,-0.639790609

H,0,7.0264919527,-0.657355383,-1.0761635627

C,0,5.4223344282,1.3267629077,-1.2549367558

H,0,6.2897029344,1.522903221,-1.8976098517

H,0,4.5846228372,1.9099550847,-1.6512537176

C,0,5.7570764569,1.810457132,0.0847162227

N,0,6.052010553,2.188138595,1.1462567801

C,0,3.8178731725,-2.1040651776,-0.3668658342

H,0,4.4946928171,-2.3001534661,0.471036852

H,0,2.8390661757,-2.5131060456,-0.095987993

H,0,4.1842146581,-2.6694222904,-1.2314766893

C,0,-1.0177763041,-0.5393400659,1.6203246934

C,0,-2.030890336,-1.6599584935,1.870309864

C,0,-3.146846562,-1.62365498,0.8783182527

C,0,-2.8270780932,-1.0528288356,-0.4987555821

H,0,-3.3607301448,-1.6181240911,-1.2687634063

Cl,0,-3.510968297,0.6227972741,-0.5152358157

O,0,-4.2886014989,-2.0036193369,1.124043916

H,0,-1.53366037,-2.6348404677,1.7323403574

H,0,-2.4485302584,-1.6338336754,2.8806756897

H,0,-1.4868800803,0.4376797929,1.8092219342

H,0,-0.2047346367,-0.6447522591,2.3424018554

H,0,-1.0016430577,-2.0868507843,-2.4818752269

H,0,-1.8077882356,-0.5713590141,-2.8638993615

O,0,0.7570487985,1.812667246,-0.3651752435

H,0,0.5355985986,2.1010440704,0.5578434496

H,0,-0.0298962126,2.0670548026,-0.9134361206

Cl,0,-4.0403362004,3.9665768956,-0.7411824377

H,0,-5.477245842,2.7304011663,0.5019057589

O,0,-6.044697443,2.2008132532,1.1019758119

H,0,-5.4389236963,1.8151644049,1.7698825228

O,0,-4.9776168779,-3.4929849467,-1.3558371752

H,0,-5.6056274901,-4.2285942273,-1.4342659547

H,0,-5.1588606692,-3.0680708373,-0.4963290236

O,0,-2.3555229242,-4.2032521097,-0.4895663966

H,0,-2.5494770647,-4.4469185025,0.429477198

H,0,-3.2268783509,-3.9972496216,-0.8930366331

O,0,0.5182771589,-3.9345816634,-0.2724306357

H,0,0.7536479289,-3.7112524267,0.6528235929

H,0,-0.455101722,-3.8881943462,-0.3387118836

O,0,-6.6859491942,-0.4175771756,0.1166801478

H,0,-6.4692689828,0.5044443814,0.3811759639

H,0,-5.9578402072,-0.9656174146,0.462659075

O,0,-1.3309214314,2.7134318747,-1.962019492

H,0,-2.1660061143,3.0347334445,-1.5538253574

H,0,-0.9523143097,3.4822642226,-2.4168165541

H,0,3.3399506515,3.7442162046,-1.5025957757

O,0,2.7576135058,3.7958805612,-0.7300033957

H,0,2.1327350079,3.0414770575,-0.793263679

O,0,-5.8791022151,-1.0263052369,-2.6092767623

H,0,-6.1598136446,-0.6614515553,-1.7442534645

H,0,-5.5244649048,-1.907988531,-2.3824372398

O,0,1.3176696449,-3.4465412551,2.4459514988

H,0,2.2011552454,-3.0590739333,2.6293379437

H,0,0.6783470031,-2.8004138194,2.7875993285

O,0,3.7138399266,-2.055871342,3.2554265103

H,0,4.5503859586,-1.7874264164,2.8129592029

H,0,3.2546726369,-1.2216837378,3.4433906459

O,0,0.0460853726,2.6500423265,2.2152255121

H,0,0.1217483864,1.9005445053,2.8276853543

H,0,-0.9257960906,2.8077083863,2.1077596896

O,0,-2.6759727541,2.9919399578,1.9669845156

H,0,-3.0477048924,3.2960003786,1.108813205

H,0,-3.1582856345,2.173604314,2.1993669303

H,0,3.0444457578,3.5001937866,1.1733658609

O,0,2.8683474665,3.3472900357,2.1231150828

H,0,1.8998399624,3.2395680987,2.1803278955

H,0,6.1895159338,-0.9692714406,1.2255753628

O,0,6.1989228164,-1.0569956575,2.204110174

H,0,6.1785856899,-0.1444968089,2.5352800287

O,0,-4.334061141,0.778589256,2.9316508837

H,0,-4.570130317,0.9144774452,3.8630085752

H,0,-4.5643366232,-0.144080739,2.7310156896

C4-C9 chlorohydrin

SCF Done: E(RMN15) = -3123.44092638

Sum of electronic and thermal Free Energies= -3122.728824

C,0,-1.0698164753,-0.8379235031,-2.3009383216

C,0,-1.4728446762,-0.7649584583,-0.8514166007

C,0,-0.657119286,-0.459587183,0.1740536054

C,0,0.7591916543,0.0131773143,-0.1164595428

C,0,1.2759754408,-0.6121755334,-1.4288485437

C,0,0.3340971124,-0.3018716418,-2.5873815194

H,0,0.3173233973,0.782755197,-2.7631859544

H,0,0.719670562,-0.7585251493,-3.5058828224

H,0,1.2514378449,-1.7001494709,-1.2682652262

C,0,2.7159876665,-0.1917259366,-1.6939273499

H,0,2.726490294,0.9076908682,-1.772358776

C,0,3.668256902,-0.5628374323,-0.5414239572

C,0,3.17364321,0.1627886996,0.7092449044

C,0,1.7353341264,-0.2486148164,1.0334809012

H,0,1.6765828154,-1.3161026484,1.2775951172

C,0,5.0312167565,-0.1195894498,-1.1333912884

C,0,4.9155153607,-0.5019656514,-2.6370135946

C,0,3.4170818343,-0.7438171863,-2.9367321139

H,0,3.2013447902,-1.8150022316,-3.0458780168

H,0,3.093122165,-0.2484351835,-3.8571873833

H,0,5.3303502341,0.2939483808,-3.2653529451

H,0,5.517267387,-1.4008283078,-2.810158435

C,0,3.7326461976,-2.0838114587,-0.303571965

H,0,4.3418313296,-2.301750044,0.5799614298

H,0,2.7426760511,-2.5188018505,-0.1343359537

H,0,4.1844683542,-2.6077203944,-1.1536194177

C,0,-1.1910386479,-0.4303525892,1.5878946333

C,0,-2.2390726272,-1.5231278782,1.8065013143

C,0,-3.318069225,-1.4794165097,0.7771012108

C,0,-2.9401185598,-0.9400352185,-0.5958268832

H,0,-3.4442427232,-1.5262603372,-1.3703834379

O,0,-4.4712898853,-1.8495165378,0.9959092157

H,0,-1.7558542906,-2.508022769,1.6827702883

H,0,-2.6905547485,-1.4927953843,2.8025407777

H,0,-1.6252594399,0.5568496675,1.8069182251

H,0,-0.3896868178,-0.5813144737,2.3157393176

H,0,-1.1362609221,-1.8973443099,-2.5991320592

H,0,-1.8205894248,-0.309528065,-2.9057793135

Cl,0,-3.6947184204,0.7212973273,-0.6631550003

H,0,-5.163132056,3.1484438548,0.3659765494

O,0,-5.8075650571,2.7299560347,0.9708141166

H,0,-5.2709250235,2.283800394,1.6602603565

O,0,-5.2332064593,-3.3159427878,-1.3848830338

H,0,-5.8743430288,-4.0429262324,-1.3423988022

O,0,-2.5913816148,-4.1940500379,-0.5756302416

H,0,-2.8102402501,-4.3620575008,0.3548357144

O,0,0.2900457493,-3.9419667204,-0.2214147919

H,0,0.4953524139,-3.7071189164,0.708079179

H,0,-0.6766544199,-3.8578999383,-0.3300364668

H,0,-5.2718308544,-2.8656759099,-0.5179619604

H,0,-3.4339244706,-3.9091379532,-0.9881375337

O,0,-6.8420459591,0.0763054499,0.3448539937

H,0,-6.4899366147,0.9859986207,0.4522650155

H,0,-6.1326803079,-0.5151546139,0.6533088232

H,0,3.8159646589,-0.0358709541,1.5762080992

O,0,-1.076492681,2.624020911,-1.7392463256

H,0,-1.8536147232,3.1417158201,-1.3335029373

H,0,-0.5039543833,3.2353238626,-2.2452816889

O,0,0.664163494,1.463364649,-0.2991067459

H,0,0.6001896983,1.903576499,0.5933276282

H,0,-0.4707849768,2.0978004551,-1.0709882714

H,0,2.749476449,3.2478965569,-2.3804714178

O,0,2.0027444202,3.4916683405,-1.8108011419

H,0,1.6787255681,2.6530829794,-1.4235788624

O,0,-6.4403872456,-0.8606912584,-2.3991357649

H,0,-6.5709896593,-0.4483802054,-1.5203382572

H,0,-6.0216050605,-1.7225929297,-2.2068371472

O,0,1.0021498672,-3.446909029,2.5247634357

H,0,1.8879925965,-3.0599624583,2.6945619802

H,0,0.3678219075,-2.7673356758,2.8053004196

O,0,3.4514154634,-2.1307481811,3.3458362719

H,0,4.3081009383,-1.9088097662,2.9183210154

H,0,3.0197454411,-1.2735476598,3.4918286033

H,0,3.1835796126,1.2490124984,0.5342183819

H,0,1.4076535988,0.2964438978,1.9269597907

O,0,6.0913416365,-0.820857934,-0.4789539736

H,0,6.9424716194,-0.5821490851,-0.8835643372

C,0,5.3218990041,1.3944005954,-0.9981379884

H,0,6.1704529454,1.6475138684,-1.646222038

H,0,4.4681664283,2.0024923248,-1.3237700668

C,0,5.6909415179,1.7764183401,0.3653470686

N,0,6.0138016073,2.0716047947,1.4449139341

O,0,0.3155572022,2.7556797145,2.0869837161

H,0,0.5144113289,2.1630751809,2.8294521993

H,0,-0.6670304216,2.882209752,2.087884177

O,0,-2.4175352605,3.1138704169,2.0121499129

H,0,-2.737404645,3.457150989,1.1498304483

H,0,-2.9925127808,2.3550225741,2.2356073364

H,0,2.5986018211,3.9151061752,0.0082817641

O,0,2.7494389384,3.9970117575,0.9710726164

H,0,1.9409361678,3.6390410599,1.3851948188

H,0,6.0376728102,-1.0628463531,1.3866971428

O,0,6.0206367428,-1.2231606333,2.3557160583

H,0,6.0229645682,-0.3378237437,2.7538927721

O,0,-4.3626375744,1.1130533347,2.849003765

H,0,-4.6226136252,1.2540031461,3.7732796315

H,0,-4.6330445351,0.2054745137,2.6312572616

Cl,0,-3.4073398438,4.2340922656,-0.7976017214

C9-C10 epoxide product complex

SCF Done: E(RMN15) = -3123.43041050

Sum of electronic and thermal Free Energies= -3122.724815

C,0,-1.1392303434,-0.770254186,-2.1909943525

C,0,-1.5414808873,-0.9837875422,-0.7560528719

C,0,-0.5466162183,-0.8433049915,0.3309118048

C,0,0.8436953527,-0.5728692573,-0.0113500729

C,0,1.26631922,-0.6657377298,-1.4895928285

C,0,0.1926619882,-0.0604142662,-2.3916150352

H,0,0.1012535575,1.0154499533,-2.1885614739

H,0,0.49643803,-0.1642345425,-3.439025223

H,0,1.3726372103,-1.7315984548,-1.7413459339

C,0,2.6084737799,0.0434676294,-1.6643420652

H,0,2.4386277066,1.1020835777,-1.40129258

C,0,3.7110889728,-0.472633868,-0.7233591762

C,0,3.2386777807,-0.2298547743,0.7095010904

C,0,1.9371008089,-0.9918414679,0.9592973459

H,0,2.0893247572,-2.0705652054,0.8401845143

C,0,4.9333783435,0.3442216015,-1.2154281266

C,0,4.7312147305,0.4092511479,-2.756661652

C,0,3.2653037406,0.0085220553,-3.045796248

H,0,3.2061552434,-1.0069729024,-3.4596552909

H,0,2.7832988011,0.6832514785,-3.7598318723

H,0,4.9551426166,1.4154502139,-3.1275787677

H,0,5.443128341,-0.2775869076,-3.2272263844

C,0,4.013662133,-1.9653694694,-0.9608787135

H,0,4.6618634669,-2.3539334026,-0.1669108379

H,0,3.1033826957,-2.5726630364,-0.9710594717

H,0,4.5267673957,-2.1245877685,-1.9157195803

C,0,-1.0290226703,-1.0804544987,1.7432809131

C,0,-2.080596889,-2.1882316076,1.8295904087

C,0,-3.1794944009,-1.9428400498,0.8413318134

C,0,-2.7877036391,-1.4276797511,-0.4674284951

H,0,-3.5314732816,-1.4863277877,-1.2601018141

O,0,-4.3673992152,-2.2173254979,1.0890068505

H,0,-1.6350030947,-3.1630846812,1.5808993665

H,0,-2.4977280116,-2.2524778277,2.8376053859

H,0,-1.486453365,-0.1445412127,2.0883815191

H,0,-0.2006658142,-1.3033040386,2.4175519842

H,0,-1.061423976,-1.779297316,-2.6251790254

H,0,-1.9579166546,-0.2659737743,-2.7168936663

Cl,0,-3.2013286654,2.2481442251,-1.4090712468

H,0,-5.2739627347,2.5274900903,-0.5623054791

O,0,-6.1580449013,2.4151343871,-0.1530915876

H,0,-6.0355045141,2.6313168562,0.784756263

O,0,-4.9634333215,-3.6306017343,-1.5020913865

H,0,-5.6740449167,-4.2871600162,-1.5729415972

O,0,-2.5688257822,-4.8541056355,-0.4801726408

H,0,-2.7833210093,-4.9121072172,0.4642948064

O,0,0.1723686063,-3.886135032,-0.408536398

H,0,0.4747725126,-3.9566145378,0.5208824959

H,0,-0.7674764767,-4.1610505361,-0.4203710761

H,0,-5.1530611132,-3.1151115615,-0.6950012605

H,0,-3.3490933304,-4.4253093697,-0.8945113332

O,0,-6.1499683847,-0.4095460635,-0.4778194452

H,0,-6.1202712064,0.5459425819,-0.2514343779

H,0,-5.5515475087,-0.8813345069,0.1316863913

H,0,3.9922933612,-0.5319731924,1.4463347116

O,0,-1.2597393227,4.4294959542,-0.6983479441

H,0,-1.4614829949,4.7787103566,0.2306041081

H,0,-0.3316806354,3.9616370631,-0.6947313511

O,0,0.2214292465,0.6523584169,0.2798365976

H,0,0.5182457099,1.2570297626,1.8949411005

H,0,-1.9575898632,3.7422000335,-0.9458527934

H,0,1.4255784981,3.2022619967,-1.4828587501

O,0,0.9582563237,3.213943804,-0.6301711989

H,0,0.7300574349,2.2788486858,-0.4225092265

O,0,-5.28388376,-1.1535087522,-3.0858307013

H,0,-5.5831958122,-0.7608314487,-2.2367787872

H,0,-5.1046686409,-2.0820834209,-2.8452644712

O,0,1.2074802769,-4.1016823402,2.2997273154

H,0,2.1472271705,-3.833891461,2.3001208415

H,0,0.7425045687,-3.4001949224,2.7837974634

O,0,3.9918698838,-2.9786615015,2.7182757417

H,0,4.7708929816,-2.5719469412,2.2808301261

H,0,3.5069549012,-2.2268231218,3.0953940857

H,0,3.0383796826,0.8439831502,0.8596339191

H,0,1.617282611,-0.8213440745,1.9930142263

O,0,6.1359595524,-0.3382049752,-0.8528036771

H,0,6.9055238749,0.1321158096,-1.2153337345

C,0,5.0170356951,1.7774685419,-0.6356015123

H,0,5.7498725314,2.349662799,-1.2184029811

H,0,4.0568071176,2.3043097411,-0.7080978857

C,0,5.4674184621,1.7901961585,0.7564601605

N,0,5.8545127427,1.7940745967,1.8548628797

O,0,0.5617415471,1.6668219193,2.7970198938

H,0,0.8098719917,0.9524532401,3.40748928

H,0,-0.8825911776,2.134066326,3.0017975148

O,0,-1.8376246066,2.5111682821,3.0874981716

H,0,-1.8364327164,3.4766821217,2.796907571

H,0,-2.5098540264,1.9980176804,2.5038390352

H,0,2.152072315,3.5671886735,0.9030149442

O,0,2.5816734575,3.5433174228,1.7795013346

H,0,2.019848701,2.9365390531,2.2985494232

H,0,6.2756734781,-1.132303758,0.87657725

O,0,6.3583376146,-1.5203961822,1.7744550063

H,0,6.2751066905,-0.7620968793,2.3755686263

O,0,-3.6200534064,1.4189548997,1.6114235376

H,0,-3.9500384111,0.5210510735,1.7765127025

H,0,-3.4480253184,1.5017245291,0.6447417398

Cl,0,-1.8216142151,5.3464913079,2.0892377006

β-trenbolone

Cationic intermediate after chlorination at C4

SCF Done: E(RMN15) = -2608.91775035

Sum of electronic and thermal Free Energies= -2608.372667

C,0,-0.9332052325,-0.7761524677,1.1153087314

C,0,-1.1178738354,0.0000760729,-0.1393341119

C,0,-0.095210401,0.5220804523,-0.8974822699

C,0,1.2510835577,0.3096796246,-0.4556301246

C,0,1.5036092197,-0.3570330758,0.8727748834

C,0,0.4434882318,-1.4130077182,1.1750055806

H,0,0.5123524232,-2.2253395745,0.4359918532

H,0,0.628137025,-1.8413970922,2.1655257373

H,0,1.388503101,0.4474574406,1.6260068306

C,0,2.9240331559,-0.8887417782,0.9245672377

H,0,3.0093557073,-1.6586195672,0.1377469297

C,0,3.9414258502,0.2043788663,0.5723858381

C,0,3.6423977692,0.6880818363,-0.7981544145

C,0,2.3517733993,0.7397696225,-1.2572164869

H,0,2.1679258351,1.1495895068,-2.2448309718

C,0,5.2680541929,-0.5314688652,0.7893260738

C,0,5.006021817,-1.3508794379,2.0725147502

C,0,3.4637494532,-1.471206158,2.2294600735

H,0,3.1092274354,-0.8805686981,3.0842005938

H,0,3.1378954779,-2.5031998949,2.3865570151

H,0,5.4905475336,-2.3287167536,1.9761098802

H,0,5.4547810522,-0.8517476522,2.9372598026

O,0,5.4845008331,-1.3712137567,-0.34820439

H,0,6.2287381464,-1.9697742899,-0.1761787046

H,0,6.1117474763,0.1619978999,0.8935061959

C,0,3.9041608114,1.4330704245,1.5133832109

H,0,4.7040106731,2.1296118461,1.2365047167

H,0,2.9484608109,1.966539637,1.452645676

H,0,4.0687458961,1.1255315886,2.5521016485

C,0,-0.4261787606,1.2442362871,-2.1871888483

C,0,-1.6998180255,2.0823584502,-2.0647487428

C,0,-2.8539667314,1.3273267543,-1.4897812476

C,0,-2.5311190723,0.1100872847,-0.6293351862

H,0,-3.2528165748,0.0316095961,0.1905505999

O,0,-4.0267724186,1.6267707078,-1.6793312798

H,0,-1.5147451108,2.9194806958,-1.3754671274

H,0,-2.001285522,2.512161855,-3.0238658245

H,0,-0.5317355849,0.5108928082,-2.9997781591

H,0,0.3808319501,1.9204215827,-2.4731129613

H,0,-1.0717729195,-0.0647734396,1.9497614268

H,0,-1.7340404471,-1.5218707735,1.2050238131

Cl,0,-2.7765759075,-1.3423961568,-1.6890926636

H,0,-3.942743317,-2.7470759598,1.0911425207

O,0,-4.6743906579,-2.0922112299,1.2061960311

H,0,-5.3757998083,-2.5654555171,1.6813180118

O,0,-5.1300512941,2.0272680832,1.5819558807

H,0,-5.8109070753,2.6431009311,1.891513361

O,0,-2.3868360566,2.452514705,1.1851161404

H,0,-2.2280715519,3.3191459428,0.7786585532

O,0,0.1559182859,2.4976220036,2.5156222891

H,0,0.556314876,2.8334596994,1.6851942085

H,0,-0.7780036472,2.3402273932,2.2734504492

H,0,-5.5660632621,1.378236506,0.9803246192

H,0,-3.3592256125,2.3855643372,1.3123422576

O,0,-5.9964931859,0.0442926451,-0.1747207129

H,0,-5.5078302686,-0.7304182875,0.1776085197

H,0,-5.4301516419,0.4349735779,-0.8680553365

H,0,4.4476070459,1.0688624634,-1.4280487637

O,0,-0.3518695922,-4.3252041797,-1.2178226888

H,0,-1.0050840705,-4.2223912551,-0.4846821453

H,0,-0.8107507808,-4.8229370998,-1.9119481769

O,0,1.0689491029,-2.2903441008,-2.4364442511

H,0,0.4755569358,-1.5922576077,-2.7534175998

H,0,0.5064820195,-2.9460864001,-1.9606372152

H,0,4.3310289775,-1.924741039,-1.7804023408

O,0,3.9034840766,-2.2099916348,-2.6113787101

H,0,2.9374963682,-2.1952559014,-2.4511566072

O,0,-3.8056154315,-0.0611175177,3.036949906

H,0,-4.0534236379,-0.7864249021,2.425120188

H,0,-4.2548182027,0.7298403616,2.6779976025

O,0,0.8979787416,3.5660418362,-0.0140204283

H,0,1.8064789335,3.7342342792,-0.3538293926

H,0,0.4502115687,4.4260992714,-0.0079546613

O,0,3.466326193,4.0345507765,-1.0727616678

H,0,4.1230026058,3.6228373857,-0.4876912711

H,0,3.4716880147,3.495204093,-1.8800431477

Cl,0,-2.3979623614,-4.2385764797,1.1473605346

TS for deprotonation at C4

SCF Done: E(RMN15) = -2608.90164537

Sum of electronic and thermal Free Energies= -2608.358925

C,0,0.2192432545,-1.4933742886,-0.3522500527

C,0,0.6332440341,-0.3645841761,0.5322325429

C,0,-0.2822589146,0.611470321,0.9676865748

C,0,-1.6286225697,0.5035264037,0.5776230551

C,0,-2.0552799848,-0.5054067345,-0.4652504521

C,0,-1.2645682093,-1.795867132,-0.291336257

H,0,-1.517847539,-2.255728565,0.6756179235

H,0,-1.5342157137,-2.5059694693,-1.0807103975

H,0,-1.7739993439,-0.0747739166,-1.4446013186

C,0,-3.5585361195,-0.7078594461,-0.425248752

H,0,-3.8018105706,-1.1582537709,0.5524263262

C,0,-4.3167927957,0.6253545578,-0.477453995

C,0,-3.8997628006,1.438423304,0.6983813672

C,0,-2.6243116889,1.3694578811,1.1699343338

H,0,-2.3391509825,1.9983557216,2.0074185616

H,0,-4.6050246659,2.1323438706,1.1551613429

C,0,-5.7702975467,0.1404930727,-0.5082007032

C,0,-5.7136030938,-1.0723186118,-1.4662097161

C,0,-4.2322890348,-1.5413884743,-1.5141799215

H,0,-3.7820311999,-1.3204676152,-2.4910791186

H,0,-4.1267296349,-2.6161952018,-1.3404079975

H,0,-6.383500741,-1.8535620607,-1.0902898941

H,0,-6.0733957237,-0.7895043056,-2.460870168

O,0,-6.1255145491,-0.2449421233,0.8216656491

H,0,-7.0114957884,-0.6402315746,0.8104670272

H,0,-6.4561546301,0.9228111556,-0.8590320867

C,0,-4.0472122677,1.4515632574,-1.7534913109

H,0,-4.60775422,2.3921749453,-1.7070333855

H,0,-2.9840772004,1.6946939263,-1.8607346653

H,0,-4.3718774229,0.9075459364,-2.6478975537

C,0,0.1900729461,1.7209321544,1.8870193486

C,0,1.6468463538,2.0917985148,1.6009019294

C,0,2.5205004477,0.8755148808,1.685249879

C,0,2.0567496613,-0.2148275789,0.7907881268

H,0,2.3500911069,0.2996355056,-0.3625030106

Cl,0,3.0172011336,-1.7006488658,0.9172779923

O,0,3.5529806238,0.8121318808,2.3468992251

H,0,1.718755951,2.4750271244,0.5696693184

H,0,2.0187878555,2.8573165836,2.2873113112

H,0,0.0705652588,1.4131988571,2.9352508299

H,0,-0.4136232423,2.6213254894,1.7425863646

H,0,0.5036774738,-1.1701115453,-1.3738010124

H,0,0.8273366274,-2.3755604449,-0.1487716316

Cl,0,2.3122283401,-4.2858637988,-1.810495112

H,0,3.2874401061,-2.3633206591,-2.1380523685

O,0,3.7501851319,-1.5286077239,-2.3963933038

H,0,4.0581257108,-1.6598033992,-3.3067931029

O,0,4.623622083,2.7663040856,-0.078499757

H,0,5.4568226938,3.2493274747,-0.1885330497

H,0,4.8239954175,1.8314083654,-0.3100833167

O,0,2.490517123,4.1124115561,-1.2719755109

H,0,2.8694732764,4.8006510735,-1.8394745941

H,0,3.2553967404,3.6185692631,-0.8923985275

O,0,0.1610600725,1.6176861577,-2.4118626699

H,0,0.2196153482,2.0312062212,-3.2873599859

O,0,5.4334136671,0.0849262248,-0.5846303781

H,0,4.9065505282,-0.507118718,-1.1604604698

H,0,5.3857239152,-0.3012048935,0.3045167076

O,0,0.5613757513,-4.6618376361,0.8121647974

H,0,1.146910791,-4.5581375965,0.0253123229

H,0,0.8400475836,-5.4829256308,1.245934943

O,0,0.2798428568,-2.6894818974,2.8110433364

H,0,1.1230922183,-2.2329230713,2.9536255847

H,0,0.4234245238,-3.3227031465,2.0702192963

H,0,-2.692809686,-1.2950129217,2.5367572882

O,0,-2.1380732258,-1.3287781634,3.3319863749

H,0,-1.3091040568,-1.7789600187,3.0581280157

O,0,2.5933555275,0.7958987947,-1.6018783584

H,0,1.7051407829,1.0890618082,-1.9535513553

H,0,2.8980399576,-0.0042157825,-2.100536886

O,0,0.6713761324,5.1527851891,0.5535659519

H,0,-0.546689831,4.101050213,-0.2900791237

H,0,1.4157975689,4.814997319,-0.0000344278

O,0,-1.1015123736,3.563932427,-0.9034423783

H,0,-0.2479038095,2.2966957674,-1.8208729651

H,0,-1.8489530767,3.225325428,-0.3839052145

H,0,0.8363146769,4.8344792459,1.454685608

C4-Cl product complex

SCF Done: E(RMN15) = -2608.93741830

Sum of electronic and thermal Free Energies= -2608.387541

C,0,0.3944645647,-1.2657652233,-1.019997243

C,0,0.8215059412,-0.5016142184,0.1969227343

C,0,-0.1001641914,0.4465931471,0.7997256855

C,0,-1.3705595492,0.5960545319,0.295772288

C,0,-1.893475903,-0.2388390838,-0.8656469143

C,0,-1.0996421712,-1.5245539332,-1.042601024

H,0,-1.3634073003,-2.2119222492,-0.2251103637

H,0,-1.3820188197,-2.0114694056,-1.9823819801

H,0,-1.8237588854,0.3677067242,-1.7869355314

C,0,-3.3477482394,-0.5666047785,-0.551886883

H,0,-3.3230170259,-1.041907749,0.4434598173

C,0,-4.2422269657,0.6650684149,-0.4058476673

C,0,-3.6263951031,1.6076480932,0.5759363827

C,0,-2.3115637031,1.5561786674,0.8856552799

H,0,-1.9282682894,2.2799184941,1.5983611657

C,0,-5.5573744995,-0.0073782219,0.0125123643

C,0,-5.6133744041,-1.2602695779,-0.9002626375

C,0,-4.1735213213,-1.4925245027,-1.4405891014

H,0,-4.0909512523,-1.1985140758,-2.4953503981

H,0,-3.8627688935,-2.538973394,-1.363182922

H,0,-5.9632167059,-2.1119376346,-0.3056815968

H,0,-6.3330800714,-1.1156473936,-1.7123753775

O,0,-5.4498143532,-0.3565093667,1.3984171191

H,0,-6.2015608798,-0.9181682805,1.6461142514

H,0,-6.4225668007,0.6542671023,-0.126528815

C,0,-4.4970057009,1.4153884883,-1.7281045963

H,0,-5.0122714277,2.3609720764,-1.5221600877

H,0,-3.5614569273,1.6428254723,-2.251211458

H,0,-5.1300082659,0.8324648929,-2.4071806607

C,0,0.3875576898,1.1019175397,2.0711062108

C,0,1.8497831657,1.5066785943,1.9617590759

C,0,2.7126682729,0.3664131459,1.5221928971

C,0,2.0935091372,-0.6517515468,0.7173451629

O,0,3.9436311131,0.3796721926,1.7897806103

H,0,1.9787830308,2.3000933025,1.206135803

H,0,2.2442745382,1.8911166559,2.9067577234

H,0,0.2781729397,0.3759651653,2.8927469775

H,0,-0.2042224363,1.9753367727,2.3431909242

H,0,0.6837962643,-0.6368290134,-1.8823411503

H,0,0.9583620751,-2.1955392618,-1.1140549353

Cl,0,3.1036081619,-1.9981609156,0.2701678443

O,0,2.1379348112,-4.5538032622,-1.6601799175

H,0,1.8131783644,-5.1885609039,-2.3166154213

H,0,2.388041549,-3.7483231734,-2.1667689559

O,0,2.8178595762,-2.2983968162,-3.2550384527

H,0,1.9464777287,-2.0038802039,-3.564917884

O,0,4.7079833542,2.8070810007,0.1508896651

H,0,4.5995613231,2.2729755937,0.9580090586

O,0,2.6010404353,4.5520709407,-0.2163383822

H,0,2.8855911337,5.2759417638,-0.7945503413

O,0,1.4465447086,2.3110034883,-1.6267235323

H,0,2.1785693909,1.6797374775,-1.7777605368

H,0,1.8452690795,3.0929067114,-1.1880633323

H,0,4.5261691202,2.1893042694,-0.5857769227

H,0,3.4023217073,4.0109253022,-0.0162053265

O,0,5.7595679279,-0.2101127748,0.1827480266

H,0,6.1581430331,-1.1016284947,0.1968879321

H,0,5.0241112788,-0.0977412841,0.9245357066

H,0,-4.2539827764,2.3792353048,1.0229770987

O,0,0.265078665,-4.3815386237,0.4483455815

H,0,0.93528979,-4.3706647492,-0.2731261673

H,0,0.4644369692,-5.1664277952,0.9807991207

O,0,-0.5878457573,-2.5320803339,2.4373863914

H,0,0.1228448377,-1.9054691462,2.6477737427

H,0,-0.2538253578,-3.0675487993,1.6839433627

H,0,-3.6674027527,-0.7684189921,2.4555635881

O,0,-2.9856176927,-0.9920817671,3.1170024787

H,0,-2.2754138985,-1.4732097012,2.647185575

H,0,3.1991056959,-1.5192176392,-2.8001992764

H,0,5.3072461894,-0.0465905028,-0.7084748575

O,0,-0.1050492311,4.3400849659,0.6827329965

H,0,-0.5416534939,4.0315061984,-0.1374645558

H,0,0.8400263986,4.4304926416,0.4472829882

O,0,-1.175136849,3.3168357677,-1.8209219082

H,0,-0.3821295208,2.740426229,-1.8448949467

H,0,-1.8961894878,2.7737733985,-1.4626961261

Cl,0,4.1127969417,0.5343782621,-2.2038714442

TS for C4-C12 chlorohydrin formation

SCF Done: E(RMN15) = -2608.90808884

Sum of electronic and thermal Free Energies= -2608.359909

C,0,0.9347483812,0.6378764569,1.0782240224

C,0,1.0722206259,-0.2613378411,-0.1087965098

C,0,0.0286639214,-0.8281761136,-0.7755254037

C,0,-1.3347510035,-0.512913164,-0.3628902883

C,0,-1.5337518355,0.3023599458,0.8981866238

C,0,-0.4169421152,1.3294936209,1.0751549789

H,0,-0.4572664461,2.0542107246,0.2471261218

H,0,-0.5736847797,1.8814299304,2.0087305774

H,0,-1.4594785123,-0.4140316666,1.7372216946

C,0,-2.9087174069,0.9497774256,0.9189822884

H,0,-2.9175642407,1.7335836418,0.1422333973

C,0,-4.0168331833,-0.0392764423,0.5513345364

C,0,-3.7450090704,-0.6148450309,-0.7936673426

C,0,-2.4093065149,-0.939979062,-1.1234083342

H,0,-2.252119985,-1.5132206,-2.0313373659

C,0,-5.294311898,0.7628619237,0.8540626621

C,0,-4.9382067821,1.4698009677,2.178561262

C,0,-3.388435545,1.5498929627,2.2453541079

H,0,-3.0045638453,0.9517361261,3.0823123735

H,0,-3.0295139947,2.5731686396,2.3854050674

H,0,-5.4094767654,2.4582634618,2.1899196921

H,0,-5.3441090573,0.9116816682,3.0280384099

O,0,-5.5172023898,1.7224418649,-0.1822136515

H,0,-6.2176366121,2.34033921,0.0813096724

H,0,-6.1720176582,0.1106742742,0.9391210805

C,0,-4.0530821817,-1.2917094045,1.4751637556

H,0,-4.8981019461,-1.9319612843,1.1981211973

H,0,-3.1329107166,-1.8824353277,1.4107973001

H,0,-4.1932659596,-0.9803714899,2.5156543268

C,0,0.3065018197,-1.6811480371,-1.9954624051

C,0,1.5707780402,-2.5235260419,-1.8133339814

C,0,2.7524050041,-1.7087320828,-1.4001286147

C,0,2.4656081514,-0.421752308,-0.6358704904

H,0,3.2179227898,-0.2868933244,0.1498409167

O,0,3.9151871668,-2.0149427201,-1.6410040241

H,0,1.3984225674,-3.2420707359,-0.9960986219

H,0,1.8261289145,-3.0966294314,-2.708644186

H,0,0.397042473,-1.0371624168,-2.8833021212

H,0,-0.5181113774,-2.3696204908,-2.1879492436

H,0,1.0568523599,0.0236845626,1.9866825135

H,0,1.752689844,1.3698987267,1.0783537372

Cl,0,2.7296037793,0.9289096316,-1.8318884589

H,0,3.8474972189,2.6592463381,0.6817422504

O,0,4.5821046211,2.020548887,0.8527482906

H,0,5.2895777457,2.5414856925,1.2649087383

O,0,5.3050524691,-1.9016491175,1.579595876

H,0,6.0244744136,-2.4983464475,1.8350168721

O,0,2.6269068414,-2.7233234805,1.4869592062

H,0,2.6020320901,-3.5393102261,0.9635985656

O,0,-0.1551549203,-2.6838880241,2.2982088812

H,0,-0.5912399441,-3.0391164943,1.494519746

H,0,0.7812288503,-2.552773262,2.0526285069

H,0,5.6419538035,-1.3212536767,0.856804147

H,0,3.5703752296,-2.4460818675,1.502679151

O,0,5.9039553672,-0.1347309542,-0.4981421956

H,0,5.3998961434,0.6441955756,-0.1770857911

H,0,5.3095834971,-0.6311983478,-1.0928479212

H,0,-4.5521194893,-1.104245323,-1.3351906417

O,0,0.2214873148,3.9917501148,-1.6884508312

H,0,0.8852104549,3.9818522962,-0.9575329324

H,0,0.6826296178,4.354105769,-2.4604384453

O,0,-1.3319084342,1.8773085021,-2.5921540411

H,0,-0.7629999632,1.0937639458,-2.6632247275

H,0,-0.767549798,2.5870323193,-2.2040201993

H,0,-4.5530119918,1.5715781642,-1.6234038597

O,0,-4.0062498596,1.0112334014,-2.220121708

H,0,-3.100142548,1.3990799251,-2.2547905902

O,0,3.8818230931,0.1750394447,2.9327231238

H,0,4.0408911489,0.8458577775,2.2352216406

H,0,4.3978070857,-0.6025689041,2.6413339734

O,0,-1.295460202,-3.9535080337,0.0217561217

H,0,-2.220969694,-3.9071220238,-0.3091397062

H,0,-1.1443296753,-4.8852030631,0.2435970742

O,0,-3.9218186885,-3.9871721779,-1.0277154326

H,0,-4.5375359064,-3.5304560321,-0.4321016196

H,0,-3.9023744118,-3.4490078028,-1.8353248782

Cl,0,2.3053195629,4.1742186711,0.6427244573

C4-C12 chlorohydrin

SCF Done: E(RMN15) = -2608.93401066

Sum of electronic and thermal Free Energies=   -2608.381209

C,0,-1.0764036933,-0.6463016439,1.0246993965

C,0,-1.2414569764,0.298540589,-0.1313248539

C,0,-0.2152420918,0.9150389153,-0.765149961

C,0,1.1813839718,0.6221355528,-0.3764470535

C,0,1.3853672384,-0.1279256015,0.9302889895

C,0,0.3279373449,-1.2239419027,1.064843582

H,0,0.4451917618,-1.9258322913,0.2261674908

H,0,0.4917943449,-1.7889822332,1.9909533237

H,0,1.2417187124,0.5929439912,1.7538334487

C,0,2.7881626675,-0.7096503606,1.0197512513

H,0,2.8312083681,-1.5685279257,0.3281661384

C,0,3.8588405262,0.2904052033,0.5682263116

C,0,3.6421452179,0.6076240214,-0.9091372123

C,0,2.2082223591,0.9639405171,-1.1867293973

H,0,2.026660335,1.4747237415,-2.1302695694

C,0,5.1541426579,-0.4340939668,0.970529586

C,0,4.821089551,-1.0184648587,2.3529111961

C,0,3.2763740169,-1.1767523507,2.3994619372

H,0,2.844714684,-0.5395941212,3.1825341411

H,0,2.9708167784,-2.2053138014,2.613579055

H,0,5.350405265,-1.9685631944,2.4765885592

H,0,5.1756981349,-0.3448259184,3.1396845333

O,0,5.3976756355,-1.5067040451,0.0296296492

H,0,6.3316399465,-1.7700983749,0.0487589327

H,0,6.0231311611,0.2322739434,0.971981676

C,0,3.8072170567,1.612172616,1.3515703356

H,0,4.6652809369,2.2394344087,1.0783359329

H,0,2.8900408104,2.1745729054,1.1414688324

H,0,3.8508506116,1.4337150292,2.4311634674

C,0,-0.517883244,1.853026535,-1.9135652406

C,0,-1.7813606312,2.6712445062,-1.6322742652

C,0,-2.9536289385,1.819745318,-1.2598275552

C,0,-2.6348090622,0.4596872216,-0.653067518

H,0,-3.3966670031,0.2010295411,0.0907535924

O,0,-4.1211665635,2.1643610517,-1.4037961407

H,0,-1.5977087468,3.3143485458,-0.754980219

H,0,-2.0605179342,3.322114038,-2.4656474914

H,0,-0.6328179528,1.286429848,-2.8486886255

H,0,0.3077937048,2.5525754189,-2.0684113534

H,0,-1.3049771295,-0.1033137721,1.9570435174

H,0,-1.8174024264,-1.4519310253,0.9432278513

Cl,0,-2.8892285188,-0.7233638825,-2.0346924668

H,0,-3.3055333523,-3.1062546074,0.1459311352

O,0,-4.1481587688,-2.7286440377,0.4931883305

H,0,-4.6580053935,-3.4840447031,0.8261475864

O,0,-5.5097551592,1.2508433613,1.4168211175

H,0,-6.3379281459,1.4711980181,1.8691431121

O,0,-3.0393466978,2.5592209114,1.7577594974

H,0,-3.0943561486,3.3470515843,1.1940039966

O,0,-0.1831039183,2.8207704553,2.2478941334

H,0,0.2290137871,3.1833650197,1.4355178025

H,0,-1.0896762024,2.5554126746,2.0013515276

H,0,-5.7285477561,0.6071682907,0.6997937661

H,0,-3.8866719768,2.0805662551,1.6226574862

O,0,-5.9373864888,-0.7310864089,-0.4665493624

H,0,-5.2846606885,-1.4294426869,-0.2358367955

H,0,-5.6807175137,-0.3957537422,-1.3404049123

H,0,4.3213005847,1.3940957088,-1.2549530763

O,0,1.6621206383,-4.1394340632,-0.5108582733

H,0,0.6784481978,-4.2025704588,-0.4494414235

H,0,1.9582027506,-4.9782988223,-0.8975190791

O,0,2.0280067551,-2.0600133962,-2.2487163968

H,0,1.203625339,-1.5478284506,-2.1697848408

H,0,1.937894509,-2.8220437977,-1.6139483228

H,0,4.6870395282,-1.0948730676,-1.2401522867

O,0,3.9761280812,-0.5639792787,-1.75887802

H,0,3.1433360429,-1.1993350408,-1.9460645788

O,0,-4.108450829,-0.8902557417,2.735138795

H,0,-4.0396964074,-1.5119120248,1.9814713336

H,0,-4.5665278854,-0.1030770087,2.3764543127

O,0,0.9866739436,4.2098135453,0.0589675232

H,0,1.852641363,4.1163507977,-0.398748724

H,0,0.9635189431,5.1192379301,0.3938110786

O,0,3.4645980297,4.0993240916,-1.2883146746

H,0,4.0976224998,3.6764461023,-0.6854483576

H,0,3.380344652,3.4880612553,-2.0377182568

Cl,0,-1.4953391994,-4.2460588526,-0.4174989581

C11-C12 epoxide product complex

SCF Done: E(RMN15) = -2608.92181428

Sum of electronic and thermal Free Energies= -2608.366239

C,0,-0.7720987444,-0.5362558699,1.3744748292
C,0,-1.0773221259,0.2522765706,0.1326123586
C,0,0.0460978199,0.8186175888,-0.6273003383
C,0,1.32547547,0.6444593351,-0.1949797899
C,0,1.6783157238,-0.232465571,0.9988171154
C,0,0.5693984897,-1.2404126193,1.2731440749
H,0,0.5408257448,-1.9674648818,0.449417125
H,0,0.7832586723,-1.7919444881,2.1957395687
H,0,1.7914862426,0.4071685656,1.8918540749
C,0,3.0042157012,-0.9372303912,0.7258707615
H,0,2.8768396016,-1.5268589273,-0.195872126
C,0,4.1689433101,0.0285324314,0.4758113433
C,0,3.8658493281,0.9105948736,-0.7052411402
C,0,2.4547016344,1.2620688176,-0.9656992537
H,0,2.2613641198,2.2264483652,-1.4298672631
C,0,5.3480342165,-0.9430224127,0.3489862168
C,0,5.0710535223,-1.9785707294,1.4625174961
C,0,3.5575110481,-1.8830779967,1.7957806588
H,0,3.4008183389,-1.4562469088,2.7957475371
H,0,3.0616222419,-2.858841859,1.7774889307
H,0,5.3552189008,-2.9737551769,1.1019895301
H,0,5.6873292657,-1.7668806134,2.3426182662
O,0,5.3024610747,-1.5371038453,-0.9517170297
H,0,5.978433299,-2.2315849418,-0.9995106942
H,0,6.3121229487,-0.4333681154,0.4812819067
C,0,4.4422192576,0.9713072457,1.6575006565
H,0,5.2830842003,1.6338429997,1.416124792

H,0,3.5713162303,1.5974153903,1.8795809621

H,0,4.7029710708,0.4158071658,2.5648296654

C,0,-0.3245214305,1.48079779,-1.935695751

C,0,-1.6251198094,2.2728522531,-1.8399007955

C,0,-2.739041492,1.453310642,-1.2494813235

C,0,-2.3714502771,0.4727796456,-0.2366279395

H,0,-3.184568744,-0.0112955965,0.3041377772

O,0,-3.925603347,1.6812804427,-1.5426151565

H,0,-1.4946583449,3.1371801113,-1.1696491059

H,0,-1.9395418522,2.6556020677,-2.8150503115

H,0,-0.4599040812,0.6841383268,-2.6835766223

H,0,0.4722140406,2.1266027882,-2.3103187807

H,0,-0.7428091932,0.1774121896,2.2147954091

H,0,-1.5893041255,-1.2356723467,1.5764166896

Cl,0,-5.2491121294,-1.6730602007,-2.7741269748

H,0,-3.2188267395,-2.8259652529,0.8266706944

O,0,-3.933249744,-2.5109251206,1.4246061273

H,0,-4.4001056922,-3.3056420263,1.7265927288

O,0,-5.092368243,1.6554794185,1.491385328

H,0,-5.9191396786,1.9946996556,1.8677626546

O,0,-3.346624713,3.6842790491,0.9587359318

H,0,-3.4313944455,3.9446185087,0.026485942

O,0,-0.9642227889,3.2288592101,1.562372625

H,0,-0.2821445666,3.6522344855,0.8830284371

H,0,-1.9710745488,3.407597841,1.2968783589

H,0,-5.3412931687,0.910860498,0.8873023572

H,0,-3.9662865501,2.920302305,1.0956126999

O,0,-5.7560324396,-0.6848259396,0.1818020196

H,0,-5.1553638847,-1.31222994,0.6400623745

H,0,-5.6099769508,-0.8665201135,-0.7770822182

H,0,4.6275927909,1.6436269444,-0.9795758088

O,0,1.1688909168,-3.8477269304,-1.2744612763

H,0,0.1930338293,-3.8537543983,-0.9875911723

H,0,1.344904253,-4.6408084634,-1.8145084868

O,0,1.5397427579,-1.7977421124,-2.5895472721

H,0,0.6974513462,-1.312754147,-2.6430070461

H,0,1.3619625782,-2.9507424774,-1.8436819672

O,0,3.1851725002,0.3558697295,-1.8368416382

H,0,2.203249425,-1.1605009413,-2.2364760828

O,0,-3.7620727165,-0.230315515,3.2048001718

H,0,-3.7503199159,-1.0141168218,2.6192838126

H,0,-4.2009908766,0.4730065519,2.6838634574

O,0,0.6731074562,4.1934209554,-0.0729483854

H,0,1.6131914004,4.1857018506,0.244439119

H,0,0.46375021,5.1112170539,-0.309675704

O,0,3.272397136,4.2076270244,0.7799558956

H,0,3.3492982497,3.8941349593,1.6952726757

H,0,3.8335287735,3.6129536717,0.2556806819

Cl,0,-1.7066979928,-3.7556454351,-0.5094676506

H,0,-0.7931660652,3.5768940179,2.4557794561

Literature Cited

1. Clesceri, L. S.; Eaton, A. D.; Greenberg, A. E. , *Standard Methods for the Examination of Water and Wastewater*. 1998.
2. Schäfer, A.; Horn, H.; Ahlrichs, R., Fully optimized contracted Gaussian basis sets for atoms Li to Kr. *J. Chem. Phys.* **1992,** *97*, 2571-2577.
3. Weigend, F.; Ahlrichs, R., Balanced basis sets of split valence, triple zeta valence and quadruple zeta valence quality for H to Rn: Design and assessment of accuracy. *Phys. Chem. Chem. Phys.* **2005,** *7*, 3297-305.
4. Becke, A. D., Density-functional thermochemistry. III. The role of exact exchange. *J. Chem. Phys.* **1993,** *98*, 5648-5652.
5. Lee, C.; Yang, W.; Parr, R. G., Development of the Colle-Salvetti correlation-energy formula into a functional of the electron density. *Phys. Rev. B* **1988,** *37*, 785-789.
6. Neese, F.; Wennmohs, F.; Hansen, A.; Becker, U., Efficient, approximate and parallel Hartree–Fock and hybrid DFT calculations. A 'chain-of-spheres' algorithm for the Hartree–Fock exchange. *J. Chem. Phys.* **2009,** *356*, 98-109.
7. Cossi, M.; Rega, N.; Scalmani, G.; Barone, V., Energies, structures, and electronic properties of molecules in solution with the C-PCM solvation model. *J. Comput. Chem.* **2003,** *24*, 669-681.
8. Neese, F., The ORCA program system. *WIREs Computational Molecular Science* **2012,** *2* (1), 73-78.
9. Bruhn, T.; Schaumlöffel, A.; Hemberger, Y.; Bringmann, G., SpecDis: Quantifying the Comparison of Calculated and Experimental Electronic Circular Dichroism Spectra. *Chirality* **2013,** *25*, 243-249.
10. Wammer, K. H.; Anderson, K. C.; Erickson, P. R.; Kliegman, S.; Moffatt, M. E.; Berg, S. M.; Heitzman, J. A.; Pflug, N. C.; McNeill, K.; Martinovic-Weigelt, D.; Abagyan, R.; Cwiertny, D. M.; Kolodziej, E. P., Environmental Photochemistry of Altrenogest: Photoisomerization to a Bioactive Product with Increased Environmental Persistence via Reversible Photohydration. *Environ. Sci. Technol.* **2016,** *50*, 7480-8.
